# Studies toward the Caged Sesquiterpenoid Daphnepapytone A

**DOI:** 10.1021/acs.joc.6c00079

**Published:** 2026-05-15

**Authors:** Kamar Shakeri, Letizia Lanfredi, Christian Zachau, Luisa Eichner, Iulia Bîlici, Esra Dural, Jan-H. Dickoff, Manuela Weber, Mathias Christmann

**Affiliations:** Department of Biology, Chemistry, Pharmacy, Institute of Chemistry and Biochemistry, Freie Universität Berlin, Takustraße 3, 14195 Berlin, Germany

## Abstract

Daphnepapytone A is an unprecedented caged guaiane sesquiterpenoid with α-glucosidase inhibitory activity. We describe progress toward its total synthesis using an α-santonin-type rearrangement strategy. In the course of these studies, a range of guaiane frameworks, including daphbolides A and B, were prepared. However, attempted C9 oxidations of the guaiane proved problematic. Notably, some decalin substrates underwent unexpected rearrangements to yield phenolic products, revealing novel reactivity patterns in this compound class.

## Graphical abstract

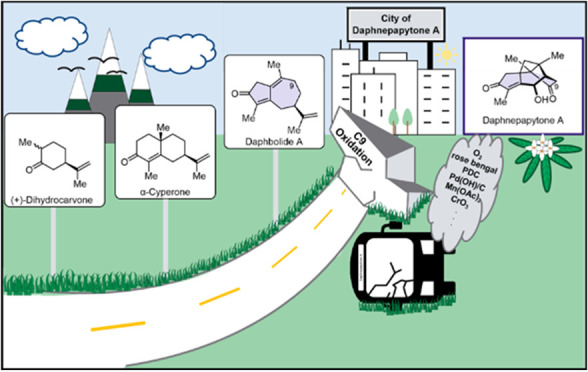

## Introduction

In 2022, Dai, Zhao, and co-workers reported daphnepapytone A (**1**), a structurally unique guaiane-type sesquiterpenoid characterized by an unprecedented caged 5/6/4/5 carbon skeleton bearing five stereocenters, three of which are quaternary (Figure 1).^1^

**Figure 1:**
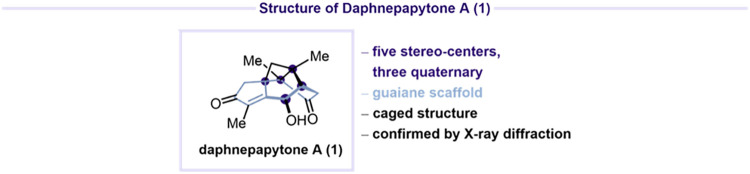
Structure of daphnepapytone A (**1**).

Daphnepapytone A (**1**) was isolated from *Daphne papyracea*, and its structure was unambiguously characterized by X-ray diffraction. *Daphne* is a genus in the plant family Thymelaeaceae, native to Asia, Europe, and North Africa, comprising 95 species of deciduous and evergreen shrubs.^2^ This genus has long been recognized for its anti-inflammatory, cytotoxic, and antiviral bioactivities and has been used in traditional Asian medicine for the treatment of diabetes and rheumatic pain.^1,3^ Daphnepapytone A (**1**) has demonstrated biological activity as an α-glucosidase inhibitor (IC_50_ = 159 ± 2.1 μM).^1^ Only 5.3 mg of this compound was isolated from 12.3 kg of air-dried *Daphne papyracea* stems, prompting chemists to develop synthetic methods to access larger quantities of this natural product.

Dai and Zhao proposed a biosynthesis leading through oleodaphnone (**2**), which undergoes a [2 + 2]-cycloaddition for the construction of the cyclobutane moiety between C1, C10, C11, and C12 with a subsequent allylic oxidation at C6. Alternatively, the two steps were proposed to occur in either order (Scheme 1A). In 2025, Nay,^4^ Li and She,^5^ Stoltz,^6^ and Hanson^7^ have independently shown four total syntheses of **1** using a Pauson–Khand reaction for the construction of the guaiane scaffold (Scheme 1B). Breit^8^ and Liang and Banwell^9^ published two further approaches toward the natural product in 2026.

**Scheme 1:**
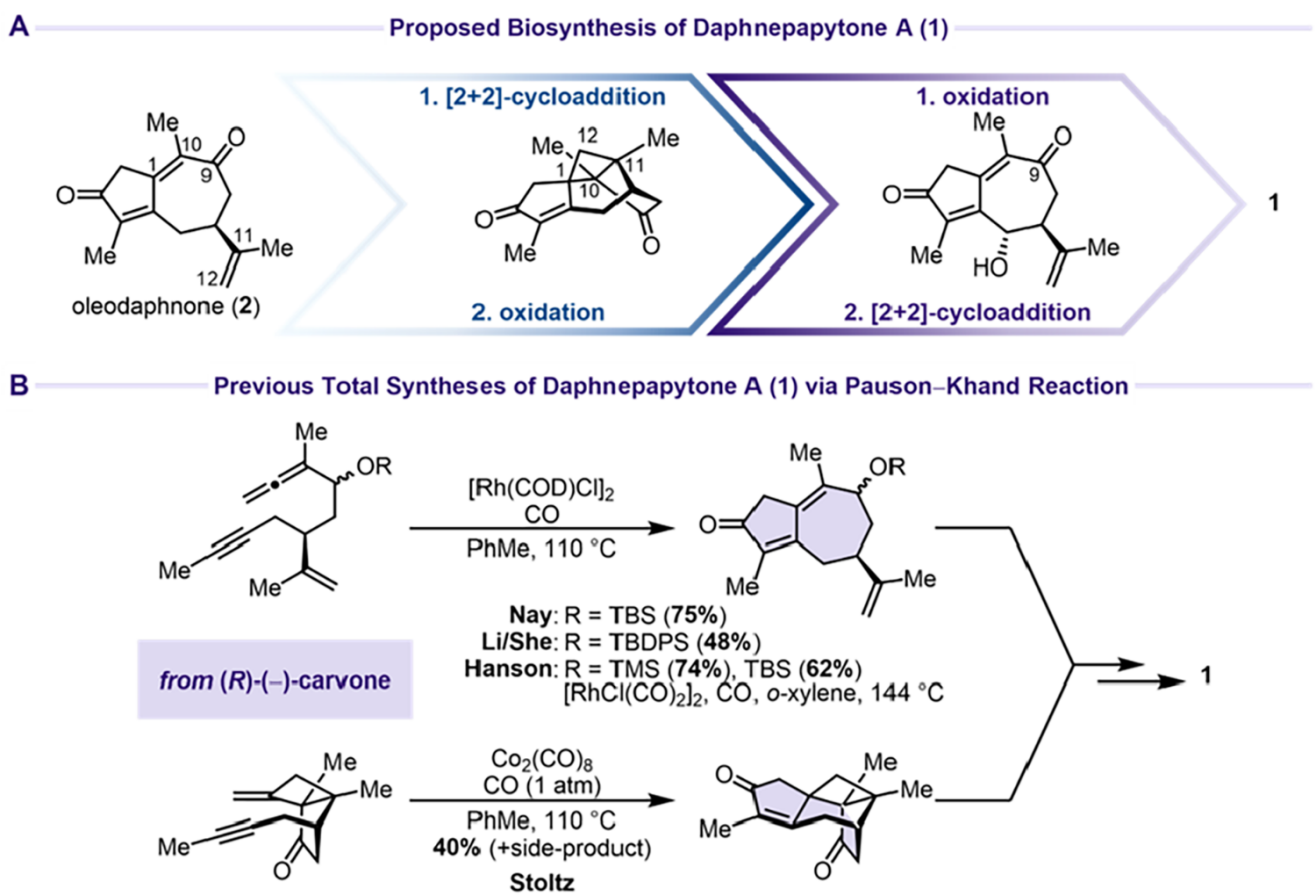
Biosynthesis and Previous Work on **1** ^a^

a (A): Proposed biosynthesis of daphnepapytone A (**1**), (B): Previous total syntheses of daphnepapytone A (**1**) via Pauson–Khand reaction. COD = 1,5-cyclooctadiene, TBS = *tert*-butyldimethylsilyl, TBDPS = *tert*-butyldiphenylsilyl, TMS = trimethylsilyl.

As this transformation relies on carbon monoxide, a highly toxic gas, for the introduction of the cyclopentenone carbonyl moiety, we were intrigued to explore an alternative, inherently safer approach to access the guaiane core. Among the diverse repertoire of guaiane-forming strategies, including metathesis,^10^ cyclopropanation/oxy-Cope sequences,^11^ Dieckmann condensations,^12^ and Nazarov cyclizations,^13^ the α-santonin rearrangement stood out to us as particularly attractive. This photochemical transformation converts readily available 5,6,7,8-tetrahydronaphthalen-2­(4a*H*)-one derivatives into the corresponding 4,5,6,7,8,8a-hexahydroazulen-2­(1*H*)-ones under UV-light irradiation. The operational simplicity, absence of hazardous reagents, and direct access to the characteristic guaiane skeleton rendered the α-santonin rearrangement the most practical and conceptually elegant solution for our synthetic design. This well-established transformation has moreover proven successful in numerous synthetic applications.^14–21^

Inspired by this transformation, we planned the synthesis of **1** with an α-santonin rearrangement as the key step. Herein, we describe the various challenges encountered during this synthetic route. A preliminary version of this work was previously disclosed as a preprint in ChemRxiv.^22^

## Results and Discussion

The bioinspired retrosynthetic analysis began with the disconnection of the C6–O bond, which would be installed through an allylic oxidation (Scheme 2). The pivotal transformation to construct the caged framework of daphnepapytone A (**1**) was envisioned via [2 + 2]-cycloaddition to forge the cyclobutane ring spanning C1, C10, C11, and C12. These two operations could potentially be executed in reverse order. Both disconnections converge on oleodaphnone (**2**), an intermediate previously proposed by Dai and Zhao.^1^ Compound **2** was further traced to daphbolide A (**3**), a diterpene isolated from *Daphne bholua* by Bai and Song in 2022,^23^ which would require allylic oxidation at C9.

**Scheme 2:**
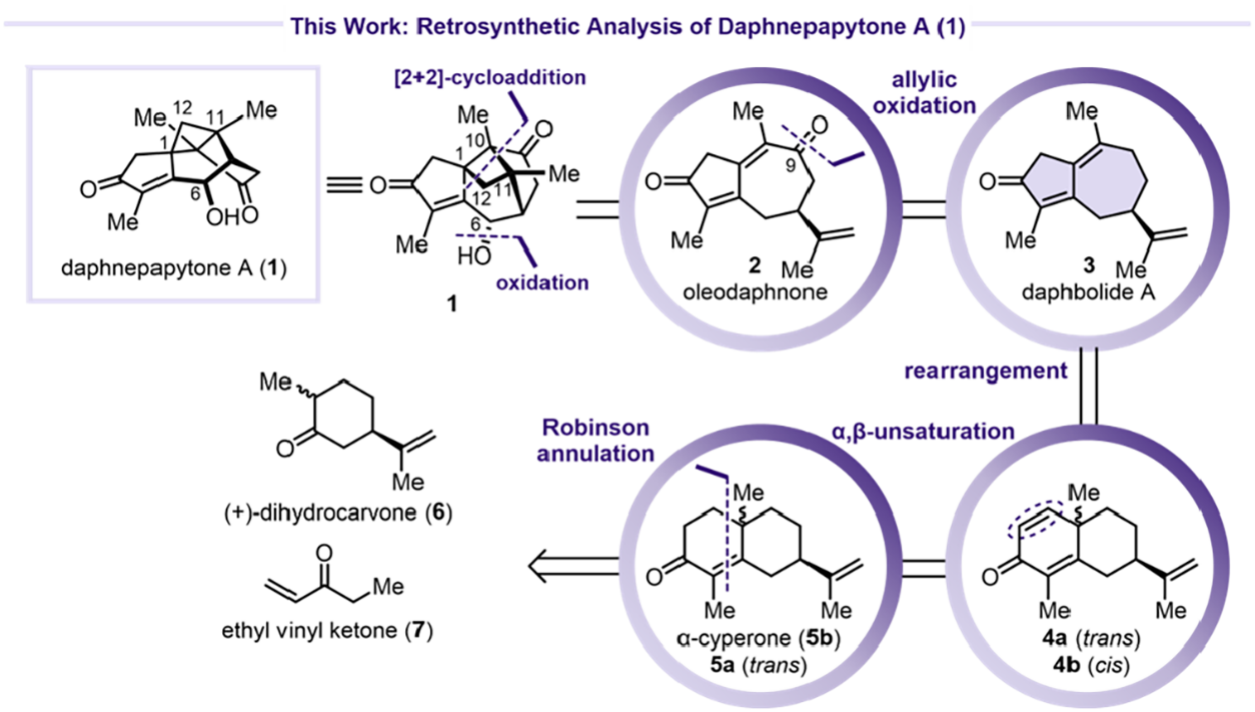
Retrosynthetic Analysis of **1**

Daphbolide A (**3**) could be accessed from dienones **4a** and **4b** via an α-santonin rearrangement. Dienones **4a** and **4b** would then be approachable through annulation of (+)-dihydrocarvone (**6**) and ethyl vinyl ketone (**7**). Starting the synthesis, Robinson annulation between **6** and **7** delivered 10-*epi*-α-cyperone (**5a**) and α-cyperone (**5b**) in 90% isolated yield (**5a**/**5b** = 3:1) (Scheme 3A).^18^ Introduction of the α,β-unsaturation was initially attempted through an α-bromination/elimination sequence. This approach resulted in a complex product mixture (not including any isolatable byproduct) that did not contain the desired dienones **4a** and **4b**. Consequently, a single-step oxidation using DDQ was explored, which afforded dienones **4a** and **4b** in 20% yield (brsm) with a diastereomeric ratio of 3:1.^24^ Nevertheless, due to the low yield, an alternative α-selenylation/oxidation sequence was employed to afford **4a** and **4b** in 69% combined yield (**4a**/**4b** = 4:1). With both dienones in hand, the key photochemical rearrangement for constructing the guaiane core, as reported by Pedro and co-workers, was undertaken.^25^ Compounds **4a** and **4b** were irradiated with UV light (365 or 370 nm) in acetic acid/acetic anhydride (10:1, v/v) for 6 h to afford the guaiane-core products **8a** and **8b** in 54% combined yield with an 8:1 ratio favoring **8a**. Treatment of **8a** with DBU in refluxing acetonitrile furnished daphbolide A (**3**) in 65% isolated yield (78% brsm) (Scheme 3B). Alternatively, reaction of **8a** with Ba­(OH)_2_·8H_2_O in methanol provided both the hydrolysis product **9** (24%) and daphbolide A (**3**) (16%). Compound **9** was isolated as a crystalline solid, and its structure was unambiguously confirmed by single-crystal X-ray diffraction analysis,^26^ thereby establishing the absolute configuration of **8a** as well. Attempts to promote elimination under acidic conditions with weaker acids, including phosphorous acid (p*K*_a_ = 2), oxalic acid (p*K*_a_ = 1.23), and trifluoroacetic acid (p*K*_a_ = 0.23), gave no conversion. Stronger acids capable of eliminating acetic acid, such as sulfuric acid (p*K*_a_ = −1.9) and methanesulfonic acid (p*K*_a_ = −3), instead caused isomerization of the isopropenyl double bond to afford **10** in 74% yield.

**Scheme 3:**
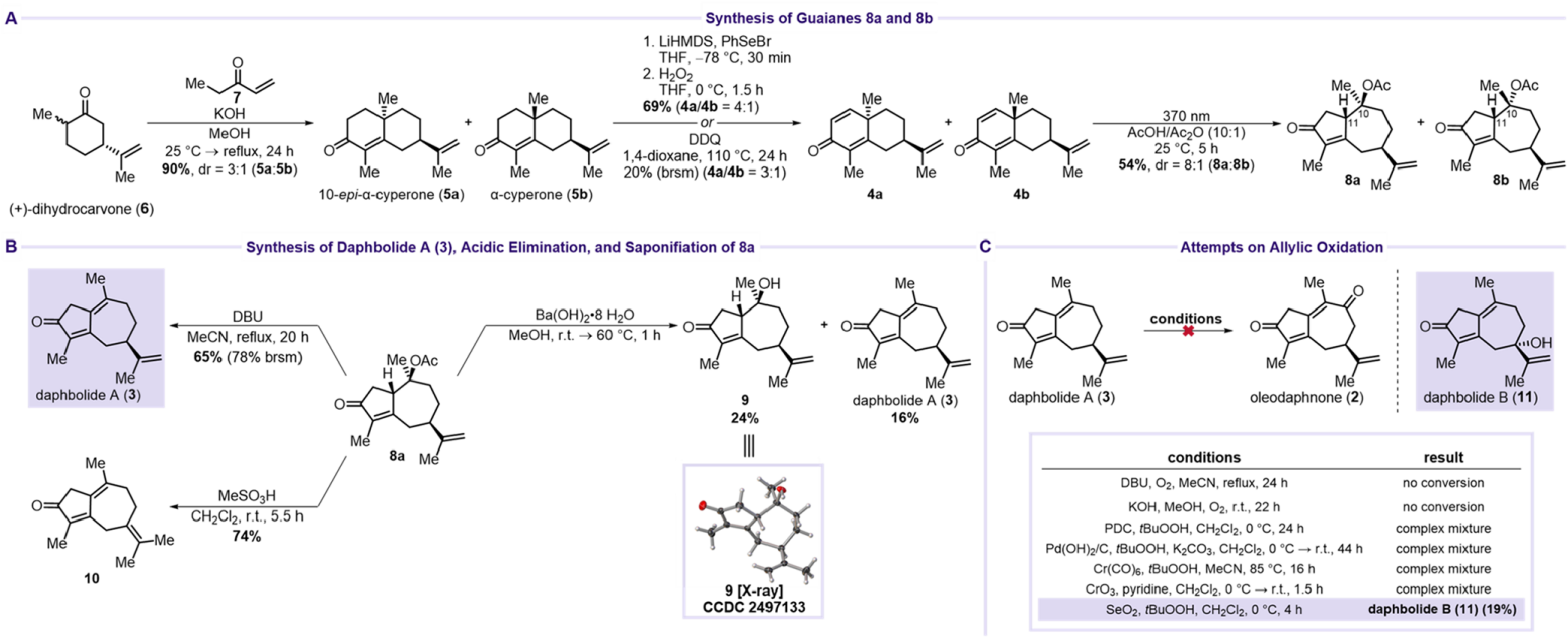
Synthesis of Daphbolide A (**3**) and B (**11**)­^a^

a (A): Synthesis of guaianes **8a** and **8b**, (B): Synthesis of daphbolide A (**3**), acidic elimination and saponification on **8a**, (C): Attempts on allylic oxidation. LiHMDS = lithium bis­(trimethylsilyl)­amide, DDQ = 2,3-dichloro-5,6-dicyano-1,4-benzoquinone, DBU = 1,8-diazabicyclo[5.4.0]­undec-7-ene, PDC = pyridinium dichromate.

Having secured access to daphbolide A (**3**), we next targeted C9 oxidation to furnish oleodaphnone (**2**). Despite extensive screening of allylic oxidation conditions (see the SI), no successful conversion was achieved (Scheme 3C). Reddy’s DBU/O_2_ protocol for dienone oxidation and variations,^27,28^ as well as various transition-metal-catalyzed^29–34^ and photocatalyzed^35–37^ allylic oxidation approaches, all failed to deliver oleodaphnone (**2**). Interestingly, Riley conditions led to oxidation at the tertiary C7 position instead, affording daphbolide B (**11**) in 19% yield.

A major challenge for allylic C9 oxidation of daphbolide A (**3**) was the presence of seven different allylic positions (Scheme 4A). We therefore revised our strategy to install the allylic oxidation at C6 early in the synthesis rather than at the end. This approach would simultaneously block C7, preventing formation of the undesired C6 oxidized analog of **11**, while the remainder of the synthetic route would proceed as described above. In contrast to the strategies by Nay,^4^ Li and She,^5^ Stoltz,^6^ and Hanson,^7^ who faced a demanding late-stage C6 oxidation typically requiring an oxidation/reduction two-step sequence, we envisioned that introducing the C6 oxygen functionality at an early stage would offer a more streamlined and controllable route. Early installation of this oxidation state not only avoids challenging late-stage functionalization but also provides a useful handle to guide downstream transformations and stereochemical outcomes. Conveniently, Baran and co-workers had already prepared a suitable precursor, **12**, bearing the requisite C6 stereocenter in their synthesis of (−)-thapsigargin (Scheme 4B).^18^ Compound **12** was prepared by Robinson annulation of (+)-dihydrocarvone (**6**) with ethyl vinyl ketone (**7**), followed by γ-oxidation by stirring the reaction mixture under an oxygen atmosphere to afford **12** as a single diastereomer in 48% yield (Scheme 5A). Introduction of α,β-unsaturation into **12** via an α-bromination/elimination sequence afforded dienone **13** in 51% yield. Alternatively, DDQ-mediated oxidation provided **13** in 47% yield. Although slightly lower yielding, the DDQ protocol was operationally more convenient and time-efficient. The structure of **13** was confirmed by single-crystal X-ray diffraction. Attempted rearrangement of **13** led to ether **14** in 20% yield (22% brsm), consistent with observations by Pedro and co-workers.^25^ Compound **14** likely forms via intramolecular attack of the C6 hydroxyl group at C10 during the rearrangement process. To prevent this side reaction, the C6 hydroxyl was protected as the TBS ether to afford **15** in 73% yield. Photochemical rearrangement of **15** under UV-light irradiation (365 or 370 nm) in a mixture of acetic acid and acetic anhydride (10:1, *v*/*v*) furnished guaiane **16** in 33% yield. Additionally, phenol derivative **17** was isolated as a crystalline byproduct in 35% yield. The formation of **17** can be rationalized by the following mechanism (Scheme 5B). Protonation of **13** might form a tertiary carbocation that undergoes Wagner–Meerwein rearrangement with methyl migration from C10 to C5. Subsequent cleavage of the O–TBS bond by acetate, followed by oxidation to the carbonyl, C5–C6 bond cleavage, and aromatization, furnishes **17** after isomerization.

**Scheme 4:**
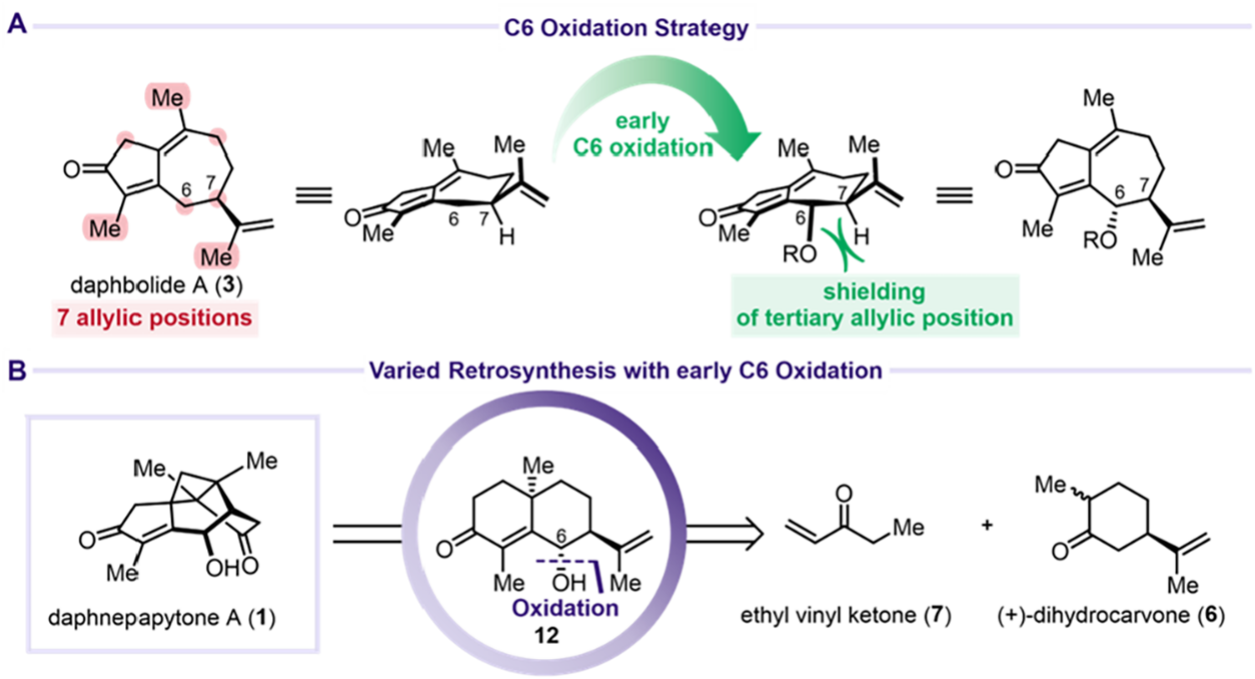
Variation of the Retrosynthesis Implementing Early C6 Oxidation^a^

a (A): C6 oxidation strategy, (B): Varied retrosynthesis with early C6 oxidation.

**Scheme 5:**
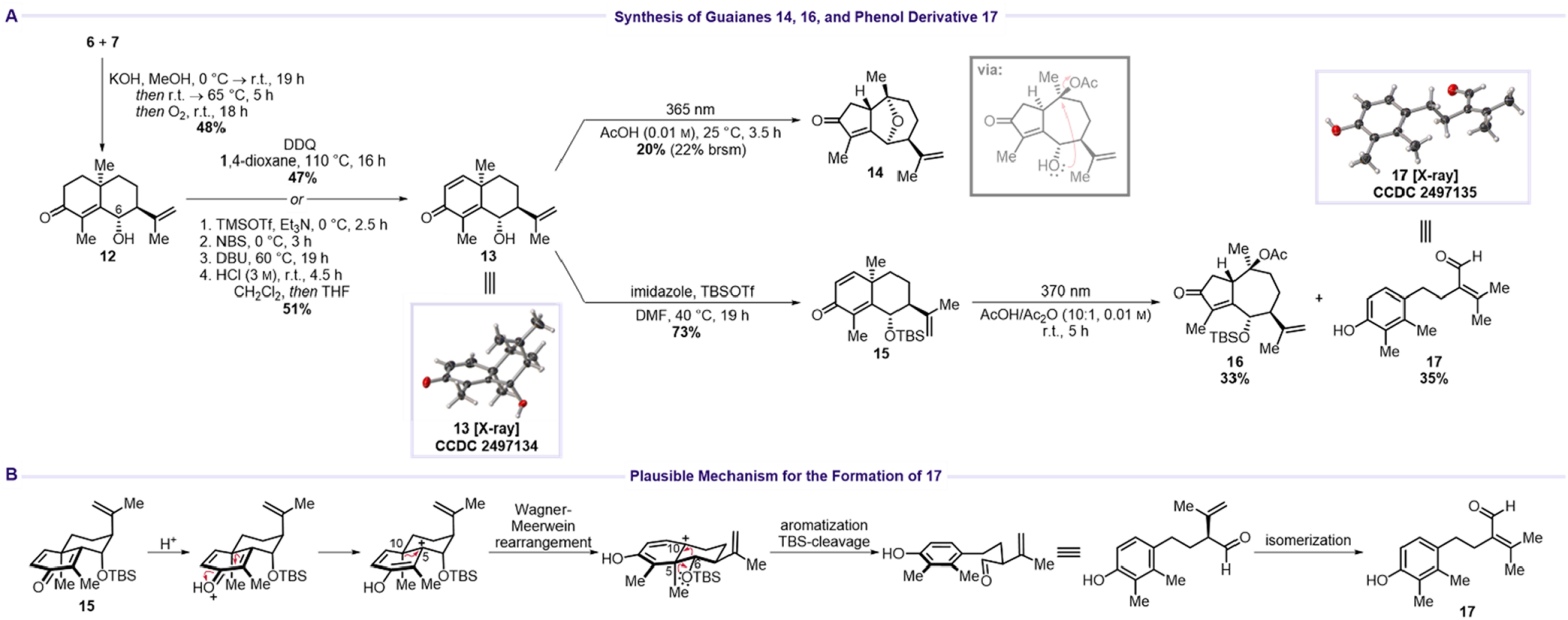
Early C6 Oxidation Route^a^

a (A): Synthesis of guaianes **14**, **16**, and phenol derivatives **17**, (B): Plausible mechanism for the formation of **17**. DDQ = 2,3-dichloro-5,6-dicyano-1,4-benzoquinone, TMSOTf = trimethylsilyl trifluoromethanesulfonate, NBS = *N*-bromosuccinimide, DBU = 1,8-diazabicyclo[5.4.0]­undec-7-ene, TBSOTf = *tert*-butyldimethylsilyl trifluoromethanesulfonate, DMF = *N*,*N*-dimethylformamide.

The structure of **17** was confirmed by single-crystal X-ray diffraction. Next, elimination of acetic acid from guaiane **16** using DBU in refluxing acetonitrile afforded dienone **21** in 35% yield (44% brsm) (Scheme 6). Unfortunately, allylic C9 oxidation of **21** to **22** also proved to be unsuccessful.^29,31,33,38–40^

**Scheme 6:**
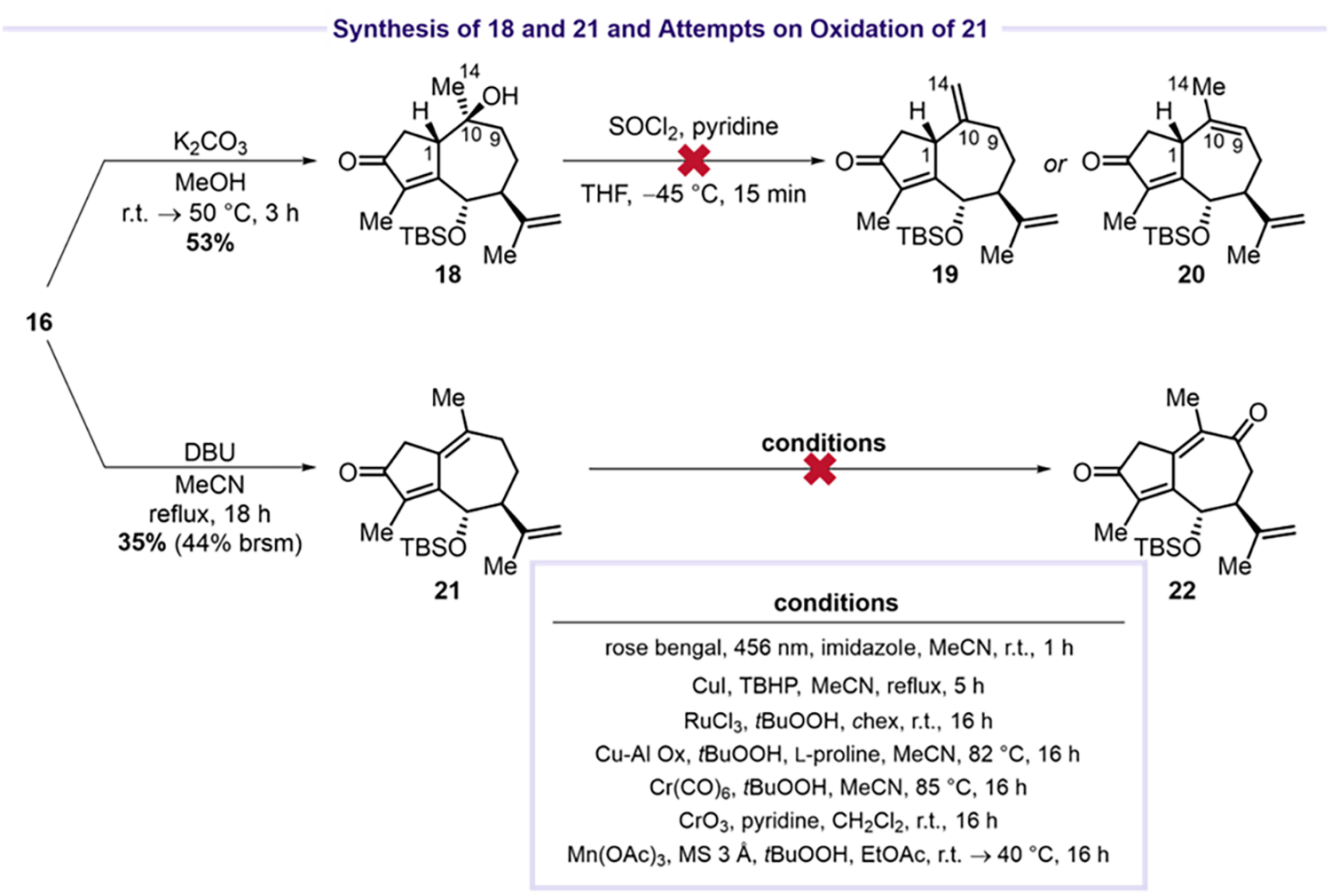
Synthesis of **18** and **21** and Attempted Oxidation of **21** ^a^

a DBU = 1,8-diazabicyclo[5.4.0]­undec-7-ene.

We therefore adopted an alternative strategy involving allylic oxidation with allylic transposition, as demonstrated by Edgar and Greene in their synthesis of oxoisodehydroleucodin.^15^ This approach required installation of the C9=C10 double bond present in **20**, which could potentially be accessed either by direct elimination to **20** or through elimination to **19** followed by isomerization (Scheme 6).^41^ Saponification of **16** using K_2_CO_3_ in methanol furnished alcohol **18** in 53% yield. Attempted elimination with SOCl_2_ resulted in complex product mixtures (not including any isolatable byproduct).

An alternative approach to minimize the number of competing allylic positions employed α-santonin (**23**) as the starting material. This substrate already contains the dienone moiety and masks the isopropenyl group within its lactone ring, which would reduce allylic site complexity (Figure 2).

**Figure 2:**
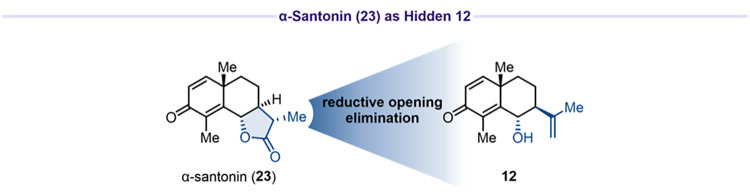
α-Santonin (**23**) as as a masked precursor to **12** carrying a protected isopropenyl group.

The isopropenyl group might be accessible from the lactone via reductive opening with subsequent elimination. Photochemical rearrangement of α-santonin (**23**) under UV-light irradiation (365 or 370 nm) in either a water/acetic acid (6:1, v/v) mixture or neat acetic acid afforded guaianolides **24** and **25** in 18% and 25% yield, respectively (Scheme 7A). Treatment of alcohol **24** and acetate **25** with sulfuric acid furnished diene **26** in 84% and 98% yields, respectively. Alternatively, elimination of acetic acid from **25** using LDA provided **26** in 97% yield. Interestingly, when **25** was heated under reflux with DBU in MeCN, decarboxylation occurred to afford **27** in 28% yield. Subjecting dienone **26** to various allylic oxidation conditions failed to provide the desired C9 oxidized product **28** (Scheme 7B). Instead, oxidative cleavage of the C1=C10 double bond occurred when **26** was treated with CrO_3_ or Mn­(OAc)_3_/TBHP, affording trienone **29** in up to 29% yield, matching the observations by Hanson and co-workers.^7^ Attention then turned to preparing a suitable precursor for Babler–Dauben oxidation (Scheme 7C). Epoxidation of dienone **26** furnished **30** in 64% yield, which was subsequently treated with various Brønsted and Lewis acids. Unfortunately, none of these conditions yielded the desired alcohol **31**.

**Scheme 7:**
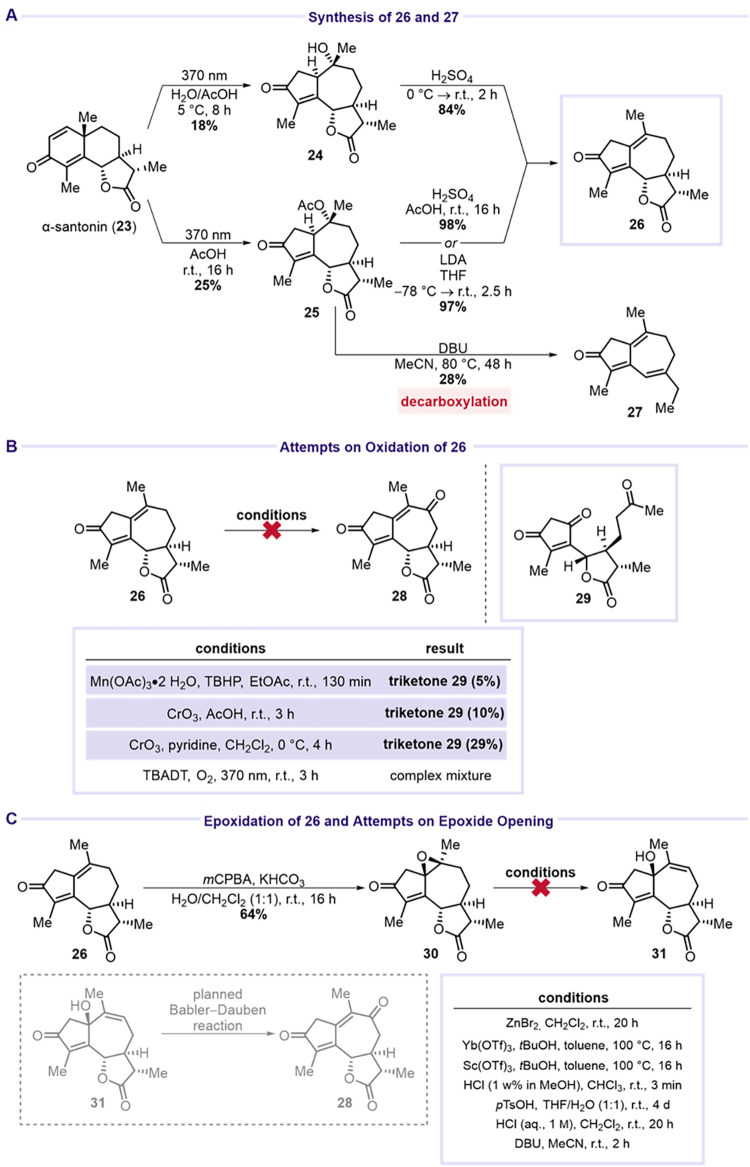
Attempts toward **28** Starting from α-Santonin (**23**)­^a^

a (A): Synthesis of **26** and **27**, (B): Attempts on oxidation of **26**, (C): Epoxidation of **26** and attempts on epoxide opening. LDA = lithium diisopropylamide, DBU = 1,8-diazabicyclo[5.4.0]­undec-7-ene, TBHP = *tert*-butyl hydroperoxide, TBADT = tetra-*n*-butylammonium decatungstate, *m*CPBA = *meta*-chloroperoxybenzoic acid, *p*TsOH = 4-methylbenzene-1-sulfonic acid.

Since allylic oxidation at C9 of the guaiane scaffold proved unsuccessful, we revised our strategy to introduce the C9 oxidation earlier in the synthetic route, returning to decalin intermediates **32a** and **32b** (Scheme 8). Epoxidation of (*R*)-(−)-carvone (**33**) afforded **34a** and **34b** in 89% combined yield (**34a**/**34b** = 1:16) (Scheme 9A).^42^ Redox-neutral epoxide opening of **34b** with aqueous NaOH furnished dienone **35** in 90% yield.^43^ Michael addition of ethyl vinyl ketone (**7**) to **35** provided **36** in 71% yield (dr = 1:2.8).^44^ Subsequent aldol condensation using either l-phenylalanine or l-proline gave decalins **32a** and **32b** in 75% yield (**32a**/**32b** = 2.1:1) or 59% yield (**32a**/**32b** = 3.8:1), respectively.^45^ Unfortunately, attempts to introduce α,β-unsaturation using DDQ, Saegusa conditions, or α-selenylation/elimination protocols all proved unsuccessful (Scheme 9B).

**Scheme 8:**
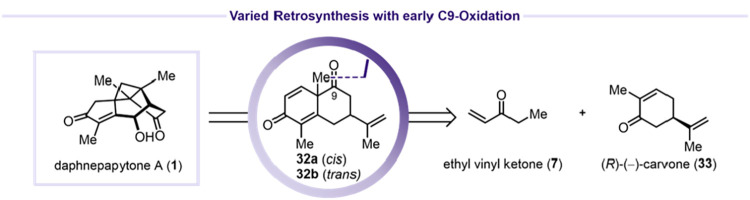
Synthesis Variation Using Early C9 Oxidation

**Scheme 9:**
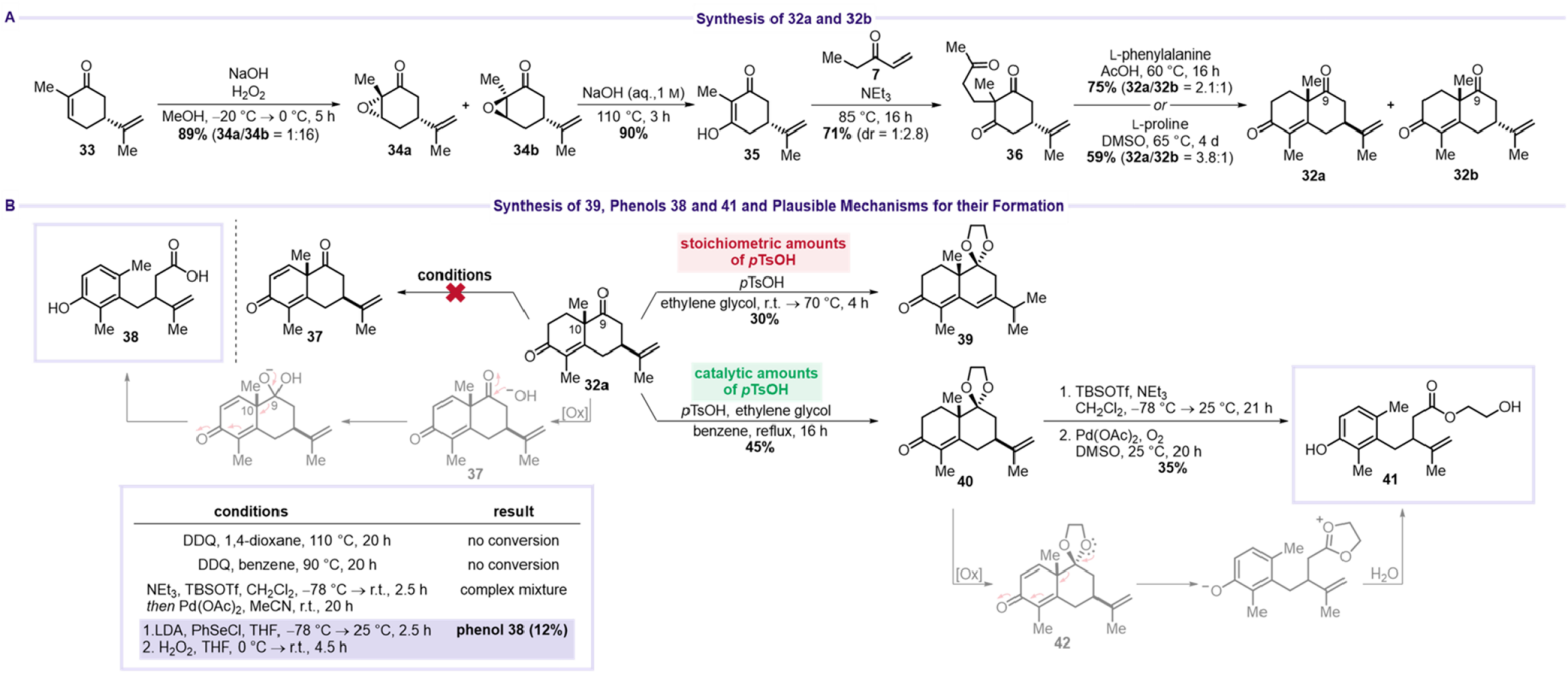
Fragmentation of **32a** and **40** to Their Corresponding Phenol Derivatives **38** and **41** ^a^

a (A): Synthesis of **32a** and **32b**, (B): Synthesis of **39**, Phenols **38** and **41**, and plausible mechanism for their formation. DMSO = Dimethyl sulfoxide, *p*TsOH = 4-methylbenzene-1-sulfonic acid, DDQ = 2,3-dichloro-5,6-dicyano-1,4-benzoquinone, TBSOTf = *tert*-butyldimethylsilyl trifluoromethanesulfonate, LDA = lithium diisopropylamide.

Notably, the α-selenylation/elimination sequence afforded phenol **38** in 12% yield. A plausible mechanism for the formation of **38** involves initial generation of dienone **37**, which undergoes nucleophilic attack by *in situ* generated hydroxide at C9, as described by Waring and Zaidi.^46^ Formation of a deprotonated hydrate at C9 facilitates C9–C10 bond cleavage with concomitant generation of a carboxylic acid, driven by aromatization of the electron-withdrawing dienone.

To circumvent this degradation pathway, the C9 ketone was protected as an acetal using *p*TsOH and ethylene glycol. Stoichiometric amounts of *p*TsOH led to isomerization, providing acetal **39** in 30% yield, whereas catalytic quantities of *p*TsOH afforded the desired **40** in 45% yield. Attempted DDQ oxidation of **40** resulted in a complex product mixture (not including any isolatable byproduct). Under Saegusa conditions, another phenol derivative, **41**, was isolated in 35% yield. The formation of **41** likely proceeds through a similar degradation pathway, wherein the electron-withdrawing acetal group promotes C9–C10 bond cleavage and aromatization. Subsequent hydrolysis of the resulting oxonium ion opens the cyclic acetal to furnish ester **41**.

## Conclusion

In conclusion, we have explored different synthetic routes toward daphnepapytone A (**1**) using α-santonin rearrangement as a key transformation. Through these investigations, we have successfully synthesized diverse guaiane frameworks, including daphbolide A (**3**) and B (**11**). Nevertheless, attempts at crucial C9 oxidation proved unsuccessful across various substrates using multiple oxidation protocols, representing a significant synthetic challenge that ultimately prevented completion of the total synthesis. Further, we discovered unexpected fragmentations, in which C6- or C9-functionalized dienone-bearing decalins rearranged to phenol derivatives. Our studies highlight the power and limitations of photochemical rearrangement strategies in terpenoid synthesis and the necessity of late-stage C9 oxidation protocols for guaianes.

## Supplementary Material



## Data Availability

The data underlying this study are available in the published article and its Supporting Information.

## Notes

Experimental details; characterization data; NMR spectra; HRMS analysis; and X-ray crystallographic data (PDF)

## Abbreviations

UV: ultraviolet
brsm: based on recovered starting material
