# Supplementary material for: Studies toward the Caged Sesquiterpenoid Daphnepapytone A
Source: J Org Chem. 2026 May 15;91(21):7164–72. doi: 10.1021/acs.joc.6c00079 (PMC13227466; doi:10.1021/acs.joc.6c00079)
Supplement: Supplementary file 1 [file jo6c00079_si_001.pdf]

Supporting Information

Studies toward the Caged Sesquiterpenoid Daphnepapytone A

Kamar Shakeri, Letizia Lanfredi, Christian Zachau, Luisa Eichner, Iulia Bîlici, Esra Dural, Jan-H. Dickoff, Manuela Weber, and Mathias Christmann*

Department of Biology, Chemistry, Pharmacy, Institute of Chemistry and Biochemistry, Freie Universität Berlin, Takustr. 3, 14195 Berlin, Germany.

*E-Mail: mathias.christmann@fu-berlin.de

1. General Information S1

1.1 Materials and Methods S1

1.2 Analysis S1

2. Screening Tables S3

2.1 α,β-Unsaturation of 5a and 5b S3

2.2 TBS-Protections of Alcohol 13 .... S3

2.3 Attempts on Allylic Oxidation of Daphbolide A (3) and Synthesis of Daphbolide B (11) S4

2.4 Attempts on Allylic Oxidation of 21 .... S5

2.5 Attempts on Allylic Oxidation of 26 and Synthesis of 29 .... S5

2.6 Attempts on Epoxide Opening of 30 .... S6

3. Synthetic Procedures S6

3.1 (4a*R*,7*R*)-1,4a-Dimethyl-7-(prop-1-en-2-yl)-4,4a,5,6,7,8-hexahydronaphthalen-2(3*H*)-one (10-*epi*-α-cyperone) (5a) and (4a*S*,7*R*)-1,4a-dimethyl-7-(prop-1-en-2-yl)-4,4a,5,6,7,8-hexahydronaphthalen-2(3*H*)-one (α-cyperone) (5b) S6

3.2 (4a*R*,7*R*)-1,4a-Dimethyl-7-(prop-1-en-2-yl)-5,6,7,8-tetrahydronaphthalen-2(4a*H*)-one (4a) and (4a*S*,7*R*)-1,4a-dimethyl-7-(prop-1-en-2-yl)-5,6,7,8-tetrahydronaphthalen-2(4a*H*)-one (4b) S7

3.2.1 Separate DDQ Oxidations of 5a and 5b S10

3.3 (3a*S*,4*S*,7*R*)-1,4-Dimethyl-2-oxo-7-(prop-1-en-2-yl)-2,3,3a,4,5,6,7,8-octahydroazulen-4-yl acetate (8a) and (3a*S*,4*R*,7*R*)-1,4-Dimethyl-2-oxo-7-(prop-1-en-2-yl)-2,3,3a,4,5,6,7,8-octahydroazulen-4-yl acetate (8b) S11

3.4 (*R*)-3,8-Dimethyl-5-(prop-1-en-2-yl)-4,5,6,7-tetrahydroazulen-2(1*H*)-one (Daphbolide A) (3) S12

3.5 (5*R*,8*S*,8a*S*)-8-Hydroxy-3,8-dimethyl-5-(prop-1-en-2-yl)-4,5,6,7,8,8a-hexahydroazulen-2(*1H*)-one (9) S14

3.6 3,8-Dimethyl-5-(propan-2-ylidene)-4,5,6,7-tetrahydroazulen-2(1*H*)-one (10) S15

3.7 (*S*)-5-Hydroxy-3,8-dimethyl-5-(prop-1-en-2-yl)-4,5,6,7-tetrahydroazulen-2(1*H*)-one (Daphbolide B) (11) S16

3.8 (4a*R*,7*S*,8*S*)-8-Hydroxy-1,4a-dimethyl-7-(prop-1-en-2-yl)-4,4a,5,6,7,8-hexahydronaphthalen-2(3*H*)-one (12) S16

3.9 (4a*R*,7*S*,8*S*)-8-Hydroxy-1,4a-dimethyl-7-(prop-1-en-2-yl)-5,6,7,8-tetrahydronaphthalen-2(4a*H*)-one (13) S17

3.10 (4*S*,5*S*,8*R*)-3,8-Dimethyl-5-(prop-1-en-2-yl)-4,5,6,7,8,8a-hexahydro-4,8-epoxyazulen-2(1*H*)-one (14) S19

3.11 (4a*R*,7*S*,8*S*)-8-((*tert*-Butyldimethylsilyl)oxy)-1,4a-dimethyl-7-(prop-1-en-2-yl)-5,6,7,8-tetrahydronaphthalen-2(4a*H*)-one (15) S20

3.12 (3a*R*,4*S*,7*S*,8*S*)-8-((*tert*-Butyldimethylsilyl)oxy)-1,4-dimethyl-2-oxo-7-(prop-1-en-2-yl)-2,3,3a,4,5,6,7,8-octahydroazulen-4-yl acetate (16) S22

3.13 (3a*R*,4*S*,7*S*,8*S*)-8-((*tert*-Butyldimethylsilyl)oxy)-1,4-dimethyl-2-oxo-7-(prop-1-en-2-yl)-2,3,3a,4,5,6,7,8-octahydroazulen-4-yl acetate (16) and 2-(4-Hydroxy-2,3-dimethylphenethyl)-3-methylbut-2-enal (17) S23

3.14 (4*S*,5*S*,8*S*,8a*S*)-4-((*tert*-Butyldimethylsilyl)oxy)-8-hydroxy-3,8-dimethyl-5-(prop-1-en-2-yl)-4,5,6,7,8,8a-hexahydroazulen-2(1*H*)-one (18) S24

3.15 (4*S*,5*S*)-4-((*tert*-Butyldimethylsilyl)oxy)-3,8-dimethyl-5-(prop-1-en-2-yl)-4,5,6,7-tetrahydroazulen-2(1*H*)-one (21) S25

3.16 (3S,3aS,6R,6aR,9bS)-6-hydroxy-3,6,9-trimethyl-3a,5,6,6a,7,9b-hexahydroazuleno[4,5-b]furan-2,8(3H,4H)-dione (24) S26

3.17 (3*S*,3a*S*,6*R*,6a*R*,9b*S*)-3,6,9-Trimethyl-2,8-dioxo-2,3,3a,4,5,6,6a,7,8,9b-decahydroazuleno[4,5-b]furan-6-yl acetate (25) S26

3.18 (3*S*,3a*S*,9b*S*)-3,6,9-Trimethyl-3a,5,7,9b-tetrahydroazuleno[4,5-b]furan-2,8(3*H*,4*H*)-dione (26) S27

3.19 5-Ethyl-3,8-dimethyl-6,7-dihydroazulen-2(1*H*)-one (27) S29

3.20 4-Methyl-5-((2*S*,3*S*,4*S*)-4-methyl-5-oxo-3-(3-oxobutyl)tetrahydrofuran-2-yl)cyclopent-4-ene-1,3-dione (29) S29

3.21 (3a*R*,4a*S*,6a*S*,7*S*,9a*S*)-1,4a,7-Trimethyl-5,6,6a,9a-tetrahydro-3*H*-oxireno[2',3':8,8a]azuleno[4,5-b]furan-2,8(4a*H*,7*H*)-dione (30) S30

3.22 (1S,4*R*,6*S*)-1-Methyl-4-(prop-1-en-2-yl)-7-oxabicyclo[4.1.0]heptan-2-one (34a) (1*R*,4*R*,6*R*)-1-methyl-4-(prop-1-en-2-yl)-7-oxabicyclo[4.1.0]heptan-2-one (34b) S31

3.23 (*R*)-3-Hydroxy-2-methyl-5-(prop-1-en-2-yl)cyclohex-2-en-1-one (35) S32

3.24 2-Methyl-2-(3-oxopentyl)-5-(prop-1-en-2-yl)cyclohexane-1,3-dione (36) S32

3.25 5,8a-Dimethyl-3-(prop-1-en-2-yl)-3,4,8,8a-tetrahydronaphthalene-1,6(2*H*,7*H*)-diones (32a and 32b) S33

3.26 3-(3-Hydroxy-2,6-dimethylbenzyl)-4-methylpent-4-enoic acid (38) S34

3.27 3-Isopropyl-5,8a-dimethyl-8,8a-dihydro-2*H*-spiro[naphthalene-1,2'-[1,3]dioxolan]-6(7*H*)-one (39) S36

3.28 (3S,8aS)-5,8a-dimethyl-3-(prop-1-en-2-yl)-3,4,8,8a-tetrahydro-2H-spiro[naphthalene-1,2'-[1,3]dioxolan]-6(7H)-one (40) S36

3.29 2-Hydroxyethyl 3-(3-hydroxy-2,6-dimethylbenzyl)-4-methylpent-4-enoate (41)...S-37

3.30 Further Compounds Prepared during those Studies S38

- **3.30.1 (1*S*,2*S*,4a*R*)-4a,8-Dimethyl-7-oxo-2-(prop-1-en-2-yl)-1,2,3,4,4a,5,6,7-octahydronaphthalen-1-yl 4-nitrobenzoate (S1) S38**
- **3.30.2 4a,8-Dimethyl-7-oxo-2-(prop-1-en-2-yl)-1,2,3,4,4a,5,6,7-octahydronaphthalen-1-yl 3,5-dinitrobenzoate (S2) S39**
- **3.30.3 (1*S*,2*S*,4a*R*)-4a,8-Dimethyl-7-oxo-2-(prop-1-en-2-yl)-1,2,3,4,4a,7-hexahydronaphthalen-1-yl 3,5-dinitrobenzoate (S3) S40**

3.30.4 (4*S*,5*S*,8*S*,8a*S*)-8-Acetoxy-3,8-dimethyl-2-oxo-5-(prop-1-en-2-yl)-1,2,4,5,6,7,8,8a-octahydroazulen-4-yl 3,5-dinitrobenzoate (S4) S41

3.30.5 (3*R*,5*R*)-3-Hydroxy-2-methyl-5-(prop-1-en-2-yl)cyclohexan-1-one (S5) S41

3.30.6 (2*S*,3*R*,5*R*)-2-Methyl-5-(prop-1-en-2-yl)-3-((tetrahydro-2H-pyran-2-yl)oxy)cyclohexan-1-one (S6) S43

3.30.7 (3S,3aS,6aS,9bS)-3,9-dimethyl-6-methylene-3a,5,6,6a,7,9b-hexahydroazuleno[4,5-b]furan-2,8(3H,4H)-dione (S7) S44

4. X-ray data S45

4.1 X-ray data of 9 ..... S45

4.2 X-ray data of 13 ..... S46

4.3 X-ray data of 17 ..... S47

5. NMR and Mass Spectrometry Data S48

6. References S112

1. General Information

1.1 Materials and Methods

Reactions involving air or moisture sensitive substances were carried out under an argon atmosphere using standard Schlenk techniques. Ambient or room temperature (r.t.) refers to 18–23 °C. Heating of reactions was performed with an oil bath unless otherwise noted. Brine refers to a saturated aqueous NaCl solution.

Unless otherwise stated, all starting materials and reagents were obtained from commercial suppliers and used without further purification. Anhydrous benzene, dichloromethane, and tetrahydrofuran were dispensed from an MBraun SPS-800 solvent purification system (MBraun) using HPLC grade solvents purchased from Fisher Scientific and Roth. Solvents for extraction, crystallization, and flash column chromatography were purchased in technical grade and distilled under reduced pressure prior to use.

Column chromatography was performed on silica gel 60 M (0.040–0.063 mm, 230–400 mesh, Macherey-Nagel). Medium-pressure liquid chromatography (MPLC) was carried out on a Teledyne ISCO CombiFlash Rf 200 system using prepacked silica columns and cartridges (Teledyne ISCO). UV detection was monitored at 254 and 280 nm. Cyclohexane (99.5+%) and EtOAc (HPLC grade) were used as eluents.

1.2 Analysis

Reaction monitoring: Reactions were monitored by thin-layer chromatography (TLC). TLC analysis was performed on silica gel coated aluminum plates (ALUGRAM Xtra SIL G/UV254, Macherey-Nagel). Spots were visualized under UV light at 254 nm and by staining with Ce(SO4)2- and anisaldehyde-based reagents. Unless otherwise noted, the term "complex mixture" indicates an indistinguishable and inseparable combination of multiple products as observed by TLC.

NMR spectroscopy: ^{1}H, ^{13}C, and ^{19}F NMR spectra were recorded on JEOL (ECX 400, ECP 500), Varian (Inova 600), and Bruker (AVANCE III 500, AVANCE III 700) spectrometers in the indicated deuterated solvents. Chemical shifts (δ) are reported in parts per million (ppm) relative to the residual nondeuterated solvent signal ($CDCl_3$: δ_H 7.26, δ_C 77.2; CD_2Cl_2: δ_H 5.32, δ_C 53.8; DMSO-*d6*: δ_H 2.50, δ_C 39.5). Integrals are consistent with the stated assignments;

coupling constants (*J*) are reported in Hz. Multiplicities are indicated as follows: s (singlet), d (doublet), t (triplet), q (quartet), p (quintet), br (broad), and combinations thereof. Where no clear multiplicity could be assigned, signals are reported as m (multiplet). ^{13}C NMR spectra are ^{1}H broadband decoupled.

Structural assignments were made with additional information from gCOSY, gHMQC, and gHMBC experiments.

High-resolution mass spectrometry: High-resolution mass spectra (HRMS) were recorded on an Agilent 6210 ESI-TOF mass spectrometer (10 µL min^{-1}, 1.0 bar, 4 kV).

Infrared spectroscopy: Infrared (IR) spectra were recorded on a Jasco FT/IR-4100 Type A spectrometer equipped with a TGS detector. Wavenumbers ($\tilde{\nu}$) are reported in cm^{-1}.

X-ray: X-ray diffraction data was collected on a BRUKER D8 Venture CMOS area detector (Photon 100) diffractometer with Cu Kα radiation. Single crystals were coated with perfluoroether oil and mounted on a 0.2 mm Micromount. The structures were solved with the ShelXT1 structure solution program using intrinsic phasing and refined with the ShelXL2 refinement package using least squares on weighted F2 values for all reflections using OLEX2.

Light source: A self-assembled and partially 3d-printed setup was used for all photoreactions unless otherwise stated. As a light source, CHANZON LED chips (365 nm, 30 W) or KESSIL lamps (370 nm (44 W), 456 nm, blue tuna) were used and cooled from the bottom using a water cooler (40 × 40 mm, made from aluminum from the company KALOLARY). The distance between the light source and the vessel was around 1 – 2 cm.

2. Screening Tables

2.1 α,β-Unsaturation of 5a and 5b

Table S1. α,β-Unsaturation of **5a** and **5b**.

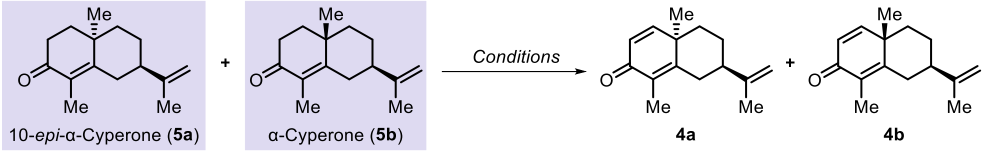

Entry	Conditions	Result
**1**	1. TMSOTf, NEt_3, CH_2Cl_2, 0 °C, 2.5 h<br>2. NBS, CH_2Cl_2, 0 °C, 3 h<br>3. DBU, THF/CH_2Cl_2 (3:8), 50 °C, 18 h	complex mixture
**2**	DDQ, 1,4-dioxane, 110 °C, 24 h	20% (brsm), dr = 3:1 (**4a**:**4b**)
**3**	1. LDA, PhSeCl, THF, –78 °C → r.t., 2.5 h<br>2. H_2O_2, THF, 0 °C → r.t., 3 h	52% over two steps, dr = 4:1 (**4a**:**4b**)
**4**	1. LiHMDS, PhSeBr, THF, –78 °C, 30 min<br>2. H_2O_2, THF, 0 °C, 1.5 h	69% over two steps, dr = 4:1 (**4a**:**4b**)

a) see chapter 3.2.1 for further investigation of the DDQ-oxidation

2.2 TBS-Protections of Alcohol 13

Table S2. TBS-protection of **13**.

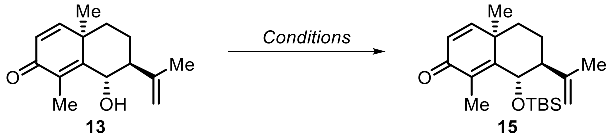

Entry	Conditions	Result[a)]
**1**	2,6-lutidine, TBSOTf, CH_2Cl_2, r.t. → 40 °C, 25 h	31% (52% brsm)
**2**	imidazole, TBSCl, DMF, 40 °C, 20 h<br>*then* DMF, 45 °C, 24 h	37% (65% brsm)
**3**	imidazole, TBSOTf, DMF, 40 °C, 19 h	73%

a) isolated yield

2.3 Attempts on Allylic Oxidation of Daphbolide A (3) and Synthesis of Daphbolide B (11)

Table S3. Attempts on oxidation of daphbolide A (**3**) to oleodaphnone (**2**).

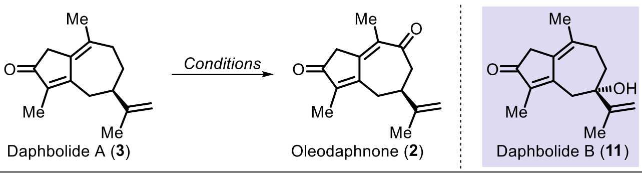

Entry	Conditions	Result[a)]
**1**	DBU, O_2, MeCN, reflux, 24 h	no conversion
**2**	DABCO, O_2, MeCN, reflux, 24 h	no conversion
**3**	KOH, MeOH, O_2, r.t., 22 h	no conversion
**4**	KO*t*Bu, O_2, MeCN, r.t., 24 h	complex mixture
**5**	PDC, *t*BuOOH, CH_2Cl_2, 0 °C, 24 h	complex mixture
**6**	PDC, *t*BuOOH, benzene, 3 Å MS, r.t., 24 h	complex mixture
**7**	$Pd(OH)_2$/C, *t*BuOOH, K_2CO_3, CH_2Cl_2, 0 °C → r.t., 44 h	complex mixture
**8**	$Cr(CO)_6$, *t*BuOOH, MeCN, 85 °C, 16 h	complex mixture
**9**	NHPI, $Mn(OAc)_2$, $Co(OAc)_2$, O_2, acetone, r.t. → 50 °C, 16 h	complex mixture
**10**	NHPI, $Co(acac)_2$, O_2, MeCN, r.t., 16 h, *then* 50 °C, 16 h	no conversion
**11**	CrO_3, pyridine, CH_2Cl_2, 0 °C → r.t., 1.5 h	complex mixture
**12**	CrO_3, AcOH, CH_2Cl_2, r.t., 1 h *or* 16 h	complex mixture
**13**	CrO_3, *t*BuOOH, CH_2Cl_2, r.t., 16 h	complex mixture
**14**	$Mn(OAc)_3 \cdot 2\ H_2O$, *t*BuOOH, MS 3 Å, EtOAc, r.t., 16 h	complex mixture
**15**	TBADT, 370 nm, MeCN, O_2, r.t., 16 h	complex mixture
**16**	Rose bengal, *n*Bu_4NBr, O_2, 456 nm, MeCN, r.t., 2.5 h	complex mixture
**17**	Ethyl-2-mercapto propanoate, 4-CzIPN, 456 nm, MeCN, r.t., 16 h	complex mixture
**18**	SeO_2, *t*BuOOH, CH_2Cl_2, 0 °C, 4 h	Daphbolide B (**11**) (19%)

a) after checking crude ^{1}H NMR; "complex mixture" indicates an indistinguishable combination of products not including oleodaphnone (**2**)

2.4 Attempts on Allylic Oxidation of 21

Table S4. Attempts on allylic oxidation of **21**.

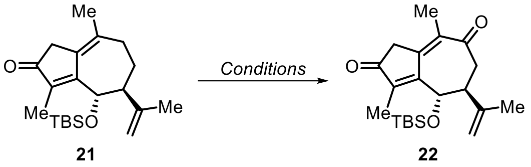

Entry	Conditions	Result
**1**	Rose bengal, 456 nm, imidazole, MeCN, r.t., 1 h	complex mixture
**2**	CuI, *t*BuOOH, MeCN, reflux, 5 h	complex mixture
**3**	$RuCl_3$, *t*BuOOH, *c*Hex, r.t., 16 h	no conversion
**4**	Cu-Al Ox, *t*BuOOH, L-proline, MeCN, 82 °C, 16 h	no conversion
**5**	$Cr(CO)_6$, *t*BuOOH, MeCN, 85 °C, 16 h	complex mixture
**6**	CrO_3, pyridine, CH_2Cl_2, r.t., 16 h	complex mixture
**7**	$Mn(OAc)_3$, MS 3 Å, *t*BuOOH, EtOAc, r.t. → 40 °C, 16 h	complex mixture

2.5 Attempts on Allylic Oxidation of 26 and Synthesis of 29

Table S5. Attempts on allylic oxidation of **26**.

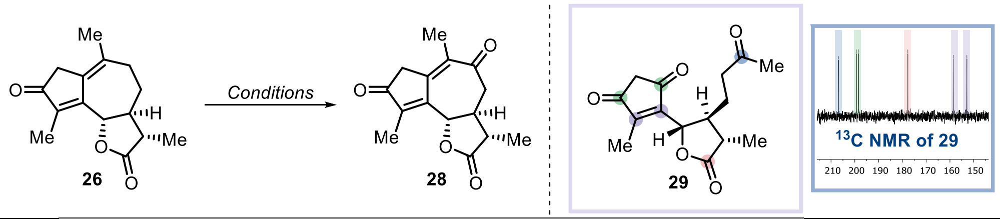

Entry	Conditions	Result
**1**	CrO_3, AcOH, r.t., 3 h	triketone **29** (10%)
**2**	$Mn(OAc)_3 \cdot 2\ H_2O$, TBHP, EtOAc, r.t., 130 min	triketone **29** (5%)
**3**	CrO_3, pyridine, CH_2Cl_2, 0 °C, 4 h	triketone **29** (29%)
**4**	TBADT, O_2, 370 nm, r.t., 3 h	complex mixture
**5**	Rose bengal, *n*Bu_4NBr, MeCN, 456 nm, r.t.	no conversion
**6**	SeO_2, 1,4-dioxane, 100 °C, 70 min	complex mixture
**7**	SeO_2, 1,4-dioxane, 40 °C, 16 h	complex mixture
**8**	SeO_2, 1,4-dioxane, 40 °C, 16 h	complex mixture
**9**	SeO_2, TBHP (70% in H_2O), CH_2Cl_2, 0 °C → r.t., 20 h	no conversion
**10**	SeO_2, TBHP (5.5 M in decane), CH_2Cl_2, 45 °C	no conversion

2.6 Attempts on Epoxide Opening of 30

Table S6. Attempts on epoxide opening of **30**.

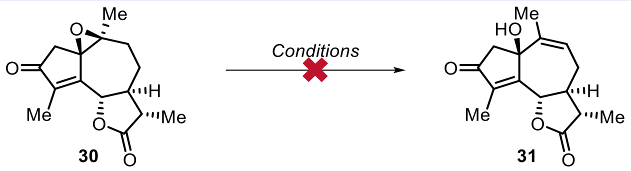

Entry	Conditions	Result
1	$ZnBr_2$, CH_2Cl_2, r.t., 20 h	complex mixture
2	$Yb(OTf)_3$, *t*BuOH, toluene, 100 °C, 16 h	complex mixture
3	$Sc(OTf)_3$, *t*BuOH, toluene, 100 °C, 16 h	complex mixture
4	HCl (1 w% in MeOH), $CHCl_3$, r.t., 3 min	complex mixture
5	*p*TsOH, THF/H_2O (1:1), r.t., 4 d	no conversion
6	HCl (aq., 1 M), CH_2Cl_2, 20 h	complex mixture
7	DBU, MeCN, r.t., 2 h	complex mixture

3. Synthetic Procedures

3.1 (4a*R*,7*R*)-1,4a-Dimethyl-7-(prop-1-en-2-yl)-4,4a,5,6,7,8-hexahydronaphthalen-2(3*H*)-one (10-*epi*-α-cyperone) (5a) and (4a*S*,7*R*)-1,4a-dimethyl-7-(prop-1-en-2-yl)-4,4a,5,6,7,8-hexahydronaphthalen-2(3*H*)-one (α-cyperone) (5b)

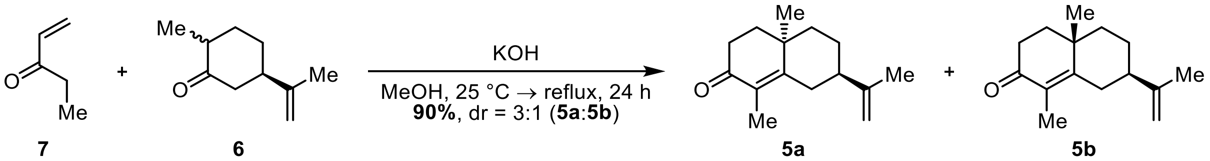

Enones **5a** and **5b** were synthesized according to a modified literature procedure:[1] A 250 mL Schlenk flask equipped with a Teflon-coated magnetic stirring bar was charged with KOH (10% in MeOH, 115 mL), and (+)-dihydrocarvone (**6**, 16.1 mL, 98.5 mmol, 1.00 equiv) was added. The solution was cooled to 0 °C and stirred for 15 min. Enone **7** (11.8 mL, 118 mmol, 1.20 equiv) was added dropwise over 30 min at 0 °C, and the reaction mixture was stirred for an additional 30 min. The reaction mixture was warmed to room temperature and stirred for 18 h, then heated at reflux in MeOH for 4.5 h, cooled to room temperature, and concentrated to a volume of 50 mL. The mixture was diluted with EtOAc (120 mL) and quenched with saturated aqueous NH_4Cl (100 mL). The resulting white salt was filtered off and the aqueous layer was extracted with EtOAc (3 × 100 mL). The combined organic layers were dried over $MgSO_4$, filtered, and concentrated under reduced pressure. The orange residue was purified by flash

column chromatography (SiO_2, pentane/Et_2O 5:1) to afford a mixture of enones **5a** and **5b** (19.2 g, 87.9 mmol, 90% yield, dr = 3:1 (**5a**:**5b**)) as a light-yellow oil.

NMR data of 10-*epi*-α-cyperone (5a)

^{1}H NMR (400 MHz, $CDCl_3$): δ = 4.80 (s, 1H), 4.61 (s, 1H), 2.88 (dq, J = 16.0, 2.5 Hz, 1H), 2.61 – 2.51 (m, 2H), 2.44 – 2.31 (m, 2H), 1.96 – 1.83 (m, 2H), 1.81 (br s, 3H), 1.71 (s, 3H), 1.70 – 1.58 (m, 2H), 1.50 (tt, J = 13.3, 3.3 Hz, 1H), 1.33 (dq, J = 13.6, 3.5 Hz, 1H), 1.23 (d, J = 2.7 Hz, 3H) ppm.

The spectroscopic data are consistent with those reported in the literature.[2]

NMR data of α-cyperone (5b)

^{1}H NMR (400 MHz, $CDCl_3$): δ = 4.78 (s, 2H), 2.78 – 2.69 (m, 1H), 2.52 (ddd, J = 16.9, 13.5, 6.1 Hz, 1H), 2.40 (dt, J = 16.9, 4.5 Hz, 1H), 2.09 – 2.00 (m, 2H), 1.84 – 1.59 (m, 5H), 1.78 (s, 6H), 1.47 – 1.39 (m, 1H), 1.22 (s, 3H) ppm.

The spectroscopic data are consistent with those reported in the literature.[3,4]

3.2 (4a*R*,7*R*)-1,4a-Dimethyl-7-(prop-1-en-2-yl)-5,6,7,8-tetrahydronaphthalen-2(4a*H*)-one (4a) and (4a*S*,7*R*)-1,4a-dimethyl-7-(prop-1-en-2-yl)-5,6,7,8-tetrahydronaphthalen-2(4a*H*)-one (4b)

Method A

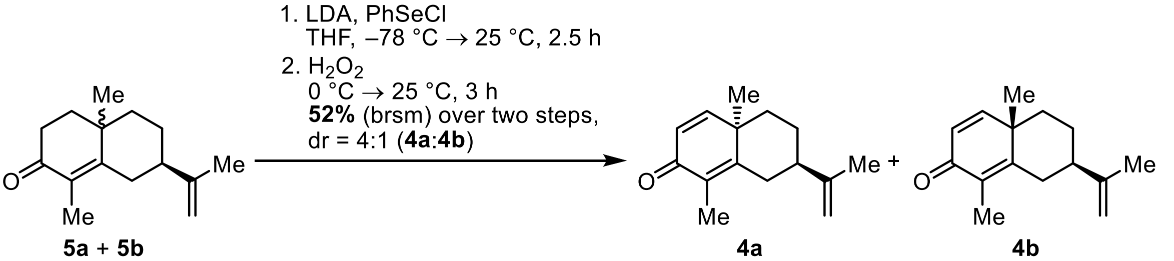

Dienones **4a** and **4b** were synthesized according to a modified literature procedure:[5] A dry 2 L Schlenk flask equipped with a Teflon-coated magnetic stirring bar was charged with a solution of enones **5a** and **5b** (3.30 g, 15.1 mmol, 1.00 equiv, dr = 3:1 (**5a**:**5b**)) in dry THF (363 mL) under argon and cooled to −78 °C. LDA (0.171 M, 124 mL, 21.2 mmol, 1.40 equiv) [prepared from *n*BuLi (2.50 M, 8.46 mL, 21.2 mmol, 1.40 equiv) and diisopropylamine (2.99 mL, 21.2 mmol, 1.40 equiv) in dry THF (124 mL) at −78 °C] was added dropwise to the stirring solution, and the mixture was stirred for 30 min. A solution of phenylselenyl chloride (3.18 g,

16.6 mmol, 1.10 equiv) in dry THF (124 mL) was added dropwise at −78 °C. The reaction mixture was stirred for 2 h while warming to room temperature. The mixture was poured into 1 M aqueous HCl (600 mL), and the aqueous layer was extracted with Et_2O (3 × 200 mL). The combined organic layers were washed with H_2O (300 mL) and brine (2 × 300 mL), dried over $MgSO_4$, and concentrated under reduced pressure to give a green oil. The crude product was purified by flash column chromatography (SiO_2, *c*Hex to *c*Hex/EtOAc 99:1) to give phenyl selenide (4.32 g, still impure) as a yellow oil and recovered starting material **5a** and **5b** (0.255 g, 1.17 mmol). The phenyl selenide was used in the subsequent elimination step without further purification.

A 1 L round-bottom flask equipped with a Teflon-coated magnetic stirring bar was charged with a solution of the selenide (4.32 g, 11.6 mmol, 1.00 equiv) in THF (320 mL) and cooled to 0 °C. Hydrogen peroxide (15% in H_2O, 23.6 mL, 116 mmol, 10.0 equiv) was added dropwise over 10 min to the stirring solution. After 1 h, the mixture was warmed to 10 °C, and after an additional 2 h, the mixture was warmed to room temperature. The solution was poured into saturated aqueous $NaHCO_3$ (400 mL), and the aqueous layer was extracted with Et_2O (3 × 200 mL). The combined organic layers were dried over $MgSO_4$, filtered, and concentrated under reduced pressure. The resulting yellow oil was dissolved in Et_2O (100 mL) and washed with saturated aqueous Na_2CO_3 (3 × 50 mL) and H_2O (2 × 50 mL). The aqueous layers were re-extracted with CH_2Cl_2 (3 × 100 mL), and the combined organic phases were dried over $MgSO_4$, filtered, and concentrated under reduced pressure. Purification by flash column chromatography (SiO_2, pentane/Et_2O 9:1) afforded dienones **4a** and **4b** (1.70 g, 7.85 mmol, 52% over two steps, dr = 4:1 (**4a**:**4b**)) as a light-yellow oil.

NMR data of the major isomer 8,9-Dehydro-10-*epi*-α-cyperone (4a)
^{1}H NMR (400 MHz, $CDCl_3$): δ = 6.72 (d, J = 9.9 Hz, 1H), 6.21 (d, J = 9.7 Hz, 1H), 4.76 (d, J = 1.4 Hz, 1H), 4.60 (br s, 1H), 3.05 (dt, J = 14.8, 2.1 Hz, 1H), 2.65 (br s, 1H), 2.50 (ddd, J = 14.8, 6.5, 1.4 Hz, 1H), 2.05 – 1.98 (m, 1H), 1.95 (d, J = 1.4 Hz, 3H), 1.87 – 1.81 (m, 1H), 1.70 (d, J = 0.7 Hz, 3H), 1.61 – 1.52 (m, 2H), 1.26 (s, 3H) ppm.

The spectroscopic data are consistent with those reported in the literature.[6]

Method B

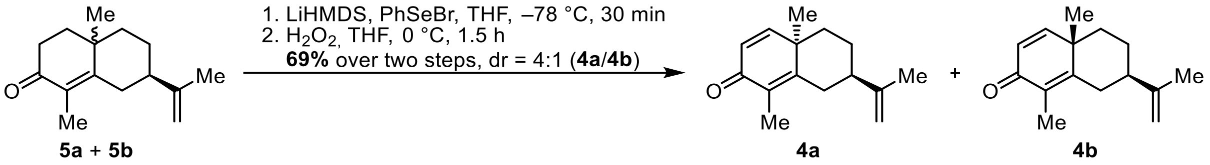

Enones **5a** and **5b** (500 mg, 2.29 mmol, 1.00 equiv) were dissolved in THF (5 mL) and cooled to −78 °C. LiHMDS (1.0 M in THF, 2.98 mL, 2.98 mmol, 1.30 equiv) was added dropwise, and the solution was stirred for 10 min. PhSeBr (702 mg, 2.98 mmol, 1.30 equiv) in THF (1.25 mL) was then added dropwise, and the reaction mixture was stirred at −78 °C for 30 min. Saturated aqueous NH_4Cl (5 mL) was added to quench the reaction. The aqueous phase was separated and extracted with Et_2O (3 × 10 mL), and the combined organic phases were dried over $MgSO_4$, filtered, and concentrated under reduced pressure. The crude product was used directly in the next step without further purification.

The crude product was dissolved in THF (20 mL) and cooled to 0 °C. Hydrogen peroxide (35% in H_2O, 1.35 mL, 2.75 mmol, 1.20 equiv) was added dropwise, and the reaction mixture was stirred for 1.5 h before being poured into saturated aqueous $NaHCO_3$ (10 mL). The aqueous phase was extracted with CH_2Cl_2 (3 × 20 mL), and the combined organic phases were dried over $MgSO_4$, filtered, and concentrated under reduced pressure. Purification by flash column chromatography (SiO_2, pentane/Et_2O 9:1) afforded dienones **4a** and **4b** (341 mg, 1.58 mmol, 69% over two steps, dr = 4:1 (**4a**:**4b**)) as a light-yellow oil.

Method C

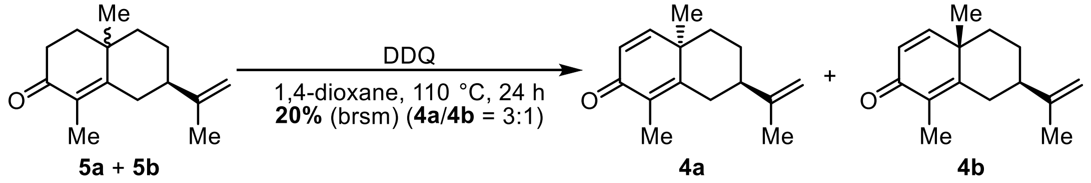

Enones **5a** and **5b** (100 mg, 458 µmol, 1.00 equiv, dr = 3:1 (**5a**:**5b**)) were dissolved in dry 1,4-dioxane (6.0 mL), and DDQ (207 mg, 916 µmol, 2.00 equiv) was added. The resulting suspension was heated at reflux for 24 h. Upon completion of the reaction, the solvent was concentrated under reduced pressure to one-fourth of its original volume. The residue was adsorbed on basic alumina and washed with 30% EtOAc in cyclohexane until TLC analysis showed no traces of product. The solvent was removed under reduced pressure to afford dienones **4a** and **4b** as a yellow oil (19.8 mg, 91.6 µmol, 20% yield (brsm)).

3.2.1 Separate DDQ Oxidations of 5a and 5b

Interestingly, the recovered starting material consisted exclusively of the minor diastereomer α-cyperone (**5b**), whereas the product was obtained with the same diastereomeric ratio (dr = 3:1) as the starting material. This observation suggests that **5b** reacts with a higher effective yield, since its proportion in the starting material and in the product mixture remains constant despite being the only diastereomer recovered unreacted. To test this hypothesis, diastereomers **4a** and **4b** were separated by HPLC and subjected independently to oxidation with DDQ (Scheme S1). 10-*Epi*-α-cyperone (**5a**) afforded dienone **4a** in 18% yield, whereas α-cyperone (**5b**) gave dienone **4b** in 43% yield together with 13% of recovered **5b**, which supports this hypothesis. We attribute the lower yield for **4a** to steric hindrance of the isopropenyl group in the hydride abstraction step when C9–H is *cis* to this substituent, as in **5a**.

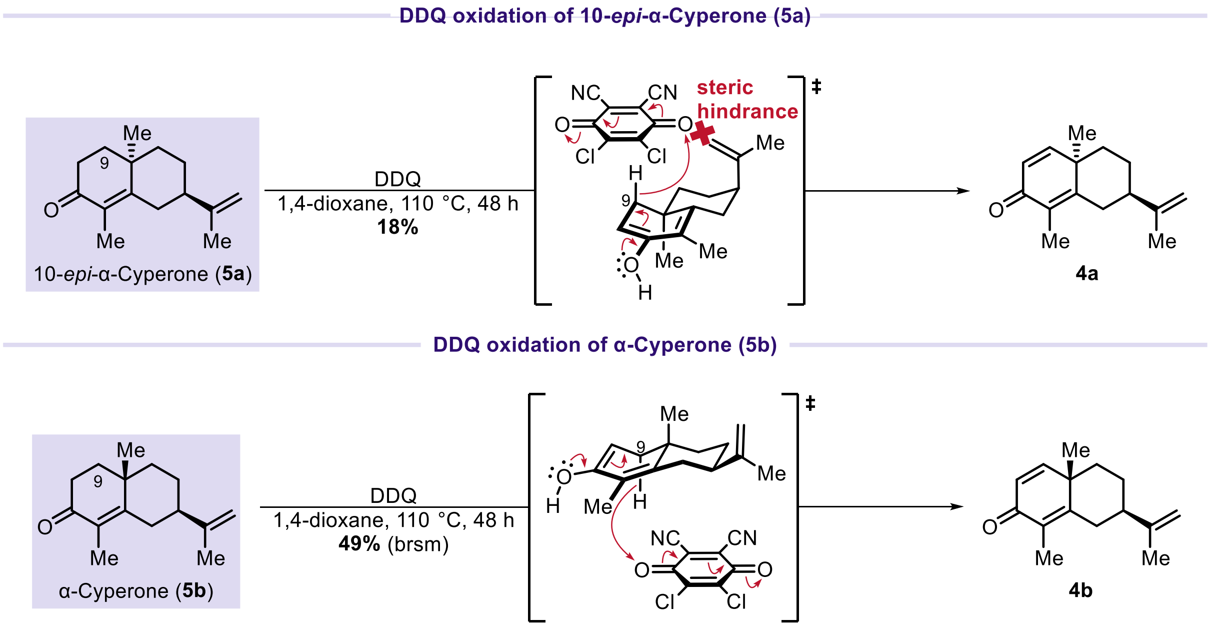

Scheme S1. Separate DDQ oxidations of **5a** and **5b** and rationale for the observed outcome.

3.3 (3a*S*,4*S*,7*R*)-1,4-Dimethyl-2-oxo-7-(prop-1-en-2-yl)-2,3,3a,4,5,6,7,8-octahydroazulen-4-yl acetate (8a) and (3a*S*,4*R*,7*R*)-1,4-Dimethyl-2-oxo-7-(prop-1-en-2-yl)-2,3,3a,4,5,6,7,8-octahydroazulen-4-yl acetate (8b)

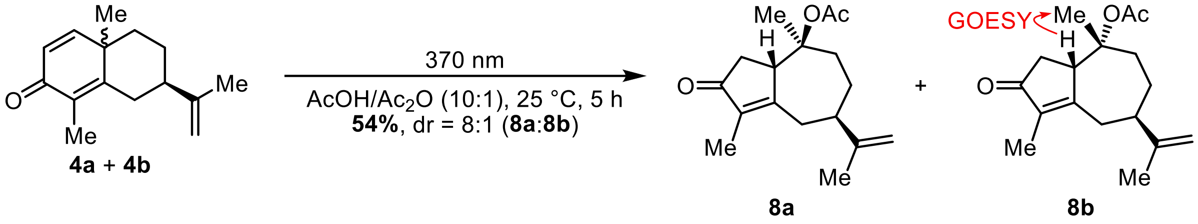

A 100 mL round-bottom flask equipped with a Teflon-coated magnetic stirring bar was charged with dienones **4a** and **4b** (661 mg, 3.06 mmol, 1.00 equiv, dr = 4:1 (**4a**:**4b**)) in a mixture of glacial AcOH (31.5 mL) and Ac_2O (3.15 mL) under argon. The solution was irradiated with a UV LED (370 nm, 44 W) for 5 h. The mixture was then concentrated under reduced pressure to afford the crude product as a yellow oil. Purification by flash column chromatography (SiO_2, *c*Hex to *c*Hex/EtOAc 9:1) gave **8a** and **8b** (458 mg, 1.66 mmol, 54% yield, dr = 8:1 (**8a**:**8b**)) as a light-yellow oil. Characterization data for the isolated major diastereomer **8a** are given below.

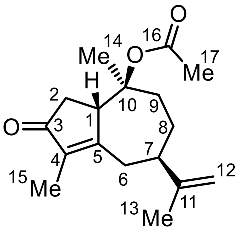

8a

^{1}H NMR (700 MHz, $CDCl_3$): δ = 4.78 (s, 1H, H_a-12), 4.66 (s, 1H, H_b-12), 3.55 – 3.53 (m, 1H, H-1), 2.69 – 2.63 (m, 2H, H-6), 2.48 (dd, J = 18.4, 6.6 Hz, 1H, H_a-2), 2.31 – 2.27 (m, 1H, H_a-9), 2.16 – 2.14 (m, 1H, H-7), 2.12 (dd, J = 18.4, 3.7 Hz, 1H, H_b-2), 2.02 (s, 3H, H-17), 1.77 – 1.73 (m, 1H, H_a-8), 1.75 (s, 3H, H-13), 1.72 – 1.70 (m, 1H, H_b-9), 1.68 (d, J = 2.8 Hz, 3H, H-15), 1.68 – 1.64 (m, 1H, H_b-8), 1.37 (s, 3H, H-14) ppm.

^{13}C{^{1}H} NMR (176 MHz, $CDCl_3$): δ = 207.3 (C-3), 173.1 (C-5), 170.2 (C-16), 148.5 (C-11), 138.1 (C-4), 110.5 (C-12), 85.3 (C-10), 51.1 (C-1), 45.2 (C-7), 38.3 (C-2), 34.2 (C-6), 34.1 (C-9), 28.5 (C-8), 22.6 (C-14), 22.5 (C-17), 20.7 (C-13), 7.9 (C-15) ppm.

HRMS (ESI, pos): *m/z* calculated for $C_{17}H_{24}O_3Na^+$ $[M+Na]^+$: 299.1618; found: 299.1625.

IR (ATR): $\tilde{\nu}$ = 2936 (w), 2867 (w), 1730 (s), 1699 (ss), 1639 (m), 1437 (w), 1368 (m), 1240 (s), 1177 (m), 1078 (m), 1015 (m), 940 (m), 893 (m), 805 (w) cm^{-1}.

$[\alpha]_D^{25}$ = –34.29° (c = 1.00, $CHCl_3$).

3.4 (*R*)-3,8-Dimethyl-5-(prop-1-en-2-yl)-4,5,6,7-tetrahydroazulen-2(1*H*)-one (Daphbolide A) (3)

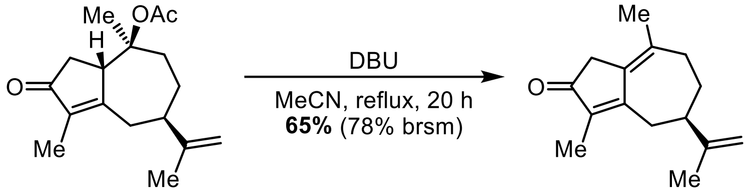

8a Daphbolide A (**3**)

A dry 25 mL Schlenk flask equipped with a Teflon-coated magnetic stirring bar was charged with a solution of **8a** (100 mg, 362 µmol, 1.00 equiv) in dry acetonitrile (3.60 mL). Freshly distilled DBU (65.4 µL, 434 µmol, 1.20 equiv) was added dropwise to the stirring solution, and the reaction mixture was heated at reflux for 20 h. Saturated aqueous NH_4Cl (3 mL) was then added, and the aqueous layer was extracted with Et_2O (3 × 3 mL). The combined organic layers were washed with H_2O (3 × 3 mL), dried over $MgSO_4$, filtered, and concentrated under reduced pressure. The crude product was purified by flash column chromatography (SiO_2, *c*Hex/EtOAc 19:1 to 9:1) to afford daphbolide A (**3**, 50.9 mg, 235 µmol, 65% yield, 78% brsm) as a light-yellow oil.

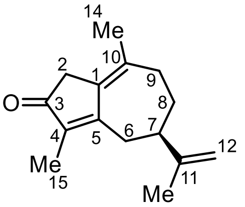

3

^{1}H NMR (400 MHz, $CDCl_3$): δ = 4.71 (s, 2H, H-12), 2.91 (s, 2H, H-2), 2.81 (dd, J = 16.0, 4.2 Hz, 1H, H_a-6), 2.67 (dd, J = 16.0, 9.4 Hz, 1H, H_b-6), 2.52 – 2.41 (m, 2H, H_a-9, H-7), 2.34 – 2.26 (m, 1H, H_b-9), 1.99 – 1.91 (m, 1H, H_a-8), 1.83 (s, 3H, H-14), 1.78 – 1.69 (m, 1H, H_b-8), 1.75 (s, 6H, H-13, H-15) ppm.

^{13}C{^{1}H} NMR (151 MHz, $CDCl_3$): δ = 204.8 (C-3), 167.2 (C-5), 149.7 (C-11), 138.2 (C-4), 136.5 (C-1), 131.4 (C-10), 109.6 (C-12), 43.2 (C-7), 40.6 (C-2), 34.7 (C-6), 34.5 (C-9), 32.5 (C-8), 24.0 (C-14), 20.5 (C-13), 8.5 (C-15) ppm.

HRMS (ESI, pos): *m/z* calculated for $C_{15}H_{20}ONa^+$ $[M+Na]^+$: 239.1406; found: 239.1410.

IR (ATR): $\tilde{\nu}$ = 3075 (w), 2965 (w), 2921 (m), 2857 (m), 1686 (ss), 1645 (m), 1596 (m), 1445 (m), 1388 (m), 1275 (w), 1223 (w), 1174 (w), 1175 (w), 1104 (w), 1073 (w), 1014 (w), 887 (m) cm^{-1}.

$[\alpha]_D^{24}$ = +42.30° (c = 1.00, $CHCl_3$).

NMR data of daphbolide A (3) in DMSO-*d*6

^{1}H NMR (700 MHz, DMSO-d_6): δ = 4.73 (s, 1H, H_a-12), 4.70 (s, 1H, H_b-12), 2.90 (d, *J* = 20.7 Hz, 1H, H_a-2), 2.85 (d, *J* = 20.7 Hz, 1H, H_b-2), 2.77 (dd, *J* = 16.1, 4.1 Hz, 1H, H-6), 2.71 (dd, *J* = 16.1, 9.1 Hz, 1H, H-6), 2.48 – 2.41 (m, 2H, H_b-9, H7), 2.31 (ddd, *J* = 17.6, 9.1, 2.8 Hz, 1H, H_a-9), 1.89 (ddt, *J* = 13.2, 8.5, 4.3 Hz, 1H, H_a-8), 1.81 (s, 3H, H-14), 1.73 (s, 3H, H-13), 1.72 – 1.66 (m, 1H, H_b-8), 1.66 (s, 3H, H-15) ppm.

^{13}C{^{1}H} NMR (176 MHz, DMSO-*d*6): δ = 203.0 (C-3), 166.3 (C-5), 149.4 (C-11), 137.1 (C-4), 136.2 (C-1), 130.9 (C-10), 109.4 (C-12), 42.5 (C-7), 40.0 (C-2), 33.8 (C-6), 33.8 (C-9), 31.9 (C-8), 23.7 (C-14), 20.2 (C-13), 8.1 (C-15) ppm.

Table S7. Comparison of ^{1}H and ^{13}C NMR data for isolated and synthetic daphbolide A (**3**)[a)]

No.	**Isolation**[b] **^{13}C NMR (150 MHz)** δ_C/ppm[c]	**Synthetic ^{13}C NMR (176 MHz)** δ_C/ppm[c]	Δ/ppm	**Isolation**[b] **^{1}H NMR (600 MHz)** δ_C/ppm[c] (*J* in Hz)	**Synthetic ^{1}H NMR (700 MHz)** δ_C/ppm[c] (*J* in Hz)	Δ/ppm
1	136.3	136.2	0.1			
2	40.1	40.0	0.1	2.85, d (20.7)<br>2.90, d (20.7)	2.85, d (20.7)<br>2.90, d (20.7)	0.00<br>0.00
3	203.1	203.0	0.1			
4	137.1	137.1	0.0			
5	166.3	166.3	0.0			
6	33.9	33.8	0.1	2.71, dd (16.1, 9.0)<br>2.77, dd (16.1, 4.4)	2.71, dd (16.1, 9.1)<br>2.77, dd (16.1, 4.1)	0.00<br>0.00
7	42.5	42.5	0.0	2.44, overlap	2.44, overlap	0.00
8	31.9	31.9	0.0	1.69, m<br>1.87, m	1.69, m<br>1.89, ddt (13.2, 8.5, 4.3)	0.00<br>0.02
9	33.8	33.8	0.0	2.31, ddd (17.5, 9.2, 3.4)<br>2.44, overlap	2.31, ddd (17.6, 9.1, 2.8)<br>2.44, overlap	0.00<br>0.00
10	130.9	130.9	0.0			
11	149.4	149.4	0.0			
12	109.5	109.4	0.1	4.70, brs<br>4.73, brs	4.70, s<br>4.73, s	0.00<br>0.00
13	20.2	20.2	0.0	1.73, s	1.73	0.00
14	23.7	23.7	0.0	1.81, s	1.81, s	0.00
15	8.1	8.1	0.0	1.66, s	1.66, s	0.00

a) All data were obtained in DMSO-*d*6. Overlapped signals were reported without designating multiplicity; b) Data from ref.[7]; c) Chemical shifts are reported relative to the corresponding residual non-deuterated solvent signal (DMSO-*d*6: ^{1}H = 2.50 ppm, ^{13}C = 39.5 ppm).

3.5 (5*R*,8*S*,8a*S*)-8-Hydroxy-3,8-dimethyl-5-(prop-1-en-2-yl)-4,5,6,7,8,8a-hexahydroazulen-2(*1H*)-one (9)

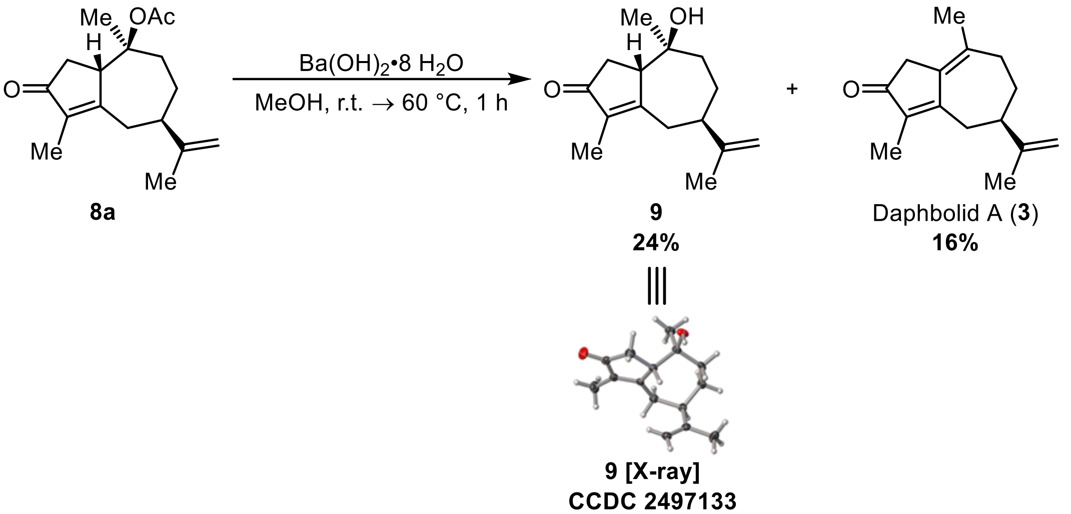

Acetate **8a** (213 mg, 771 μmol, 1.00 equiv) was dissolved in MeOH (7.7 mL). $Ba(OH)_2 \cdot 8H_2O$ (365 mg, 1.15 mmol, 1.50 equiv) was added in one portion at room temperature. The reaction mixture was stirred for 10 min, then heated to 60 °C and stirred for 1 h. The mixture was cooled to room temperature and purified directly by flash column chromatography (SiO_2, *c*Hex/EtOAc 9:1 to 4:1 to 3:2). Alcohol **9** (42.0 mg, 179 μmol, 24% yield) was obtained as a yellowish oil that solidified at 5 °C in the refrigerator. Daphbolide A (**3**, 26.0 mg, 120 μmol, 16% yield) was isolated as a yellowish oil.

NMR data of alcohol 9

^{1}H NMR (700 MHz, $CDCl_3$): δ = 4.82 – 4.81 (m, 1H), 4.66 – 4.65 (m, 1H), 3.08 – 3.05 (m, 1H), 2.87 (dd, *J* = 15.2, 8.3 Hz, 1H), 2.64 (dd, *J* = 15.3, 2.6 Hz, 1H), 2.48 (dd, *J* = 18.7, 6.5 Hz, 1H), 2.29 – 2.23 (m, 2H), 2.07 – 2.01 (m, 1H), 1.80 – 1.73 (m, 4H), 1.70 (d, *J* = 2.1 Hz, 3H), 1.66 – 1.59 (m, 2H), 1.10 (s, 3H) ppm.

^{13}C{^{1}H} NMR (176 MHz, $CDCl_3$): δ = 208.2, 173.8, 148.4, 137.7, 110.8, 73.9, 53.7, 44.2, 39.3, 38.5, 34.3, 27.9, 25.2, 21.1, 8.1 ppm.

HRMS (ESI, pos): *m/z* calculated for $C_{15}H_{22}O_2Na^+$ ($[M+Na]^+$): 257.1512; found: 257.1525.

The spectroscopic data are consistent with those reported in the literature.[8]

Single crystals of **9** suitable for X-ray diffraction were grown by slow evaporation of a solution in CH_2Cl_2 and cyclohexane at ambient temperature.

3.6 3,8-Dimethyl-5-(propan-2-ylidene)-4,5,6,7-tetrahydroazulen-2(1*H*)-one (10)

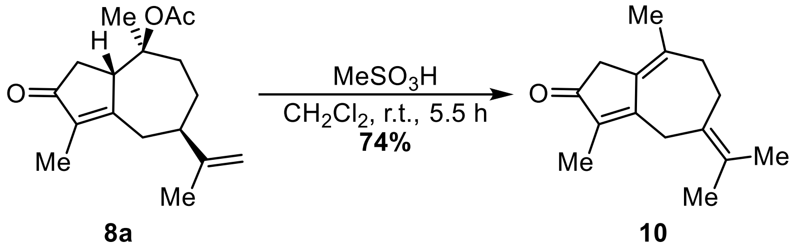

Acetate **8a** (100 mg, 362 µmol, 1.00 equiv) was dissolved in CH_2Cl_2 (3.5 mL). Methanesulfonic acid (500 µL, 725 µmol, 2.00 equiv) was added at room temperature, and the reaction mixture was stirred for 3.5 h before an additional portion of methanesulfonic acid (250 µL, 362 µmol, 1.00 equiv) was added. After 2 h, the reaction mixture was diluted with CH_2Cl_2 (10 mL) and quenched with saturated aqueous $NaHCO_3$ (10 mL). The layers were separated, the aqueous phase was extracted with EtOAc (3 × 10 mL), and the combined organic layers were washed with H_2O (1 × 20 mL) and saturated aqueous NaCl (1 × 20 mL), dried over $MgSO_4$, filtered, and concentrated under reduced pressure. The crude product was purified by flash column chromatography (SiO_2, *c*Hex/EtOAc 19:1 to 9:1) to afford compound **10** (58.0 mg, 268 µmol, 74% yield) as a pale yellow solid.

^{1}H NMR (600 MHz, $CDCl_3$): δ = 3.43 (s, 2H), 2.86 (s, 2H), 2.50 (s, 4H), 1.86 (s, 3H), 1.83 (s, 3H), 1.71 (s, 3H), 1.63 (s, 3H) ppm.

^{13}C{^{1}H} NMR (151 MHz, $CDCl_3$): δ = 205.5, 167.2, 135.9, 135.5, 131.9, 126.9, 126.5, 39.8, 32.7, 31.7, 31.4, 24.1, 20.9, 20.6, 8.4 ppm.

HRMS (ESI, pos): *m/z* calculated for $C_{15}H_{21}O^+$ ($[M+H]^+$): 217.1587, found: 217.1604.

3.7 (*S*)-5-Hydroxy-3,8-dimethyl-5-(prop-1-en-2-yl)-4,5,6,7-tetrahydroazulen-2(1*H*)-one (Daphbolide B) (11)

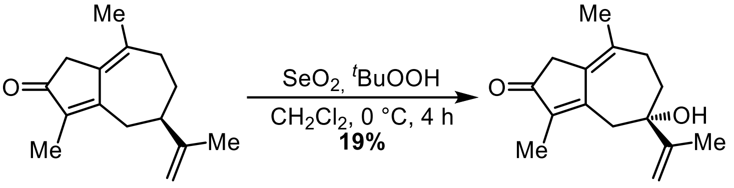

A mixture of SeO_2 (7.70 mg, 69.0 µmol, 1.50 equiv) and TBHP (5.5 M in decane, 20.0 µL, 92.0 µmol, 2.00 equiv) was stirred at room temperature for 20 min. The mixture was cooled to 0 °C, and a solution of daphbolide A (**3**) (10.0 mg, 46.0 µmol, 1.00 equiv) in CH_2Cl_2 (0.42 mL) was added dropwise. The reaction mixture was stirred at 0 °C for 4 h, then quenched by addition of saturated aqueous $Na_2S_2O_3$ (2.5 mL) and stirred for an additional 20 min. CH_2Cl_2 (12 mL) was added, the layers were separated, and the organic layer was washed with H_2O (2 × 15 mL) and brine (1 × 15 mL), dried over anhydrous $MgSO_4$, filtered, and concentrated under reduced pressure. The crude product was purified by preparative TLC (SiO_2, *c*Hex/EtOAc 1:1) to afford daphbolide B (**11**, 2.00 mg, 8.60 µmol, 19% yield) as a yellowish oil.

^{1}H NMR (600 MHz, $CDCl_3$): δ = 5.00 (s, 1H), 4.86 – 4.84 (m, 1H), 3.07 (d, *J* = 14.9 Hz, 1H), 2.93 (d, *J* = 5.3 Hz, 2H), 2.88 (d, *J* = 14.8 Hz, 1H), 2.57 (dd, *J* = 17.8, 9.4 Hz, 1H), 2.30 (dd, *J* = 17.4, 8.9 Hz, 1H), 2.18 (ddd, *J* = 14.5, 9.3, 2.1 Hz, 1H), 1.89 – 1.84 (m, 1H), 1.87 (s, 3H), 1.83 (s, 3H), 1.79 (s, 3H) ppm.
^{13}C{^{1}H} NMR (151 MHz, $CDCl_3$): δ = 205.0, 164.1, 150.6, 139.2, 137.7, 131.8, 110.1, 76.7, 41.1, 40.4, 40.2, 31.4, 24.1, 19.1, 8.6 ppm.
HRMS (ESI, pos): *m/z* calculated for $C_{15}H_{20}O_2Na^+$ $[M+Na]^+$: 255.1356; found: 255.1390.
IR (ATR): $\tilde{\nu}$ = 2923, 2857, 1682, 1540, 1507, 1456, 913, 743 cm^{-1}.

3.8 (4a*R*,7*S*,8*S*)-8-Hydroxy-1,4a-dimethyl-7-(prop-1-en-2-yl)-4,4a,5,6,7,8-hexahydronaphthalen-2(3*H*)-one (12)

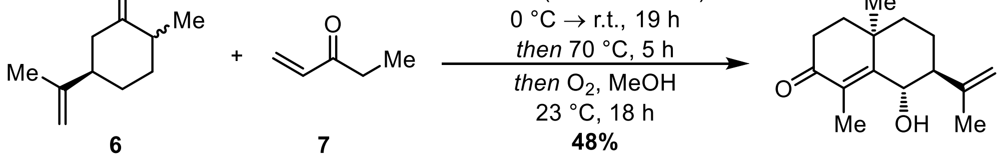

Enone **12** was synthesized according to a literature procedure:[1] A 50 mL Schlenk flask equipped with a Teflon-coated magnetic stirring bar was charged with KOH (10% in MeOH, 37.5 mL) and (+)-dihydrocarvone (**6**, 5.00 g, 5.39 mL, 32.8 mmol, 1.00 equiv). The solution was cooled

to 0 °C and stirred for 15 min. Ethyl vinyl ketone (**7**, 3.12 g, 3.93 mL, 39.4 mmol, 1.20 equiv) was added dropwise over 30 min at 0 °C, and the reaction mixture was stirred for an additional 30 min. The reaction mixture was warmed to room temperature and stirred for 19 h, then heated at reflux in MeOH for 5 h, cooled to room temperature, and oxygen was bubbled through the solution for 15 min before it was stirred under an oxygen atmosphere for 18 h (1 atm, balloon). The reaction mixture was concentrated to ca. 20 mL, diluted with EtOAc (20 mL), and quenched with saturated aqueous NH_4Cl (20 mL). The resulting white salt was filtered off, and the aqueous layer was extracted with EtOAc (2 × 50 mL). The combined organic layers were dried over $MgSO_4$, filtered, and concentrated under reduced pressure. The orange residue was purified by flash column chromatography (SiO_2, *c*Hex/EtOAc 5:1) to afford enone **12** (3.70 g, 15.8 mmol, 48% yield) as an orange oil.

^{1}H NMR (600 MHz, $CDCl_3$): δ = 4.91 (s, 1H), 4.80 (s, 1H), 4.37 (s, 1H), 2.63 (ddd, *J* = 17.9, 15.1, 5.3 Hz, 1H), 2.53 (s, 1H), 2.43 (ddd, *J* = 17.9, 4.8, 2.1 Hz, 1H), 2.25 – 2.17 (m, 1H), 1.88 (s, 3H), 1.83 (td, *J* = 14.1, 4.8 Hz, 2H), 1.74 (s, 3H), 1.62 (ddd, *J* = 13.1, 5.2, 2.0 Hz, 1H), 1.56 – 1.52 (m, 1H), 1.48 (dd, *J* = 13.4, 3.4 Hz, 1H), 1.39 (s, 3H), 1.36 (dt, *J* = 13.4, 4.0 Hz, 1H) ppm.
^{13}C{^{1}H} NMR (151 MHz, $CDCl_3$): δ = 200.3, 160.5, 145.6, 132.4, 111.8, 69.8, 48.0, 39.1, 35.4, 35.4, 34.5, 26.0, 23.3, 18.9, 10.7 ppm.

The spectroscopic data are consistent with those reported in literature.[1]

3.9 (4a*R*,7*S*,8*S*)-8-Hydroxy-1,4a-dimethyl-7-(prop-1-en-2-yl)-5,6,7,8-tetra-hydronaphthalen-2(4a*H*)-one (13)

Method A

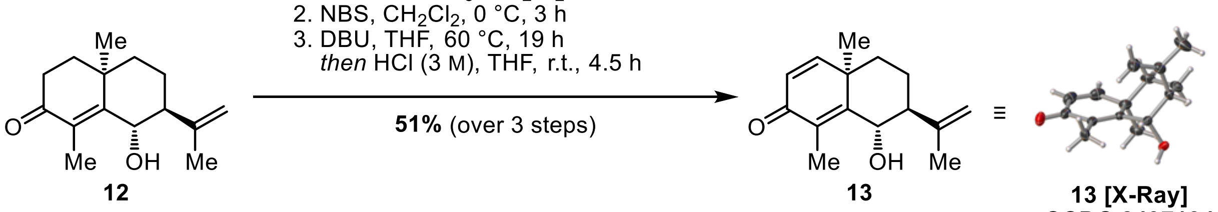

13 [X-Ray]
CCDC 2497134

Dienone **13** was synthesized according to a literature procedure:[1] Hydroxyenone **12** (1.56 g, 6.66 mmol, 1.00 equiv) was dissolved in dry CH_2Cl_2 (42 mL). The reaction mixture was cooled to 0 °C in an ice bath, and NEt_3 (3.37 g, 4.64 mL, 33.3 mmol, 5.00 equiv) and TMSOTf (4.00 g, 3.25 mL, 18.0 mmol, 2.70 equiv) were added successively. The solution was stirred at 0 °C for

2.5 h until complete consumption of the starting material was observed by TLC. *N*-Bromosuccinimide (1.54 g, 8.65 mmol, 1.30 equiv) was then added portionwise at 0 °C, and the reaction mixture was stirred at this temperature for 3 h. The solution was warmed to room temperature and concentrated under reduced pressure. Dry THF (16 mL) was added, followed by dropwise addition of DBU (10.1 g, 9.94 mL, 66.6 mmol, 10.0 equiv) at room temperature. The reaction mixture was heated to reflux (60 °C) for 19 h and then cooled to room temperature. 3 M aqueous HCl (16 mL) was added and the mixture was stirred for 4.5 h, after which a second portion of 3 M aqueous HCl (16 mL) was added and stirring was continued for an additional 2 h. The mixture was diluted with EtOAc (50 mL) and washed with saturated aqueous $NaHCO_3$ (3 × 30 mL) and brine (1 × 50 mL). The aqueous phase was extracted with EtOAc (30 mL). The combined organic layers were dried over $MgSO_4$, filtered, and concentrated under reduced pressure. Purification by flash column chromatography (SiO_2, *c*Hex/EtOAc 4:1) afforded **13** (789 mg, 3.40 mmol, 51% yield) as a yellowish solid. Crystallization from *c*Hex/CH_2Cl_2 provided colorless crystals suitable for X-ray analysis.

^{1}H NMR (400 MHz, $CDCl_3$): δ = 6.71 (d, *J* = 9.8 Hz, 1H), 6.18 (d, *J* = 9.8 Hz, 1H), 5.07 (s, 1H), 4.79 (s, 1H), 4.40 (s, 1H), 2.62 (s, 1H), 2.36 – 2.24 (m, 1H), 2.03 (s, 3H), 1.74 (s, 3H), 1.68 – 1.51 (m, 4H), 1.46 (s, 3H) ppm.
^{13}C{^{1}H} NMR (151 MHz, $CDCl_3$): δ = 187.3, 158.6, 156.8, 145.0, 133.7, 125.4, 112.2, 70.4, 48.4, 40.3, 32.0, 27.6, 23.5, 19.3, 11.2 ppm.
IR (ATR): $\tilde{\nu}$ = 3403, 2920, 2250, 1653, 1617, 1453, 1402, 1369, 1325, 1036, 1020, 1003, 891, 835, 731 cm^{-1}.

Single crystals of **13** suitable for X-ray diffraction were grown by slow evaporation of a solution in CH_2Cl_2 and cyclohexane at ambient temperature.

The spectroscopic data are consistent with those reported in literature.[1]

Method B

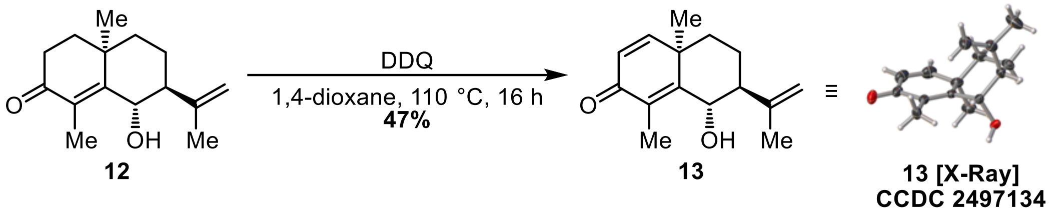

Hydroxyenone **12** (426 mg, 1.82 mmol, 1.00 equiv) was dissolved in dry 1,4-dioxane (24 mL), and DDQ (826 mg, 3.64 mmol, 2.00 equiv) was added. The resulting suspension was heated at reflux for 16 h. After completion of the reaction, the solvent was concentrated under reduced pressure to one-fourth of the original volume. The residue was adsorbed on basic alumina and washed with 30% EtOAc in cyclohexane until TLC analysis showed no traces of product. Removal of the solvent under reduced pressure afforded **13** as a yellow oil (198 mg, 0.85 mmol, 47% yield).

3.10 (4*S*,5*S*,8*R*)-3,8-Dimethyl-5-(prop-1-en-2-yl)-4,5,6,7,8,8a-hexahydro-4,8-epoxyazulen-2(1*H*)-one (14)

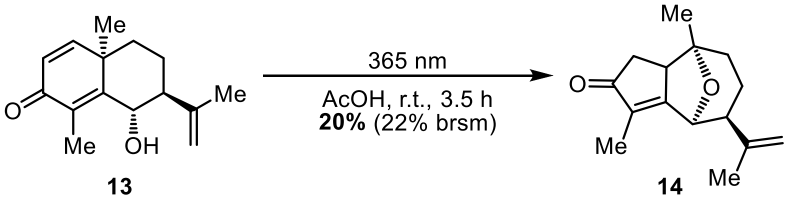

Dienone **13** (93.0 mg, 400 µmol, 1.00 equiv) was dissolved in glacial AcOH (40 mL), and the resulting solution was stirred at room temperature for 3.5 h while being irradiated with a UV LED (365 nm, 30 W). The reaction mixture was concentrated under reduced pressure, and the residue was purified by flash column chromatography (SiO_2, *c*Hex/EtOAc 4:1) to afford **14** (18.6 mg, 80.0 µmol, 20% yield, 22% brsm) as a yellow oil.

^{1}H NMR (700 MHz, $CDCl_3$): δ = 4.86 (s, 1H), 4.76 (s, 1H), 4.68 (d, J = 3.8 Hz, 1H), 3.06 – 2.95 (m, 1H), 2.73 – 2.64 (m, 1H), 2.58 (dd, J = 16.0, 6.4 Hz, 1H), 2.28 (dd, J = 16.0, 5.6 Hz, 1H), 1.88 – 1.83 (m, 3H), 1.76 (s, 3H), 1.71 (d, J = 2.3 Hz, 3H), 1.67 – 1.65 (m, 1H), 1.28 (s, 3H) ppm.

^{13}C{^{1}H} NMR (176 MHz, $CDCl_3$): δ = 210.2, 177.0, 145.1, 133.2, 111.9, 78.3, 77.8, 48.0, 45.4, 42.4, 37.5, 23.3, 22.5, 22.1, 9.5 ppm.

HRMS (ESI, pos): *m/z* calculated for $C_{15}H_{20}O_2Na^+$ $[M+Na]^+$: 255.1356; found: 255.1361.

The spectroscopic data are consistent with those reported in the literature.[8]

3.11 (4a*R*,7*S*,8*S*)-8-((*tert*-Butyldimethylsilyl)oxy)-1,4a-dimethyl-7-(prop-1-en-2-yl)-5,6,7,8-tetrahydronaphthalen-2(4a*H*)-one (15)

Method A

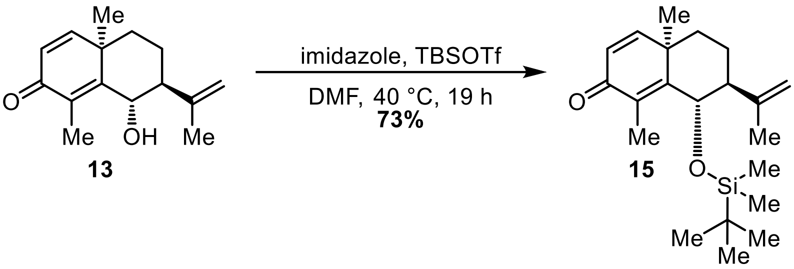

Alcohol **13** (184 mg, 0.792 mmol, 1.00 equiv) was dissolved in dry DMF (1.0 mL). The solution was stirred for 10 min, then imidazole (269 mg, 3.96 mmol, 5.00 equiv) and TBSOTf (1.05 g, 910 µL, 3.98 mmol, 5.00 equiv) were added slowly. The reaction mixture was stirred at 40 °C for 19 h. After cooling to room temperature, H_2O (3 mL) was added, the layers were separated, and the aqueous layer was extracted with EtOAc (3 × 3 mL) and CH_2Cl_2 (2 mL). The combined organic layers were washed with saturated aqueous NH_4Cl (3 mL) and brine (3 mL), dried over $MgSO_4$, filtered, and concentrated under reduced pressure. The residue was purified by flash column chromatography (SiO_2, *c*Hex/EtOAc 9:1) to afford TBS ether **15** (200 mg, 0.578 mmol, 73% yield) as a yellow oil.

^{1}H NMR (700 MHz, $CDCl_3$): δ = 6.66 (d, *J* = 9.8 Hz, 1H), 6.16 (d, *J* = 9.8 Hz, 1H), 5.05 (s, 1H), 4.77 (s, 1H), 4.42 (s, 1H), 2.46 (d, *J* = 5.3 Hz, 1H), 2.35 (tdd, *J* = 13.9, 5.3, 3.9 Hz, 1H), 2.01 (s, 3H), 1.72 (s, 3H), 1.68 – 1.64 (m, 1H), 1.57 (td, *J* = 13.6, 3.7 Hz, 1H), 1.50 (dt, *J* = 13.0, 3.2 Hz, 1H), 1.44 (s, 3H), 0.89 (s, 9H), 0.14 (s, 3H), 0.04 (s, 3H) ppm.
^{13}C{^{1}H} NMR (176 MHz, $CDCl_3$): δ = 187.7, 159.0, 156.8, 144.5, 132.2, 125.3, 112.1, 70.8, 49.9, 40.7, 32.4, 27.1, 26.1, 24.0, 18.8, 18.3, 11.7, -4.1, -4.4 ppm.
HRMS (ESI, pos): *m/z* calculated for $C_{21}H_{34}O_2SiNa^+$ $[M+Na]^+$: 369.2220; found: 369.2232.
IR (ATR): $\tilde{\nu}$ = 2952, 2928, 2885, 2856, 1660, 1631, 1471, 1461, 1364, 1254, 1092, 1054, 1005, 907, 883, 862, 835, 789, 775, 732 cm^{-1}.

Method B

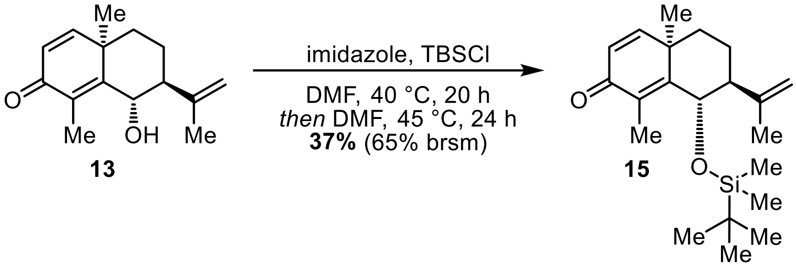

Alcohol **13** (207 mg, 0.891 mmol, 1.00 equiv) was dissolved in dry DMF (1.1 mL). The solution was stirred for 10 min, then imidazole (304 mg, 4.46 mmol, 5.00 equiv) and TBSCl (670 mg, 4.46 mmol, 5.00 equiv) were added slowly. The reaction mixture was stirred at 40 °C for 20 h. A second portion of imidazole (304 mg, 4.46 mmol, 5.00 equiv) and TBSCl (670 mg, 4.46 mmol, 5.00 equiv) was then added, and the mixture was stirred at 45 °C for an additional 24 h. After cooling to room temperature, H_2O (3 mL) was added, the layers were separated, and the aqueous layer was extracted with Et_2O (3 × 2 mL) and CH_2Cl_2 (2 mL). The combined organic layers were washed with saturated aqueous NH_4Cl (3 mL) and brine (3 mL), dried over $MgSO_4$, filtered, and concentrated under reduced pressure. The residue was purified by flash column chromatography (SiO_2, *c*Hex/EtOAc 4:1 to 2:1) to afford TBS ether **15** (114 mg, 329 µmol, 37% yield, 65% brsm) as a yellow oil.

Method C

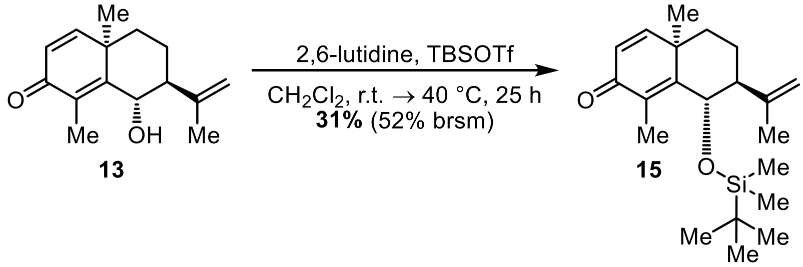

Alcohol **13** (146 mg, 628 µmol, 1.00 equiv) was dissolved in dry CH_2Cl_2 (1.85 mL). The solution was cooled to 0 °C, and 2,6-lutidine (110 µL, 101 mg, 943 µmol, 1.50 equiv) was added slowly. The reaction mixture was warmed to room temperature and stirred for 5 min. TBSOTf (217 µL, 249 mg, 943 µmol, 1.50 equiv) was then added, and the mixture was stirred for 17 h before being heated to 40 °C for an additional 8 h. After cooling to room temperature, saturated aqueous $NaHCO_3$ (3 mL) was added, the layers were separated, and the aqueous layer was extracted with CH_2Cl_2 (3 × 3 mL). The combined organic layers were washed successively with saturated aqueous $NaHSO_4$ (3 mL), saturated aqueous $NaHCO_3$ (3 mL), and brine (3 mL), dried over $MgSO_4$, filtered, and concentrated under reduced pressure. The residue was purified

by flash column chromatography (SiO_2, *c*Hex/EtOAc 9:1) to afford TBS ether **15** (68.3 mg, 197 µmol, 31% yield, 52% brsm) as a yellow oil.

3.12 (3a*R*,4*S*,7*S*,8*S*)-8-((*tert*-Butyldimethylsilyl)oxy)-1,4-dimethyl-2-oxo-7-(prop-1-en-2-yl)-2,3,3a,4,5,6,7,8-octahydroazulen-4-yl acetate (16)

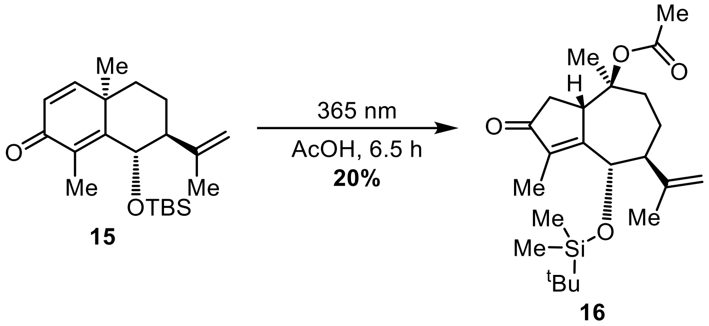

A solution of dienone **15** (50.0 mg, 144 µmol, 1.00 equiv) in AcOH (14.5 mL) was stirred and irradiated with a UV LED (365 nm, 30 W) for 6.5 h. The solvent was then removed under reduced pressure, and the residue was purified by flash column chromatography (SiO_2, *c*Hex/EtOAc 9:1 to 2:1) to afford guaiane **16** (12.0 mg, 29.5 µmol, 20% yield) as a yellow oil.

^{1}H NMR (700 MHz, $CDCl_3$): δ = 4.87 (s, 1H), 4.74 (d, *J* = 8.5 Hz, 1H), 4.64 (s, 1H), 3.53 (dt, *J* = 6.2, 3.0 Hz, 1H), 2.53 (dd, *J* = 18.7, 6.6 Hz, 1H), 2.36 (td, *J* = 9.0, 4.7 Hz, 1H), 2.20 (ddd, *J* = 14.1, 8.6, 2.5 Hz, 1H), 2.12 (dd, *J* = 18.7, 3.6 Hz, 1H), 2.06 (s, 3H), 1.90 (d, *J* = 2.0 Hz, 3H), 1.76 (s, 3H), 1.72 – 1.65 (m, 1H), 1.64 – 1.58 (m, 2H), 1.47 (s, 3H), 0.86 (s, 9H), 0.07 (s, 3H), -0.02 (s, 3H) ppm.
^{13}C{^{1}H} NMR (176 MHz, $CDCl_3$): δ = 208.1, 173.8, 170.2, 146.0, 140.3, 112.7, 85.8, 72.1, 51.8, 49.5, 38.4, 33.7, 26.1, 24.8, 23.2, 22.8, 22.0, 18.5, 10.0, –4.2, –4.6 ppm.
HRMS (ESI, pos): *m/z* calculated for $C_{23}H_{38}O_4SiNa^+$ $[M+Na]^+$: 429.2432; found: 429.2444.
IR (ATR): $\tilde{\nu}$ = 2952, 2928, 2857, 1733, 1706, 1638, 1472, 1368, 1323, 1240, 1181, 1139, 1092, 1048, 1010, 938, 889, 868, 836, 775 cm^{-1}.

3.13 (3a*R*,4*S*,7*S*,8*S*)-8-((*tert*-Butyldimethylsilyl)oxy)-1,4-dimethyl-2-oxo-7-(prop-1-en-2-yl)-2,3,3a,4,5,6,7,8-octahydroazulen-4-yl acetate (16) and 2-(4-Hydroxy-2,3-dimethylphenethyl)-3-methylbut-2-enal (17)

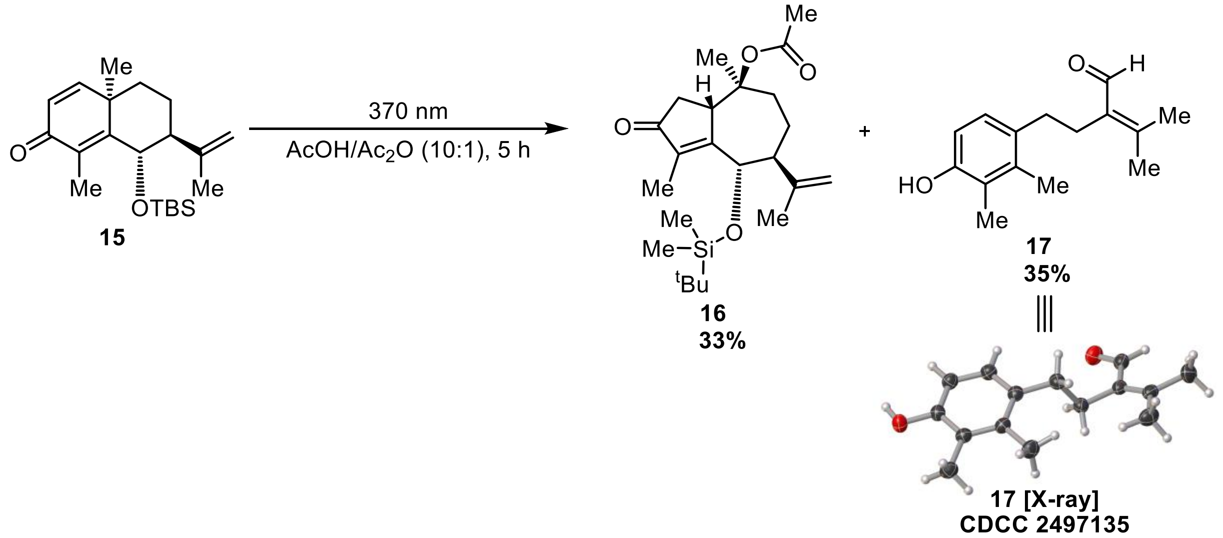

A solution of dienone **15** (575 mg, 1.66 mmol, 1.00 equiv) in AcOH (100 mL) and Ac_2O (10 mL) was stirred and irradiated with a KESSIL UV lamp (370 nm) for 5 h. The reaction mixture was concentrated under reduced pressure, and the residue was purified by flash column chromatography (SiO_2, *c*Hex/EtOAc 9:1 to 2:1) to afford guaiane **16** (221 mg, 0.543 mmol, 33% yield) as a yellow oil and phenol derivative **17** (132 mg, 0.568 mmol, 35% yield) as colorless crystals.

Analytical data of phenol 17:

^{1}H NMR (700 MHz, $CDCl_3$): δ = 10.15 (s, 1H), 6.84 (d, *J* = 8.1 Hz, 1H), 6.57 (d, *J* = 8.1 Hz, 1H), 4.62 (s, 1H), 2.53 (dd, *J* = 10.0, 6.1 Hz, 2H), 2.45 (dd, *J* = 10.1, 6.1 Hz, 2H), 2.28 (s, 3H), 2.18 (s, 6H), 1.88 (s, 3H) ppm.

^{13}C{^{1}H} NMR (151 MHz, $CDCl_3$): δ = 191.1, 155.5, 151.9, 136.6, 136.4, 132.6, 127.4, 122.8, 112.3, 33.0, 27.3, 23.3, 19.5, 15.7, 12.2 ppm.

HRMS (ESI, pos): *m/z* calculated for $C_{15}H_{20}O_2Na^+$ $[M+Na]^+$: 255.1356; found: 255.1375.

Single crystals of **17** suitable for X-ray diffraction were grown by slow evaporation of a solution in CH_2Cl_2 and cyclohexane at ambient temperature.

3.14 (4*S*,5*S*,8*S*,8a*S*)-4-((*tert*-Butyldimethylsilyl)oxy)-8-hydroxy-3,8-dimethyl-5-(prop-1-en-2-yl)-4,5,6,7,8,8a-hexahydroazulen-2(1*H*)-one (18)

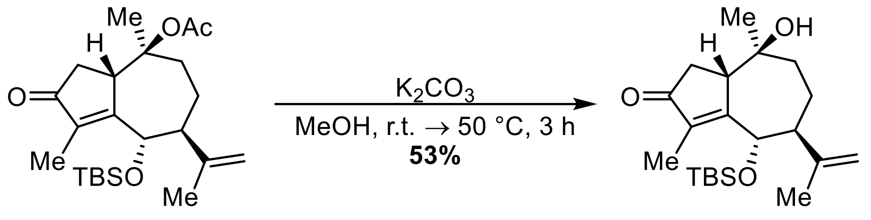

Acetate **16** (20.0 mg, 49.2 µmol, 1.00 equiv) was dissolved in MeOH (0.70 mL). K_2CO_3 (15.0 mg, 109 µmol, 2.21 equiv) was added at room temperature, and the reaction mixture was stirred for 45 min before a second portion of K_2CO_3 (15.0 mg, 109 µmol, 2.21 equiv) was added. After 15 min, the reaction mixture was heated to 50 °C. After an additional 50 min, a third portion of K_2CO_3 (15.0 mg, 109 µmol, 2.21 equiv) was added, and the mixture was stirred for a further 70 min. The reaction mixture was cooled to room temperature, filtered through cotton, rinsed with EtOAc, and concentrated under reduced pressure. The solid residue was dissolved in EtOAc, filtered to remove inorganic salts, rinsed with EtOAc, and concentrated under reduced pressure. The crude product was purified by flash column chromatography (SiO_2, *c*Hex/EtOAc 9:1 to 2:1) to afford alcohol **18** (9.51 mg, 26.1 µmol, 53% yield) as a colorless solid.

Note: Alcohol **18** was characterized only by ^{1}H and ^{13}C NMR.

^{1}H NMR (700 MHz, $CDCl_3$): δ = 4.90 (d, *J* = 7.7 Hz, 1H), 4.85 (t, *J* = 1.6 Hz, 1H), 4.62 (td, *J* = 1.4, 0.8 Hz, 1H), 3.05 – 3.03 (m, 1H), 2.50 (dd, *J* = 18.8, 6.5 Hz, 1H), 2.40 (td, *J* = 7.8, 4.8 Hz, 1H), 2.23 (dd, *J* = 18.8, 3.2 Hz, 1H), 1.92 – 1.88 (m, 1H), 1.88 (d, *J* = 2.1 Hz, 3H), 1.80 (ddd, *J* = 10.1, 4.8, 2.6 Hz, 1H), 1.77 (s, 3H), 1.68 (ddd, *J* = 12.7, 9.9, 2.7 Hz, 1H), 1.55 (ddd, *J* = 14.0, 9.0, 2.6 Hz, 1H), 1.20 (s, 3H), 0.87 (s, 9H), 0.08 (s, 3H), 0.00 (s, 3H) ppm.
^{13}C{^{1}H} NMR (176 MHz, $CDCl_3$): δ = 208.9, 173.9, 146.0, 139.8, 112.3, 74.0, 71.7, 52.2, 50.9, 38.6, 29.9, 26.0, 26.0, 23.6, 22.0, 18.4, 9.6, -4.5 ppm.

3.15 (4*S*,5*S*)-4-((*tert*-Butyldimethylsilyl)oxy)-3,8-dimethyl-5-(prop-1-en-2-yl)-4,5,6,7-tetrahydroazulen-2(1*H*)-one (21)

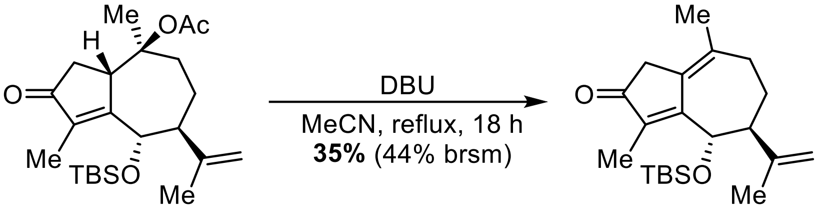

16 **21**

Acetate **16** (103 mg, 0.253 mmol, 1.00 equiv) was dissolved in MeCN (2.6 mL) in a heat-gun-dried Schlenk tube under argon. DBU (193 mg, 189 µL, 1.27 mmol, 5.00 equiv) was added dropwise at room temperature. The reaction mixture was heated to 80 °C and stirred for 18 h. Upon cooling to room temperature, the reaction was quenched by addition of saturated aqueous NH_4Cl (3 mL). The aqueous layer was extracted with CH_2Cl_2 (3 × 3 mL), and the combined organic layers were dried over Na_2SO_4, filtered, and concentrated under reduced pressure. The residue was purified by flash column chromatography (SiO_2, *c*Hex/EtOAc 9:1) to afford dienone **21** (30.0 mg, 87.0 µmol, 35% yield, 44% brsm) as a yellow oil.

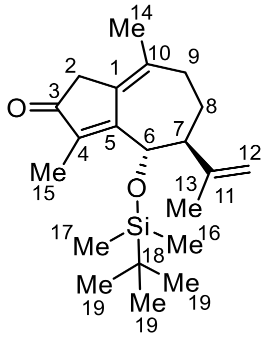

21

^{1}H NMR (700 MHz, $CDCl_3$): δ = 4.79 – 4.78 (m, 1H, H-6), 4.76 – 4.75 (m, 1H, H-12_a), 4.64 (dq, *J* = 1.7, 0.8 Hz, 1H, H-12_b), 3.06 – 2.95 (m, 1H, H-8_a), 2.94 (d, *J* = 3.4 Hz, 2H, H-2), 2.72 (ddd, *J* = 11.3, 6.6, 1.2 Hz, 1H, H-7), 2.07 – 2.04 (m, 1H, H-8_b), 2.02 – 1.97 (m, 1H, H-9), 1.86 (s, 3H, H-14), 1.77 (s, 3H, H-15), 1.71 (dd, *J* = 1.4, 0.8 Hz, 3H, H-13), 1.67 (dtd, *J* = 14.2, 11.2, 1.3 Hz, 1H, H-9), 0.83 (s, 9H, H-19), 0.06 (s, 3H, H-16 or H-17), –0.13 (s, 3H, H-17 or H-16) ppm.

^{13}C{^{1}H} NMR (176 MHz, $CDCl_3$): δ = 206.2, 166.6, 146.7, 140.0, 137.6, 128.7, 111.4, 72.1, 53.3, 40.7, 32.7, 31.6, 29.9, 27.1, 25.7, 24.0, 22.0, 18.0, 8.9, –4.8 ppm.

HRMS (ESI, pos): *m*/*z* calculated for $C_{21}H_{34}O_2SiNa^+$ [M+Na]$^+$: 369.2220; found: 369.2230.

3.16 (3S,3aS,6R,6aR,9bS)-6-hydroxy-3,6,9-trimethyl-3a,5,6,6a,7,9b-hexahydroazuleno[4,5-b]furan-2,8(3H,4H)-dione (24)

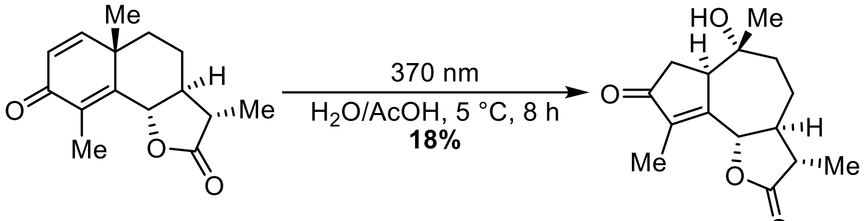

α-santonin (**23**) **24**

Guaiane **24** was synthesized according to a literature known procedure:[9] A one-necked flask was charged with α-santonin (**23**, 1.00 g, 4.06 mmol, 1.00 equiv), H_2O (70 mL), and AcOH (15 mL). The reaction mixture was degassed by bubbling argon through the solution for 5 min, placed in an ice bath, and irradiated with 370 nm KESSIL UV light for 8 h. The solvent was removed under reduced pressure, and the residue was purified by flash column chromatography (SiO_2, *c*Hex/EtOAc 1:1 to EtOAc) to afford guaiane **24** (189 mg, 0.715 mmol, 18% yield) as a white solid.

^{1}H NMR (700 MHz, $CDCl_3$): δ = 4.81 (d, *J* = 11.0 Hz, 1H), 3.25 – 3.20 (m, 1H), 2.60 (ddd, *J* = 19.7, 2.9, 0.8 Hz, 1H), 2.55 (dd, *J* = 19.7, 6.2 Hz, 1H), 2.32 (dq, *J* = 12.2, 6.9 Hz, 1H), 2.17 – 2.11 (m, 1H), 2.10 – 2.05 (m, 2H), 1.91 – 1.89 (m, 3H), 1.80 (td, *J* = 13.7, 12.7, 4.5 Hz, 1H), 1.49 – 1.39 (m, 1H), 1.29 (d, *J* = 6.9 Hz, 3H), 0.97 (s, 3H) ppm.

^{13}C{^{1}H} NMR (176 MHz, $CDCl_3$): δ = 207.8, 177.3, 161.3, 143.3, 81.6, 74.6, 50.6, 48.6, 45.5, 41.6, 37.3, 26.0, 21.5, 12.6, 9.6 ppm.

The spectroscopic data are consistent with those reported in literature.[9]

3.17 (3*S*,3a*S*,6*R*,6a*R*,9b*S*)-3,6,9-Trimethyl-2,8-dioxo-2,3,3a,4,5,6,6a,7,8,9b-decahydroazuleno[4,5-b]furan-6-yl acetate (25)

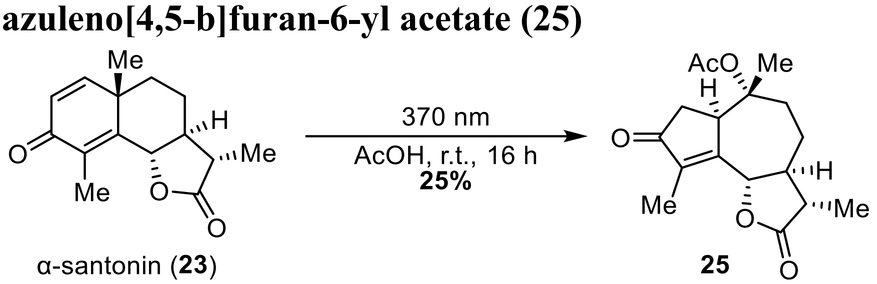

α-santonin (**23**) **25**

Acetate **25** was synthesized according to a literature known procedure:[10] A one-necked flask was charged with α-santonin (**23**, 500 mg, 2.03 mmol, 1.00 equiv) and AcOH (48 mL). The reaction mixture was degassed by bubbling argon through the solution for 5 min and then irradiated with 370 nm KESSIL UV light for 16 h. The solvent was removed under reduced pressure, and the resulting oily residue was dissolved in hot MeOH (4 mL) and stored in a freezer

for 20 h. The resulting solid was collected by filtration to afford **25** (153 mg, 0.499 mmol, 25% yield) as a white solid.

^{1}H NMR (400 MHz, $CDCl_3$): δ = 4.80 (d, *J* = 11.0 Hz, 1H), 4.19 – 4.12 (m, 1H), 2.62 (td, *J* = 13.6, 4.6 Hz, 1H), 2.50 (dd, *J* = 19.5, 6.3 Hz, 1H), 2.39 (dd, *J* = 19.6, 2.8 Hz, 1H), 2.35 – 2.27 (m, 1H), 2.26 – 2.13 (m, 2H), 2.11 – 2.04 (m, 1H), 2.00 (s, 3H), 1.91 – 1.89 (m, 3H), 1.45 (tdd, *J* = 14.4, 11.1, 3.6 Hz, 1H), 1.28 (d, *J* = 6.8 Hz, 3H), 1.08 (s, 3H) ppm.
^{13}C{^{1}H} NMR (176 MHz, $CDCl_3$): δ = 207.0, 177.1, 170.4, 160.9, 143.4, 85.7, 81.4, 48.4, 47.4, 41.5, 38.1, 37.0, 25.5, 22.4, 20.2, 12.6, 9.6 ppm.

The spectroscopic data are consistent with those reported in the literature.[10]

3.18 (3*S*,3a*S*,9b*S*)-3,6,9-Trimethyl-3a,5,7,9b-tetrahydroazuleno[4,5-b]furan-2,8(3*H*,4*H*)-dione (26)

Method A

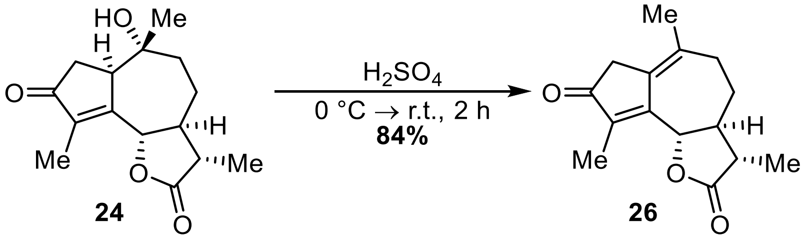

Diene **26** was synthesized according to a literature known procedure.[9] Alcohol **24** (50.0 mg, 0.189 mmol, 1.00 equiv) was added portionwise to H_2SO_4 (2.5 mL) at 0 °C over 10 min. The reaction mixture was stirred at this temperature for an additional 10 min, then warmed to room temperature and stirred for 2 h. The mixture was poured into an ice water mixture and extracted with CH_2Cl_2 (3 × 5 mL). The combined organic layers were washed successively with 1 M aqueous NaOH (1 × 10 mL) and brine (1 ×), dried over $MgSO_4$, filtered, and concentrated under reduced pressure to afford dienone **26** (39.0 mg, 0.158 mmol, 84% yield) as a white to pale yellow solid.

^{1}H NMR (700 MHz, $CDCl_3$): δ = 5.22 (d, *J* = 10.6 Hz, 1H), 2.96 (d, *J* = 3.7 Hz, 2H), 2.62 (ddd, *J* = 15.7, 8.9, 6.6 Hz, 1H), 2.40 (dq, *J* = 12.0, 7.0 Hz, 1H), 2.26 (dt, *J* = 16.2, 5.5 Hz, 1H), 2.23 – 2.13 (m, 2H), 2.02 (s, 3H), 1.88 (s, 3H), 1.71 (ddt, *J* = 11.0, 8.8, 5.7 Hz, 1H), 1.27 (d, *J* = 7.0 Hz, 3H) ppm.
^{13}C{^{1}H} NMR (176 MHz, $CDCl_3$): δ = 204.3, 177.5, 160.7, 139.3, 132.9, 129.5, 80.3, 47.6, 42.3, 40.3, 32.7, 26.9, 24.4, 12.9, 9.8 ppm.

The spectroscopic data are consistent with those reported in the literature.[9]

Method B

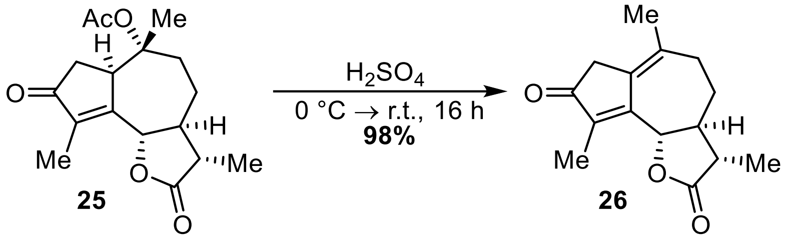

Diene **26** was synthesized according to a literature known procedure.[9] Acetate **25** (150 mg, 0.490 mmol, 1.00 equiv) was added portionwise to H_2SO_4 (6.5 mL) at 0 °C over 10 min. The reaction mixture was stirred at this temperature for an additional 10 min, then warmed to room temperature and stirred for 16 h. The mixture was poured into an ice water mixture and extracted with CH_2Cl_2 (3 × 5 mL). The combined organic layers were washed successively with 1 M aqueous NaOH (1 × 10 mL) and brine (1 × 10 mL), dried over $MgSO_4$, filtered, and concentrated under reduced pressure to afford dienone **26** (119 mg, 0.483 mmol, 98% yield) as a white to pale yellow solid.

Method C

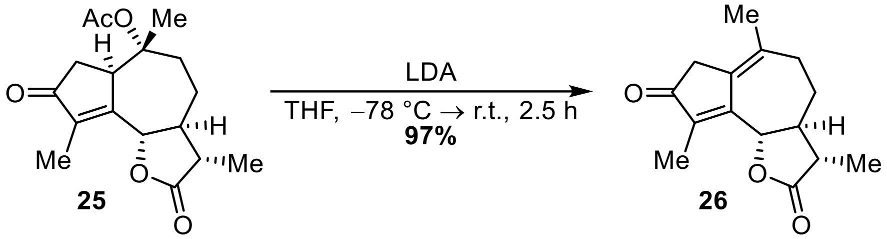

Acetate **25** (20.0 mg, 65.0 μmol, 1.00 equiv) was dissolved in dry THF (1.56 mL). LDA [prepared from *n*BuLi (2.50 M, 91.4 μL, 228 μmol, 3.50 equiv) and diisopropylamine (32.2 μL, 228 μmol, 3.50 equiv) in dry THF (1.3 mL) at −78 °C] was added dropwise at −78 °C. The reaction mixture was stirred at this temperature for 2 h and then allowed to warm to room temperature. After an additional 30 min of stirring, 1 M aqueous HCl (5 mL) was added slowly. The aqueous layer was extracted with EtOAc (3 × 5 mL), and the combined organic layers were dried over $MgSO_4$, filtered, and concentrated under reduced pressure to afford dienone **26** (15.5 mg, 63 μmol, 97% yield) as a white to pale yellow solid.

3.19 5-Ethyl-3,8-dimethyl-6,7-dihydroazulen-2(1*H*)-one (27)

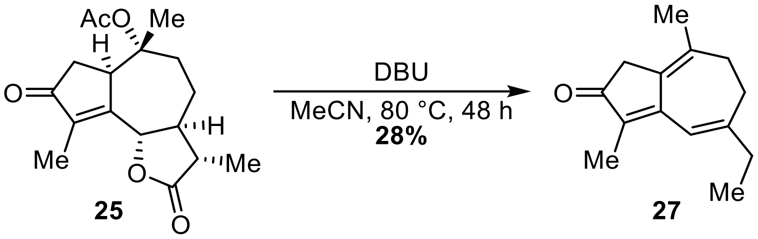

Acetate **25** (22.0 mg, 72.0 µmol, 1.00 equiv) was dissolved in dry MeCN (0.65 mL). DBU (32.2 µL, 32.8 mg, 215 µmol, 3.00 equiv) was added at room temperature, and the reaction mixture was heated to 80 °C and stirred for 48 h. Saturated aqueous NH_4Cl (2 mL) was then added, the aqueous layer was extracted with CH_2Cl_2 (3 × 2 mL), and the combined organic layers were dried over $MgSO_4$, filtered, and concentrated under reduced pressure. The residue was purified by flash column chromatography (SiO_2, *c*Hex/EtOAc 4:1) to afford trienone **27** (4.00 mg, 19.8 µmol, 28% yield) as an orange oil.

^{1}H NMR (400 MHz, $CDCl_3$): δ = 6.29 (s, 1H), 2.96 (s, 2H), 2.47 – 2.35 (m, 4H), 2.27 (q, *J* = 7.4 Hz, 2H), 1.86 (s, 3H), 1.83 (s, 3H), 1.12 (t, *J* = 7.4 Hz, 3H) ppm.
^{13}C{^{1}H} NMR (176 MHz, $CDCl_3$): δ = 205.0, 159.7, 155.8, 137.1, 135.8, 129.7, 118.8, 40.5, 34.4, 34.0, 32.1, 24.2, 12.6, 8.4 ppm.
HRMS (ESI, pos): *m/z* calculated for $C_{14}H_{18}ONa^+$ $[M+Na]^+$: 225.1250; found: 225.1267.

3.20 4-Methyl-5-((2*S*,3*S*,4*S*)-4-methyl-5-oxo-3-(3-oxobutyl)tetrahydrofuran-2-yl)cyclopent-4-ene-1,3-dione (29)

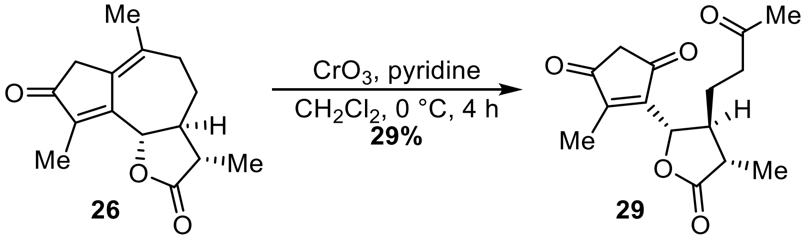

CrO_3 (81.2 mg, 812 µmol, 10.0 equiv) was added to a solution of pyridine (96.3 mg, 98.3 µL, 1.22 mmol, 15.0 equiv) in CH_2Cl_2 (2.5 mL) at 0 °C, and the mixture was stirred for 10 min. A solution of alkene **26** (20.0 mg, 81.2 µmol, 1.00 equiv) in CH_2Cl_2 (0.25 mL) was then added dropwise, and the reaction mixture was stirred at 0 °C for 4 h. The mixture was poured into saturated aqueous $NaHCO_3$ (5 mL), and the aqueous layer was extracted with CH_2Cl_2 (3 × 5 mL). The combined organic layers were dried over $MgSO_4$, filtered, and concentrated under reduced pressure. Purification by flash column chromatography (SiO_2, *c*Hex/EtOAc 1:1) afforded triketone **29** (6.50 mg, 23.5 µmol, 29% yield) as a white to pale yellow solid.

^{1}**H NMR** (700 MHz, $CDCl_3$): δ = 5.06 (d, *J* = 9.0 Hz, 1H), 2.96 (d, *J* = 5.2 Hz, 2H), 2.56 – 2.45 (m, 2H), 2.46 – 2.40 (m, 1H), 2.29 (dddd, *J* = 10.1, 9.0, 7.0, 6.1 Hz, 1H), 2.13 (s, 3H), 2.12 (s, 3H), 1.90 – 1.79 (m, 2H), 1.35 (d, *J* = 7.1 Hz, 3H) ppm.
^{13}**C{^{1}H} NMR** (176 MHz, $CDCl_3$): δ = 206.9, 199.3, 198.5, 177.7, 158.6, 152.9, 75.5, 47.8, 41.4, 41.2, 40.3, 30.1, 25.4, 15.1, 9.7 ppm.
HRMS (ESI, pos): *m/z* calculated for $C_{15}H_{18}O_5Na^+$ $[M+Na]^+$: 301.1046, found: 301.1047.

The spectroscopic data are consistent with those reported in the literature.[9]

3.21 (3a*R*,4a*S*,6a*S*,7*S*,9a*S*)-1,4a,7-Trimethyl-5,6,6a,9a-tetrahydro-3*H*-oxireno[2',3':8,8a]azuleno[4,5-b]furan-2,8(4a*H*,7*H*)-dione (30)

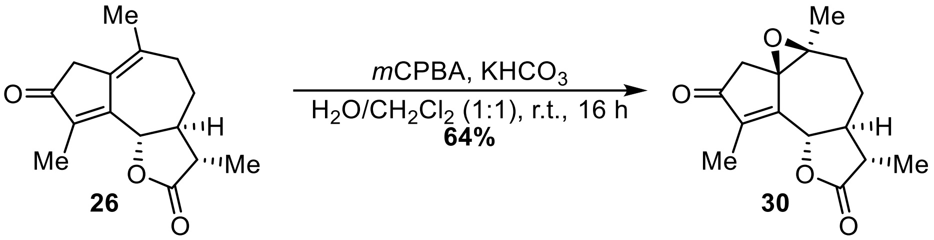

Epoxide **30** was synthesized according to a literature known procedure.[9] Alkene **26** (100 mg, 0.406 mmol, 1.00 equiv) was dissolved in CH_2Cl_2 (7 mL). *m*CPBA (70–75%, 187 mg, 0.812 mmol, 2.00 equiv) and saturated aqueous $KHCO_3$ (7 mL) were added, and the reaction mixture was stirred at room temperature for 16 h. 0.5 M aqueous NaOH (2.5 mL) was then added, the layers were separated, and the aqueous layer was extracted with CH_2Cl_2 (3 × 5 mL). The combined organic layers were washed with brine, dried over $MgSO_4$, filtered, and concentrated under reduced pressure to afford **30** (68 mg, 259 µmol, 64% yield) as a white to pale yellow solid.

^{1}**H NMR** (700 MHz, $CDCl_3$): δ = 4.84 (d, *J* = 9.3 Hz, 1H), 2.73 (d, *J* = 18.8 Hz, 1H), 2.59 (d, *J* = 18.7 Hz, 1H), 2.38 – 2.33 (m, 1H), 2.30 (dq, *J* = 11.8, 7.0 Hz, 1H), 2.03 (d, *J* = 1.5 Hz, 3H), 1.86 (dt, *J* = 12.8, 3.8 Hz, 1H), 1.68 – 1.55 (m, 3H), 1.42 (s, 3H), 1.26 (d, *J* = 7.0 Hz, 3H) ppm.
^{13}**C{^{1}H} NMR** (176 MHz, $CDCl_3$): δ = 208.3, 177.4, 165.1, 147.8, 138.1, 112.2, 81.9, 48.3, 45.2, 41.6, 40.8, 39.1, 32.0, 13.0, 9.2 ppm.
The spectroscopic data are consistent with those reported in literature.[9]

3.22 (1S,4*R*,6*S*)-1-Methyl-4-(prop-1-en-2-yl)-7-oxabicyclo[4.1.0]heptan-2-one (34a) (1*R*,4*R*,6*R*)-1-methyl-4-(prop-1-en-2-yl)-7-oxabicyclo[4.1.0]heptan-2-one (34b)

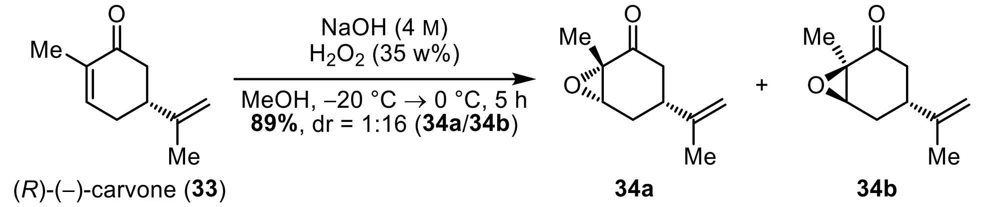

Epoxides **34a** and **34b** were synthesized according to a modified literature procedure:[11] A solution of (*R*)-(−)-carvone (**33**, 3.00 g, 20.0 mmol, 1.00 equiv) in MeOH (33 mL) was cooled to −20 °C, and 4 M aqueous NaOH (1.50 mL, 6.00 mmol, 0.30 equiv) was added. Hydrogen peroxide (35 wt % in H_2O, 2.91 mL, 30.0 mmol, 1.50 equiv) was then added dropwise, and the reaction mixture was stirred at −20 °C for 3 h before being allowed to warm to 0 °C over 2 h. Upon complete consumption of the starting material (TLC), saturated aqueous $Na_2S_2O_3$ (10 mL) was added slowly. MeOH was removed under reduced pressure, and the resulting aqueous phase was extracted with EtOAc (5 × 10 mL). The combined organic layers were dried over $MgSO_4$, filtered, and concentrated under reduced pressure. The residue was dissolved in CH_2Cl_2 (20 mL) and washed with brine (2 × 20 mL). The organic layer was dried over $MgSO_4$, filtered, and concentrated under reduced pressure to afford **34a** and **34b** (2.96 g, 17.8 mmol, 89% yield, dr = 1:16 (**34a**:**34b**)) as a yellow oil.

NMR data of major isomer 34b

^{1}H NMR (400 MHz, $CDCl_3$): δ = 4.78 (s, 1H), 4.71 (s, 1H), 3.44 (dd, *J* = 3.0, 0.9 Hz, 1H), 2.71 (tt, *J* = 11.1, 4.3 Hz, 1H), 2.58 (ddd, *J* = 17.7, 4.6, 1.3 Hz, 1H), 2.36 (dt, *J* = 14.7, 2.9 Hz, 1H), 2.02 (dd, *J* = 17.7, 11.6 Hz, 1H), 1.89 (ddd, *J* = 14.8, 11.1, 1.1 Hz, 1H), 1.70 (s, 3H), 1.40 (s, 3H) ppm.

^{13}C{^{1}H} NMR (151 MHz, $CDCl_3$) δ = 205.8, 146.6, 110.8, 61.6, 59.1, 42.1, 35.3, 29.0, 20.9, 15.6 ppm.

The spectroscopic data are consistent with those reported in the literature.[11]

3.23 (*R*)-3-Hydroxy-2-methyl-5-(prop-1-en-2-yl)cyclohex-2-en-1-one (35)

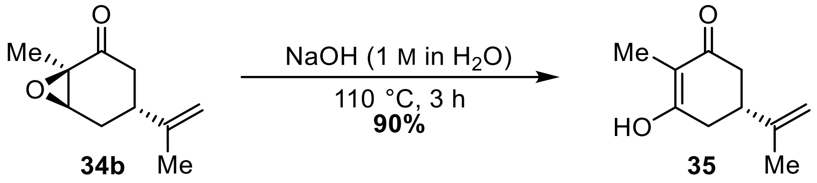

Diketone **35** was synthesized according to a modified literature procedure:[12] Epoxide **34b** (4.84 g, 29.1 mmol, 1.00 equiv) was suspended in 1 M aqueous NaOH (73 mL, 2.50 equiv) and heated at reflux for 3 h. The reaction mixture was cooled to room temperature and acidified to pH 2 with 4 M aqueous HCl. The aqueous layer was extracted with EtOAc (5 × 40 mL), and the combined organic layers were washed with brine, dried over Na_2SO_4, filtered, and concentrated under reduced pressure to afford **35** (4.32 g, 26.0 mmol, 90% yield) as a beige solid, which was used without further purification.

^{1}H NMR (600 MHz, DMSO-*d*$_6$): δ = 10.38 (s, 1H), 4.76 (d, *J* = 12.1 Hz, 2H), 2.61 (tt, *J* = 10.5, 5.1 Hz, 1H), 2.46 – 2.18 (m, 4H), 1.71 (s, 3H), 1.54 (s, 3H) ppm.
^{13}C{^{1}H} NMR (151 MHz, DMSO-*D*$_6$): δ = 147.1, 110.8, 109.6, 20.8, 7.7 ppm.
HRMS (ESI, pos): *m/z* calculated for $C_{10}H_{14}O_2Na^+$ $[M+Na]^+$: 189.0886; found: 189.0886.

The spectroscopic data are consistent with those reported in literature.[12]

3.24 2-Methyl-2-(3-oxopentyl)-5-(prop-1-en-2-yl)cyclohexane-1,3-dione (36)

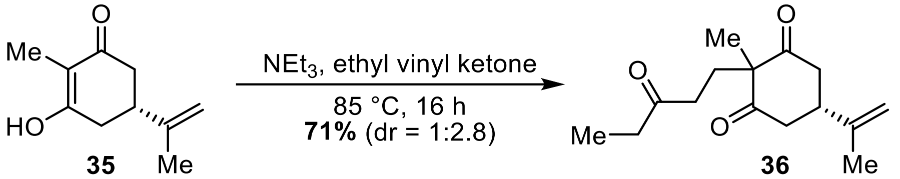

Triketone **36** was synthesized according to a modified literature procedure:[13] A one-necked flask equipped with a condenser was charged with dione **35** (4.32 g, 26.0 mmol, 1.00 equiv) and EtOAc (50 mL). NEt_3 (4.53 mL, 3.29 g, 32.5 mmol, 1.25 equiv) and ethyl vinyl ketone (2.85 mL, 2.40 g, 28.5 mmol, 1.10 equiv) were added, and the suspension was heated to 85 °C and stirred for 16 h. The reaction mixture was cooled to room temperature and concentrated under reduced pressure. The residue was purified by flash column chromatography (SiO_2, pentane/Et_2O 4:1 to 2:1 to 1:1) to afford triketone **36** (4.58 g, 18.3 mmol, 71% yield, dr = 1:2.8) as a yellow liquid.

Analytical data of major isomer:

^{1}H NMR (400 MHz, $CDCl_3$): δ = 4.85 (s, 1H), 4.75 (s, 1H), 2.85 – 2.71 (m, 4H), 2.56 – 2.46 (m, 1H), 2.39 – 2.28 (m, 4H), 2.03 (t, J = 7.8 Hz, 2H), 1.74 (s, 3H), 1.19 (s, 3H), 0.99 (t, J = 7.3 Hz, 3H).

^{13}C{^{1}H} NMR (151 MHz, $CDCl_3$): δ = 210.0, 209.5, 144.9, 111.9, 64.0, 42.8, 36.9, 36.8, 36.0, 30.6, 20.5, 18.7, 7.8 ppm.

HRMS (ESI, pos): *m/z* calculated for $C_{15}H_{22}O_3K^+$ $[M+K]^+$: 289.1200; found: 289.1197.

3.25 5,8a-Dimethyl-3-(prop-1-en-2-yl)-3,4,8,8a-tetrahydronaphthalene-1,6(2*H*,7*H*)-diones (32a and 32b)

Method A

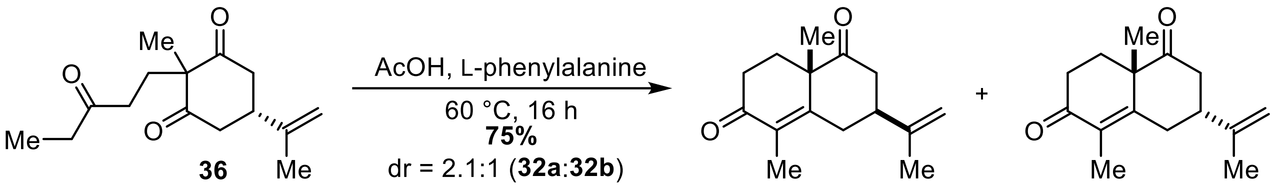

32a **32b**

Enones **32a** and **32b** were synthesized according to a modified literature procedure:[13] L-Phenylalanine (857 mg, 5.20 mmol, 1.30 equiv) was added to a solution of triketone **36** (1.00 g, 4.00 mmol, 1.00 equiv) in AcOH (8 mL). The reaction mixture was heated to 60 °C and stirred for 16 h, then cooled to room temperature and concentrated under reduced pressure. The residue was dissolved in 2 M aqueous NaOH (20 mL), and the mixture was extracted with Et_2O (4 × 8 mL). The combined organic layers were washed with brine, dried over Na_2SO_4, filtered, and concentrated under reduced pressure. The crude product was purified by flash column chromatography (SiO_2, *c*Hex/EtOAc 9:1 to 4:1) to afford **32a** and **32b** (695 mg, 2.99 mmol, 75% yield, dr = 2.1:1 (**32a**:**32b**)) as a yellow oil.

NMR data of major isomer 32a

^{1}H NMR (400 MHz, $CDCl_3$): δ = 4.87 (s, 1H), 4.84 (s, 1H), 3.01 – 2.95 (m, 1H), 2.71 – 2.63 (m, 1H), 2.59 – 2.34 (m, 5H), 2.18 – 2.01 (m, 2H), 1.84 (s, 3H), 1.80 (s, 3H), 1.43 (s, 3H) ppm.

^{13}C{^{1}H} NMR (151 MHz, $CDCl_3$): δ = 211.5, 197.9, 157.2, 146.3, 131.1, 111.0, 50.6, 42.6, 41.9, 33.6, 32.8, 29.4, 23.7, 20.3, 11.7 ppm

Analytical data of minor isomer 32b

^{1}H NMR (700 MHz, $CDCl_3$): δ = 4.80 (s, 1H), 4.63 (s, 1H), 2.89 – 2.83 (m, 2H), 2.79 – 2.73 (m, 2H), 2.51 – 2.47 (m, 3H), 2.17 – 2.13 (m, 1H), 1.99 – 1.92 (m, 1H), 1.80 (d, *J* = 1.4 Hz, 3H), 1.75 (s, 3H), 1.41 (s, 3H) ppm.

^{13}C{^{1}H} NMR (176 MHz, $CDCl_3$): δ = 212.6, 197.5, 157.2, 146.2, 132.2, 111.7, 49.7, 42.2, 38.5, 33.3, 32.2, 30.1, 23.8, 21.4, 11.6 ppm.

HRMS (ESI, pos): *m/z* calculated for $C_{15}H_{20}O_2K^+$ $[M+K]^+$: 271.1095; found: 271.1101.

The spectroscopic data are consistent with those reported in the literature.[14]

Method B

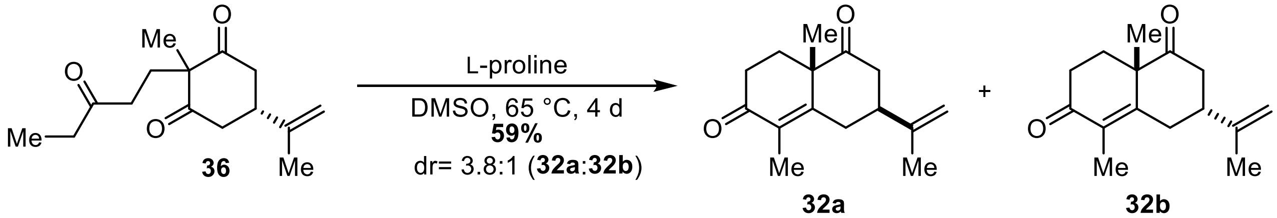

Enones **32a** and **32b** were synthesized according to a modified literature procedure:[15] A solution of triketone **36** (1.50 g, 6.00 mmol, 1.00 equiv) and L-proline (69.0 mg, 600 μmol, 10.0 mol%) in DMSO (8.5 mL) was heated at 65 °C under argon and stirred for 4 days. Perchloric acid ($HClO_4$, 1 M in H_2O, 12 mL, 12.0 mmol, 2.00 equiv) was then added, and the reaction mixture was stirred at 90 °C for 1 h. After cooling to room temperature, H_2O was added, and the mixture was extracted with CH_2Cl_2. The combined organic layers were washed with brine, dried over Na_2SO_4, filtered, and concentrated under reduced pressure. The residue was purified by flash column chromatography (SiO_2, *c*Hex/EtOAc 9:1) to afford **32a** and **32b** (819 mg, 3.53 mmol, 59% yield, dr = 3.8:1 (**32a**:**32b**)) as a yellow oil.

3.26 3-(3-Hydroxy-2,6-dimethylbenzyl)-4-methylpent-4-enoic acid (38)

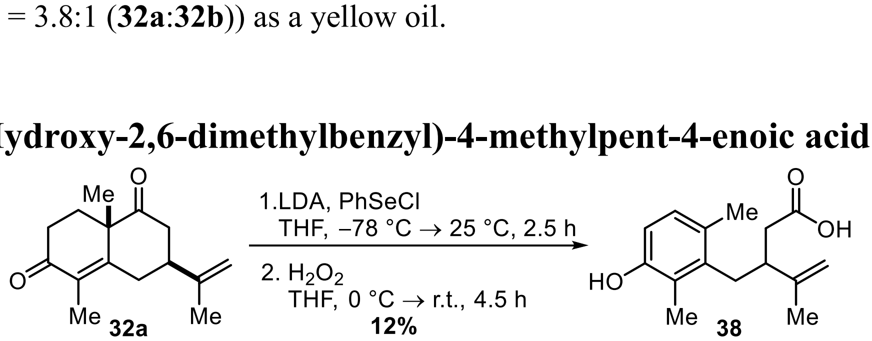

Phenol **38** was synthesized according to a modified literature procedure:[5] A dry 100 mL Schlenk flask equipped with a Teflon-coated magnetic stirring bar was charged with a solution of enone **32a** (200 mg, 860 μmol, 1.00 equiv) in dry THF (20 mL) under argon and cooled to −78 °C. LDA (0.171 M in THF, 14 mL, 1.20 mmol, 1.40 equiv) [prepared from *n*BuLi (2.50 M, 480 μL, 1.20 mmol, 1.40 equiv) and diisopropylamine (170 μL, 1.20 mmol, 1.40 equiv) in dry

THF (7 mL) at −78 °C] was added dropwise to the stirring solution, and the mixture was stirred for 30 min. A solution of phenylselenyl chloride (181 mg, 947 μmol, 1.10 equiv) in dry THF (7 mL) was then added dropwise at −78 °C. The reaction mixture was stirred for 2 h while warming to room temperature. The mixture was poured into 1 M aqueous HCl (35 mL), and the aqueous layer was extracted with Et_2O (3 × 20 mL). The combined organic layers were washed with H_2O (30 mL) and brine (2 × 30 mL), dried over $MgSO_4$, filtered, and concentrated under reduced pressure to give a green oil. The crude product was purified by flash column chromatography (SiO_2, *c*Hex/EtOAc 9:1 followed by EtOAc) to afford the corresponding selenide (97.0 mg), which was used directly in the next step without further purification.

A 1 L round-bottom flask equipped with a Teflon-coated magnetic stirring bar was charged with a solution of the selenide (97.0 mg, 250 μmol, 1.00 equiv) in THF (7 mL) and cooled to 0 °C. Hydrogen peroxide (15% in H_2O, 567 μL, 2.50 mmol, 10.0 equiv) was added dropwise over 10 min to the stirring solution. After 1 h, the mixture was warmed to 10 °C, and after an additional 2 h, it was warmed to room temperature. The solution was poured into saturated aqueous $NaHCO_3$ (20 mL), and the aqueous layer was extracted with Et_2O (3 × 20 mL). The combined organic layers were dried over $MgSO_4$, filtered, and concentrated under reduced pressure. The resulting yellow oil was dissolved in Et_2O (10 mL) and washed with saturated aqueous Na_2CO_3 (3 × 10 mL) and H_2O (2 × 10 mL). The aqueous layers were back-extracted with CH_2Cl_2 (3 × 10 mL), and the combined organic layers were dried over $MgSO_4$, filtered, and concentrated under reduced pressure. Purification by flash column chromatography (SiO_2, *c*Hex/EtOAc 19:1) afforded phenol **38** (26.8 mg, 108 μmol, 12% yield) as a light orange oil.

^{1}H NMR (600 MHz, $CDCl_3$): δ = 6.86 (d, *J* = 8.1 Hz, 1H), 6.55 (d, *J* = 8.1 Hz, 1H), 4.82 – 4.78 (m, 2H), 2.85 – 2.77 (m, 2H), 2.70 (dd, *J* = 13.5, 9.5 Hz, 1H), 2.48 (dd, *J* = 15.3, 10.2 Hz, 1H), 2.32 (dd, *J* = 15.2, 4.7 Hz, 1H), 2.25 (s, 3H), 2.21 (s, 3H), 1.78 (s, 3H) ppm.

^{13}C{^{1}H} NMR (151 MHz, $CDCl_3$): δ = 177.9, 152.2, 147.1, 138.0, 129.1, 128.4, 123.1, 113.0, 111.6, 43.3, 37.1, 34.4, 20.4, 20.3, 12.4 ppm.

HRMS (ESI, pos): *m/z* calculated for $C_{15}H_{20}O_3Na^+$ $[M+Na]^+$: 271.1305; found: 271.1307.

3.27 3-Isopropyl-5,8a-dimethyl-8,8a-dihydro-2*H*-spiro[naphthalene-1,2'-[1,3]dioxolan]-6(7*H*)-one (39)

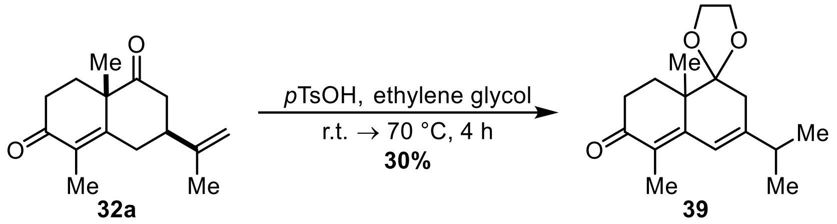

Ketone **32a** (186 mg, 0.801 mmol, 1.00 equiv) was dissolved in ethylene glycol (2.8 mL), and the resulting solution was added to ethylene glycol (0.31 mL) and *p*TsOH (152 mg, 0.801 mmol, 1.00 equiv) at room temperature. The reaction mixture was stirred for 1 h, then heated to 70 °C and stirred for an additional 2.5 h, after which saturated aqueous $NaHCO_3$ (5 mL) was added. The aqueous layer was extracted with EtOAc (2 × 5 mL), and the combined organic layers were washed with saturated aqueous $NaHCO_3$ (10 mL) and H_2O (10 mL), dried over $MgSO_4$, filtered, and concentrated under reduced pressure. The residue was purified by flash column chromatography (SiO_2, *c*Hex/EtOAc 9:1) to afford acetal **39** (66.3 mg, 0.240 mmol, 30% yield) as a yellow oil.

^{1}H NMR (700 MHz, $CDCl_3$): δ = 6.36 (d, *J* = 2.5 Hz, 1H), 4.08 – 4.01 (m, 2H), 3.97 – 3.87 (m, 2H), 2.57 – 2.48 (m, 3H), 2.43 (hept, *J* = 6.7 Hz, 1H), 2.29 (d, *J* = 18.2 Hz, 1H), 2.15 (td, *J* = 13.1, 6.9 Hz, 1H), 1.87 (s, 3H), 1.72 (ddd, *J* = 12.8, 4.6, 2.8 Hz, 1H), 1.21 (s, 3H), 1.11 (d, *J* = 6.9 Hz, 3H), 1.10 (d, *J* = 7.0 Hz, 3H) ppm.
^{13}C NMR (176 MHz, $CDCl_3$): δ = 198.9, 154.7, 153.5, 128.9, 118.3, 111.9, 65.9, 65.6, 42.7, 36.0, 34.8, 33.4, 25.3, 21.3, 20.8, 20.4, 10.9 ppm.
HRMS (ESI, pos): *m/z* calculated for $C_{17}H_{24}O_3Na^+$ $[M+Na]^+$: 299.1618; found: 299.1633.

3.28 (3S,8aS)-5,8a-dimethyl-3-(prop-1-en-2-yl)-3,4,8,8a-tetrahydro-2H-spiro[naphthalene-1,2'-[1,3]dioxolan]-6(7H)-one (40)

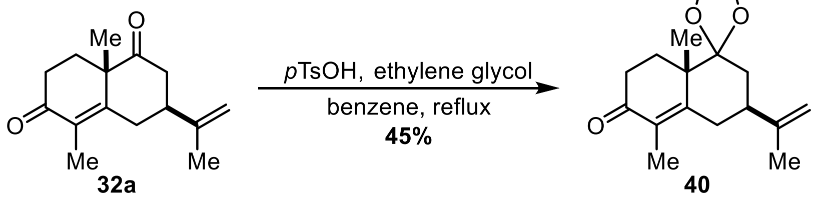

Acetal **40** was synthesized according to a literature known procedure:[14] A 25 mL round-bottom flask was charged with a solution of decalin **32a** (single diastereomer, 245 mg, 1.05 mmol, 1.00 equiv) in benzene (11 mL). Ethylene glycol (0.704 mL, 12.6 mmol, 7.33 equiv) and

pTsOH·H_2O (16.4 mg, 86.0 µmol, 5.00 mol%) were added, and the reaction mixture was heated to 100 °C using a Dean–Stark apparatus with azeotropic removal of water for approximately 20 h. The reaction was monitored by crude NMR analysis. After cooling to room temperature, the mixture was washed with saturated aqueous $NaHCO_3$ (1 × 15 mL) and H_2O (2 × 10 mL). The combined aqueous layers were extracted with CH_2Cl_2 (3 × 10 mL), and the organic layers were washed with saturated aqueous NaCl (2 × 10 mL), dried over $MgSO_4$, filtered, and concentrated under reduced pressure. The crude residue was purified by flash column chromatography (SiO_2, *c*Hex/EtOAc 9:1) to afford acetal enone **40** (131 mg, 474 µmol, 45% yield) as a yellow oil.

^{1}H NMR (700 MHz, $CDCl_3$): δ = 4.80 – 4.78 (m, 2H), 4.00 – 3.94 (m, 4H), 2.78 (ddd, J = 14.9, 4.0, 2.0 Hz, 1H), 2.48 (ddd, J = 16.5, 5.0, 3.8 Hz, 1H), 2.44 – 2.36 (m, 1H), 2.34 (tt, J = 13.0, 3.8 Hz, 1H), 2.24 (ddd, J = 14.1, 13.3, 5.0, 0.8 Hz, 1H), 2.11 – 2.06 (m, 1H), 1.87 (t, J = 13.0 Hz, 1H), 1.80 (d, J = 1.5 Hz, 3H), 1.78 (t, J = 1.1 Hz, 3H), 1.74 (ddd, J = 13.2, 3.9, 2.0 Hz, 1H), 1.67 (ddd, J = 13.2, 5.3, 3.8 Hz, 1H), 1.33 (s, 3H) ppm.
^{13}C{^{1}H} NMR (176 MHz, $CDCl_3$): δ = 198.8, 159.0, 148.1, 130.8, 113.0, 110.0, 65.6, 65.2, 44.9, 40.4, 35.0, 33.8, 32.1, 26.5, 21.1, 20.7, 11.7 ppm.
HRMS (ESI, pos): m/z calculated for $C_{17}H_{24}O_3Na^+$ $[M+Na]^+$: 299.1618; found: 299.1614.

The spectroscopic data are consistent with those reported in the literature.[14]

3.29 2-Hydroxyethyl 3-(3-hydroxy-2,6-dimethylbenzyl)-4-methylpent-4-enoate (41)

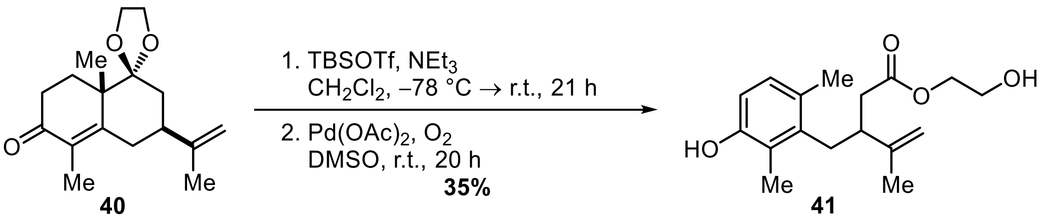

A solution of enone **40** (97.4 mg, 352 µmol, 1.00 equiv) in dry CH_2Cl_2 (6.4 mL) was cooled to −78 °C under argon, and Et_3N (89.1 mg, 122 µL, 881 µmol, 2.50 equiv) and TBSOTf (111 mg, 97.1 µL, 423 µmol, 1.20 equiv) were added successively. The reaction mixture was allowed to warm to room temperature over 2 h and stirred for an additional 16 h. The reaction was monitored by TLC (SiO_2, *c*Hex/EtOAc 2:3). Upon completion, the mixture was quenched with saturated aqueous $NaHCO_3$ (5 mL), diluted with EtOAc (50 mL), washed with H_2O (2 × 10 mL) and brine (10 mL), dried over Na_2SO_4, filtered, and concentrated under reduced pressure. The

crude residue was dissolved in DMSO (0.85 mL), $Pd(OAc)_2$ (15.8 mg, 70.0 µmol, 0.200 equiv) was added, O_2 was bubbled through the solution, and the mixture was stirred at room temperature for 7 h under an O_2 atmosphere. The reaction mixture was purified directly by flash column chromatography (SiO_2, *c*Hex/EtOAc 9:1 to 4:1) to afford **41** (36.2 mg, 124 µmol, 35% yield over two steps) as a yellow oil.

^{1}H NMR (700 MHz, $CDCl_3$): δ = 6.85 (d, *J* = 8.1 Hz, 1H), 6.57 (d, *J* = 8.1 Hz, 1H), 5.39 (s, 1H), 4.82 – 4.78 (m, 2H), 4.11 – 4.04 (m, 2H), 3.73 (t, *J* = 4.6 Hz, 2H), 2.87 (tt, *J* = 10.2, 5.3 Hz, 1H), 2.79 (dd, *J* = 14.0, 5.3 Hz, 1H), 2.72 (dd, *J* = 14.0, 10.0 Hz, 1H), 2.51 (dd, *J* = 15.1, 10.1 Hz, 1H), 2.36 (dd, *J* = 15.1, 5.3 Hz, 1H), 2.25 (s, 3H), 2.22 (s, 3H), 1.81 (s, 3H) ppm.
^{13}C{^{1}H} NMR (176 MHz, $CDCl_3$): δ = 173.2, 152.4, 147.7, 137.9, 128.9, 128.3, 123.3, 113.0, 111.3, 66.1, 61.3, 43.6, 37.5, 34.5, 20.2, 20.1, 12.5 ppm.
HRMS (ESI, pos): *m/z* calculated for $C_{17}H_{24}O_4Na^+$ $[M+Na]^+$: 315.1567; found: 315.1561.

3.30 Further Compounds Prepared during those Studies

Esters **S1** and **S2** were prepared for crystallization; however, both were obtained as oils. Attempts to introduce α,β-unsaturation into **S1** and **S2** were unsuccessful. Elimination of AcOH on **S4** failed. Robinson annulation between ethyl vinyl ketone (**7**) and **S5** or **S6** failed as well, and double-bond isomerization of **S7** to its C9=C10 isomer also remained unsuccessful.

3.30.1 (1*S*,2*S*,4a*R*)-4a,8-Dimethyl-7-oxo-2-(prop-1-en-2-yl)-1,2,3,4,4a,5,6,7-octahydronaphthalen-1-yl 4-nitrobenzoate (S1)

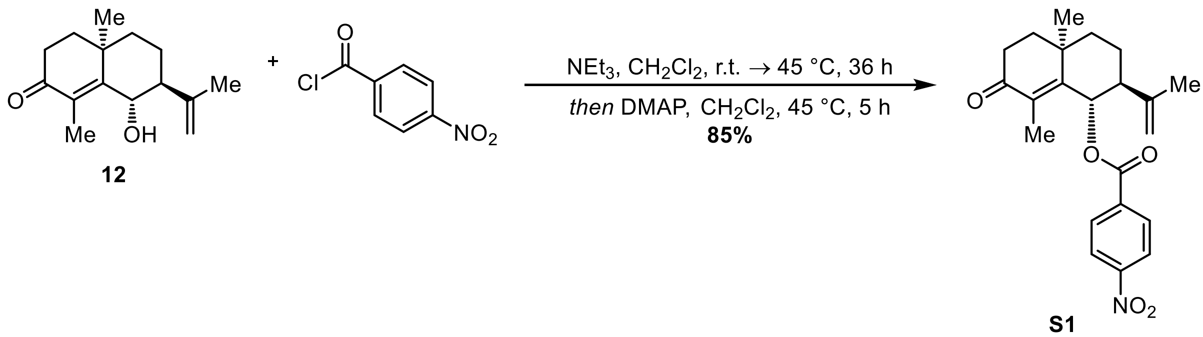

12 **S1**

4-Nitrobenzoyl chloride (42.8 mg, 0.230 mmol, 1.50 equiv) and then NEt_3 (23.3 mg, 32.1 µL, 0.230 mmol, 1.50 equiv) were added to a solution of alcohol **12** (36.0 mg, 0.154 mmol, 1.00 equiv) in CH_2Cl_2 (1.08 mL). The reaction mixture was stirred at 23 °C for 18 h. A second portion of 4-nitrobenzoyl chloride (42.8 mg, 0.230 mmol, 1.50 equiv) and NEt_3 (23.3 mg, 32.1 µL, 0.230 mmol, 1.50 equiv) was then added, and the mixture was heated to 45 °C and stirred for 18 h. DMAP (2.00 mg, 15.0 µmol, 10 mol%) was added, and stirring was continued

at 45 °C for 5 h. The solvent was removed under reduced pressure, and the residue was purified by flash column chromatography (SiO_2, cHex/EtOAc 9:1) to afford **S1** (50.0 mg, 0.130 mmol, 85% yield) as a yellow oil.

^{1}H NMR (600 MHz, $CDCl_3$): δ = 8.30 (d, *J* = 8.5 Hz, 2H), 8.18 (d, *J* = 8.6 Hz, 2H), 6.36 (s, 1H), 4.93 (s, 1H), 4.56 (s, 1H), 2.73 – 2.57 (m, 2H), 2.52 – 2.45 (m, 1H), 2.19 (tt, *J* = 13.8, 4.0 Hz, 1H), 1.97 – 1.91 (m, 4H), 1.78 (s, 3H), 1.75 – 1.59 (m, 3H), 1.52 – 1.47 (m, 1H), 1.45 (s, 3H) ppm.
^{13}C{^{1}H} NMR (151 MHz, $CDCl_3$): δ = 199.7, 163.7, 154.6, 150.9, 144.2, 135.8, 135.1, 131.0, 124.0, 112.6, 73.5, 46.0, 39.0, 35.5, 35.2, 34.4, 25.3, 23.2, 19.6, 11.5 ppm.
HRMS (ESI, pos): *m/z* calculated for $C_{22}H_{25}NO_5Na^+$ $[M+Na]^+$: 406.1625; found: 406.1626.
IR (ATR): $\tilde{\nu}$ = 3083, 2925, 1721, 1671, 1607, 1527, 1347, 1266, 1098, 908 cm^{-1}.

3.30.2 4a,8-Dimethyl-7-oxo-2-(prop-1-en-2-yl)-1,2,3,4,4a,5,6,7-octahydronaphthalen-1-yl 3,5-dinitrobenzoate (S2)

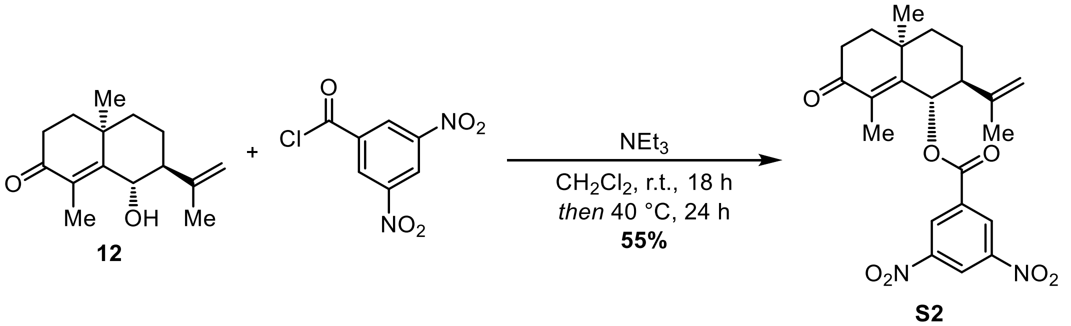

3,5-Dinitrobenzoyl chloride (17.7 mg, 77.0 µmol, 1.50 equiv) and then NEt_3 (7.80 mg, 10.7 µL, 77.0 µmol, 1.50 equiv) were added to a solution of alcohol **12** (12.0 mg, 51.2 µmol, 1.00 equiv) in CH_2Cl_2 (0.36 mL). The reaction mixture was stirred at 23 °C for 18 h, then heated to 40 °C and stirred for an additional 24 h. The solvent was removed under reduced pressure, and the residue was purified by flash column chromatography (SiO_2, cHex/EtOAc 4:1) to afford **S2** (12.0 mg, 28.0 µmol, 55% yield) as an orange oil.

^{1}H NMR (400 MHz, $CDCl_3$): δ = 9.24 (t, *J* = 2.2 Hz, 1H), 9.14 (d, *J* = 2.1 Hz, 2H), 6.43 (s, 1H), 4.96 (s, 1H), 4.57 (s, 1H), 2.73 – 2.62 (m, 2H), 2.51 (ddd, *J* = 17.9, 4.9, 2.3 Hz, 1H), 2.21 (tt, *J* = 13.6, 4.5 Hz, 1H), 2.01 – 1.91 (m, 1H), 1.95 (s, 3H), 1.81 (s, 3H), 1.79-1.70 (m, 2H), 1.65 (td, *J* = 13.5, 3.3 Hz, 1H), 1.59 – 1.53 (m, 1H), 1.49 (s, 3H) ppm.
^{13}C{^{1}H} NMR (176 MHz, $CDCl_3$): δ = 199.5, 161.7, 153.8, 149.1, 144.0, 135.6, 134.2, 129.7, 122.9, 112.9, 74.9, 46.0, 39.1, 35.5, 35.1, 34.4, 27.3, 25.3, 23.3, 19.7, 11.6 ppm.

HRMS (ESI, pos): *m/z* calculated for $C_{22}H_{24}N_2O_7K^+$ $[M+K]^+$: 467.1215; found: 467.1228.

IR (ATR): $\tilde{\nu}$ = 3101, 2924, 1729, 1671, 1544, 1344, 1270, 1158, 907 cm^{-1}.

3.30.3 (1*S*,2*S*,4a*R*)-4a,8-Dimethyl-7-oxo-2-(prop-1-en-2-yl)-1,2,3,4,4a,7-hexahydronaphthalen-1-yl 3,5-dinitrobenzoate (S3)

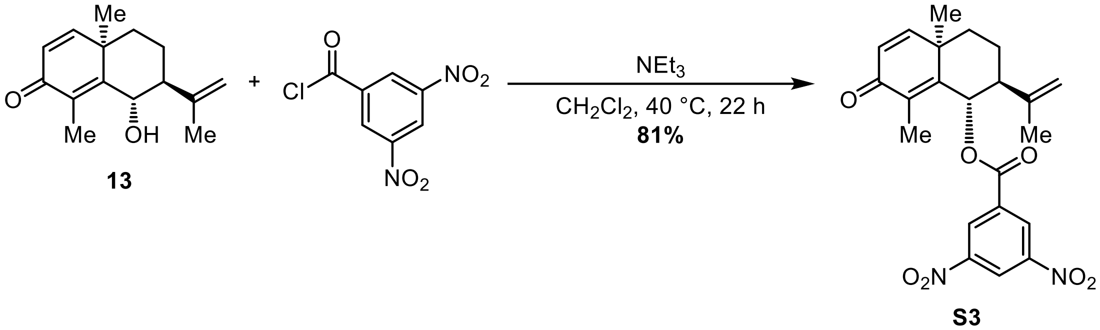

A flame dried 10 mL Schlenk tube was charged with hydroxydienone **13** (227 mg, 0.977 mmol, 1.00 equiv) in anhydrous CH_2Cl_2 (7.30 mL) under inert atmosphere and to the solution 3,5-dinitro-benzoylchloride (338 mg, 1.47 mmol, 1.50 equiv) and dry Et_3N (0.204 mL, 1.47 mmol, 1.50 equiv) were added slowly at r.t. The reaction mixture was warmed to 40 °C overnight for 22 h. After cooling to r.t., the mixture was washed with $NaHCO_3$ solution (aq., sat., 1 × 10 mL) and the aqueous phase extracted with CH_2Cl_2 (5 × 10 mL). The combined organic layers were then washed with NaCl solution (aq., sat., 1 × 10 mL), dried over $MgSO_4$, filtered and concentrated under reduced pressure. Purification by flash column chromatography (SiO_2, EtOAc/*c*Hex 10% → 20%) afforded 3,5-dinitrobenzoyl ester derivative **S3** (337 mg, 0.790 mmol, 81%) as a crystalline light-yellow solid.

^{1}H NMR (700 MHz, $CDCl_3$): δ = 9.24 (t, *J* = 2.1 Hz, 1H), 9.14 (d, *J* = 2.2 Hz, 2H), 6.77 (d, *J* = 9.9 Hz, 1H), 6.55 (dd, *J* = 2.3, 1.1 Hz, 1H), 6.25 (d, *J* = 9.9 Hz, 1H), 4.92 (s, 1H), 4.57 (s, 1H), 2.79 – 2.76 (m, 1H), 2.35 (dddd, *J* = 14.6, 13.4, 5.6, 4.0 Hz, 1H), 2.14 (s, 3H), 1.91 – 1.86 (m, 1H), 1.81 (dt, *J* = 1.4, 0.7 Hz, 3H), 1.75 (td, *J* = 13.4, 3.8 Hz, 1H), 1.69 (dtd, *J* = 13.4, 3.9, 1.0 Hz, 1H), 1.51 (s, 3H) ppm.

^{13}C NMR (176 MHz, $CDCl_3$): δ = 186.3, 161.5, 157.7, 150.0, 148.9, 143.0, 136.8, 133.9, 129.5, 125.7, 122.7, 113.1, 74.9, 45.9, 39.9, 31.6, 26.1, 23.3, 19.5, 11.8 ppm.

HRMS (ESI, pos): *m/z* calculated for $C_{22}H_{22}N_2O_7Na^+$ $[M+Na]^+$: 449.1319; found: 449.1335.

3.30.4 (4*S*,5*S*,8*S*,8a*S*)-8-Acetoxy-3,8-dimethyl-2-oxo-5-(prop-1-en-2-yl)-1,2,4,5,6,7,8,8a-octahydroazulen-4-yl 3,5-dinitrobenzoate (S4)

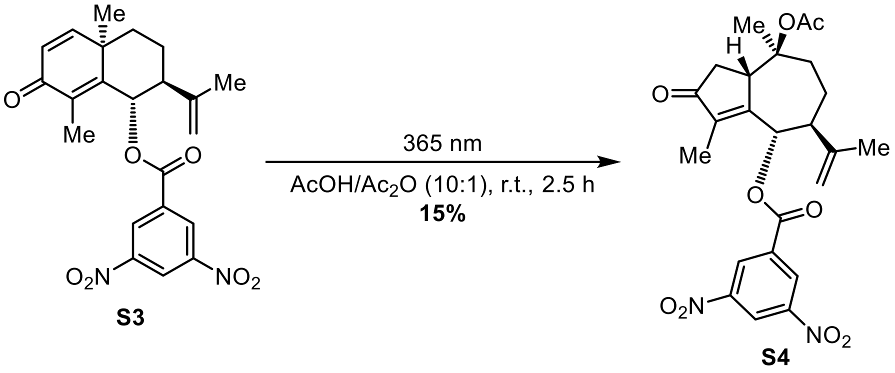

A 100 mL round-bottom flask was charged with a solution of 3,5-dinitrobenzoyl ester derivative **S3** (116 mg, 0.272 mmol, 1.00 equiv) in a mixture of AcOH (24.7 mL) and Ac_2O (2.47 mL) (0.01 M, 10:1). After degassing the reaction mixture for about 10 min under positive pressure of argon, the mixture was subjected to UV-irradiation with a LED chip (365 nm, 30 W) at r.t. for 2.5 h. The mixture changed colors from light yellow to deep orange. The solvent was evaporated under reduced pressure and the crude residue war purified by flash column chromatography (SiO_2, EtOAc/*c*Hex 10% → 20%), which afforded 3,5-dinitrobenzoyl ester guaiane enone **S4** (20.2 mg, 41.5 µmol, 15%) as an orange oil.

^{1}H NMR (600 MHz, $CDCl_3$): δ = 9.25 (t, *J* = 2.1 Hz, 1H), 9.10 (d, *J* = 2.1 Hz, 2H), 6.02 (d, *J* = 10.0 Hz, 1H), 5.01 – 4.99 (m, 1H), 4.90 (s, 1H), 3.75 – 3.71 (m, 1H), 2.78 – 2.72 (m, 1H), 2.64 (dd, *J* = 18.8, 6.8 Hz, 1H), 2.35 – 2.30 (m, 1H), 2.17 (dd, *J* = 18.8, 3.8 Hz, 1H), 2.13 (s, 3H), 1.85 – 1.80 (m, 2H), 1.76 (d, *J* = 2.1 Hz, 3H), 1.75 (s, 3H), 1.70 – 1.64 (m, 1H), 1.57 (s, 3H) ppm.
^{13}C NMR (151 MHz, $CDCl_3$): δ = 206.2, 170.1, 167.4, 161.4, 149.0, 144.3, 139.9, 133.2, 129.4, 122.9, 114.2, 84.8, 74.9, 50.0, 49.0, 38.1, 33.1, 25.4, 23.6, 22.6, 19.3, 9.5 ppm.
HRMS (ESI, pos): *m/z* calculated for $C_{24}H_{26}N_2O_9K^+$ $[M+K]^+$: 525.1270; found: 525.1296.

3.30.5 (3*R*,5*R*)-3-Hydroxy-2-methyl-5-(prop-1-en-2-yl)cyclohexan-1-one (S5)
Method A

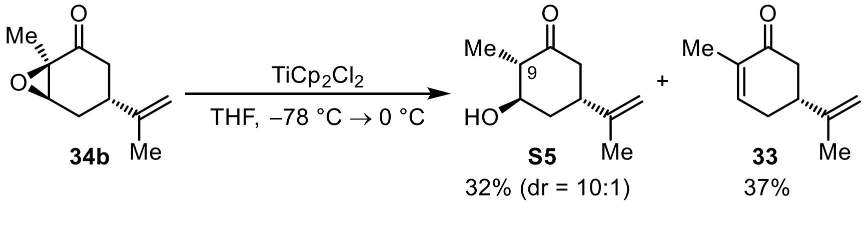

Alcohol **S5** was synthesized according to a modified literature procedure:[16] Titanocene dichloride (3.74 g, 15.0 mmol, 2.50 equiv) was added to a suspension of zinc dust (1.96 g, 30.1 mmol,

5.00 equiv) in dry THF (45 mL) at room temperature, and the mixture was stirred for 45 min. A solution of epoxide **34b** (1.00 g, 6.02 mmol, 1.00 equiv) in dry THF/MeOH (1.30 mL/0.70 mL) was then added dropwise to the resulting green mixture at −78 °C. The reaction mixture was stirred at −78 °C for 1.5 h and then warmed to 0 °C. A 10% aqueous K_2CO_3 solution (100 mL) was added at 0 °C, and the mixture was filtered through a glass frit. The layers were separated, and the aqueous layer was extracted with Et_2O (2 × 50 mL) and CH_2Cl_2 (2 × 50 mL). The combined organic layers were dried over $MgSO_4$, filtered, and concentrated under reduced pressure. Purification by flash column chromatography (SiO_2, *c*Hex/EtOAc 8:1 to 4:1) afforded alcohol **S5** (318 mg, 1.89 mmol, 32% yield) as an orange oil and (*R*)-(−)-carvone (**33**, 336 mg, 2.24 mmol, 37% yield) as a yellow oil.

NMR data of major isomer (3*R*,5*R*)-3-Hydroxy-2-methyl-5-(prop-1-en-2-yl)cyclohexan-1-one (S5)

^{1}H NMR (600 MHz, $CDCl_3$): δ = 4.79 – 4.78 (m, 1H), 4.76 (s, 1H), 4.33 – 4.30 (m, 1H), 2.87 (tt, *J* = 12.9, 3.6 Hz, 1H), 2.56 (qdd, *J* = 7.0, 3.0, 0.8 Hz, 1H), 2.49 (ddd, *J* = 13.3, 4.0, 2.3 Hz, 1H), 2.27 (td, *J* = 13.2, 1.0 Hz, 1H), 2.13 (ddd, *J* = 13.8, 5.8, 3.5 Hz, 1H), 1.83 (ddd, *J* = 13.8, 12.9, 2.2 Hz, 1H), 1.75 (s, 3H), 1.72 (s, 1H, OH), 1.11 (d, *J* = 6.8 Hz, 3H) ppm.

^{13}C{^{1}H} NMR (151 MHz, $CDCl_3$): δ = 211.2, 147.5, 110.3, 74.0, 49.6, 46.9, 40.3, 38.0, 20.9, 11.0 ppm.

The spectroscopic data are consistent with those reported in literature.[17]

Method B

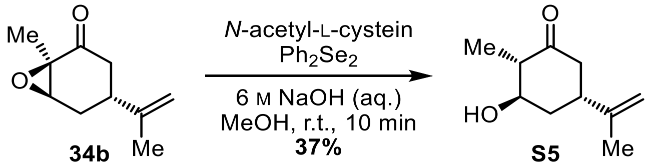

Alcohol **S5** was synthesized according to a modified literature procedure:[17] A solution of epoxide **34b** (1.00 g, 6.02 mmol, 1.00 equiv), *N*-acetyl-L-cysteine (2.94 g, 18.0 mmol, 3.00 equiv), and diphenyl diselenide (56.0 mg, 0.18 mmol, 0.03 equiv) in MeOH (15 mL) was stirred at room temperature while 6 M aqueous NaOH (3.20 mL, 19.2 mmol, 3.20 equiv) was added dropwise. The initial yellow solution became almost colorless and was stirred for 10 min. Saturated aqueous NH_4Cl (10 mL) was then added, and the resulting mixture was extracted with CH_2Cl_2 (3 × 20 mL). The combined organic layers were washed with brine (30 mL), dried over

anhydrous $MgSO_4$, filtered, and concentrated under reduced pressure to afford alcohol **S5** (377 mg, 2.24 mmol, 37% yield) as a colorless powder.

3.30.6 (2*S*,3*R*,5*R*)-2-Methyl-5-(prop-1-en-2-yl)-3-((tetrahydro-2H-pyran-2-yl)oxy)cyclohexan-1-one (S6)

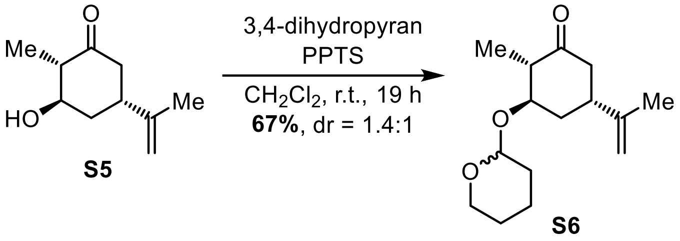

Alcohol **S5** (96.0 mg, 0.571 mmol, 1.00 equiv) and PPTS (35.9 mg, 0.143 mmol, 0.25 equiv) were dissolved in dry CH_2Cl_2 (6 mL) under argon. 3,4-Dihydropyran (96.1 mg, 0.103 mL, 1.14 mmol, 2.00 equiv) was added dropwise to the resulting solution. The yellow solution was stirred at room temperature for 20 h before it was diluted with EtOAc (10 mL), and saturated aqueous $NaHCO_3$ (10 mL) was added. The layers were separated, and the aqueous layer was extracted with EtOAc (3 × 5 mL). The combined organic layers were washed with brine (5 mL), dried over anhydrous $MgSO_4$, filtered, and concentrated under reduced pressure. Purification by flash column chromatography (SiO_2, *c*Hex/EtOAc 9:1 to 4:1) afforded **S6** (99.5 mg, 0.394 mmol, 69% yield, dr = 1.4:1) as a yellow oil.

NMR data of major isomer

^{1}H NMR (400 MHz, $CDCl_3$): δ = 4.78 (s, 2H), 4.62 (t, *J* = 3.5 Hz, 1H), 4.11 (q, *J* = 3.1 Hz, 1H), 3.83 (qd, *J* = 10.1, 9.3, 3.2 Hz, 2H), 2.90 (tt, *J* = 12.9, 3.8 Hz, 1H), 2.33 – 2.18 (m, 3H), 1.79 – 1.48 (m, 8H) 1.76 (s, 3H), 1.06 (d, *J* = 6.7 Hz, 3H) ppm.

^{13}C{^{1}H} NMR (151 MHz, $CDCl_3$): δ = 210.9, 147.6, 110.2, 101.1, 93.9, 75.1, 62.0, 49.2, 46.8, 40.1, 33.0, 31.07, 25.8, 20.9, 11.5 ppm.

Analytic data of minor isomer

^{1}H NMR (400 MHz, $CDCl_3$): δ = 4.75 (s, 2H), 4.72 (t, *J* = 3.2 Hz, 1H), 4.28 (q, *J* = 3.0 Hz, 1H), 3.55 – 3.48 (m, 2H), 2.66 (tt, *J* = 12.9, 3.8 Hz, 1H), 2.55 – 2.45 (m, 3H), 1.74 (s, 3H), 1.79 – 1.48 (m, 8H). 1.17 (d, *J* = 6.7 Hz, 3H) ppm.

^{13}C{^{1}H} NMR (151 MHz, $CDCl_3$): δ = 210.8, 147.9, 110.0, 98.8, 95.8, 81.5, 62.6, 49.7, 46.7, 40.70, 37.42, 30.8, 25.6, 19.6, 11.2 ppm.

HRMS (ESI, pos): *m/z* calculated for $C_{15}H_{24}O_3Na^+$ $[M+Na]^+$: 275.1618; found: 275.1622.

IR (ATR): $\tilde{\nu}$ = 2940, 1715, 1645, 1455, 1376, 1273, 1200, 1156, 1117, 1077, 1032, 1000, 901, 869, 815 cm^{-1}.

3.30.7 (3S,3aS,6aS,9bS)-3,9-dimethyl-6-methylene-3a,5,6,6a,7,9b-hexahydroazuleno[4,5-b]furan-2,8(3H,4H)-dione (S7)

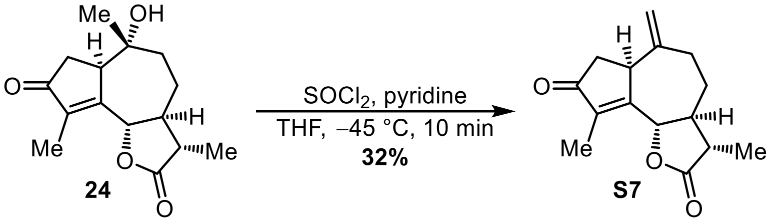

Diene **S7** was synthesized according to a literature known procedure:[18] Alcohol **24** (44.0 mg, 166 µmol, 1.00 equiv) was dissolved in THF (0.18 mL) and cooled to −45 °C. A precooled (−45 °C) solution of pyridine (0.09 mL) and $SOCl_2$ (0.09 mL) in THF (0.09 mL) was added, and the reaction mixture was stirred at −45 °C for 10 min. The mixture was then poured into a precooled mixture of H_2O and EtOAc. The layers were separated, and the aqueous layer was extracted with EtOAc (3 × 1 mL). The combined organic layers were washed with saturated aqueous $NaHCO_3$ (2 × 1 mL), H_2O, and brine, dried over $MgSO_4$, filtered, and concentrated under reduced pressure to afford diene **S7** (13.1 mg, 53.2 µmol, 32% yield) as an orange oil.

^{1}H NMR (700 MHz, $CDCl_3$): δ = 5.02 (s, 1H), 4.88 (d, J = 9.4 Hz, 1H), 4.84 (s, 1H), 3.53 (dt, J = 4.2, 2.1 Hz, 1H), 2.71 (dd, J = 19.1, 6.6 Hz, 1H), 2.62 (ddd, J = 12.6, 4.7, 2.5 Hz, 1H), 2.53 (dd, J = 19.3, 2.6 Hz, 1H), 2.35 – 2.28 (m, 2H), 2.25 – 2.20 (m, 2H), 1.89 – 1.87 (m, 3H), 1.49 – 1.41 (m, 1H), 1.29 (d, J = 6.6 Hz, 3H) ppm.
^{13}C{^{1}H} NMR (176 MHz, $CDCl_3$): δ = 203.3, 177.3, 159.5, 141.5, 82.2, 68.3, 66.4, 50.2, 41.4, 40.8, 33.6, 25.7, 24.5, 12.6, 9.3 ppm.

The spectroscopic data are consistent with those reported in the literature.[18]

4. X-ray data

4.1 X-ray data of 9

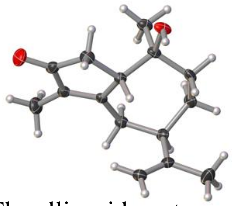

9 [X-RAY]
CCDC 2497133

Table S8. Crystal data of **9** (CCDC 2497133). The ellipsoid contour of probability level is 50%.

Identification code	compound **9** (CCDC 2497133)
Empirical formula	$C_{15}H_{22}O_2$
Formula weight	234.32
Temperature/K	150(2)
Crystal system	orthorhombic
Space group	$P2_12_12_1$
a/Å	8.7235(4)
b/Å	11.2748(6)
c/Å	13.4540(7)
α/°	90
β/°	90
γ/°	90
Volume/Å^3	1323.28(12)
Z	4
ρcalcg/cm^3	1.176
μ/mm^{-1}	0.596
F(000)	512.0
Crystal size/mm^3	0.32 × 0.16 × 0.05
Radiation	CuKα (λ = 1.54178)
2Θ range for data collection/°	10.236 to 136.658
Index ranges	-10 ≤ h ≤ 10, -13 ≤ k ≤ 13, -16 ≤ l ≤ 16
Reflections collected	9819
Independent reflections	2413 [Rint = 0.0716, Rsigma = 0.0536]
Data/restraints/parameters	2413/0/158
Goodness-of-fit on F2	1.057
Final R indexes [I>=2σ (I)]	R_1 = 0.0353, wR_2 = 0.0856
Final R indexes [all data]	R_1 = 0.0404, wR_2 = 0.0877
Largest diff. peak/hole / e Å^{-3}	0.18/-0.17
Flack parameter	0.05(16)

4.2 X-ray data of 13

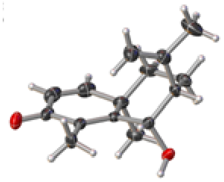
13 [X-RAY]
CCDC 2497134

Table S9. Crystal data of **13** (CCDC 2497134). The ellipsoid contour of probability level is 50%.

Identification code	compound **13** (CCDC 2497134)
Empirical formula	$C_{15}H_{20}O_2$
Formula weight	232.31
Temperature/K	150.20
Crystal system	orthorhombic
Space group	Pbca
a/Å	9.7107(2)
b/Å	11.1596(2)
c/Å	24.4558(5)
α/°	90
β/°	90
γ/°	90
Volume/Å^3	2650.21(9)
Z	8
ρcalcg/cm^3	1.164
μ/mm^{-1}	0.075
F(000)	1008.0
Crystal size/mm^3	0.61 × 0.4 × 0.29
Radiation	MoKα (λ = 0.71073)
2Θ range for data collection/°	5.356 to 52.812
Index ranges	-12 ≤ h ≤ 12, -13 ≤ k ≤ 13, -30 ≤ l ≤ 30
Reflections collected	24172
Independent reflections	2672 [Rint = 0.0442, Rsigma = 0.0210]
Data/restraints/parameters	2672/0/159
Goodness-of-fit on F2	1.084
Final R indexes [I>=2σ (I)]	R_1 = 0.0566, wR_2 = 0.1423
Final R indexes [all data]	R_1 = 0.0628, wR_2 = 0.1499
Largest diff. peak/hole / e Å^{-3}	0.45/-0.18

4.3 X-ray data of 17

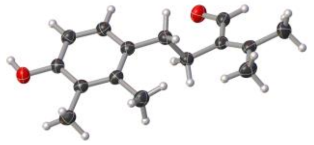

17 [X-RAY]
CCDC 2497135

Table S10. Crystal data of **17** (CCDC 2497135). The ellipsoid contour of probability level is 50%.

Identification code	compound **17** (CCDC 2497135)
Empirical formula	$C_{15}H_{20}O_2$
Formula weight	232.31
Temperature/K	150(2)
Crystal system	monoclinic
Space group	$P2_1/n$
a/Å	7.5789(3)
b/Å	14.2978(7)
c/Å	12.0674(5)
α/°	90
β/°	96.464(2)
γ/°	90
Volume/Å^3	1299.33(10)
Z	4
ρcalcg/cm^3	1.188
μ/mm^{-1}	0.077
F(000)	504.0
Crystal size/mm^3	0.33 × 0.16 × 0.14
Radiation	MoKα (λ = 0.71073)
2Θ range for data collection/°	5.698 to 52.756
Index ranges	-9 ≤ h ≤ 9, -16 ≤ k ≤ 17, -14 ≤ l ≤ 15
Reflections collected	17296
Independent reflections	2654 [Rint = 0.0417, Rsigma = 0.0239]
Data/restraints/parameters	2654/0/160
Goodness-of-fit on F2	1.061
Final R indexes [I>=2σ (I)]	R_1 = 0.0487, wR_2 = 0.1164
Final R indexes [all data]	R_1 = 0.0658, wR_2 = 0.1352
Largest diff. peak/hole / e Å^{-3}	0.20/-0.22

5. NMR and Mass Spectrometry Data

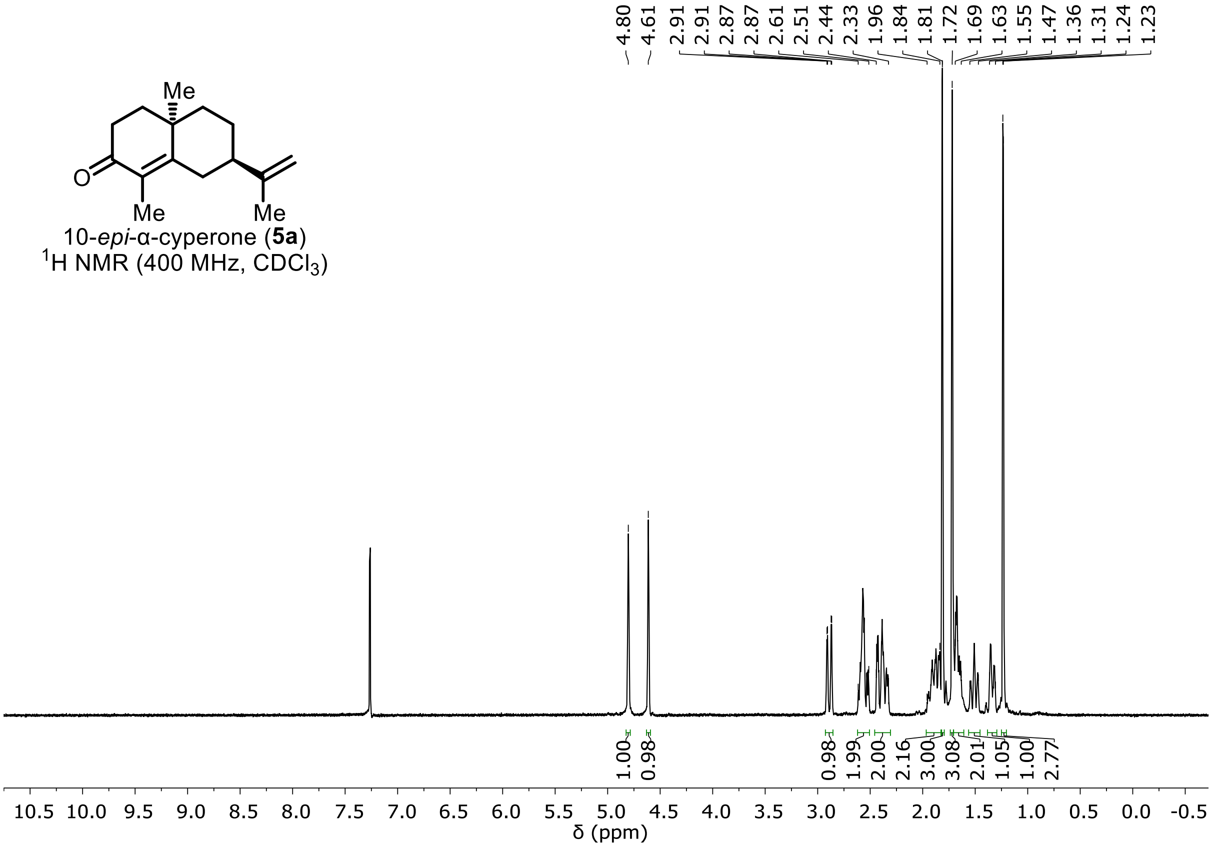

10-*epi*-α-cyperone (**5a**)
^{1}H NMR (400 MHz, $CDCl_3$)

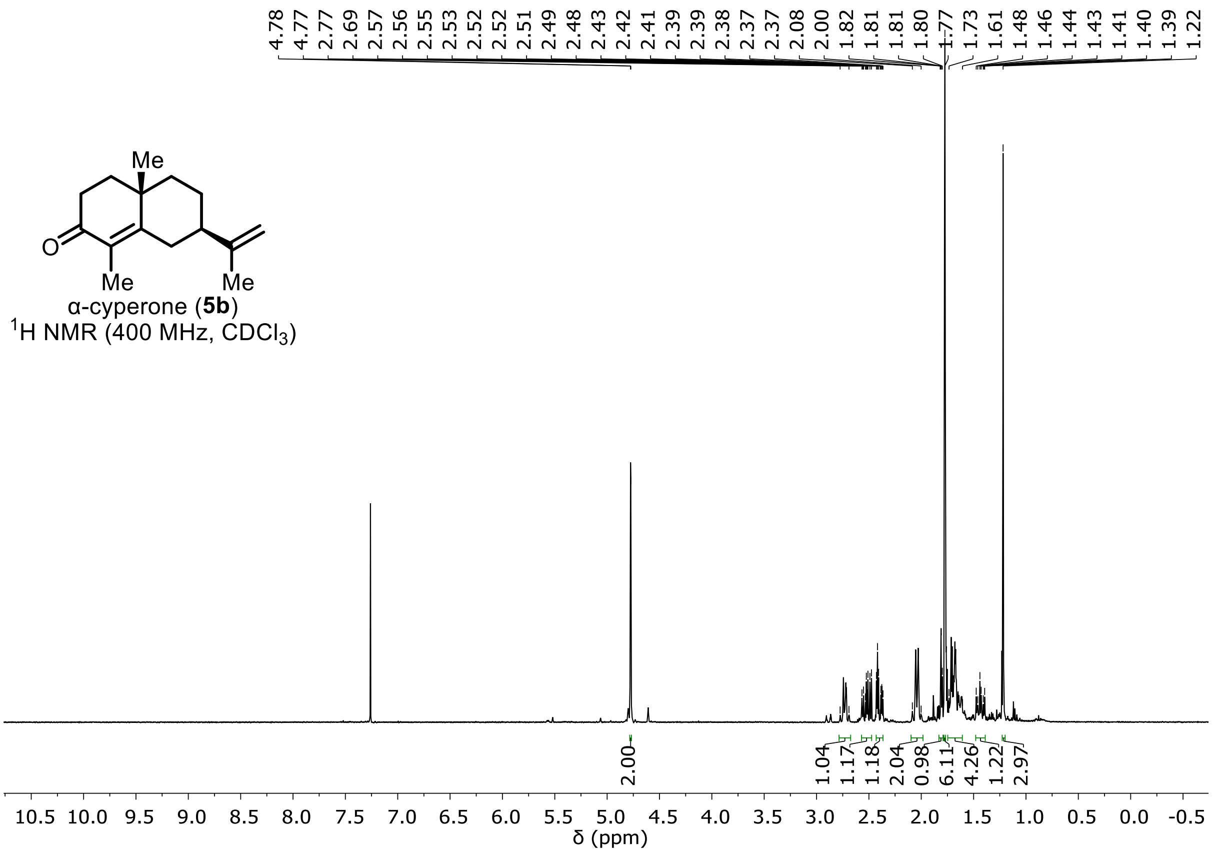

α-cyperone (**5b**)
^{1}H NMR (400 MHz, $CDCl_3$)

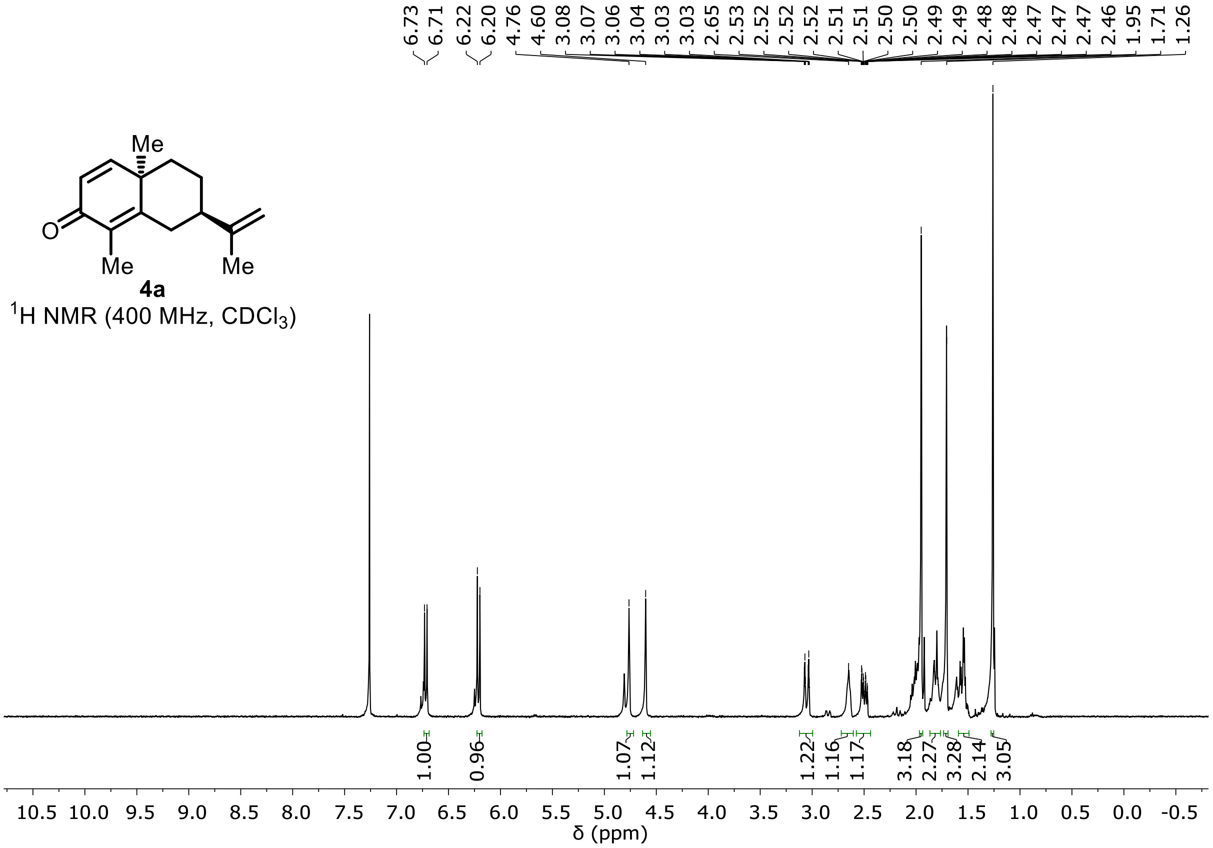
Me
O
Me
Me
4a
^{1}H NMR (400 MHz, CDCl$_3$)
6.73
6.71
6.22
6.20
4.76
4.60
3.08
3.07
3.06
3.04
3.03
3.03
2.65
2.53
2.52
2.52
2.52
2.51
2.51
2.50
2.50
2.49
2.49
2.48
2.48
2.47
2.47
2.47
2.46
1.95
1.71
1.26
1.00
0.96
1.07
1.12
1.22
1.16
1.17
3.18
2.27
3.28
2.14
3.05
10.5 10.0 9.5 9.0 8.5 8.0 7.5 7.0 6.5 6.0 5.5 5.0 4.5 4.0 3.5 3.0 2.5 2.0 1.5 1.0 0.5 0.0 -0.5
δ (ppm)

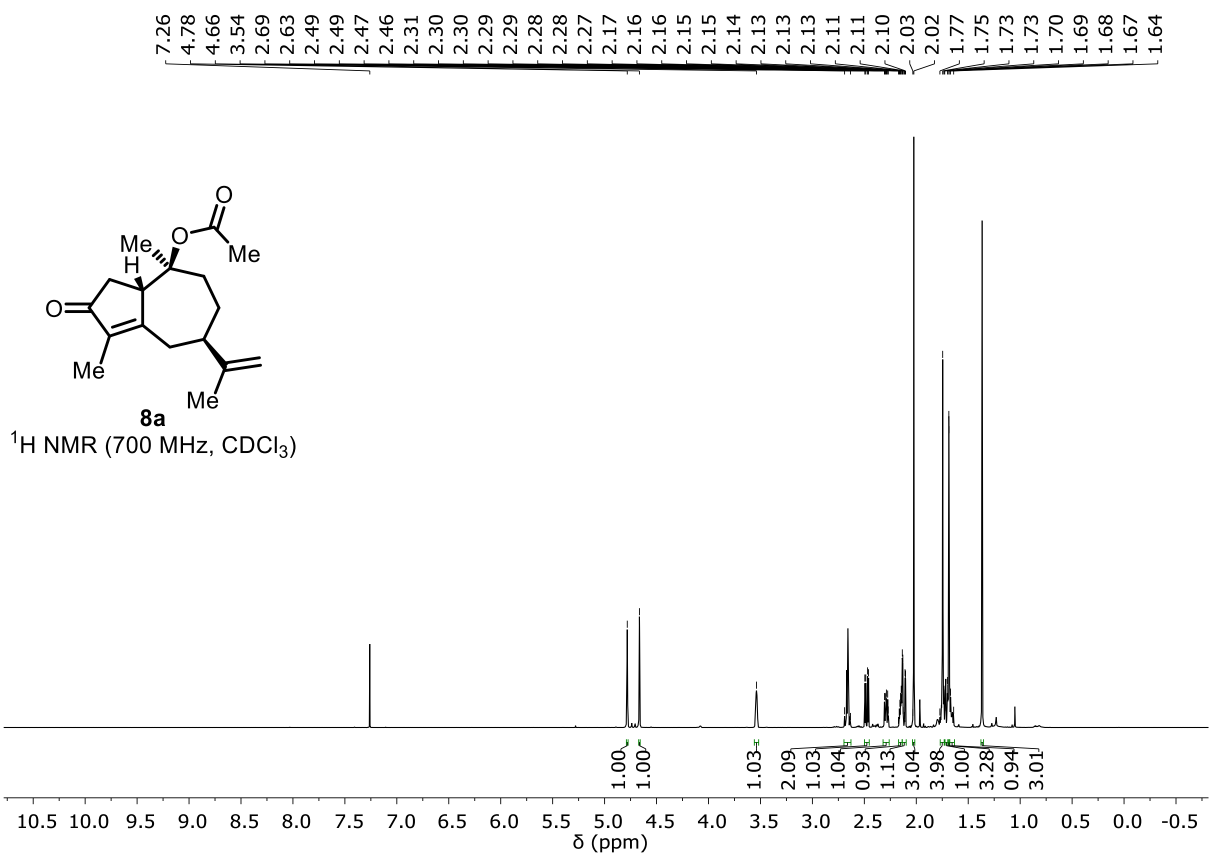
7.26
4.78
4.66
3.54
2.69
2.63
2.49
2.49
2.47
2.46
2.31
2.30
2.30
2.29
2.29
2.28
2.28
2.27
2.17
2.16
2.16
2.15
2.15
2.14
2.13
2.13
2.13
2.11
2.11
2.10
2.03
2.02
1.77
1.75
1.73
1.73
1.70
1.69
1.68
1.67
1.64
8a
^{1}H NMR (700 MHz, $CDCl_3$)
1.00
1.00
1.03
2.09
1.03
1.04
0.93
1.13
3.04
3.98
1.00
3.28
0.94
3.01
δ (ppm)

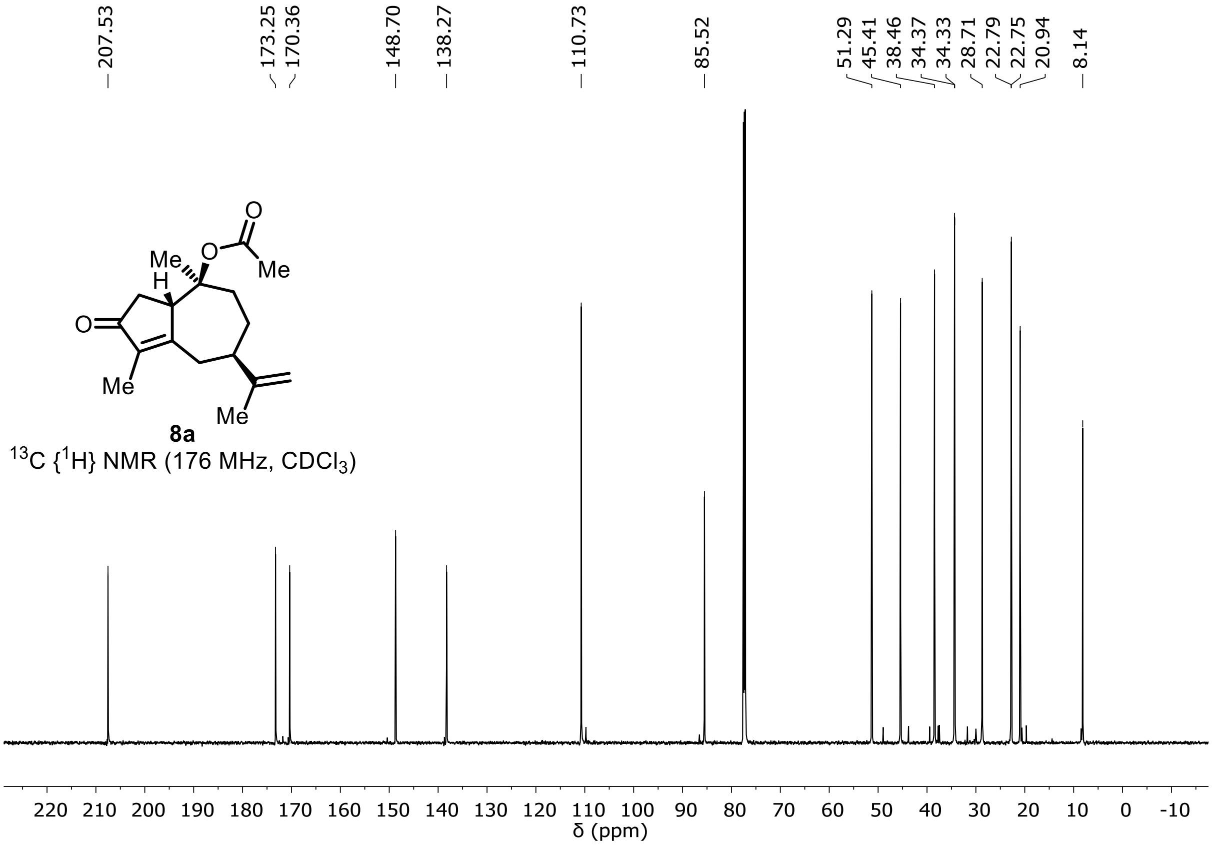
207.53
173.25
170.36
148.70
138.27
110.73
85.52
51.29
45.41
38.46
34.37
34.33
28.71
22.79
22.75
20.94
8.14
8a
^{13}C {^{1}H} NMR (176 MHz, $CDCl_3$)
δ (ppm)

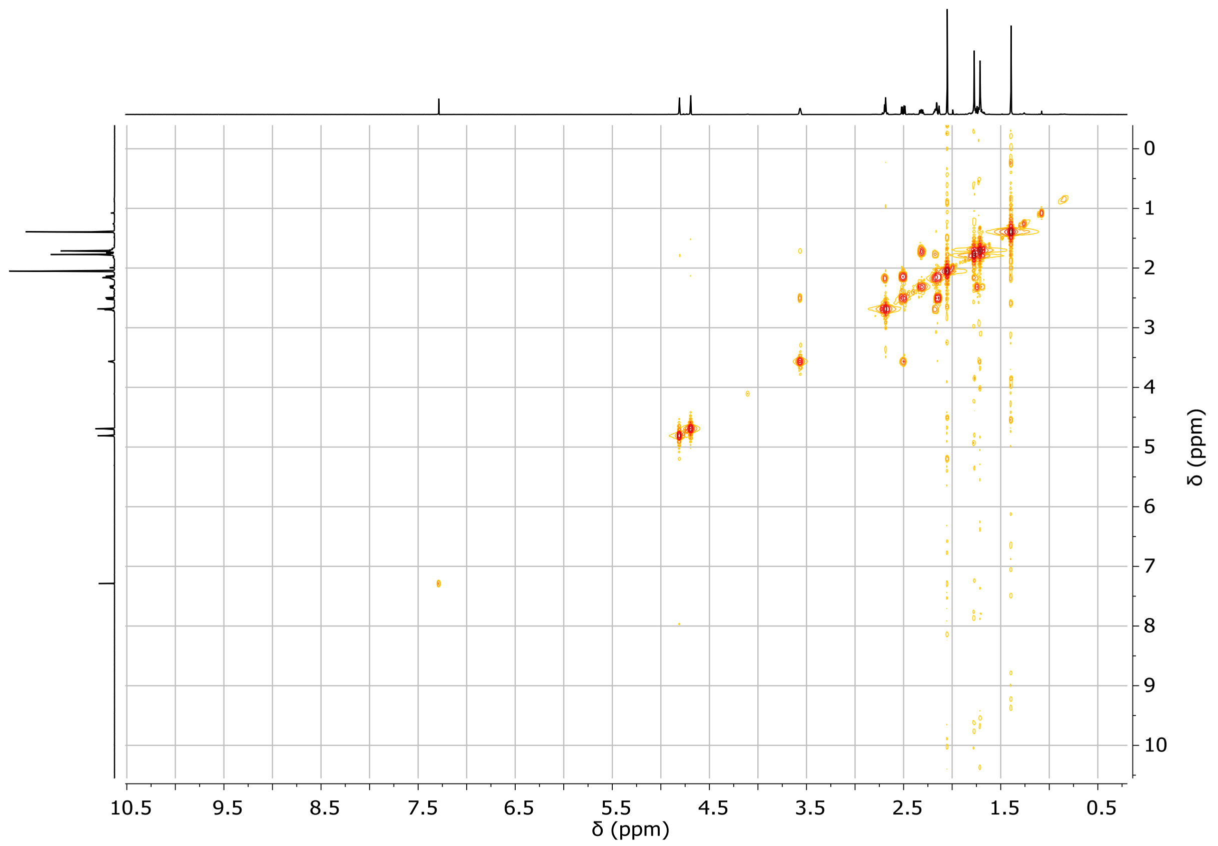

Figure S1. COSY spectra of **8a**.

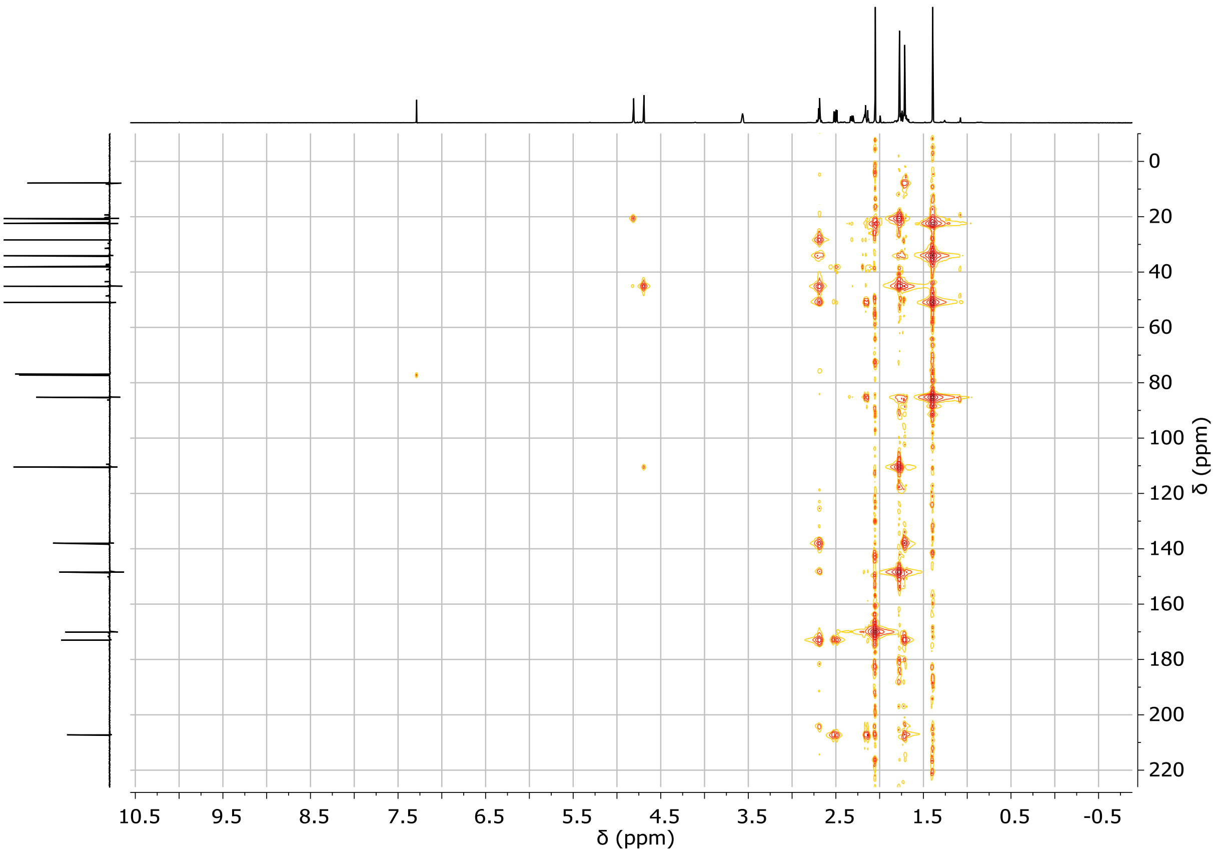

Figure S2. HMBC spectra of **8a**.

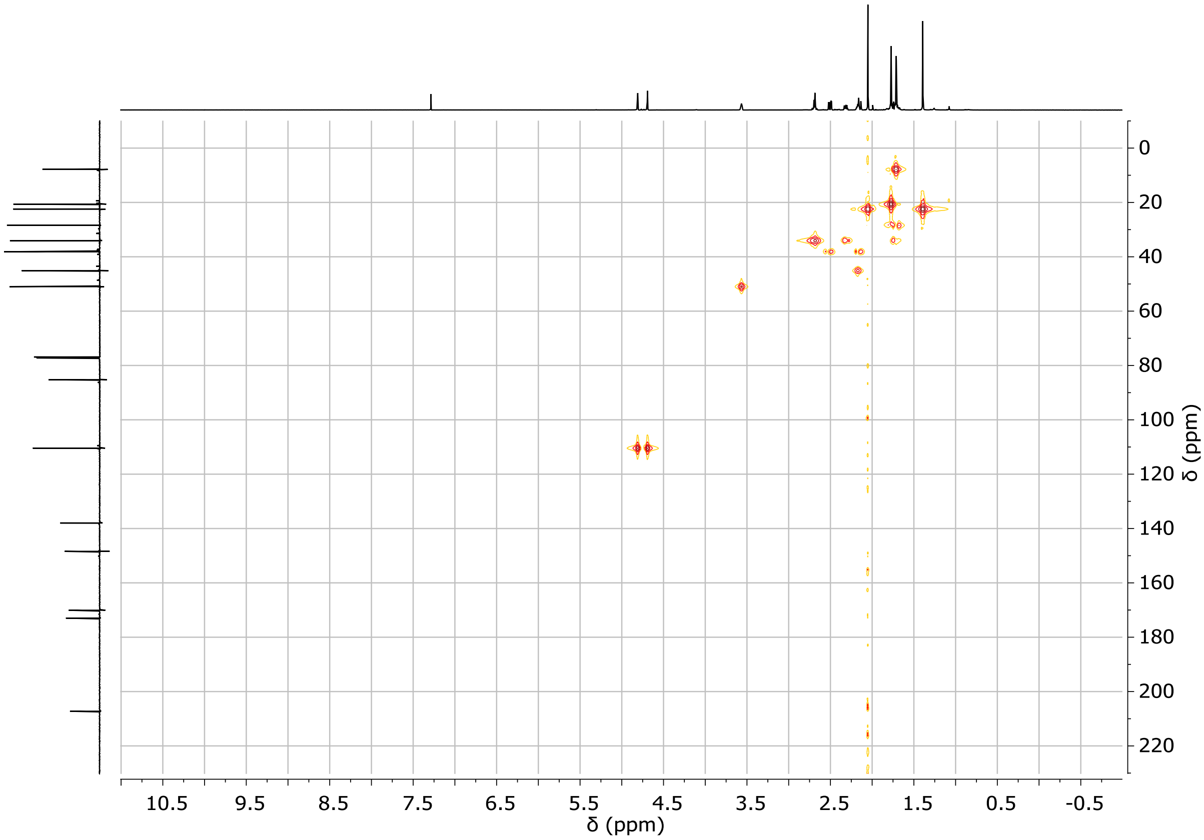

Figure S3. HMQC spectra of **8a**.

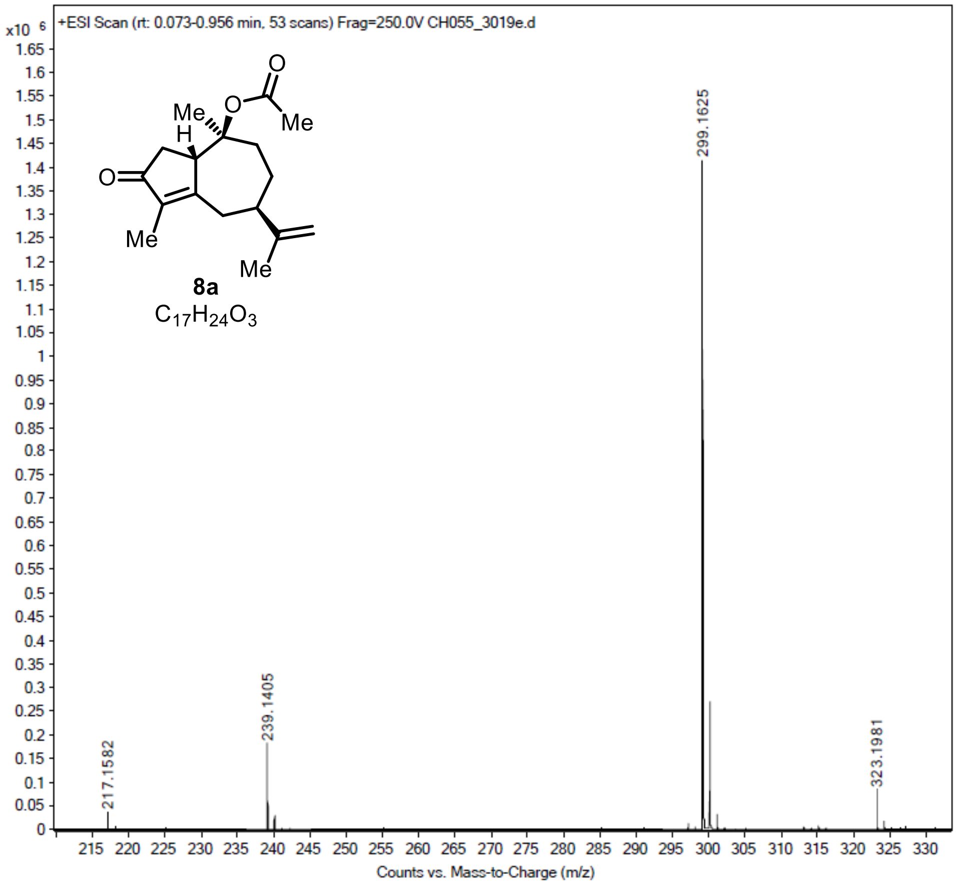

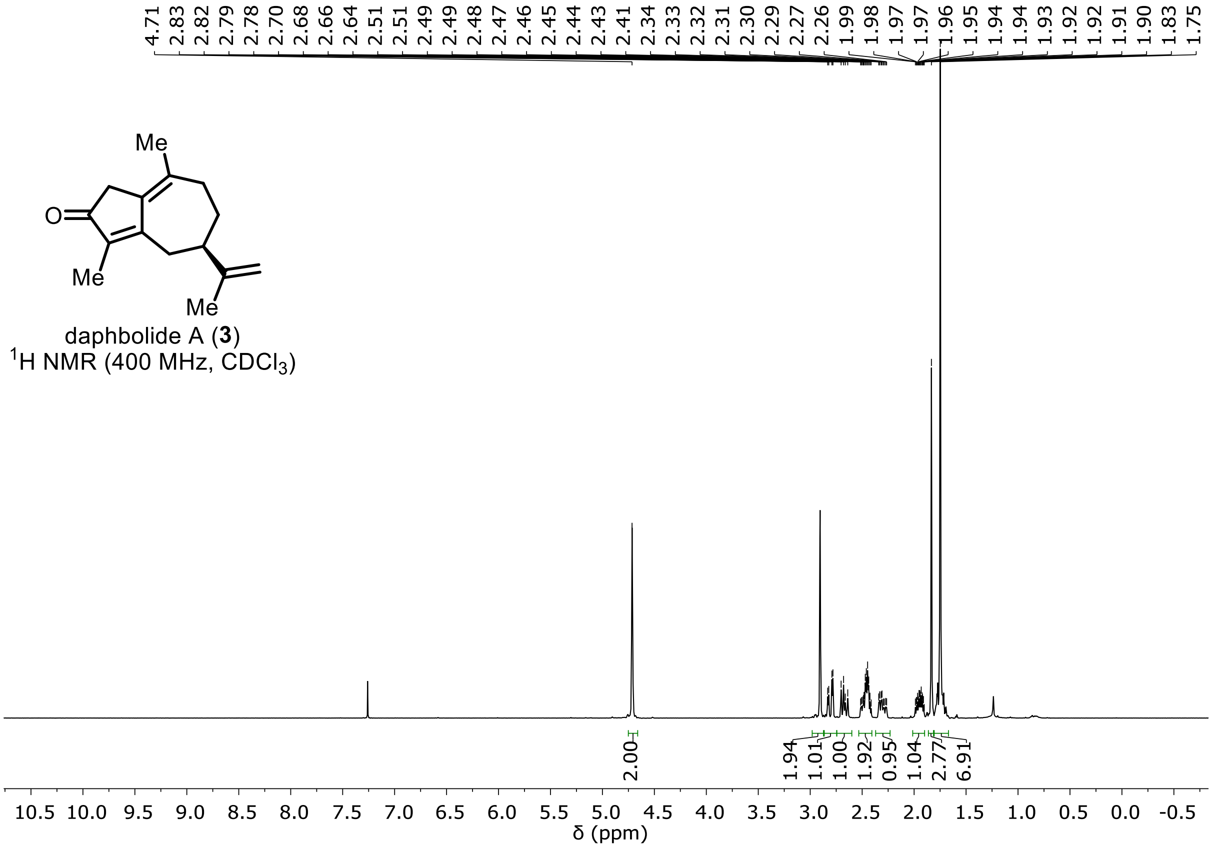

daphbolide A (**3**)
^{1}H NMR (400 MHz, $CDCl_3$)

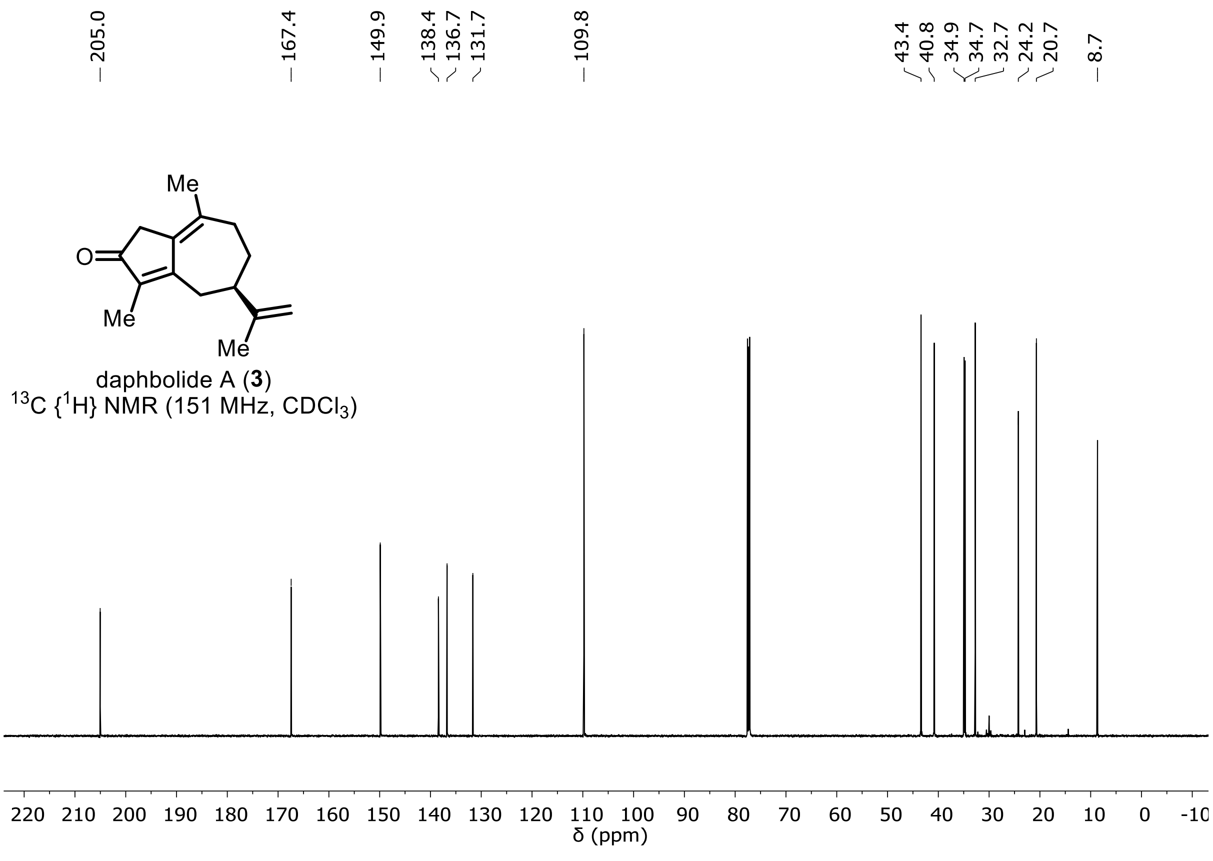

daphbolide A (**3**)
^{13}C {^{1}H} NMR (151 MHz, $CDCl_3$)

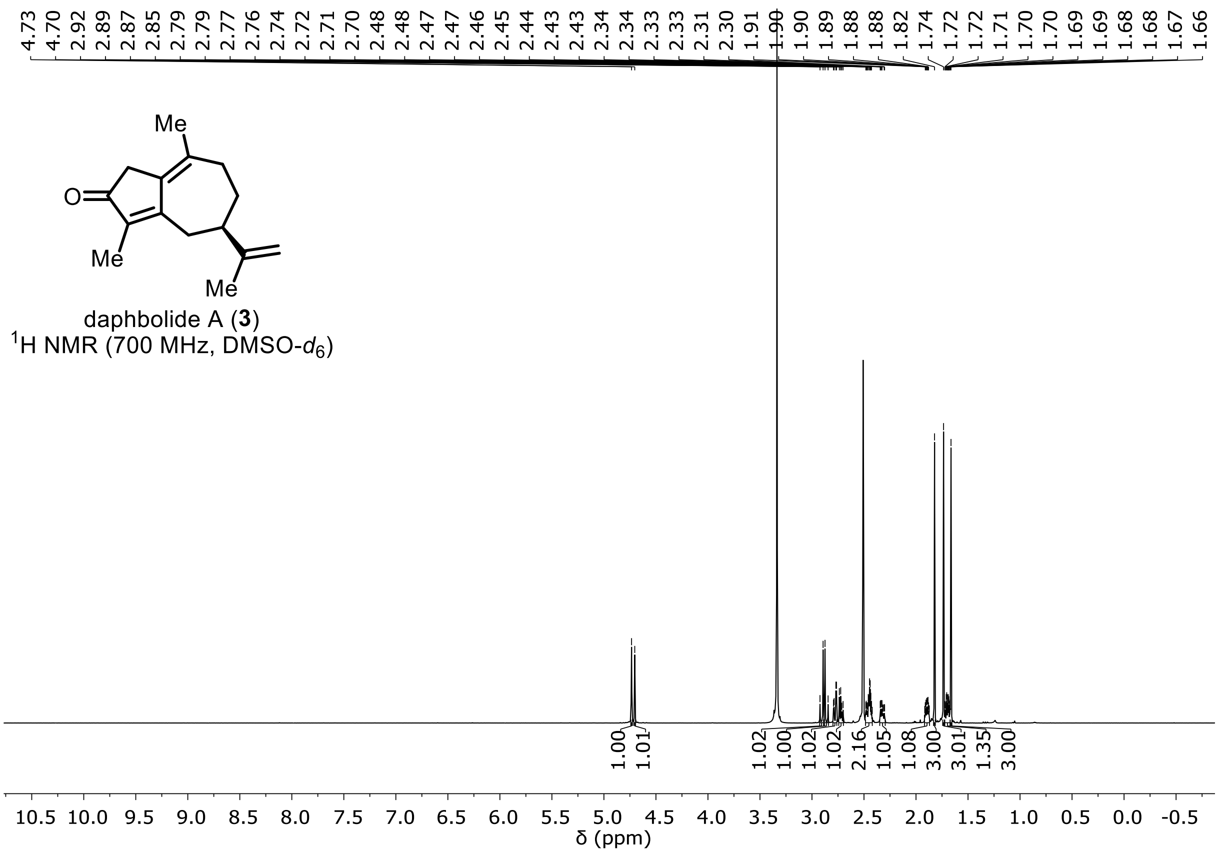
4.73
4.70
2.92
2.89
2.87
2.85
2.79
2.79
2.77
2.76
2.74
2.72
2.71
2.70
2.48
2.48
2.47
2.47
2.46
2.45
2.44
2.43
2.43
2.34
2.34
2.33
2.33
2.31
2.30
1.91
1.90
1.90
1.89
1.88
1.88
1.82
1.74
1.72
1.72
1.71
1.70
1.70
1.69
1.69
1.68
1.67
1.66
Me
O
Me
Me
daphbolide A (3)
1H NMR (700 MHz, DMSO-d6)
1.00
1.01
1.02
1.00
1.02
1.02
2.16
1.05
1.08
3.00
3.01
1.35
3.00
10.5 10.0 9.5 9.0 8.5 8.0 7.5 7.0 6.5 6.0 5.5 5.0 4.5 4.0 3.5 3.0 2.5 2.0 1.5 1.0 0.5 0.0 -0.5
δ (ppm)

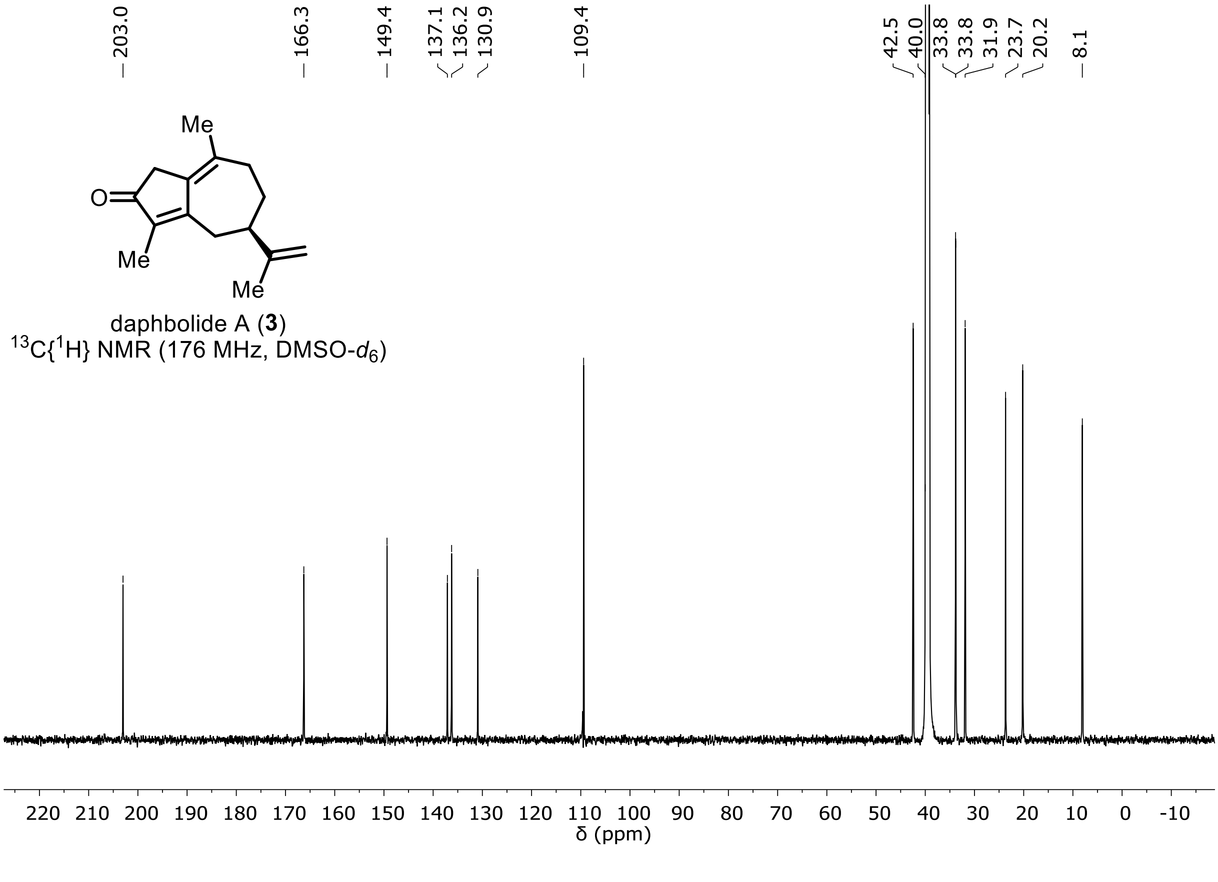
203.0
166.3
149.4
137.1
136.2
130.9
109.4
42.5
40.0
33.8
33.8
31.9
23.7
20.2
8.1
Me
O
Me
Me
daphbolide A (3)
13C{1H} NMR (176 MHz, DMSO-d6)
220 210 200 190 180 170 160 150 140 130 120 110 100 90 80 70 60 50 40 30 20 10 0 -10
δ (ppm)

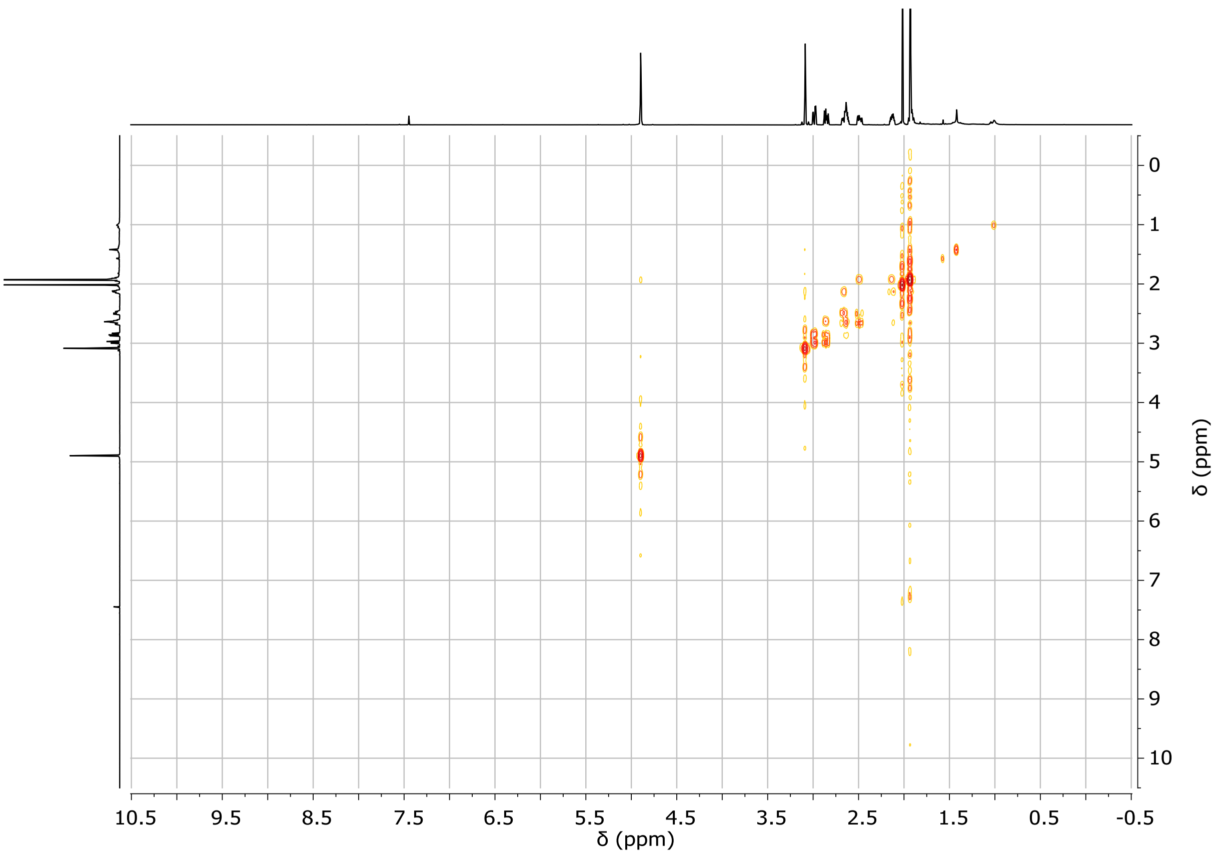

Figure S4. COSY spectra of **3**.

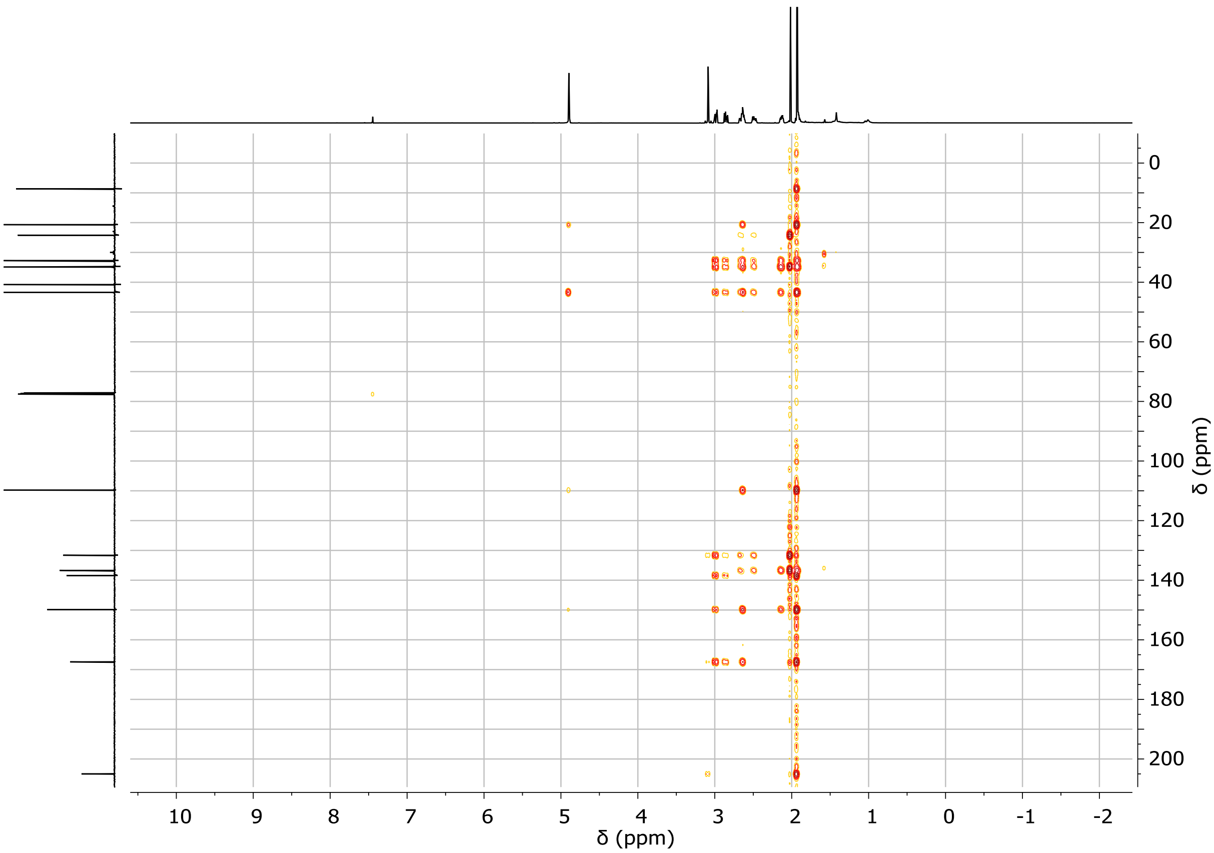

Figure S5. HMBC spectra of **3**.

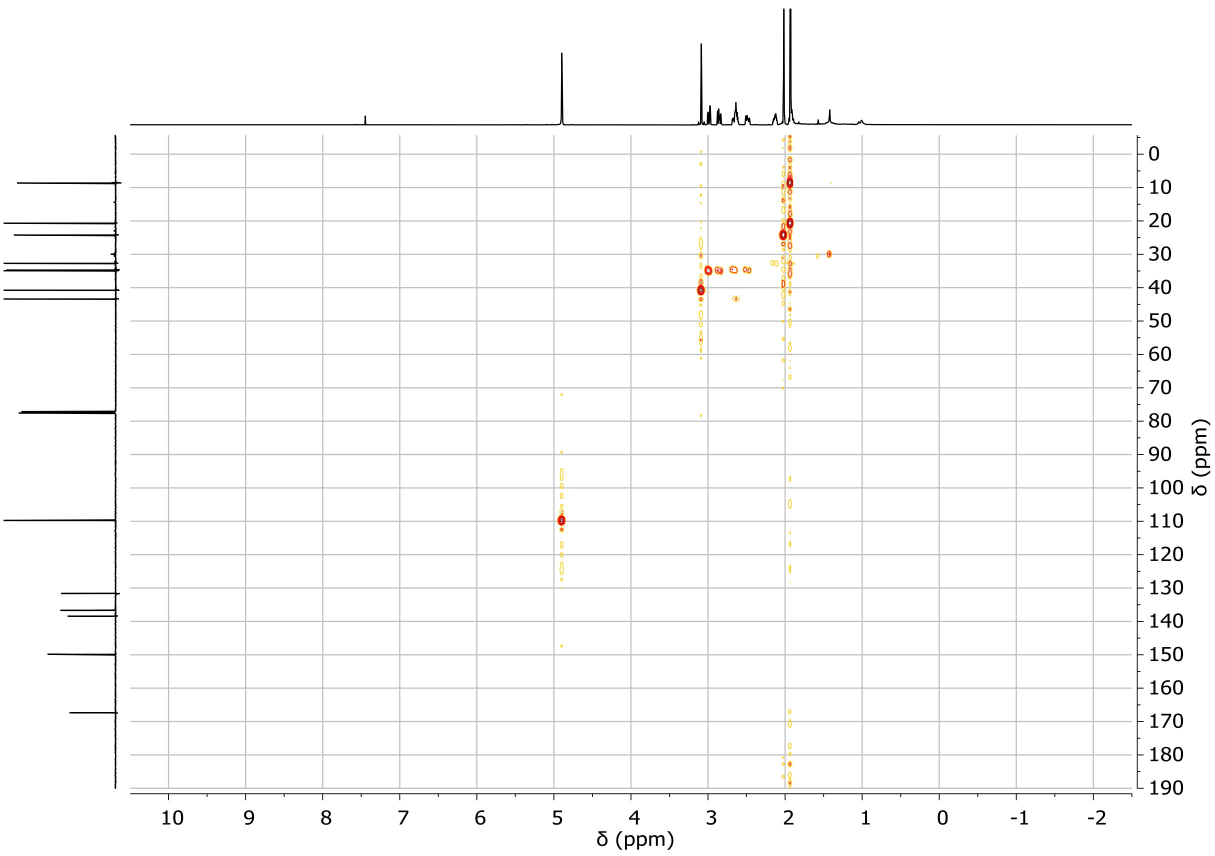

Figure S6. HMQC spectra of **3**.

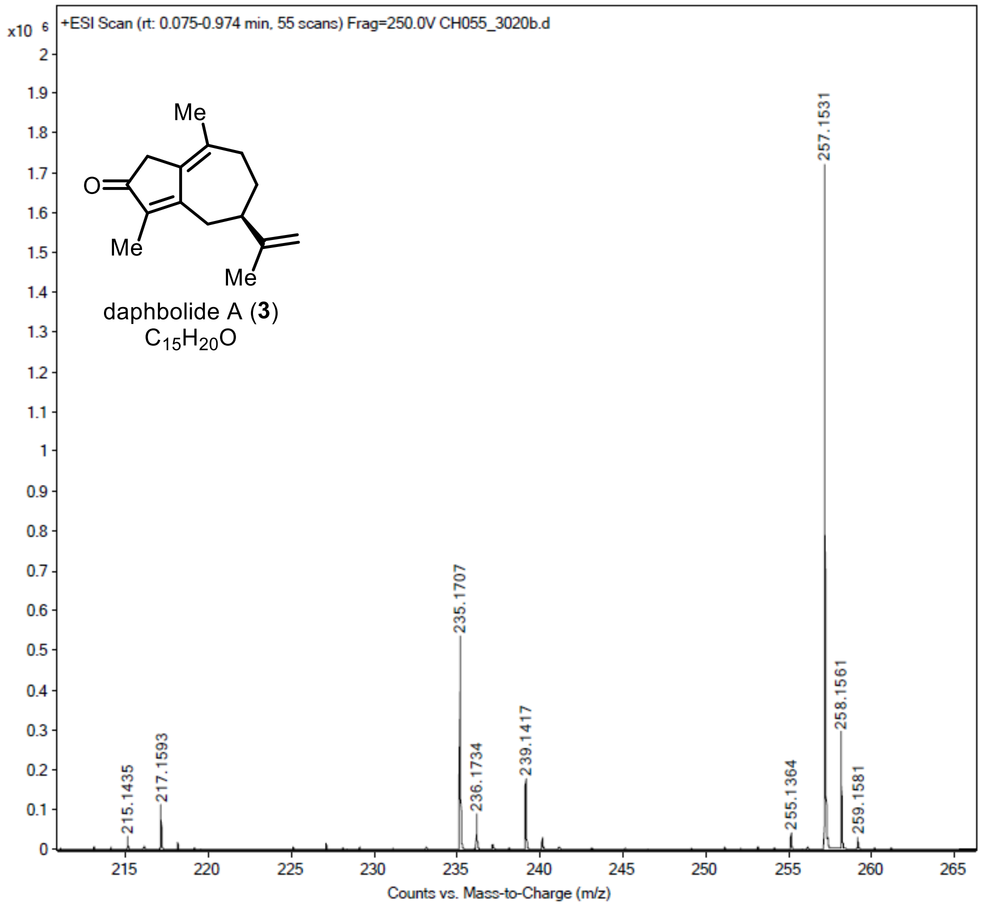

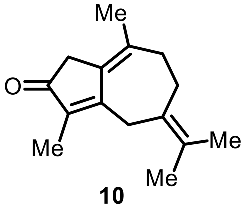

10

^{1}H NMR (600 MHz, $CDCl_3$)

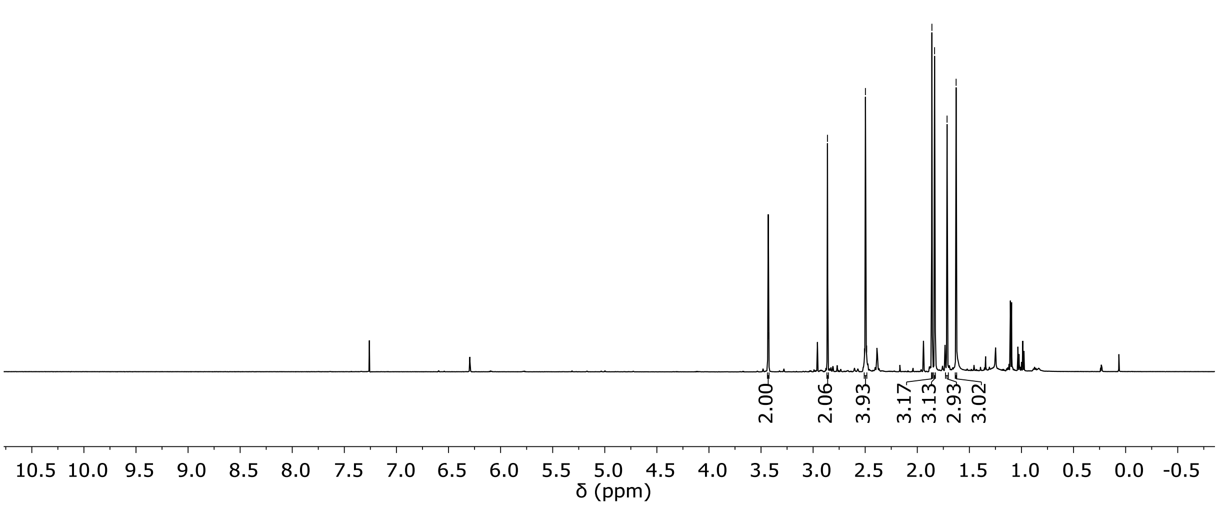

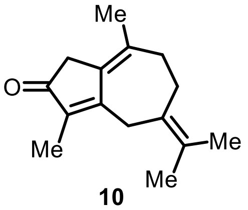

10

^{13}C {^{1}H} NMR (151 MHz, $CDCl_3$)

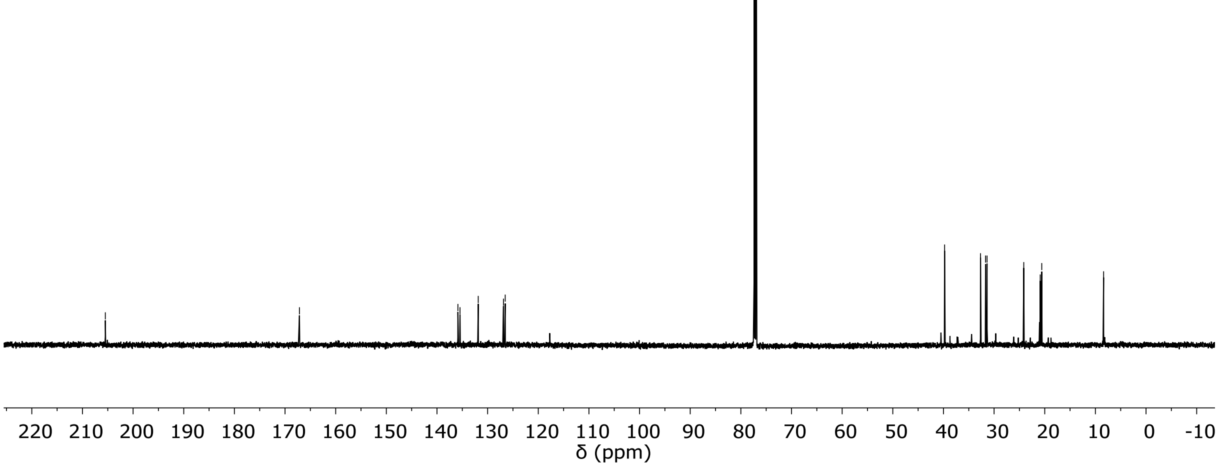

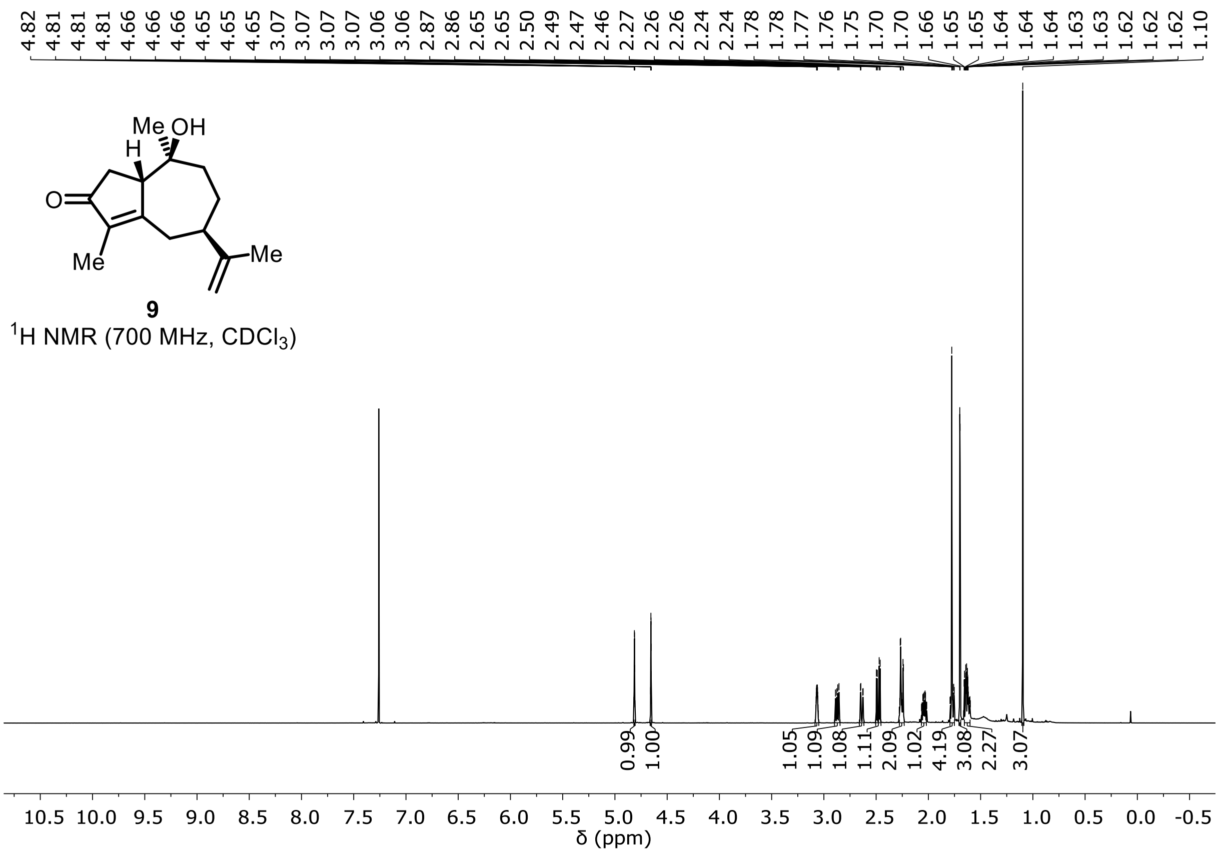

9

^{1}H NMR (700 MHz, $CDCl_3$)

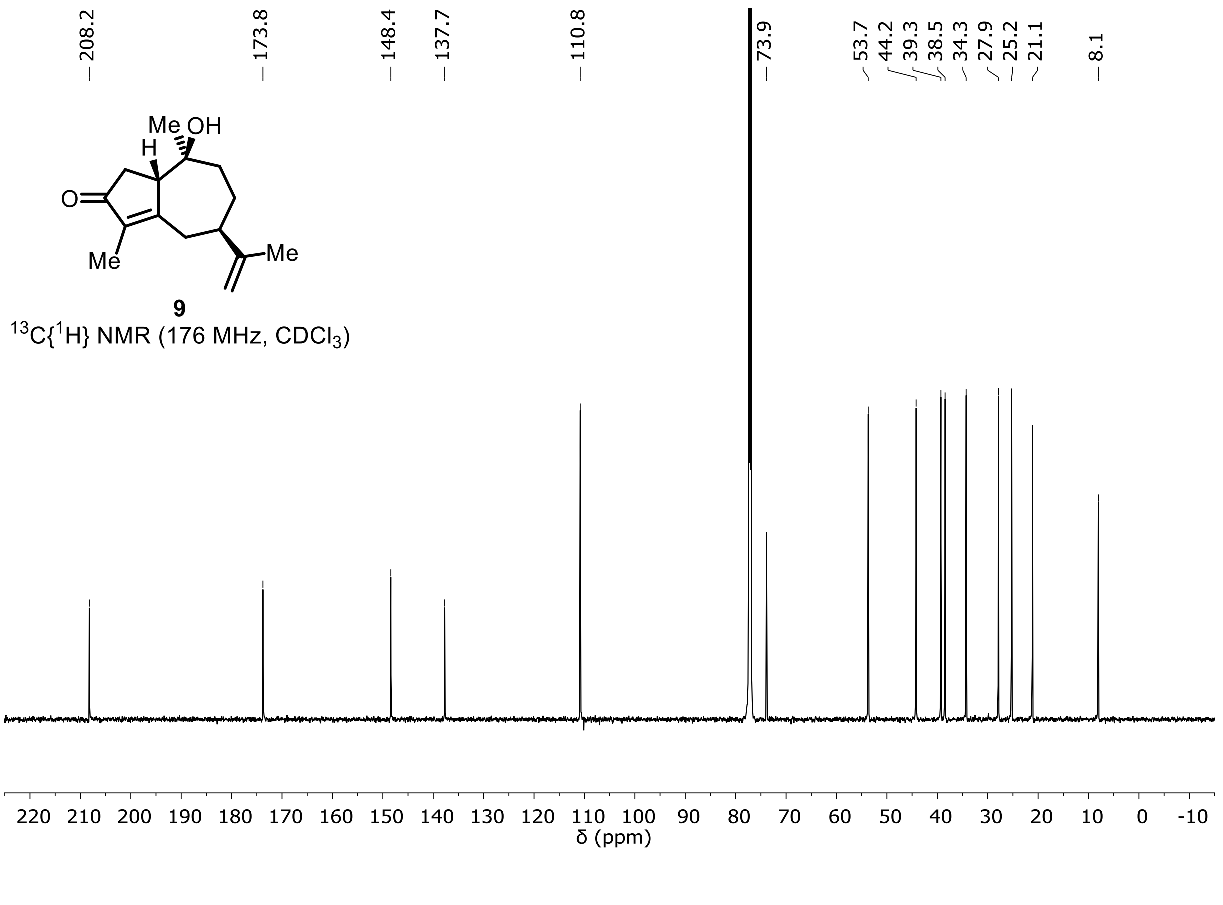

9

$^{13}C\{^{1}H\}$ NMR (176 MHz, $CDCl_3$)

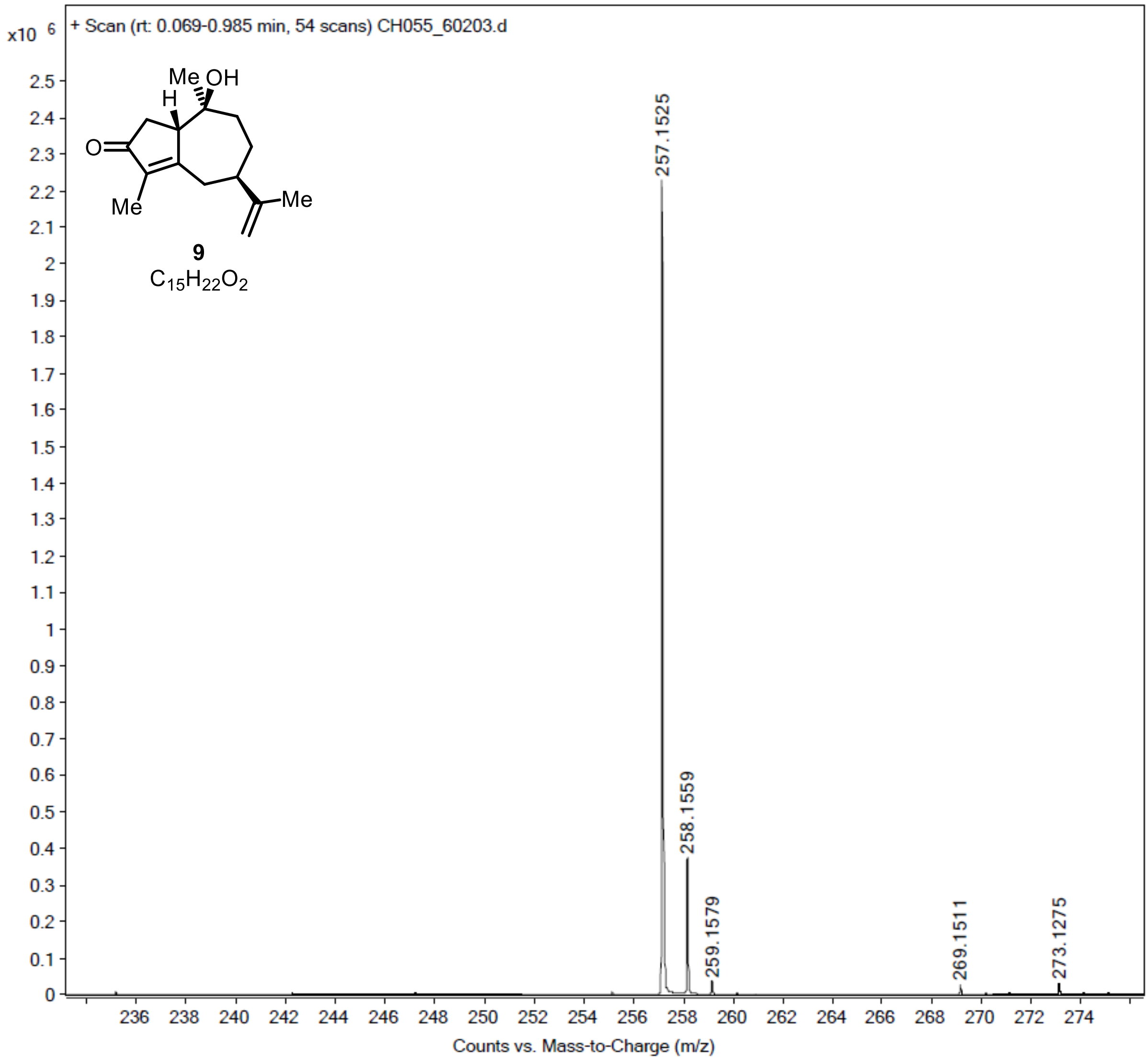
x10 6
+ Scan (rt: 0.069-0.985 min, 54 scans) CH055_60203.d
Me OH
H
O
Me
Me
9
$C_{15}H_{22}O_2$
257.1525
258.1559
259.1579
269.1511
273.1275
Counts vs. Mass-to-Charge (m/z)

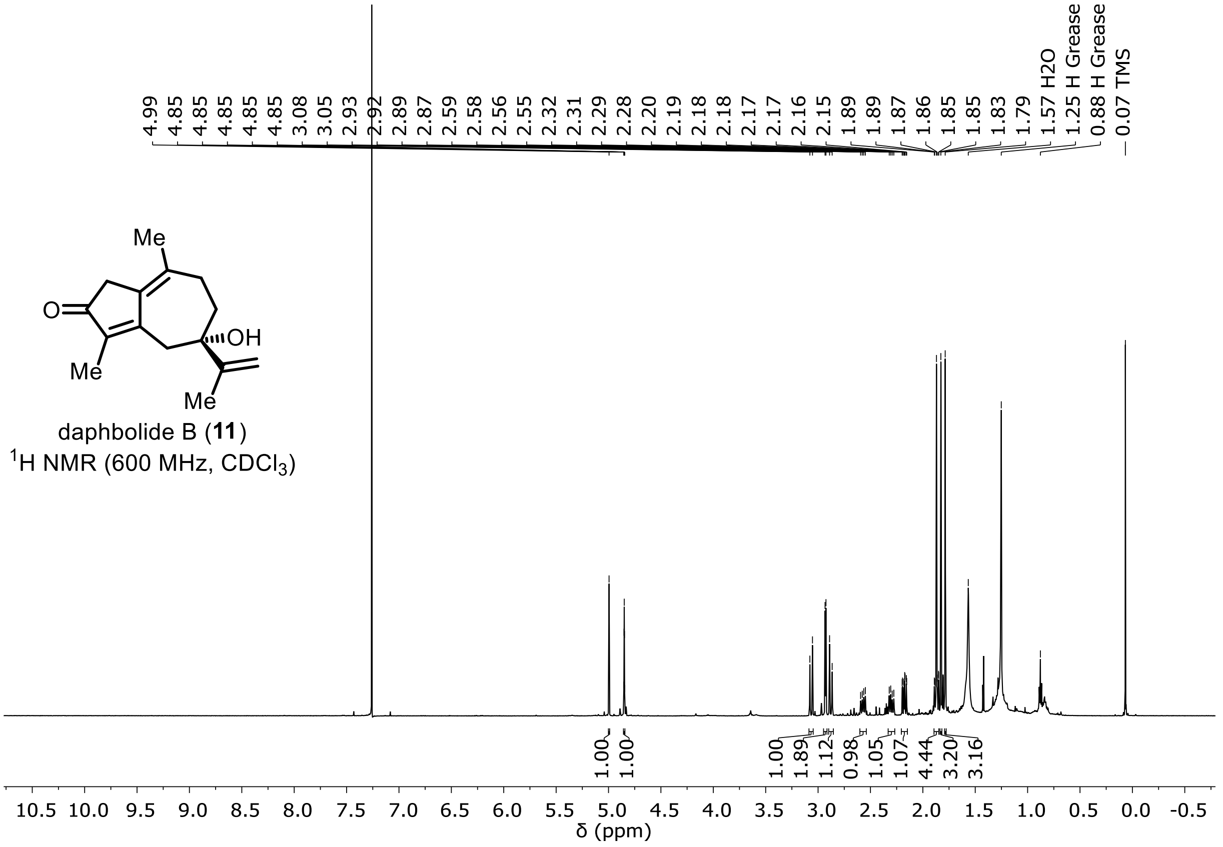
4.99
4.85
4.85
4.85
4.85
4.85
3.08
3.05
2.93
2.92
2.89
2.87
2.59
2.58
2.56
2.55
2.32
2.31
2.29
2.28
2.20
2.19
2.18
2.18
2.17
2.17
2.16
2.15
1.89
1.89
1.87
1.86
1.85
1.85
1.83
1.79
1.57 H2O
1.25 H Grease
0.88 H Grease
0.07 TMS
Me
O
OH
Me
Me
daphbolide B (11)
^{1}H NMR (600 MHz, CDCl$_3$)
1.00
1.00
1.00
1.89
1.12
0.98
1.05
1.07
4.44
3.20
3.16
10.5 10.0 9.5 9.0 8.5 8.0 7.5 7.0 6.5 6.0 5.5 5.0 4.5 4.0 3.5 3.0 2.5 2.0 1.5 1.0 0.5 0.0 -0.5
δ (ppm)

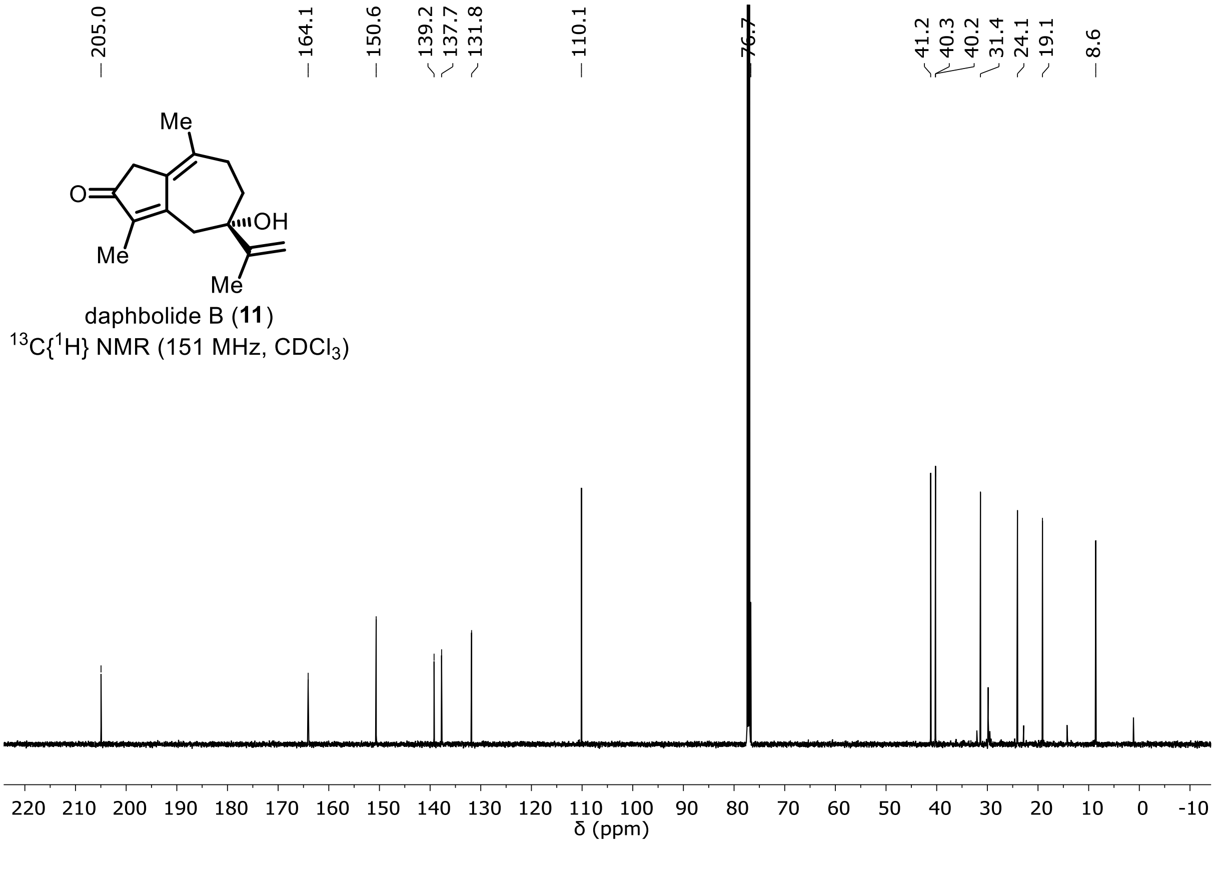
205.0
164.1
150.6
139.2
137.7
131.8
110.1
76.7
41.2
40.3
40.2
31.4
24.1
19.1
8.6
Me
O
OH
Me
Me
daphbolide B (11)
^{13}C{^{1}H} NMR (151 MHz, CDCl$_3$)
220 210 200 190 180 170 160 150 140 130 120 110 100 90 80 70 60 50 40 30 20 10 0 -10
δ (ppm)

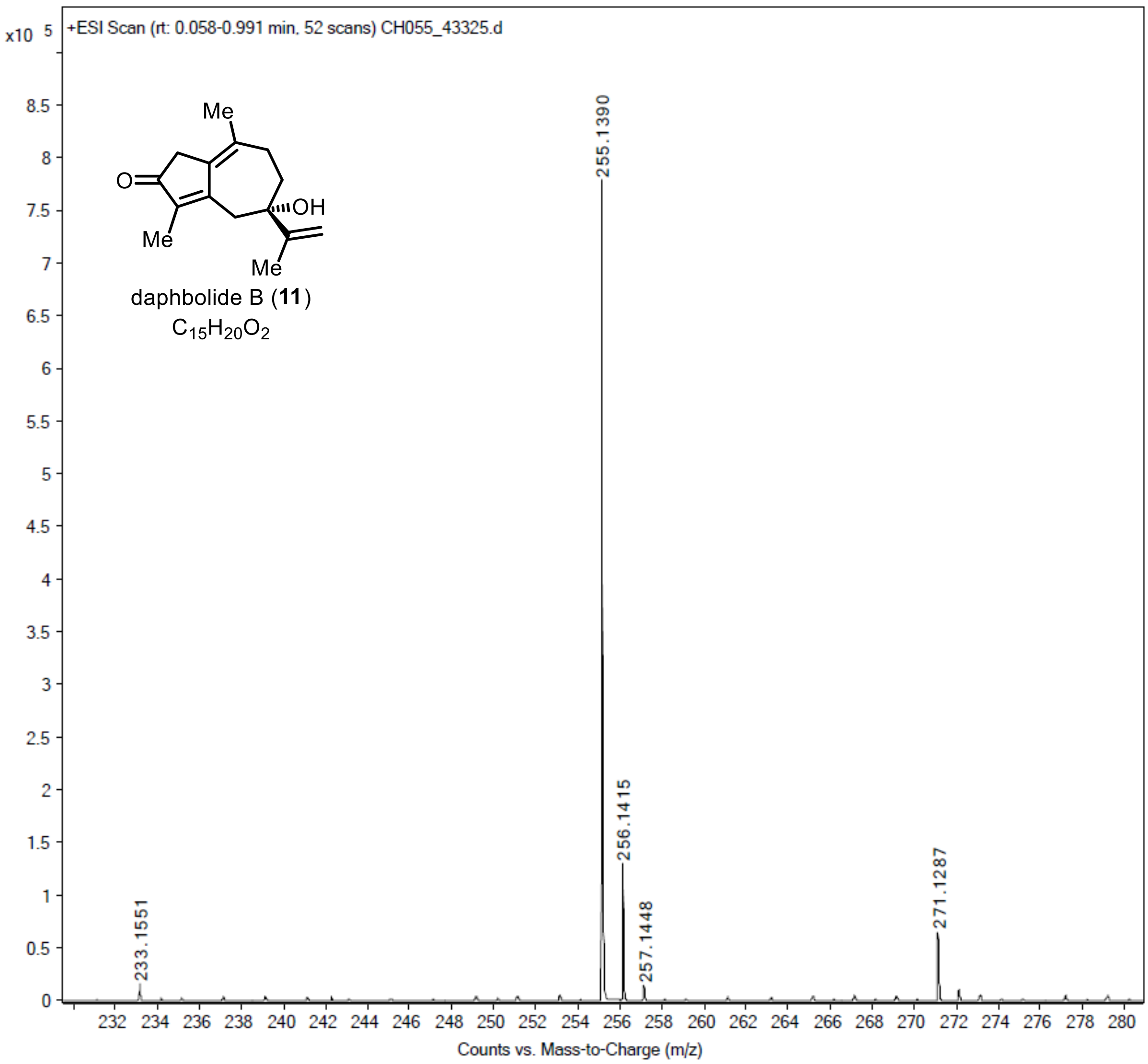

+ESI Scan (rt: 0.058-0.991 min, 52 scans) CH055_43325.d
x10 5
Me
O
OH
Me
Me
daphbolide B (**11**)
$C_{15}H_{20}O_2$
255.1390
256.1415
257.1448
271.1287
233.1551
Counts vs. Mass-to-Charge (m/z)

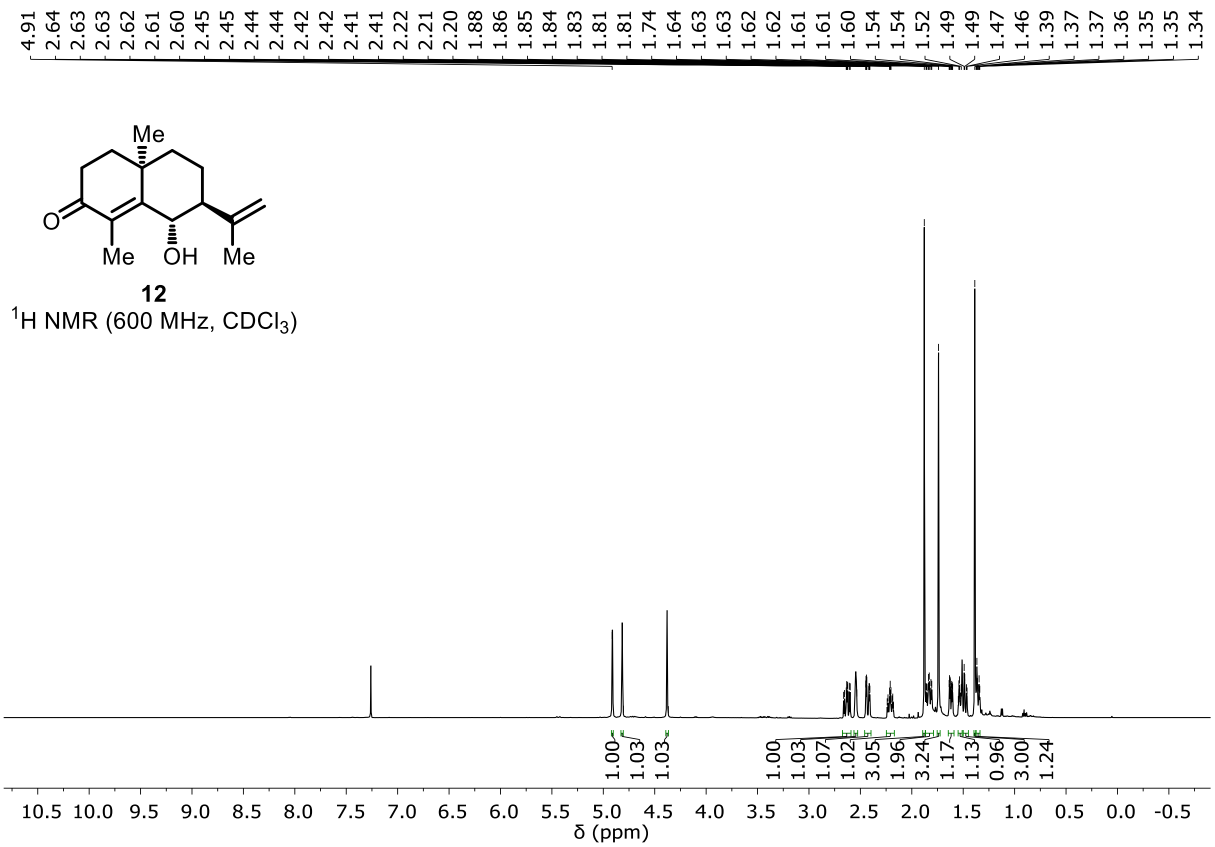

12

^{1}H NMR (600 MHz, $CDCl_3$)

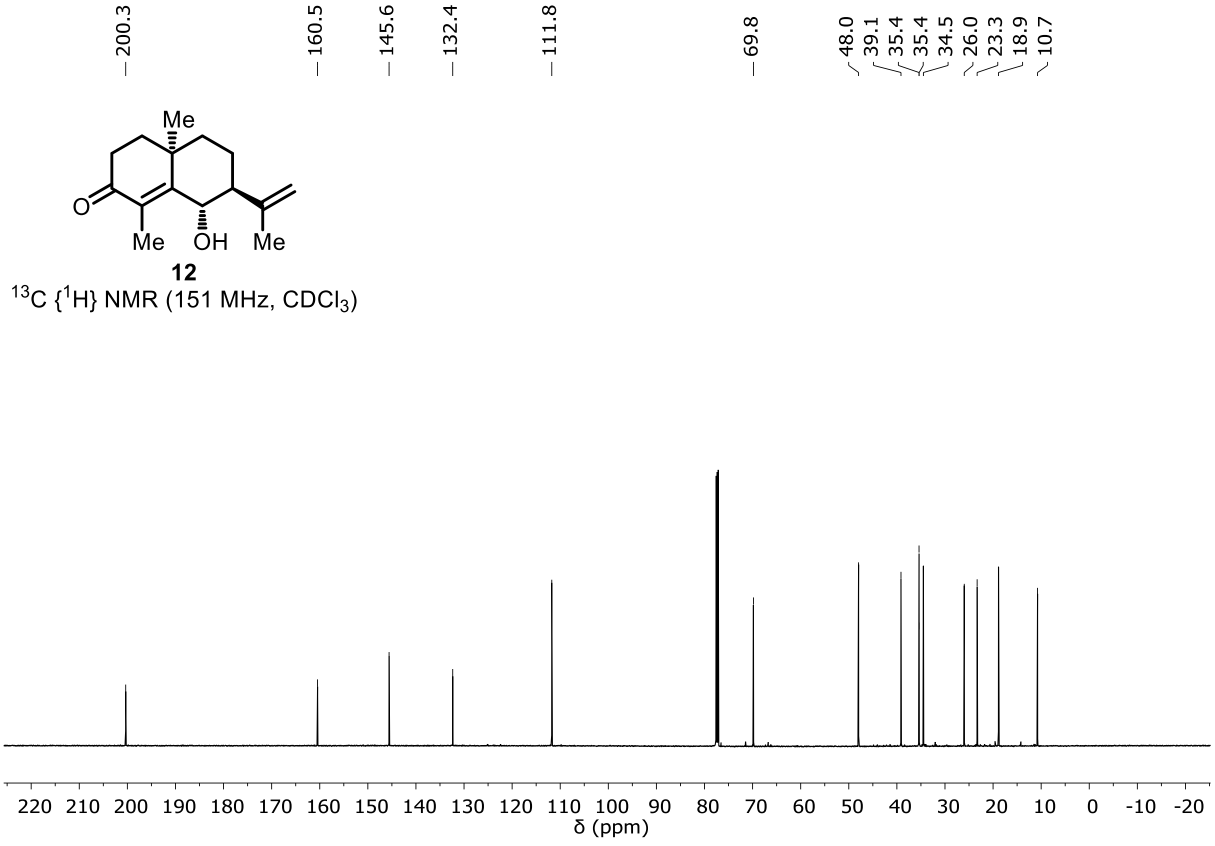

12

^{13}C {^{1}H} NMR (151 MHz, $CDCl_3$)

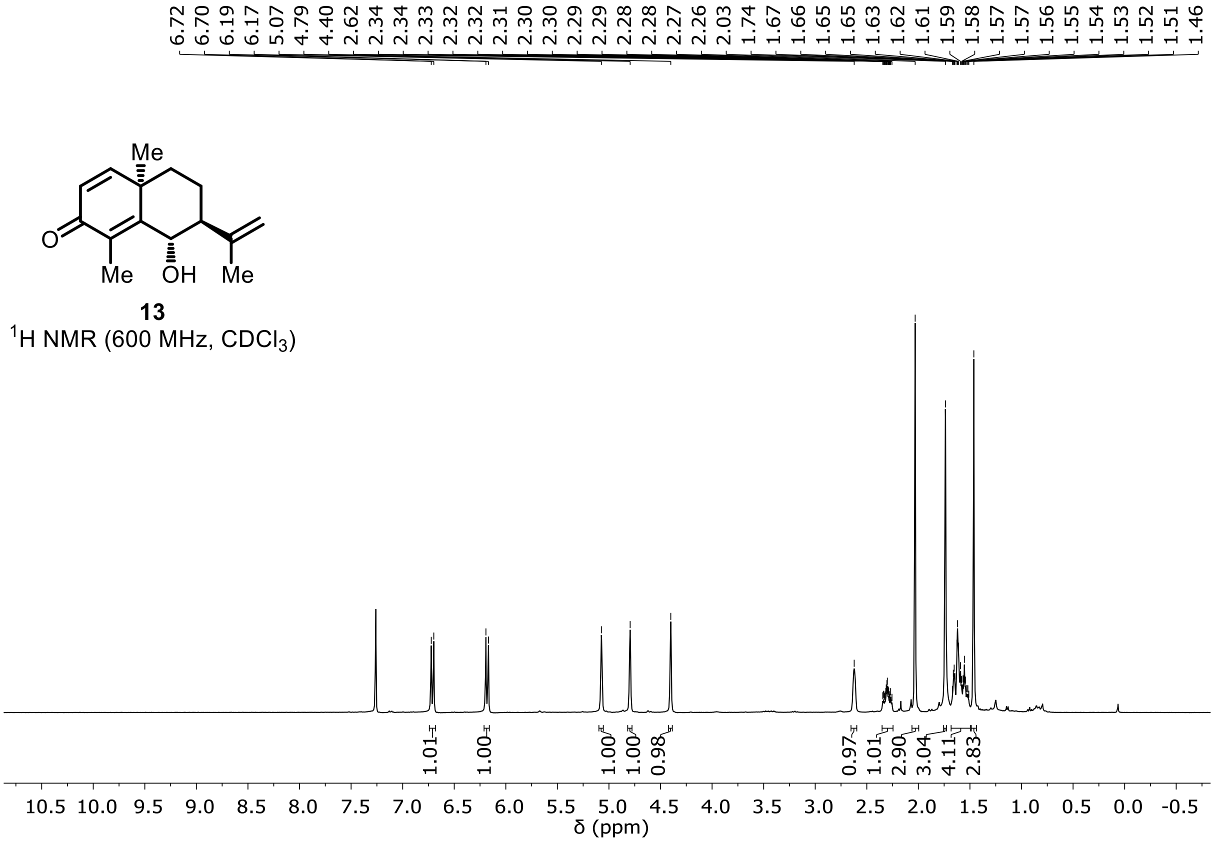
6.72 6.70 6.19 6.17 5.07 4.79 4.40 2.62 2.34 2.34 2.33 2.32 2.32 2.31 2.30 2.30 2.29 2.29 2.28 2.28 2.27 2.26 2.03 1.74 1.67 1.66 1.65 1.65 1.63 1.62 1.61 1.59 1.58 1.57 1.57 1.56 1.55 1.54 1.53 1.52 1.51 1.46
Me
O
Me
OH
Me
13
^{1}H NMR (600 MHz, $CDCl_3$)
1.01
1.00
1.00
1.00
0.98
0.97
1.01
2.90
3.04
4.11
2.83
10.5 10.0 9.5 9.0 8.5 8.0 7.5 7.0 6.5 6.0 5.5 5.0 4.5 4.0 3.5 3.0 2.5 2.0 1.5 1.0 0.5 0.0 -0.5
δ (ppm)

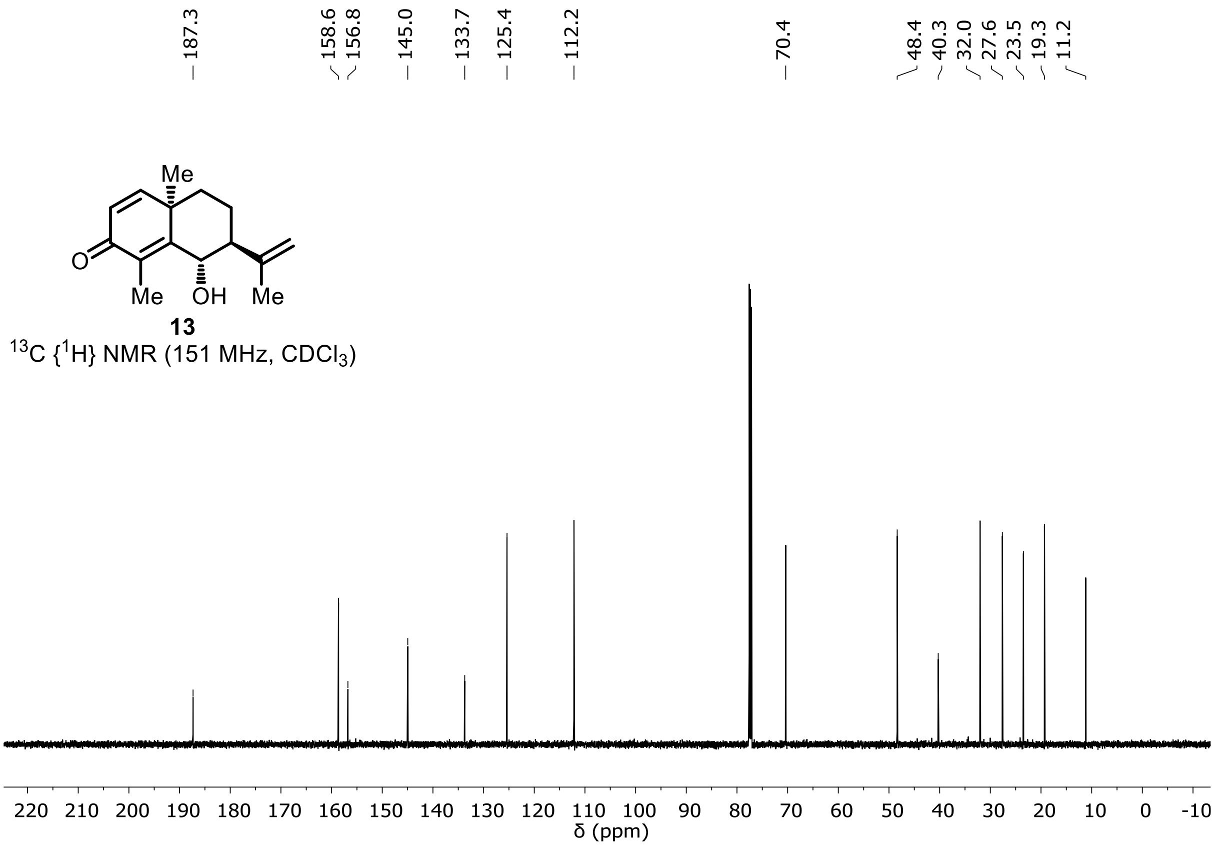
187.3
158.6 156.8
145.0
133.7
125.4
112.2
70.4
48.4 40.3 32.0 27.6 23.5 19.3 11.2
Me
O
Me
OH
Me
13
^{13}C {^{1}H} NMR (151 MHz, $CDCl_3$)
220 210 200 190 180 170 160 150 140 130 120 110 100 90 80 70 60 50 40 30 20 10 0 -10
δ (ppm)

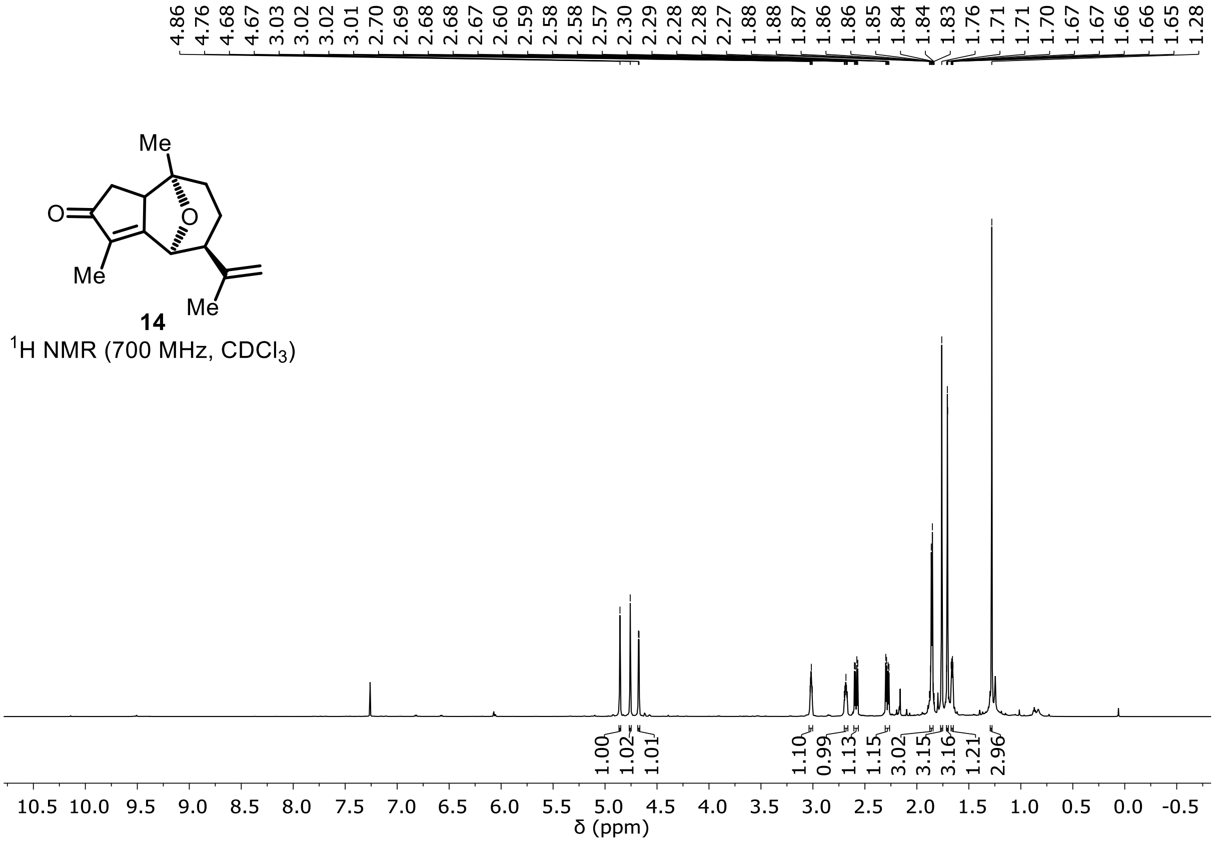

14
^1H NMR (700 MHz, $CDCl_3$)

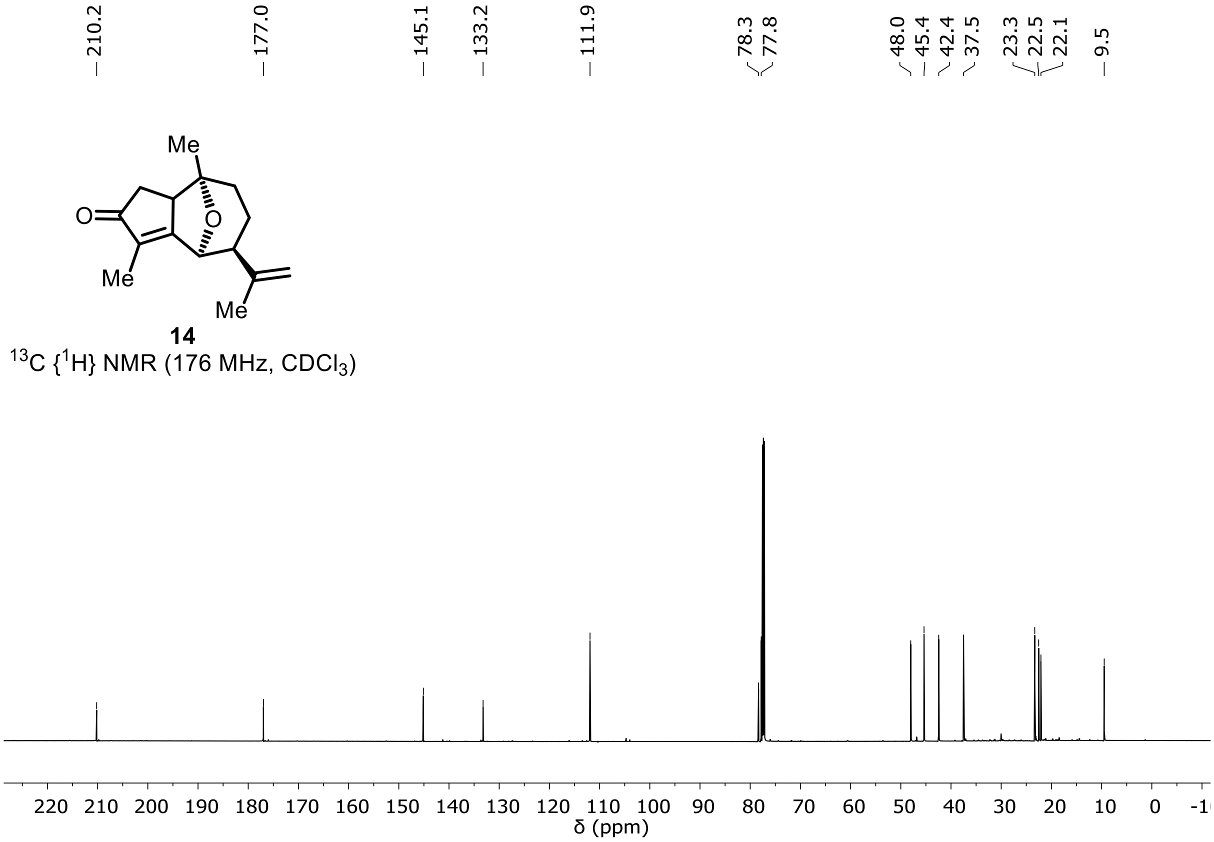

14
^{13}C {^1H} NMR (176 MHz, $CDCl_3$)

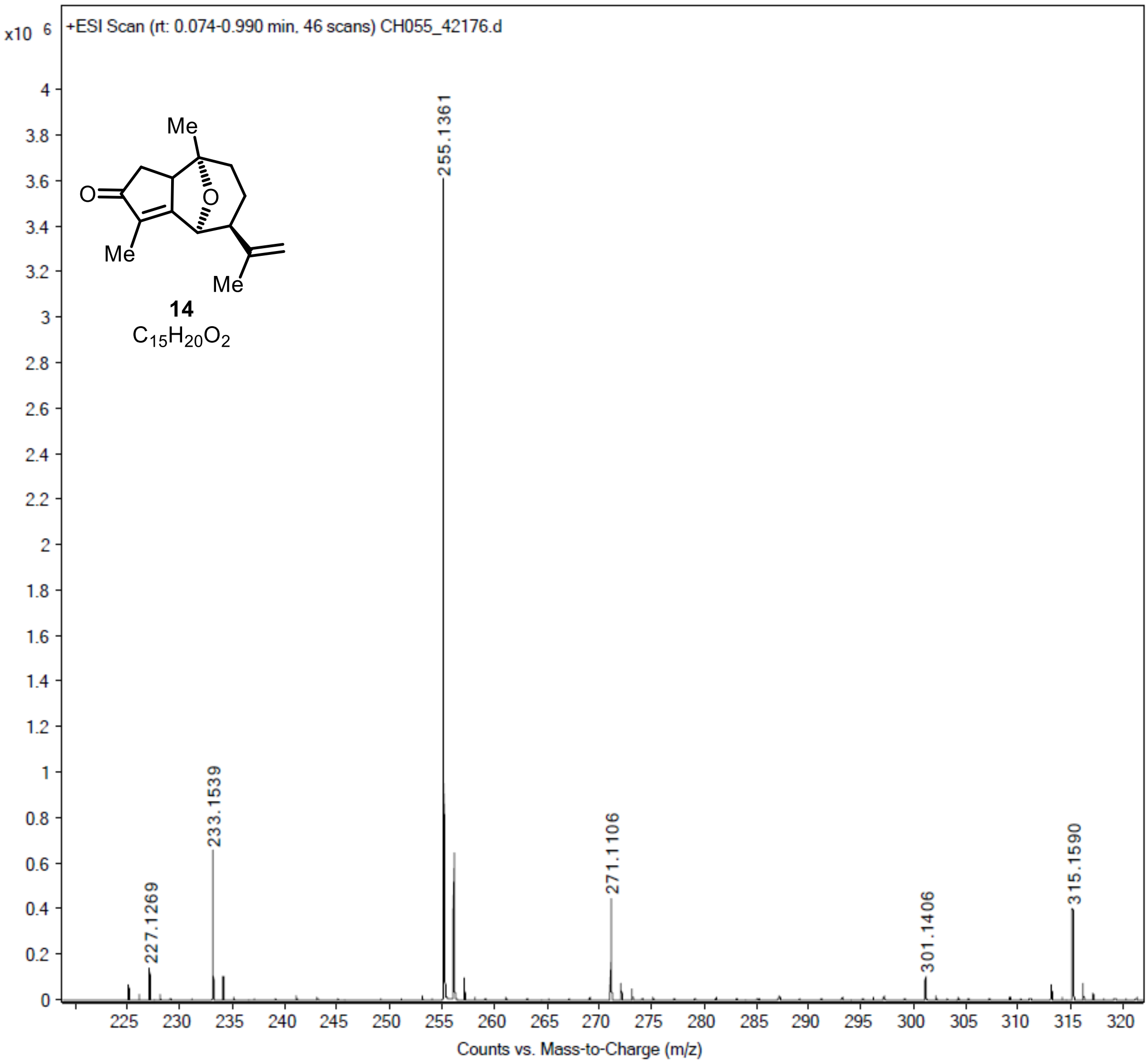
x10 6
+ESI Scan (rt: 0.074-0.990 min, 46 scans) CH055_42176.d
Me
O
O
Me
Me
14
$C_{15}H_{20}O_2$
255.1361
233.1539
227.1269
271.1106
301.1406
315.1590
Counts vs. Mass-to-Charge (m/z)

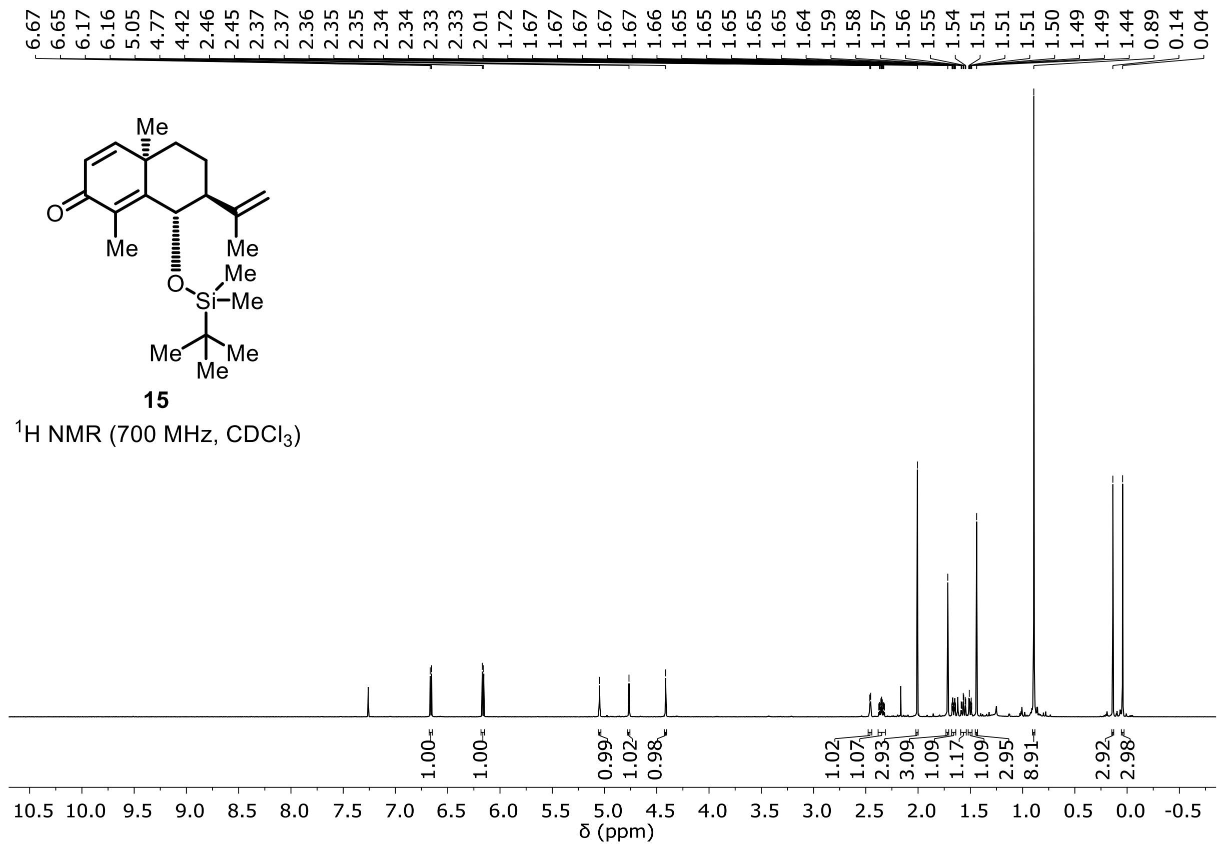

15

^{1}H NMR (700 MHz, $CDCl_3$)

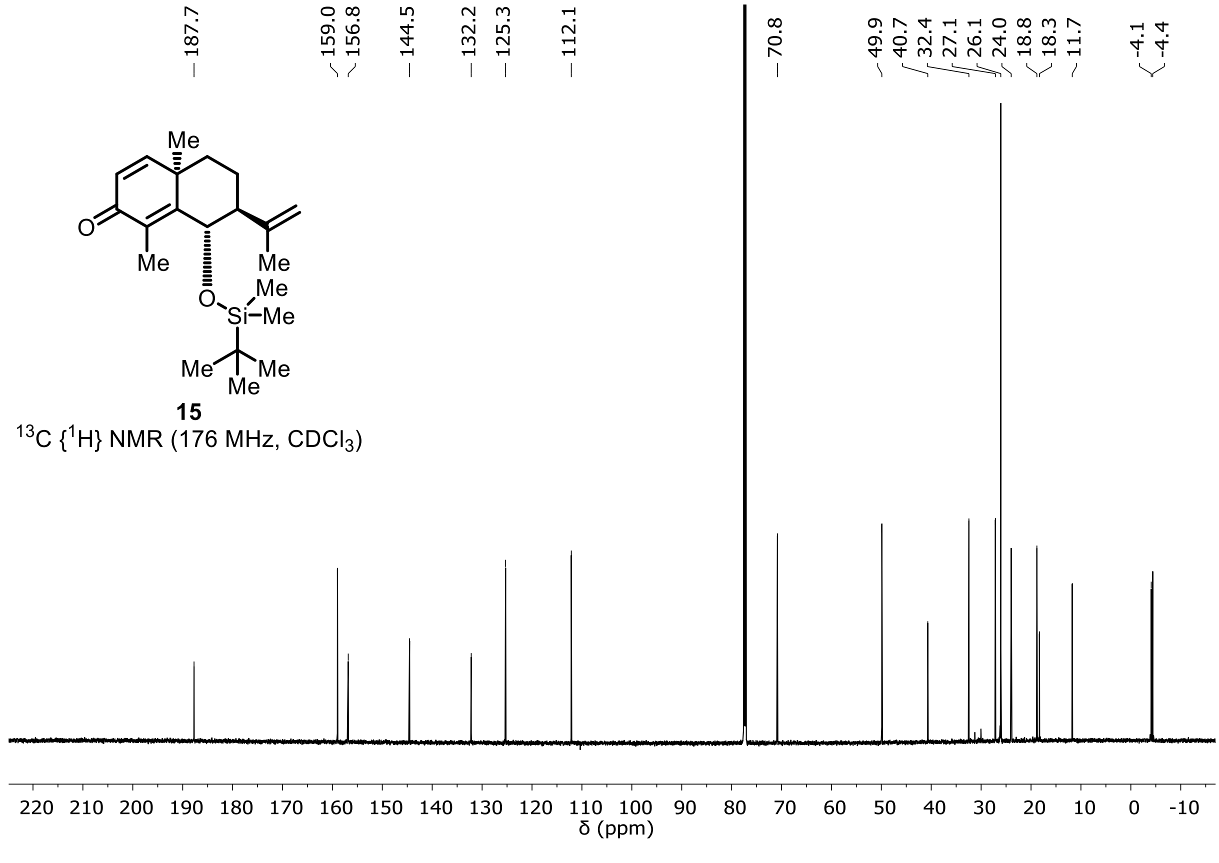

15

^{13}C {^{1}H} NMR (176 MHz, $CDCl_3$)

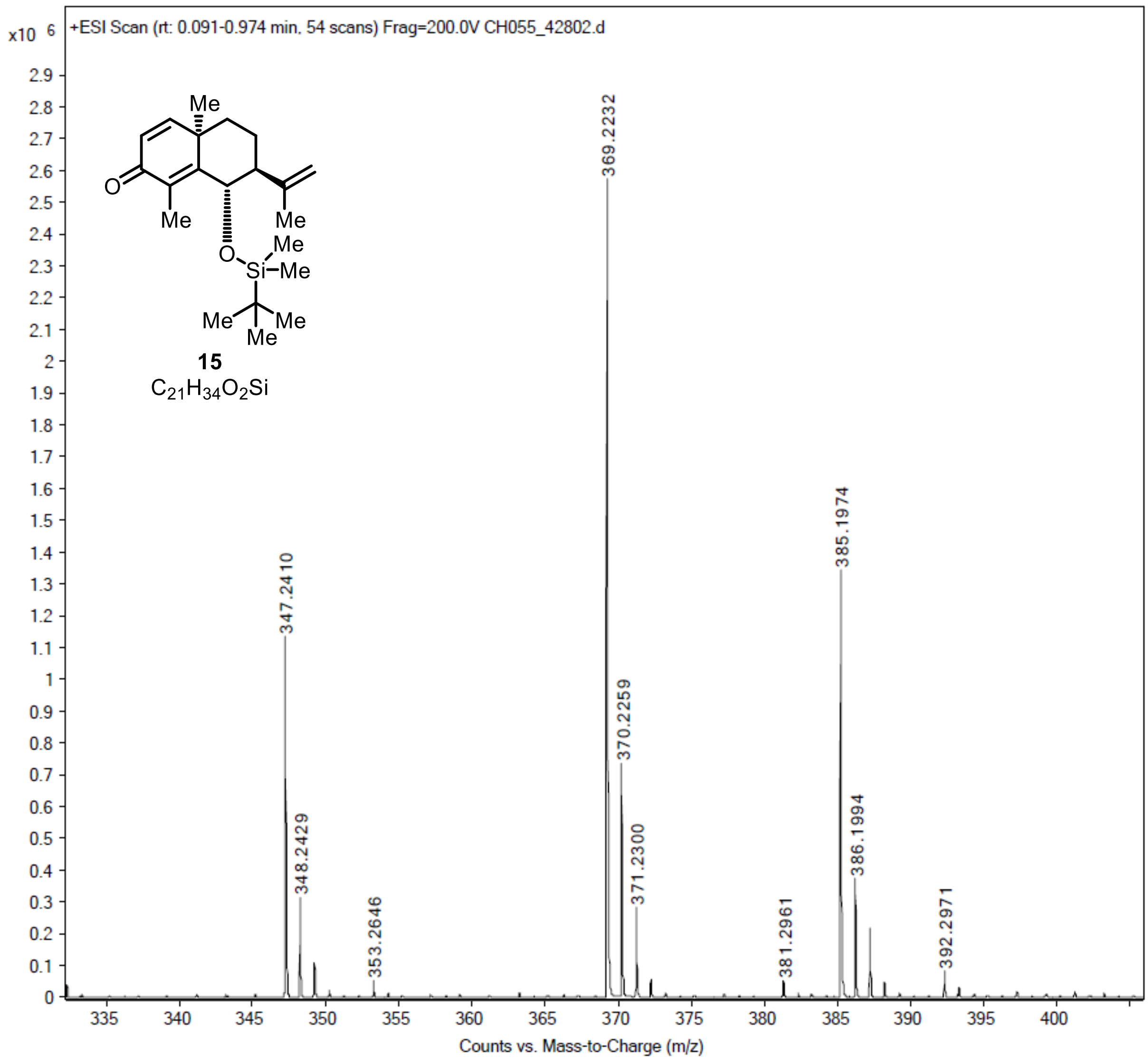
+ESI Scan (rt: 0.091-0.974 min, 54 scans) Frag=200.0V CH055_42802.d
x10 6
15
C21H34O2Si
369.2232
385.1974
347.2410
370.2259
386.1994
371.2300
348.2429
353.2646
381.2961
392.2971
Counts vs. Mass-to-Charge (m/z)

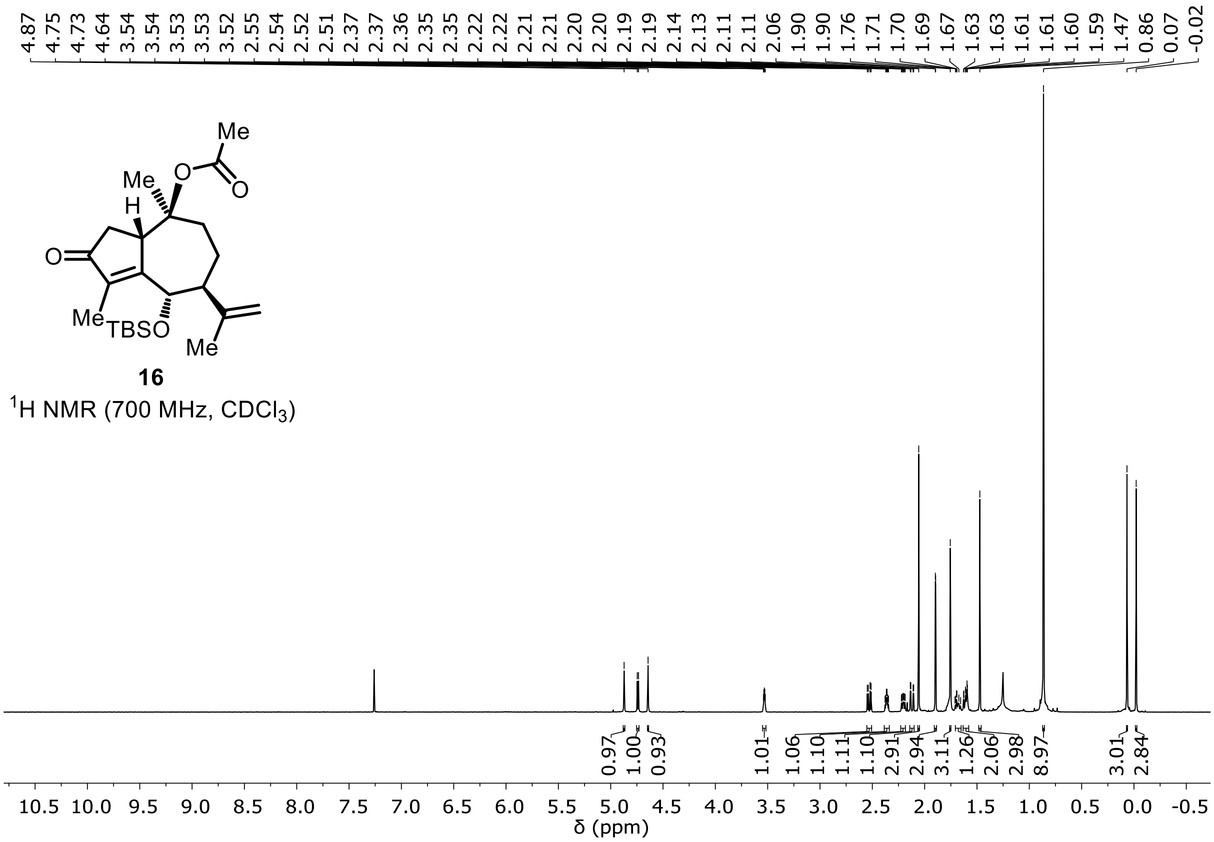

16

^{1}H NMR (700 MHz, $CDCl_3$)

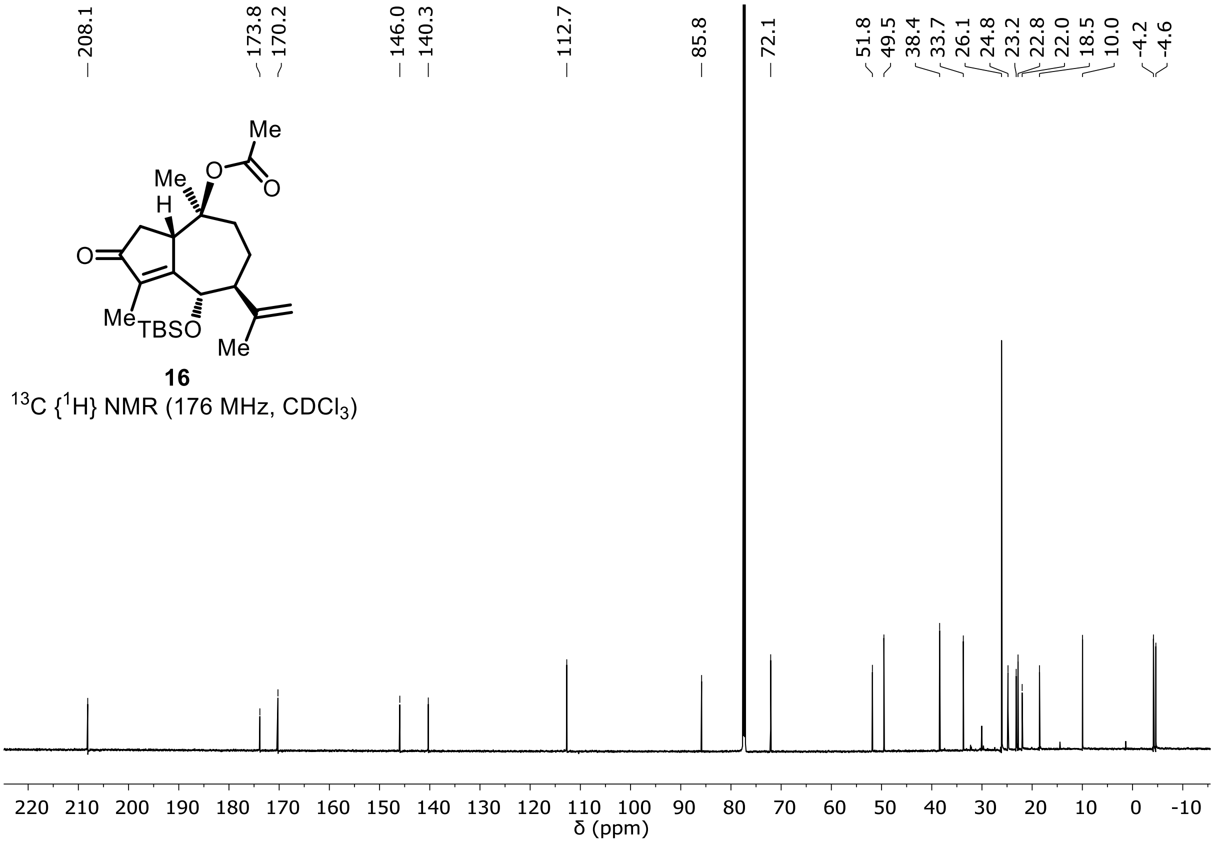

16

^{13}C {^{1}H} NMR (176 MHz, $CDCl_3$)

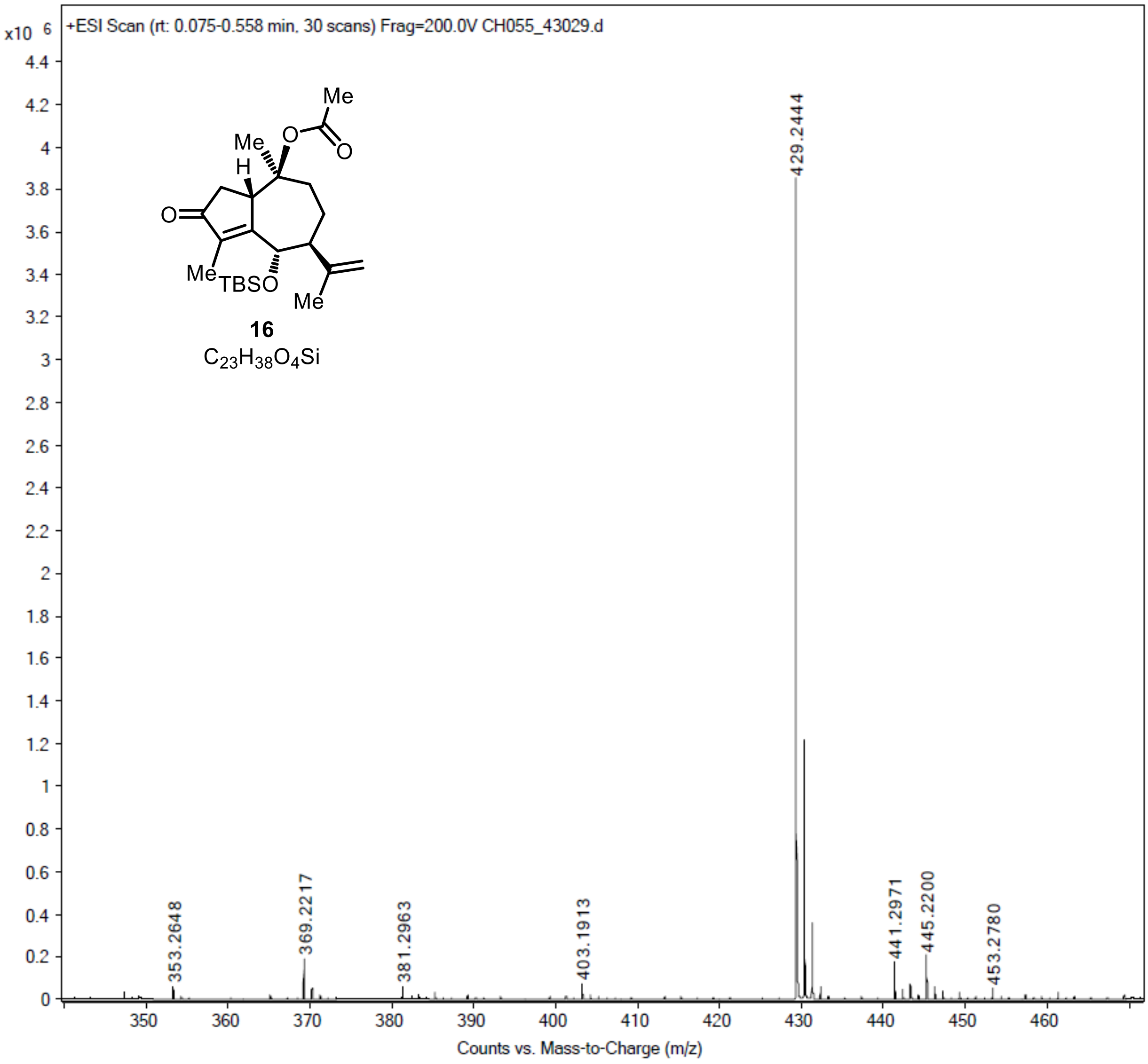

x10 6
+ESI Scan (rt: 0.075-0.558 min, 30 scans) Frag=200.0V CH055_43029.d
Me
O
O
Me
H
O
Me
TBSO
Me
16
C23H38O4Si
429.2444
353.2648
369.2217
381.2963
403.1913
441.2971
445.2200
453.2780
Counts vs. Mass-to-Charge (m/z)

21

^{1}H NMR (700 MHz, $CDCl_3$)

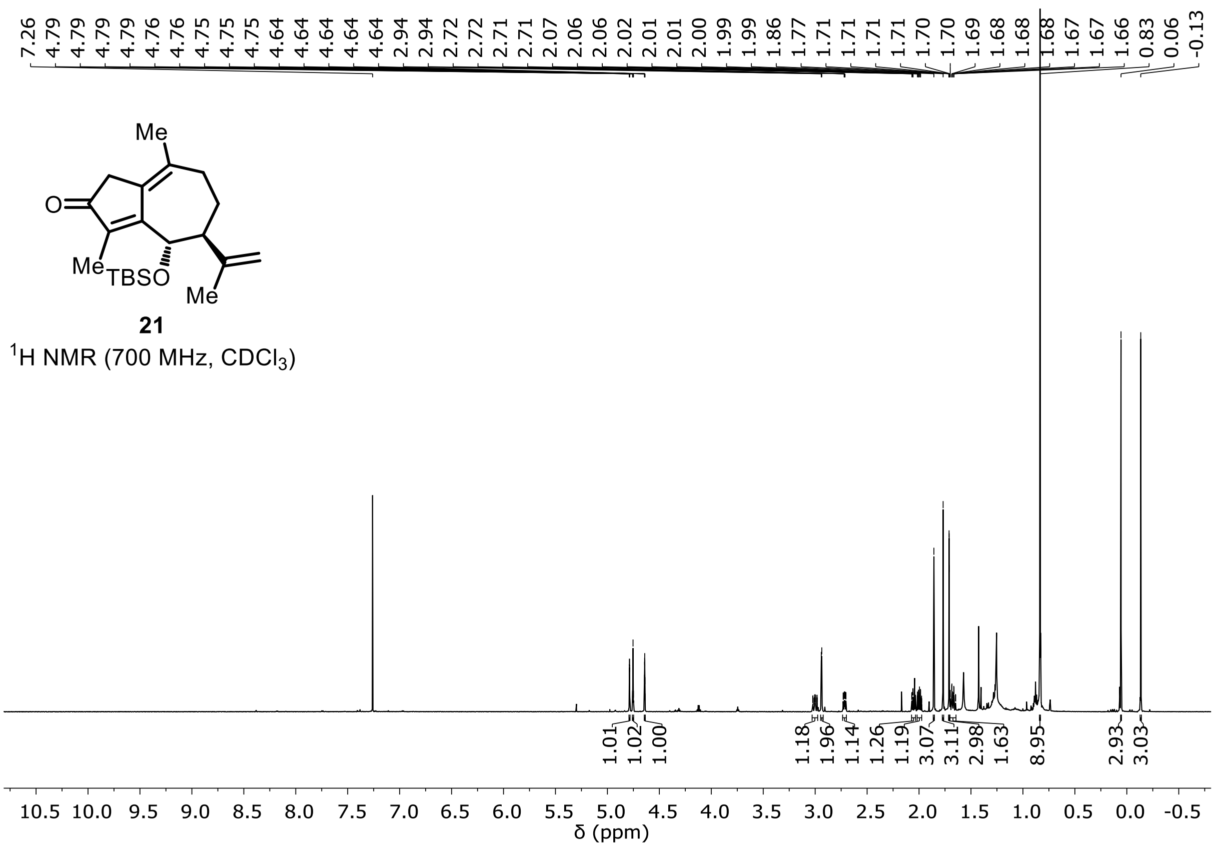

21

^{13}C {^{1}H} NMR (176 MHz, $CDCl_3$)

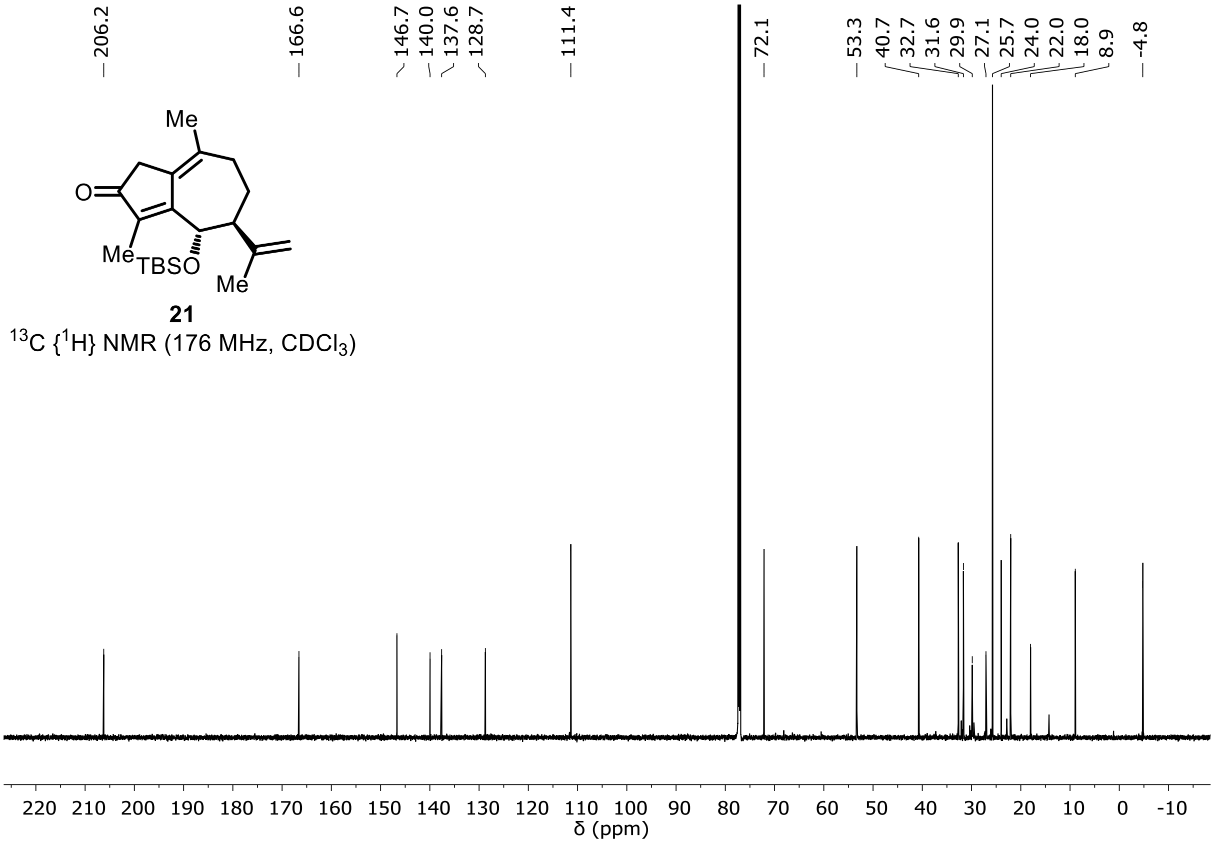

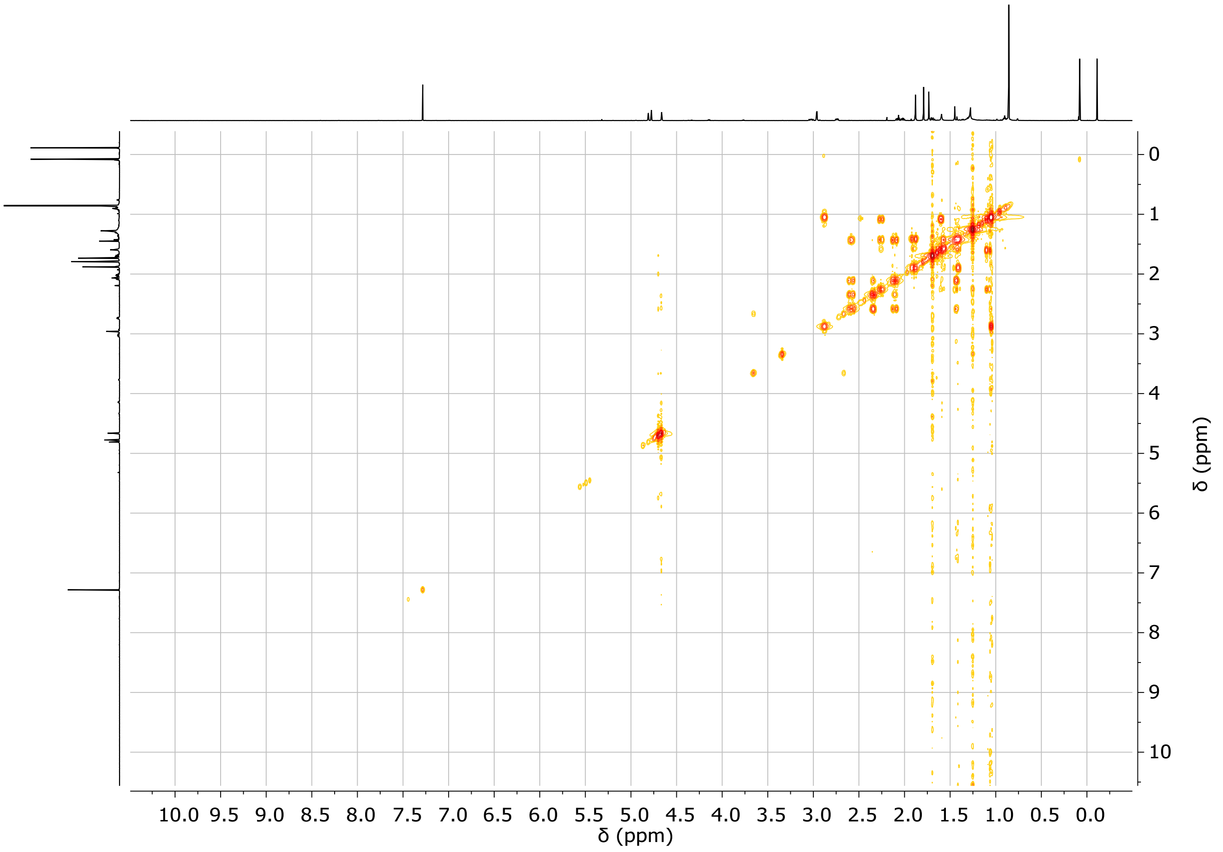

Figure S7. COSY spectra of **21**.

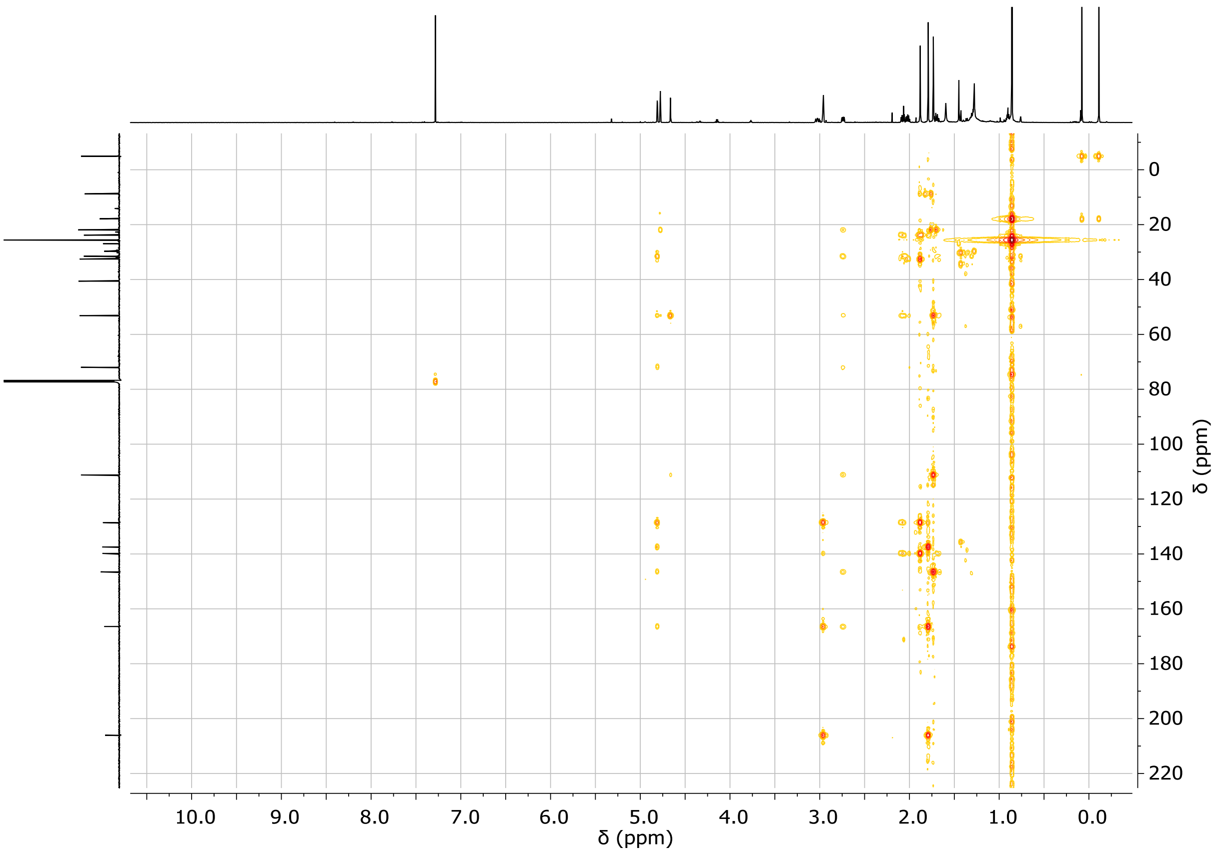

Figure S8 HMBC spectra of **21**.

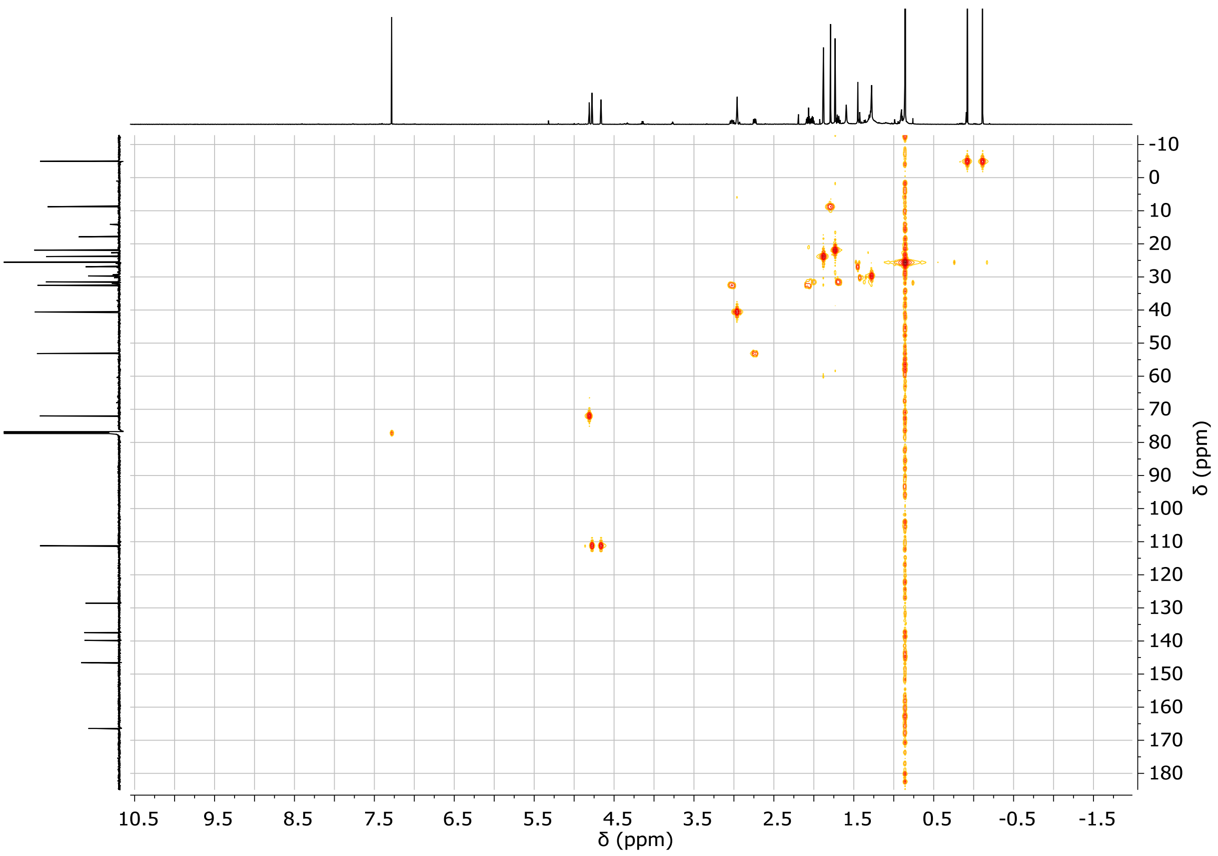

Figure S9. HMQC spectra of **21**.

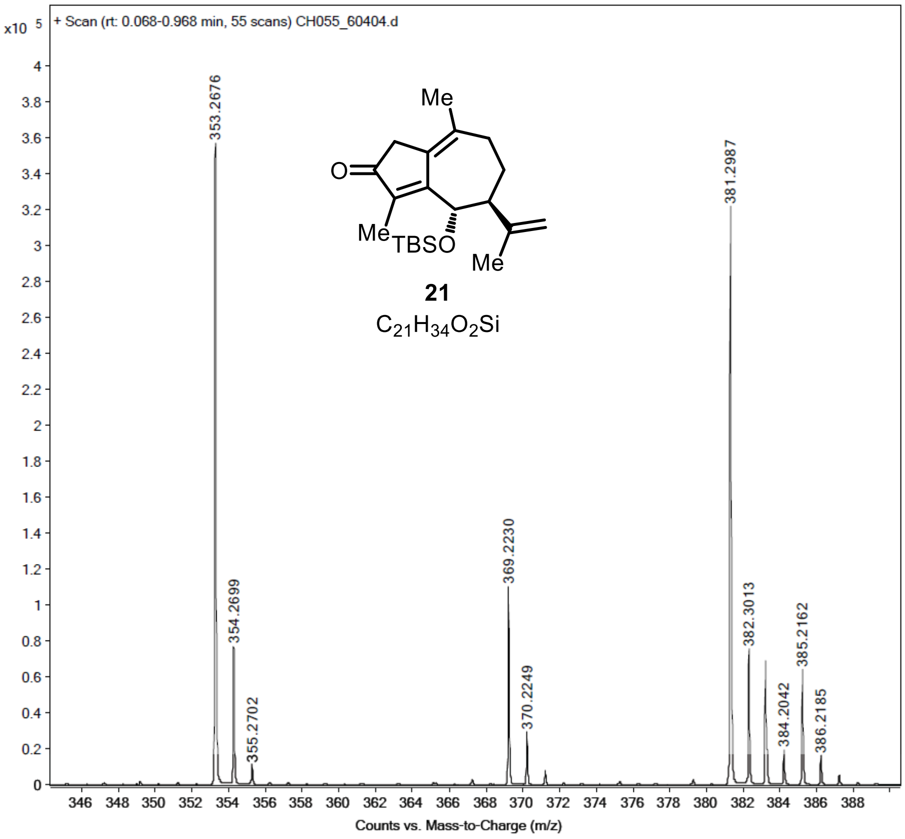

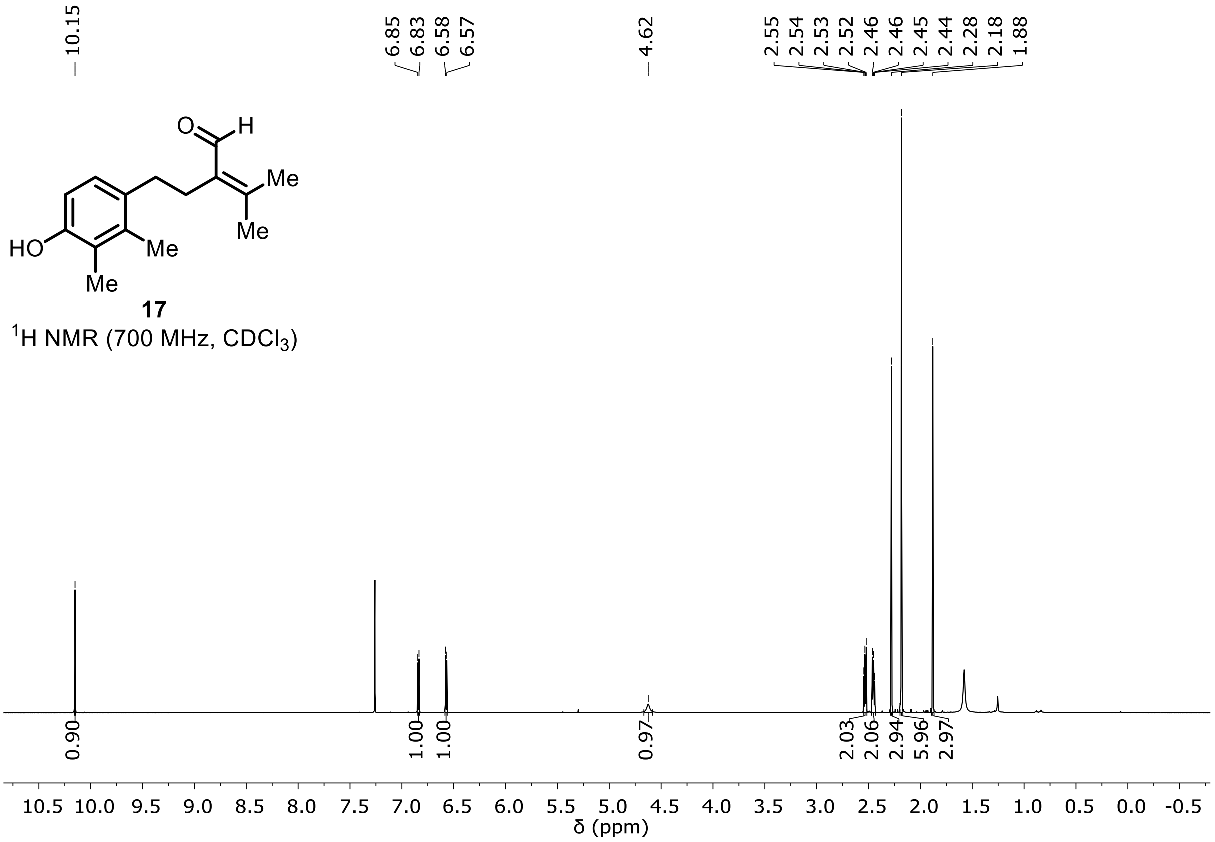
10.15
6.85
6.83
6.58
6.57
4.62
2.55
2.54
2.53
2.52
2.46
2.46
2.45
2.44
2.28
2.18
1.88
O
H
Me
Me
HO
Me
Me
17
1H NMR (700 MHz, CDCl3)
0.90
1.00
1.00
0.97
2.03
2.06
2.94
5.96
2.97
10.5 10.0 9.5 9.0 8.5 8.0 7.5 7.0 6.5 6.0 5.5 5.0 4.5 4.0 3.5 3.0 2.5 2.0 1.5 1.0 0.5 0.0 -0.5
δ (ppm)

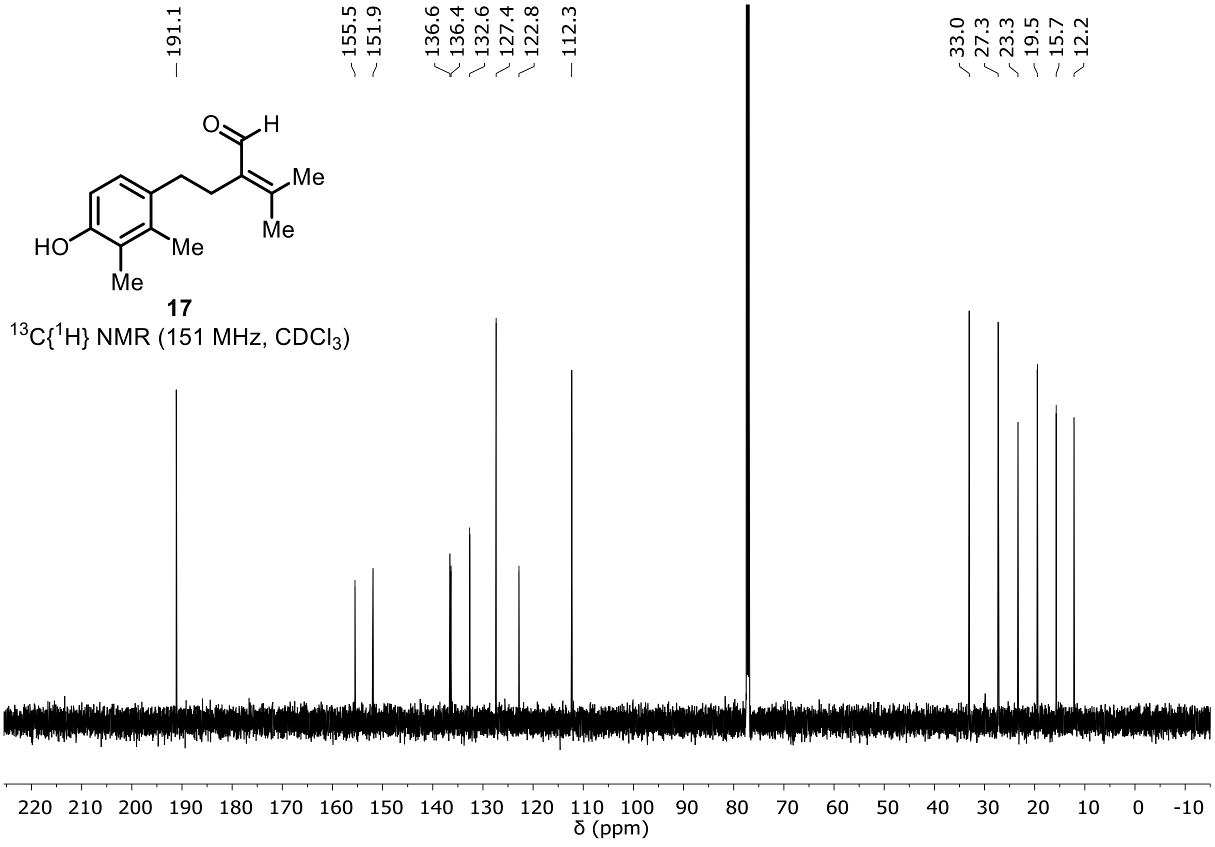
191.1
155.5
151.9
136.6
136.4
132.6
127.4
122.8
112.3
33.0
27.3
23.3
19.5
15.7
12.2
O
H
Me
Me
HO
Me
Me
17
13C{1H} NMR (151 MHz, CDCl3)
220 210 200 190 180 170 160 150 140 130 120 110 100 90 80 70 60 50 40 30 20 10 0 -10
δ (ppm)

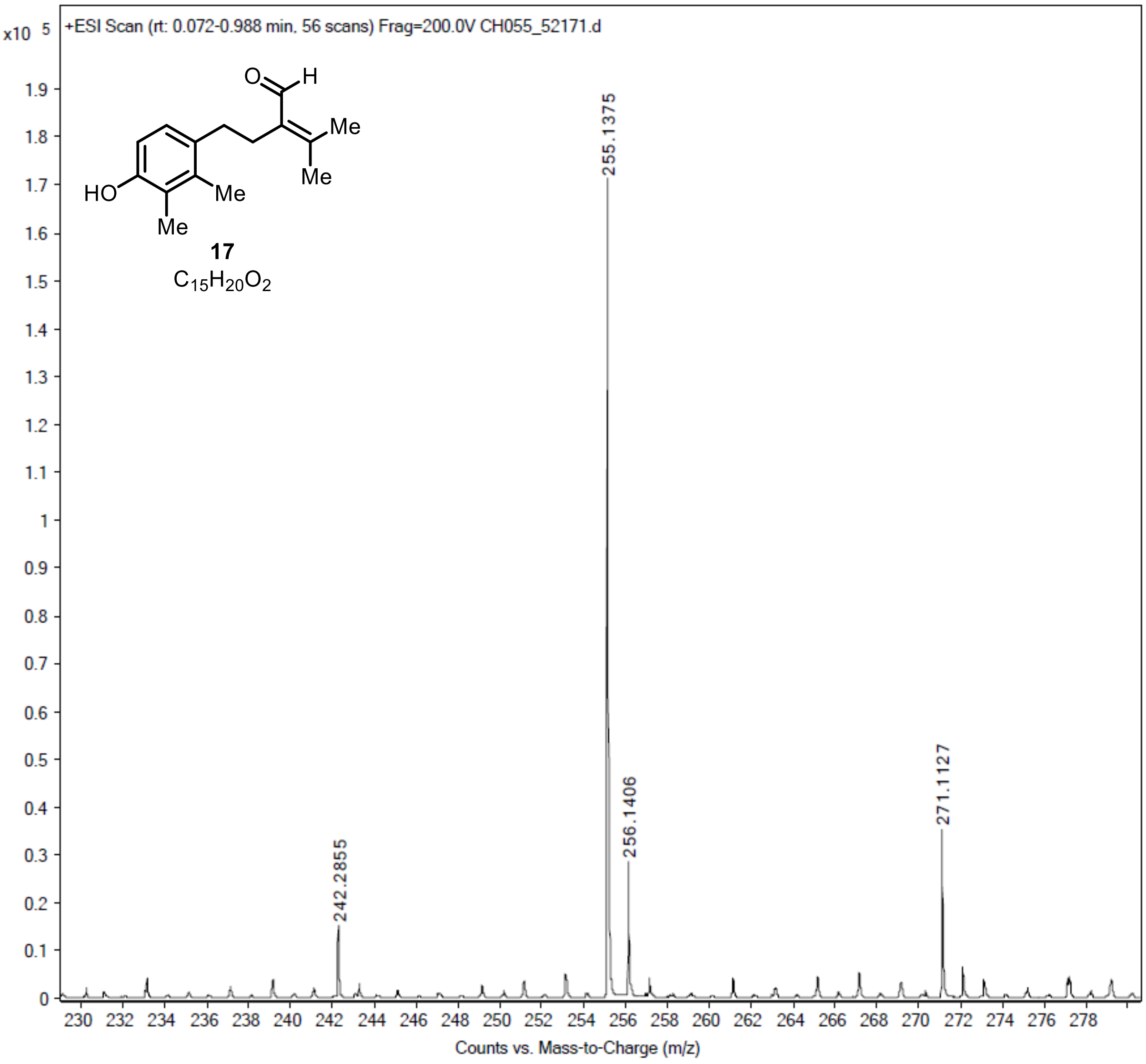

x10 5
+ESI Scan (rt: 0.072-0.988 min, 56 scans) Frag=200.0V CH055_52171.d
O
H
Me
Me
HO
Me
Me
17
$C_{15}H_{20}O_2$
255.1375
256.1406
271.1127
242.2855
Counts vs. Mass-to-Charge (m/z)

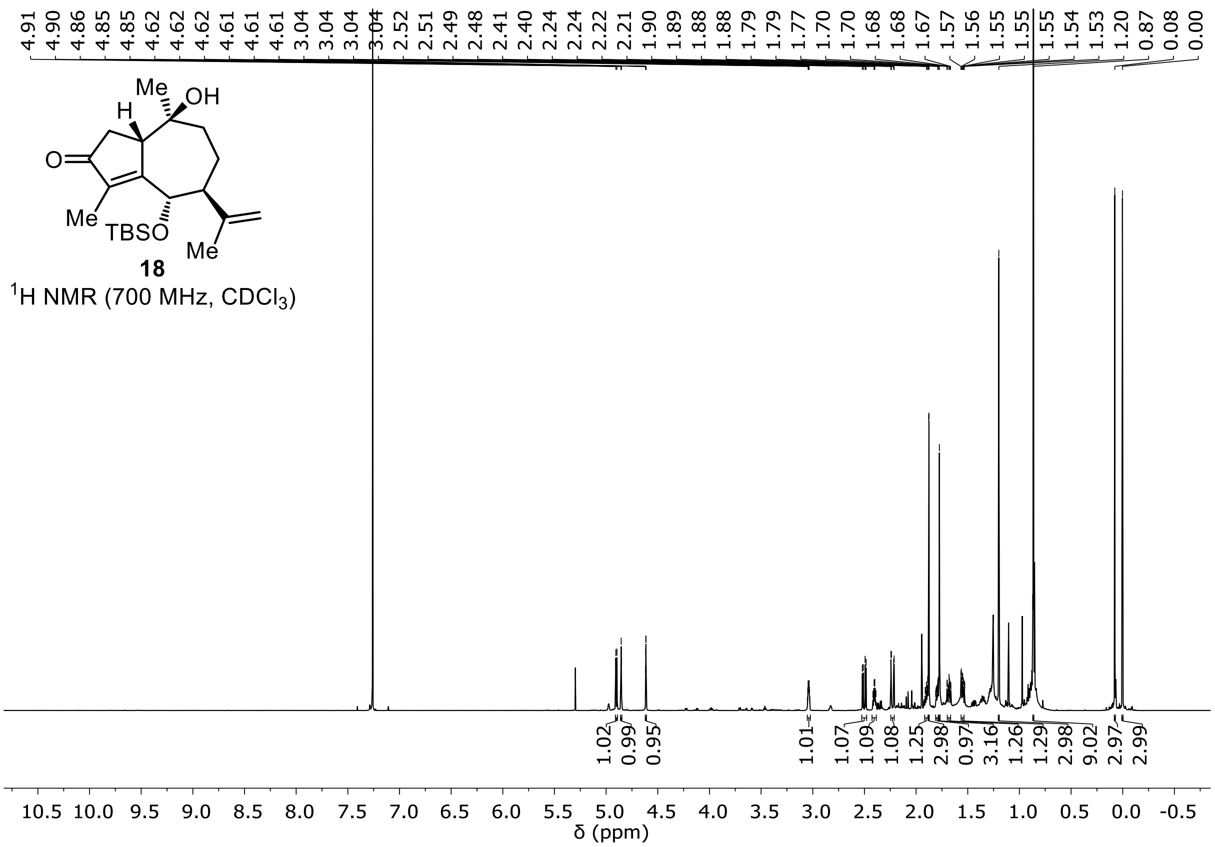

4.91 4.90 4.86 4.85 4.85 4.62 4.62 4.62 4.61 4.61 4.61 3.04 3.04 3.04 3.04 2.52 2.51 2.49 2.48 2.41 2.40 2.24 2.24 2.22 2.21 1.90 1.89 1.88 1.88 1.79 1.79 1.77 1.70 1.70 1.68 1.68 1.67 1.57 1.56 1.55 1.55 1.55 1.54 1.53 1.20 0.87 0.08 0.00
Me
OH
H
O
Me
TBSO
Me
18
1H NMR (700 MHz, CDCl3)
1.02 0.99 0.95 1.01 1.07 1.09 1.08 1.25 2.98 0.97 3.16 1.26 1.29 2.98 9.02 2.97 2.99
10.5 10.0 9.5 9.0 8.5 8.0 7.5 7.0 6.5 6.0 5.5 5.0 4.5 4.0 3.5 3.0 2.5 2.0 1.5 1.0 0.5 0.0 -0.5
δ (ppm)

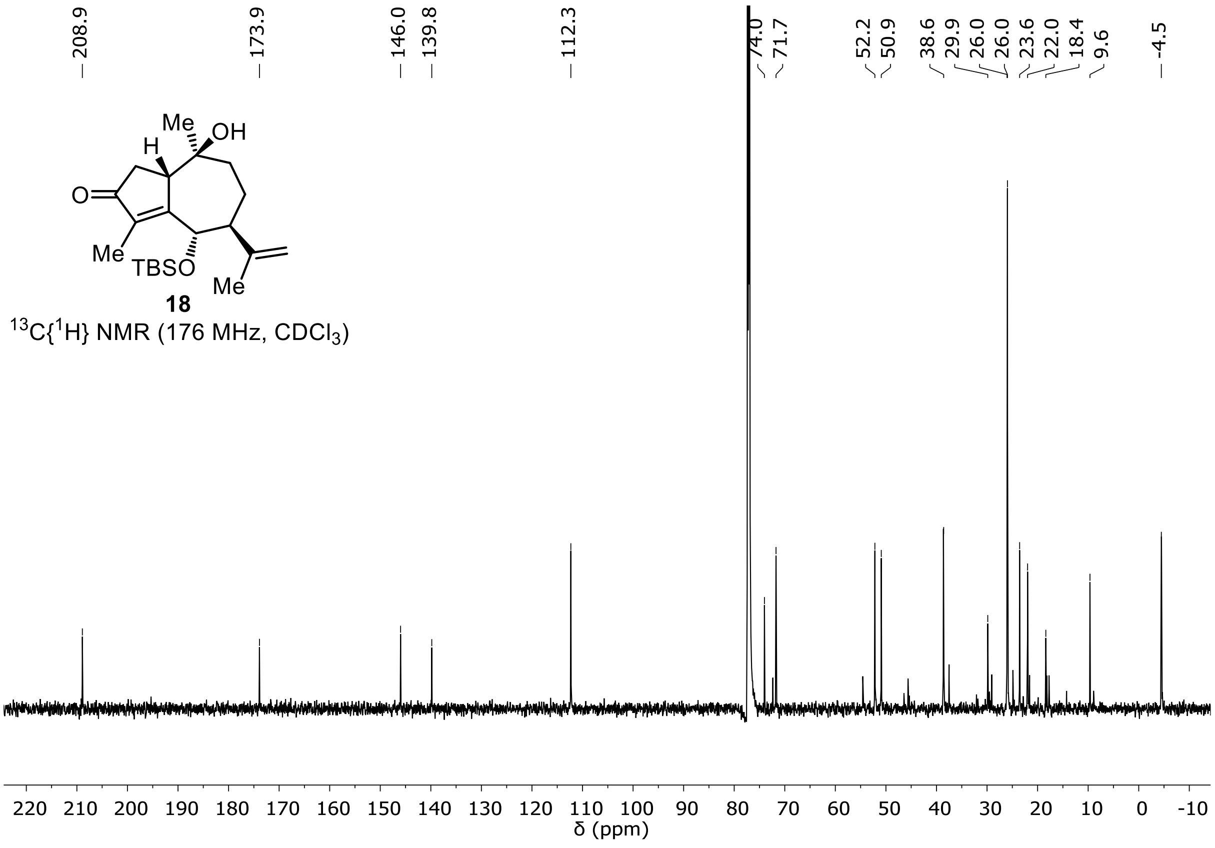

208.9 173.9 146.0 139.8 112.3 74.0 71.7 52.2 50.9 38.6 29.9 26.0 26.0 23.6 22.0 18.4 9.6 -4.5
Me
OH
H
O
Me
TBSO
Me
18
13C{1H} NMR (176 MHz, CDCl3)
220 210 200 190 180 170 160 150 140 130 120 110 100 90 80 70 60 50 40 30 20 10 0 -10
δ (ppm)

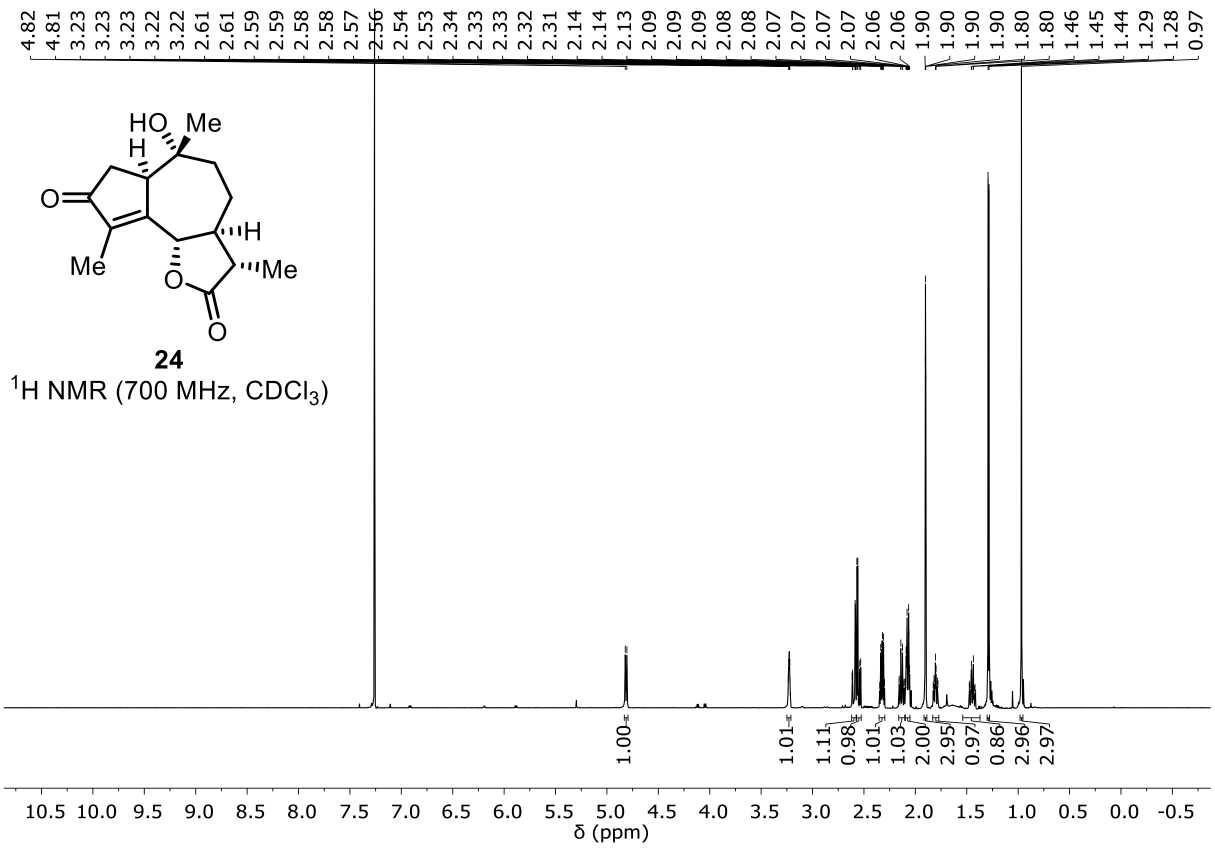
4.82 4.81 3.23 3.23 3.23 3.22 3.22 2.61 2.61 2.59 2.59 2.58 2.58 2.57 2.56 2.54 2.53 2.34 2.33 2.33 2.32 2.31 2.14 2.14 2.13 2.09 2.09 2.09 2.08 2.08 2.07 2.07 2.07 2.07 2.06 2.06 1.90 1.90 1.90 1.90 1.80 1.80 1.46 1.45 1.44 1.29 1.28 0.97
HO Me
H
O
H
Me
O
Me
O
24
¹H NMR (700 MHz, CDCl₃)
1.00 1.01 1.11 0.98 1.01 1.03 2.00 2.95 0.97 0.86 2.96 2.97
10.5 10.0 9.5 9.0 8.5 8.0 7.5 7.0 6.5 6.0 5.5 5.0 4.5 4.0 3.5 3.0 2.5 2.0 1.5 1.0 0.5 0.0 -0.5
δ (ppm)

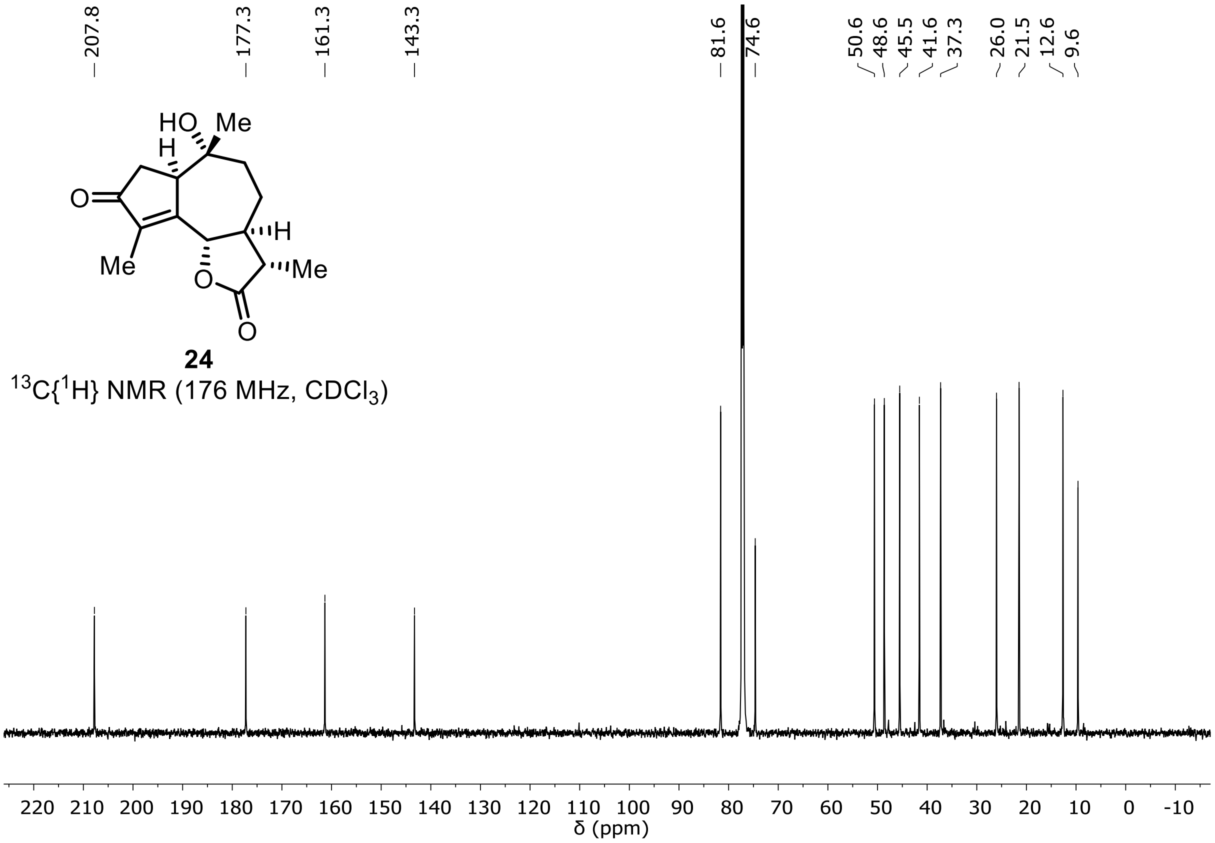
207.8 177.3 161.3 143.3 81.6 74.6 50.6 48.6 45.5 41.6 37.3 26.0 21.5 12.6 9.6
HO Me
H
O
H
Me
O
Me
O
24
¹³C{¹H} NMR (176 MHz, CDCl₃)
220 210 200 190 180 170 160 150 140 130 120 110 100 90 80 70 60 50 40 30 20 10 0 -10
δ (ppm)

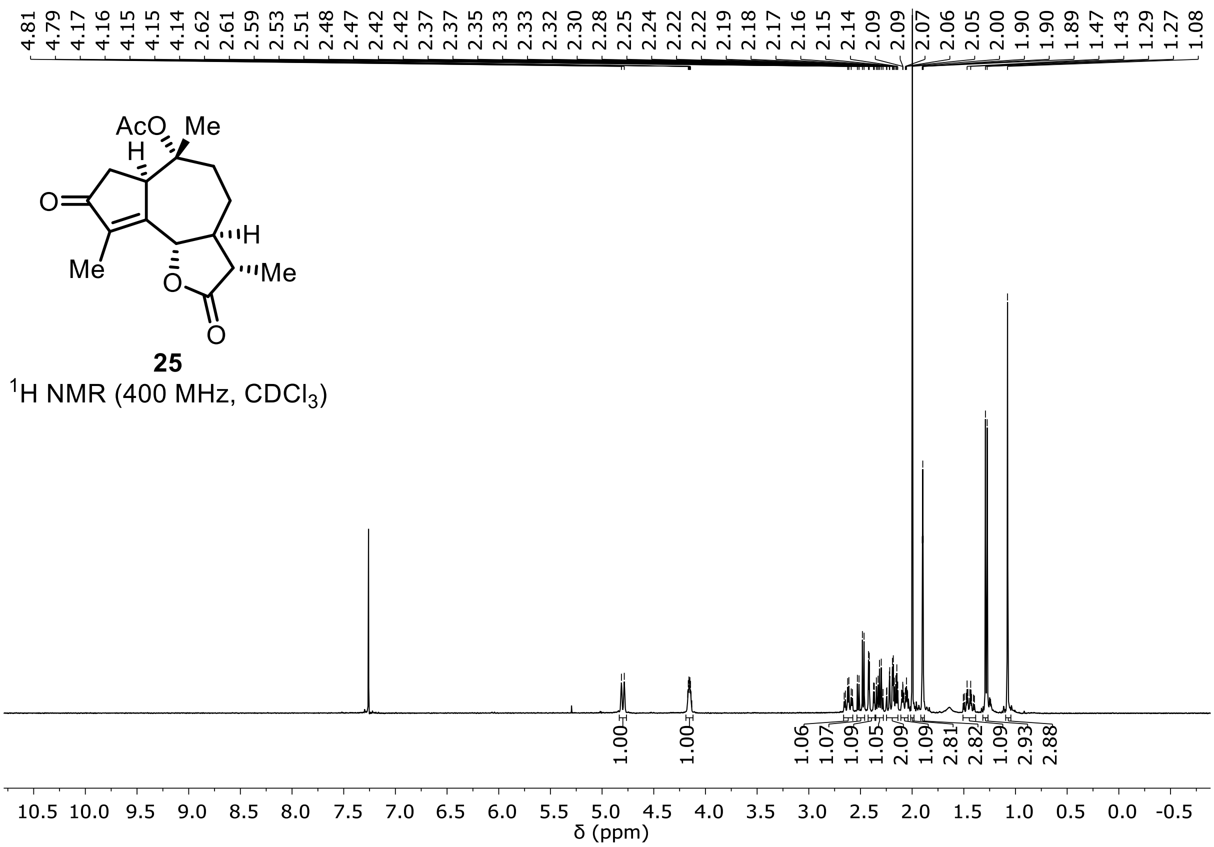

25

^{1}H NMR (400 MHz, $CDCl_3$)

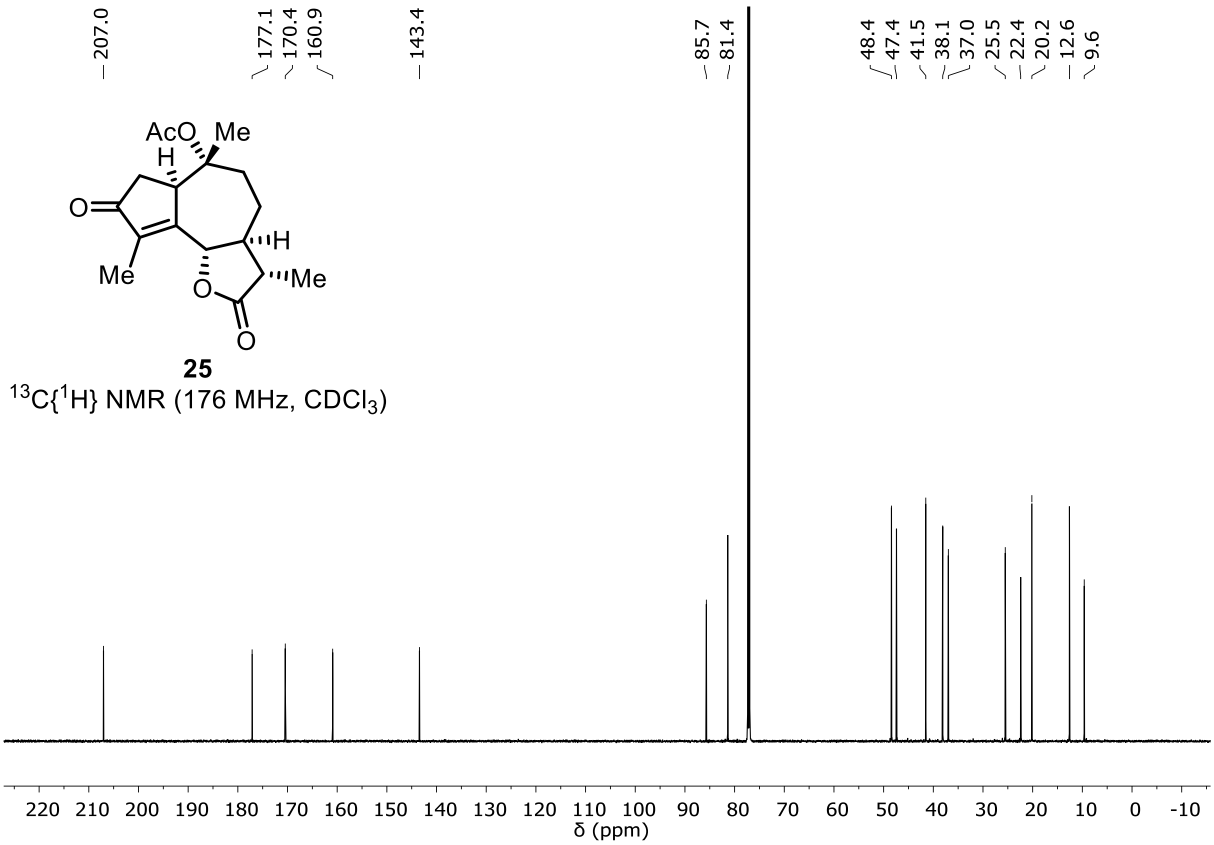

25

$^{13}C\{^{1}H\}$ NMR (176 MHz, $CDCl_3$)

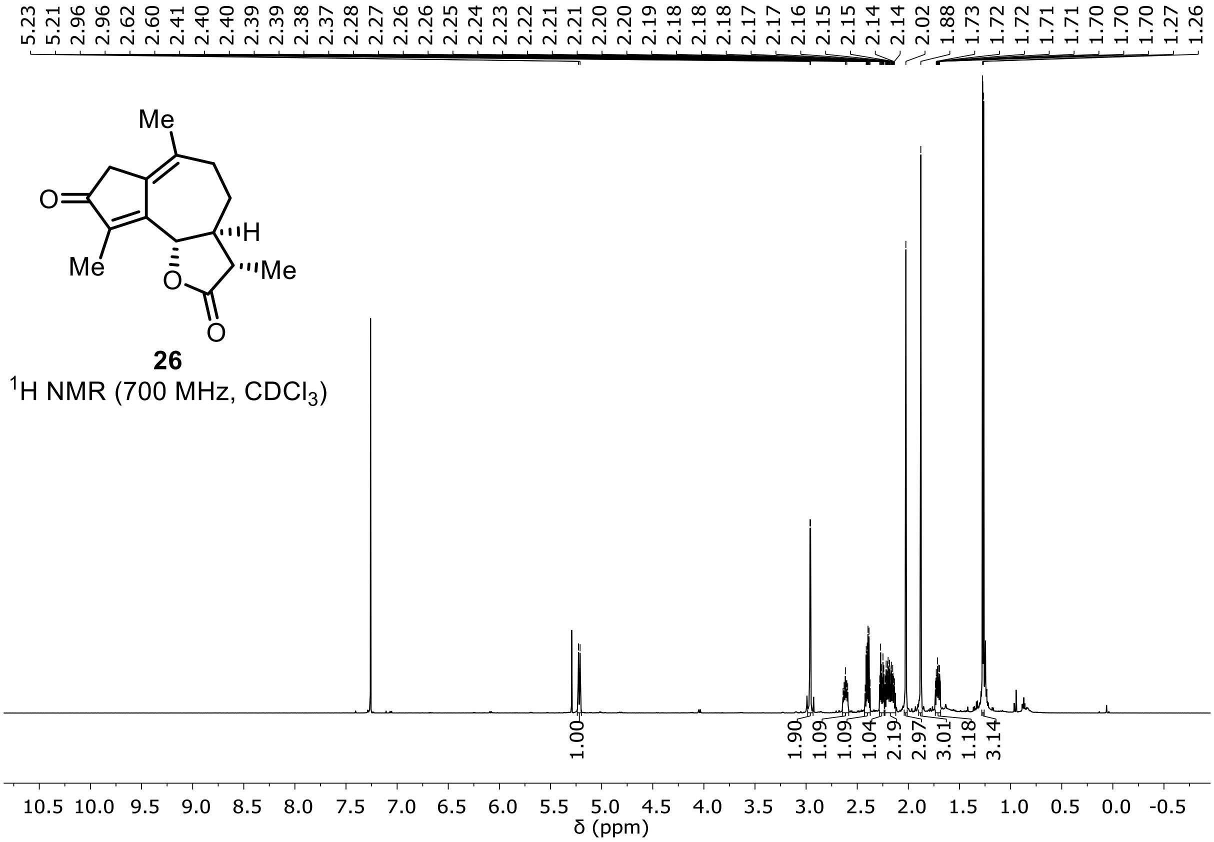

26

^{1}H NMR (700 MHz, $CDCl_3$)

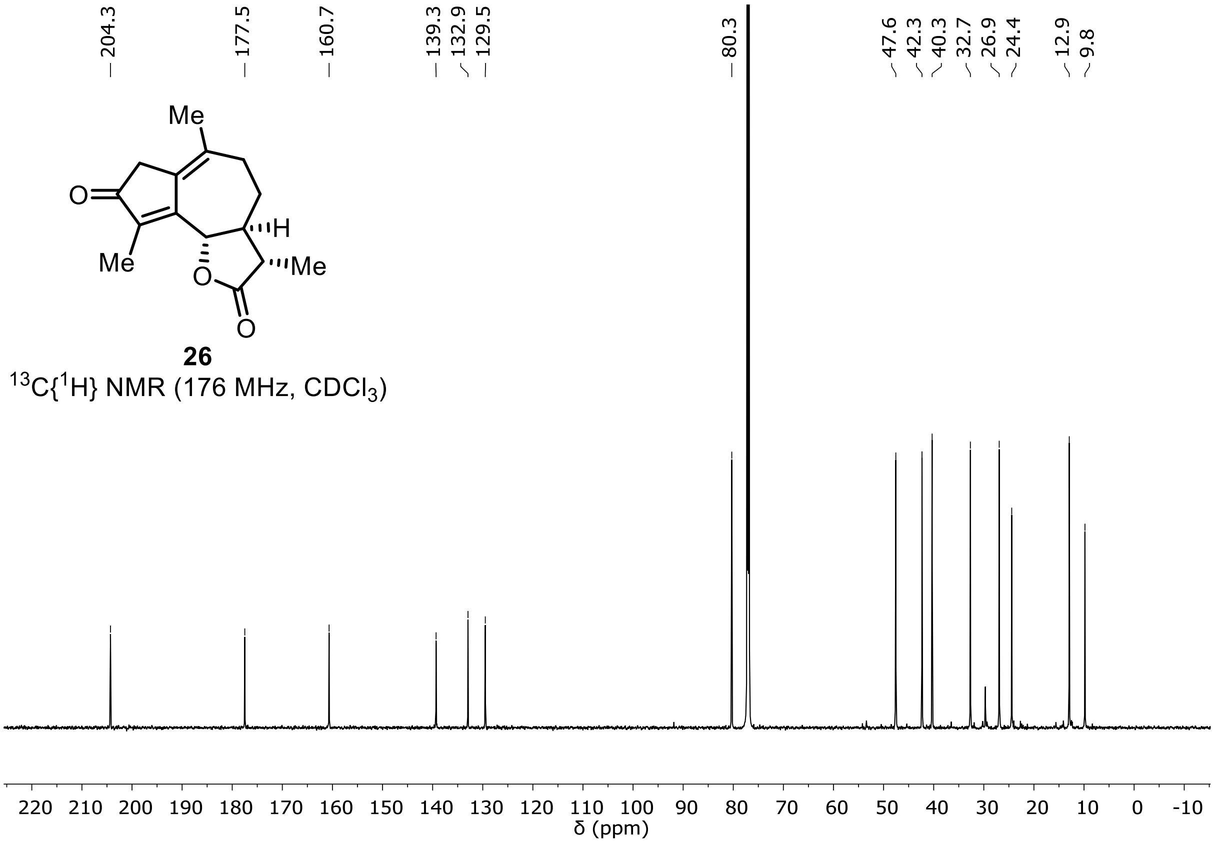

26

^{13}C{^{1}H} NMR (176 MHz, $CDCl_3$)

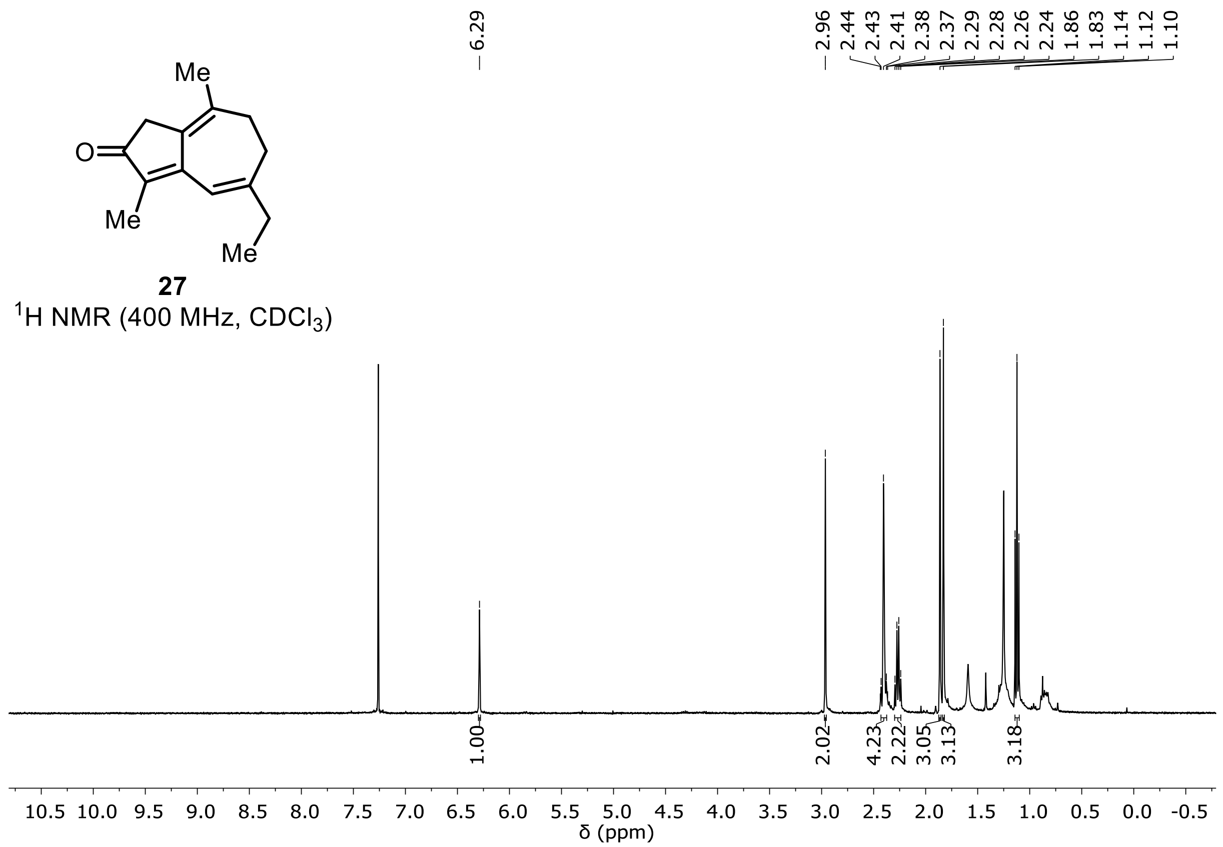

27

^{1}H NMR (400 MHz, $CDCl_3$)

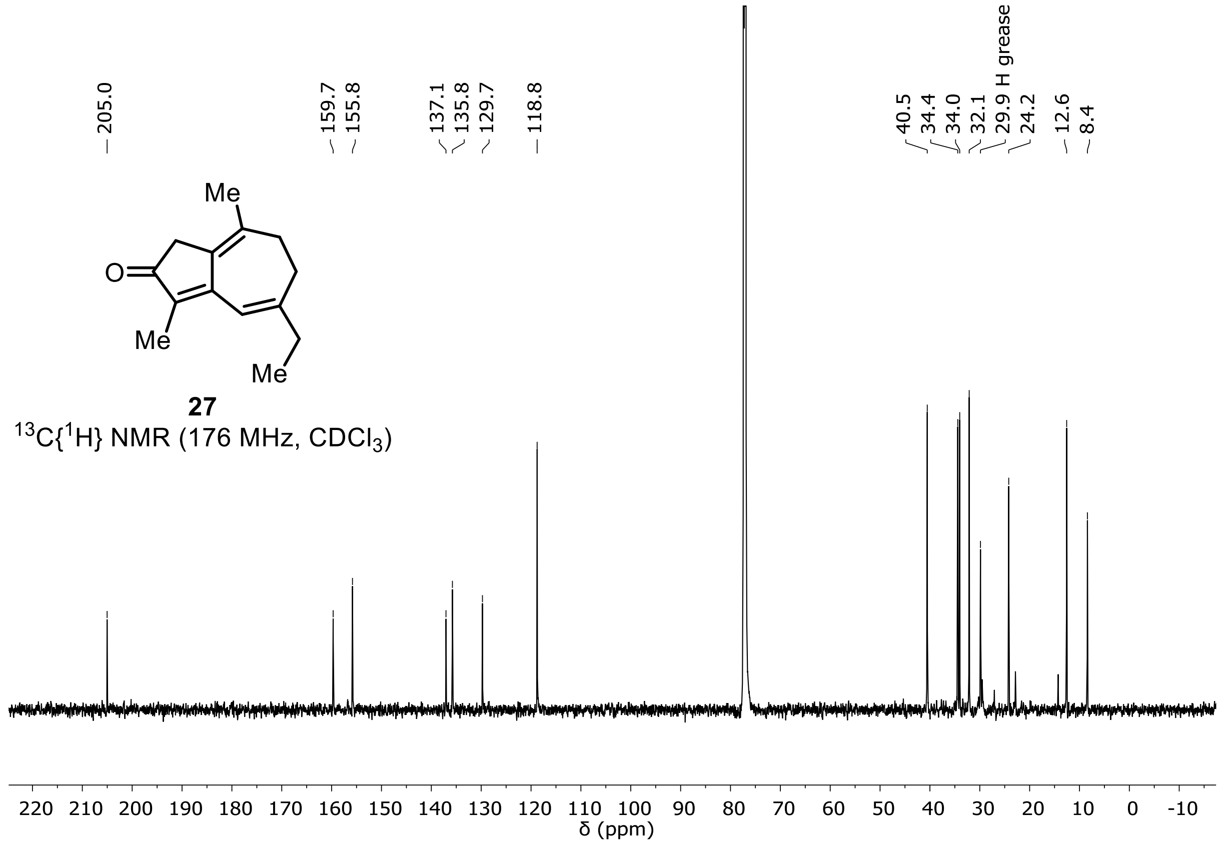

27

^{13}C{^{1}H} NMR (176 MHz, $CDCl_3$)

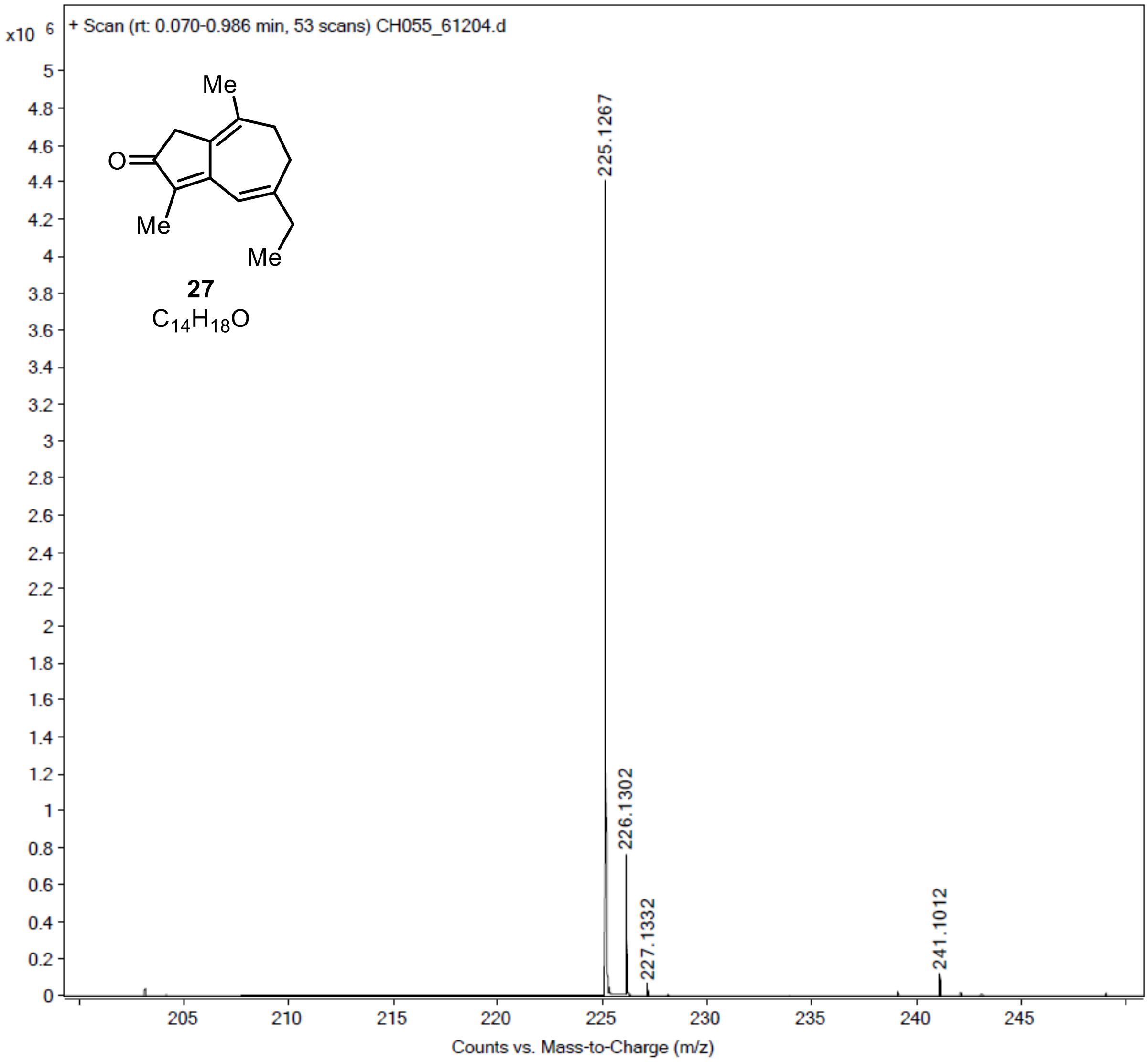

x10 6
+ Scan (rt: 0.070-0.986 min, 53 scans) CH055_61204.d
Me
O
Me
Me
27
C14H18O
225.1267
226.1302
227.1332
241.1012
Counts vs. Mass-to-Charge (m/z)

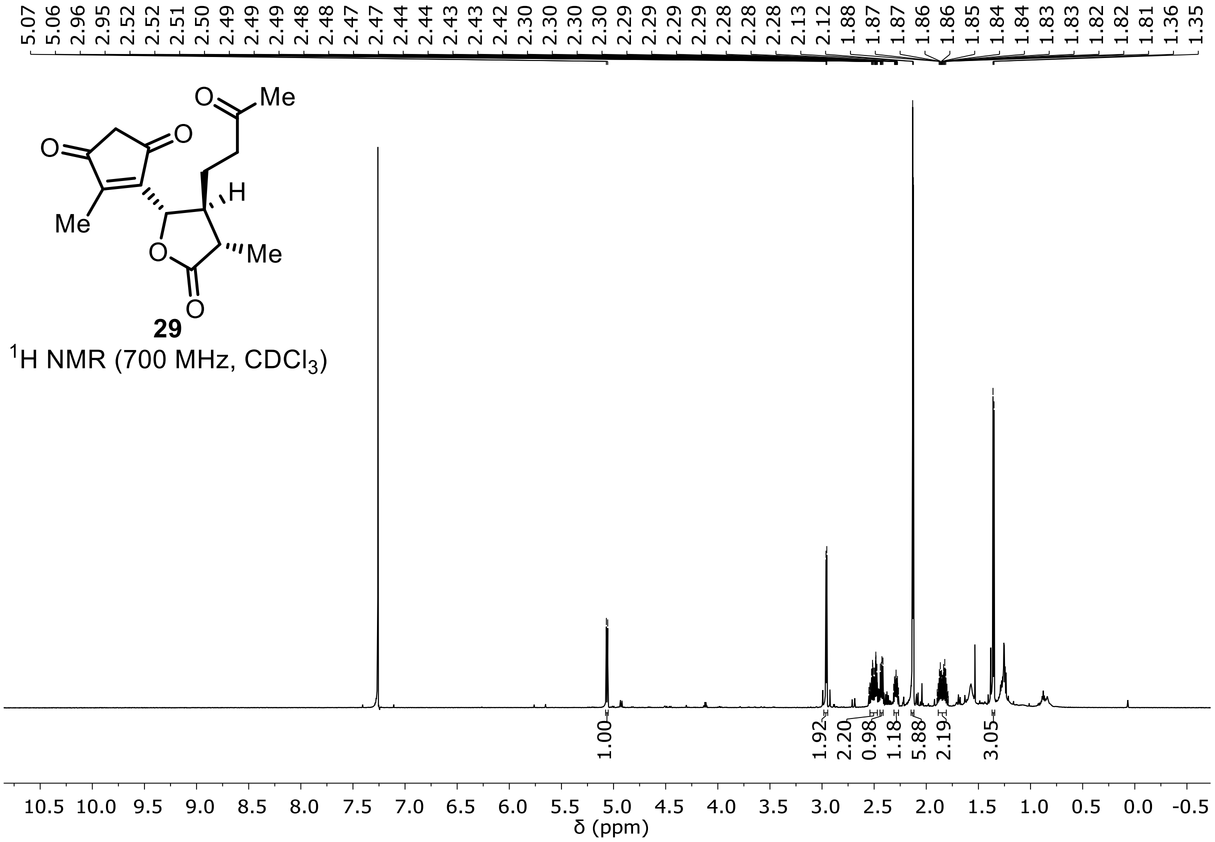
5.07 5.06 2.96 2.95 2.52 2.52 2.51 2.50 2.49 2.49 2.49 2.48 2.48 2.47 2.47 2.44 2.44 2.43 2.43 2.42 2.30 2.30 2.30 2.30 2.29 2.29 2.29 2.29 2.28 2.28 2.28 2.13 2.12 1.88 1.87 1.87 1.86 1.86 1.85 1.84 1.84 1.83 1.83 1.82 1.82 1.81 1.36 1.35
O
Me
O
O
H
Me
O
Me
O
29
^{1}H NMR (700 MHz, CDCl$_3$)
1.00
1.92
2.20
0.98
1.18
5.88
2.19
3.05
10.5 10.0 9.5 9.0 8.5 8.0 7.5 7.0 6.5 6.0 5.5 5.0 4.5 4.0 3.5 3.0 2.5 2.0 1.5 1.0 0.5 0.0 -0.5
δ (ppm)

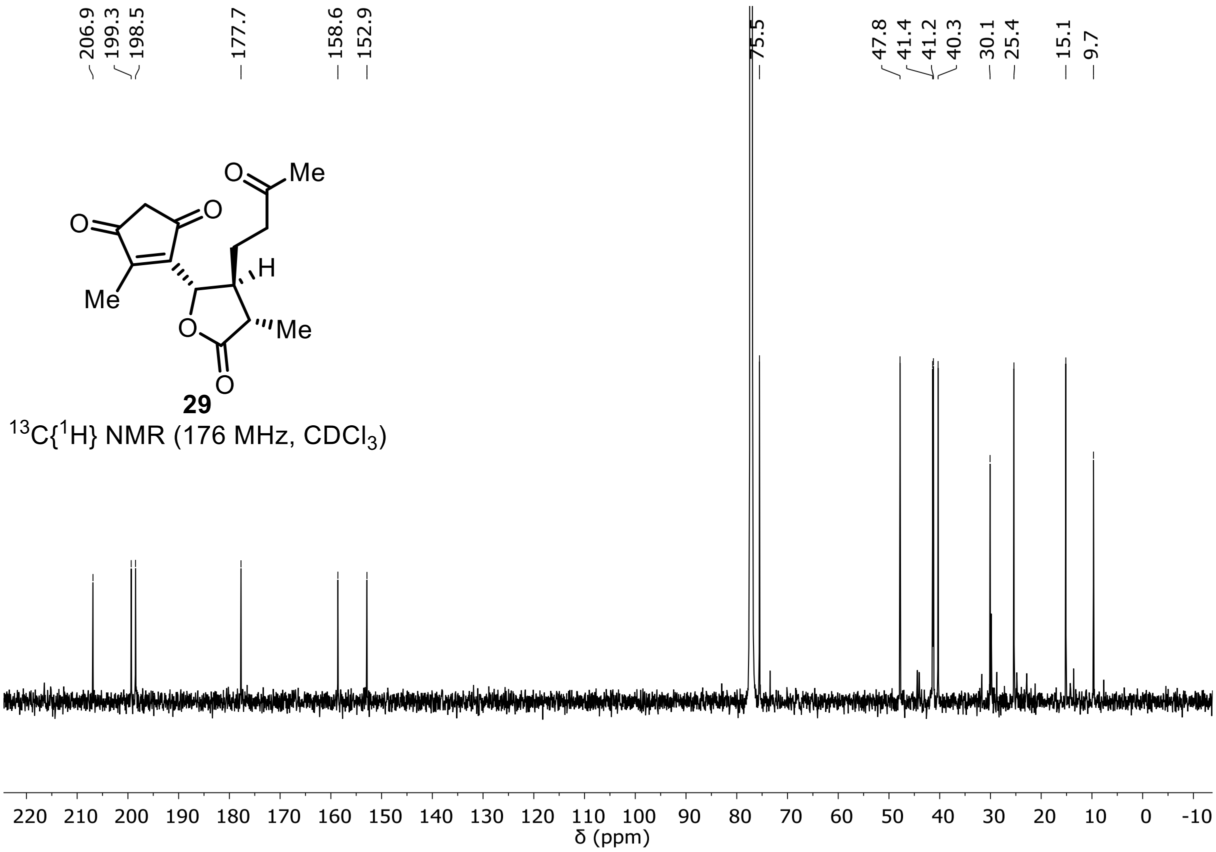
206.9 199.3 198.5 177.7 158.6 152.9 75.5 47.8 41.4 41.2 40.3 30.1 25.4 15.1 9.7
O
Me
O
O
H
Me
O
Me
O
29
^{13}C{^{1}H} NMR (176 MHz, CDCl$_3$)
220 210 200 190 180 170 160 150 140 130 120 110 100 90 80 70 60 50 40 30 20 10 0 -10
δ (ppm)

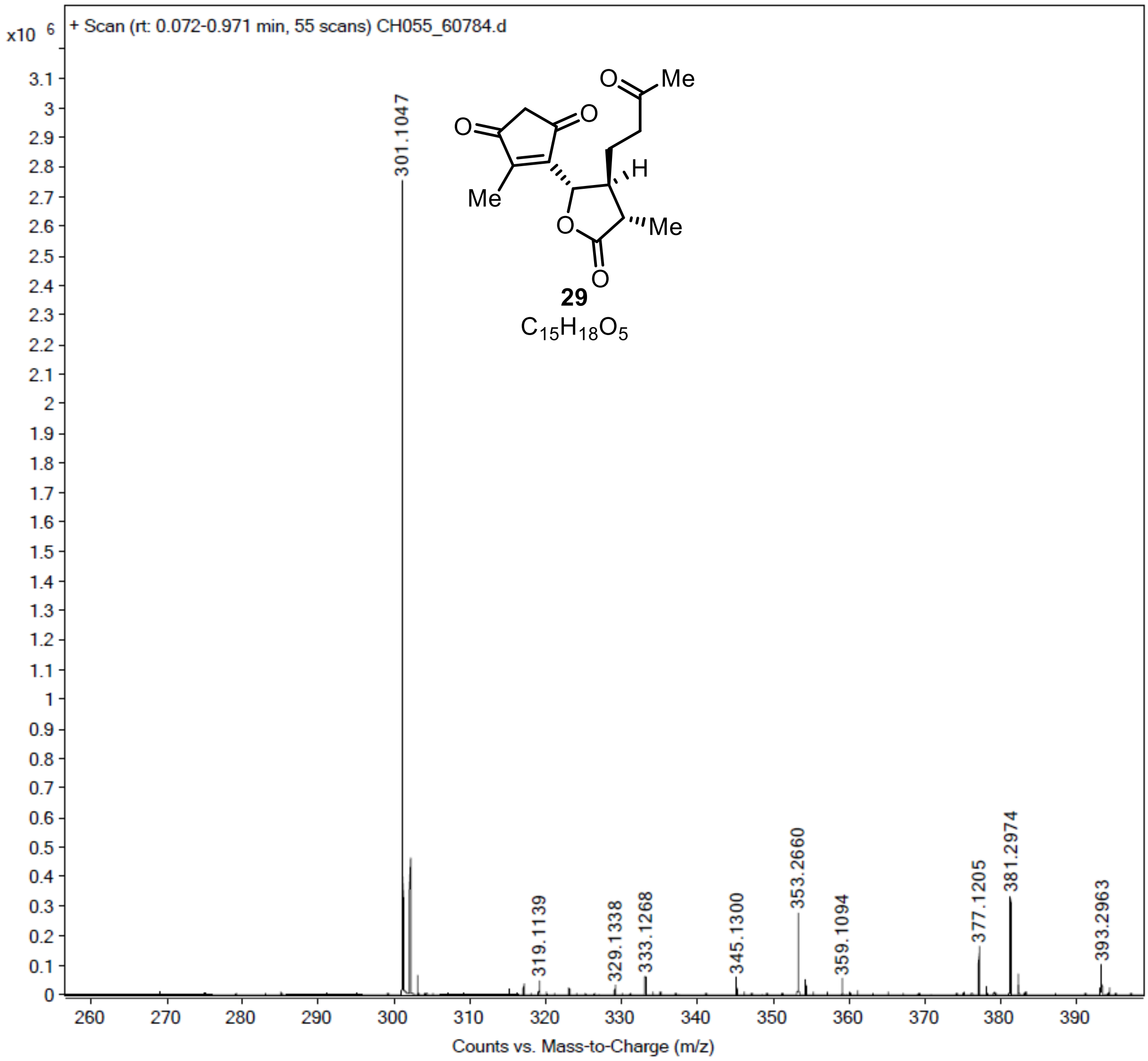

x10 6
+ Scan (rt: 0.072-0.971 min, 55 scans) CH055_60784.d
301.1047
319.1139
329.1338
333.1268
345.1300
353.2660
359.1094
377.1205
381.2974
393.2963
29
$C_{15}H_{18}O_5$
Counts vs. Mass-to-Charge (m/z)

30

^{1}H NMR (700 MHz, $CDCl_3$)

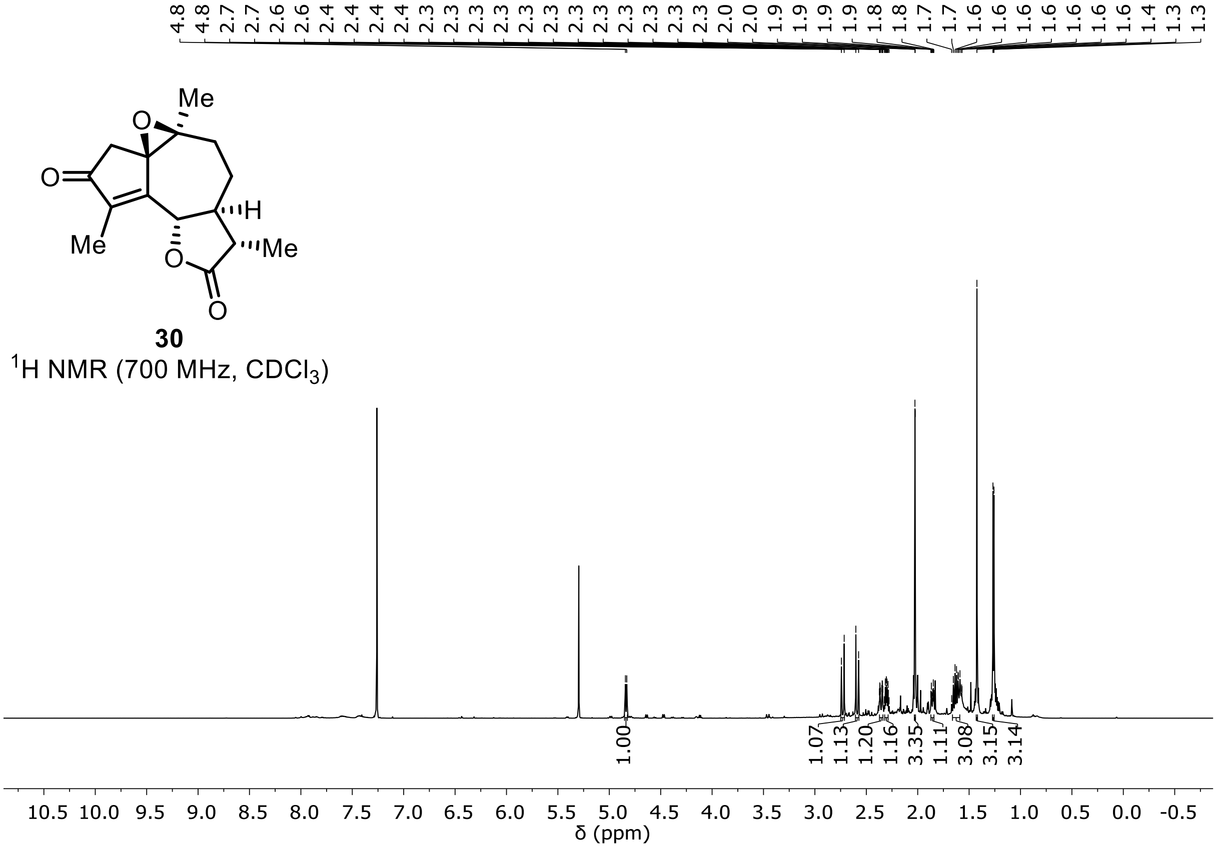

30

$^{13}C\{^{1}H\}$ NMR (176 MHz, $CDCl_3$)

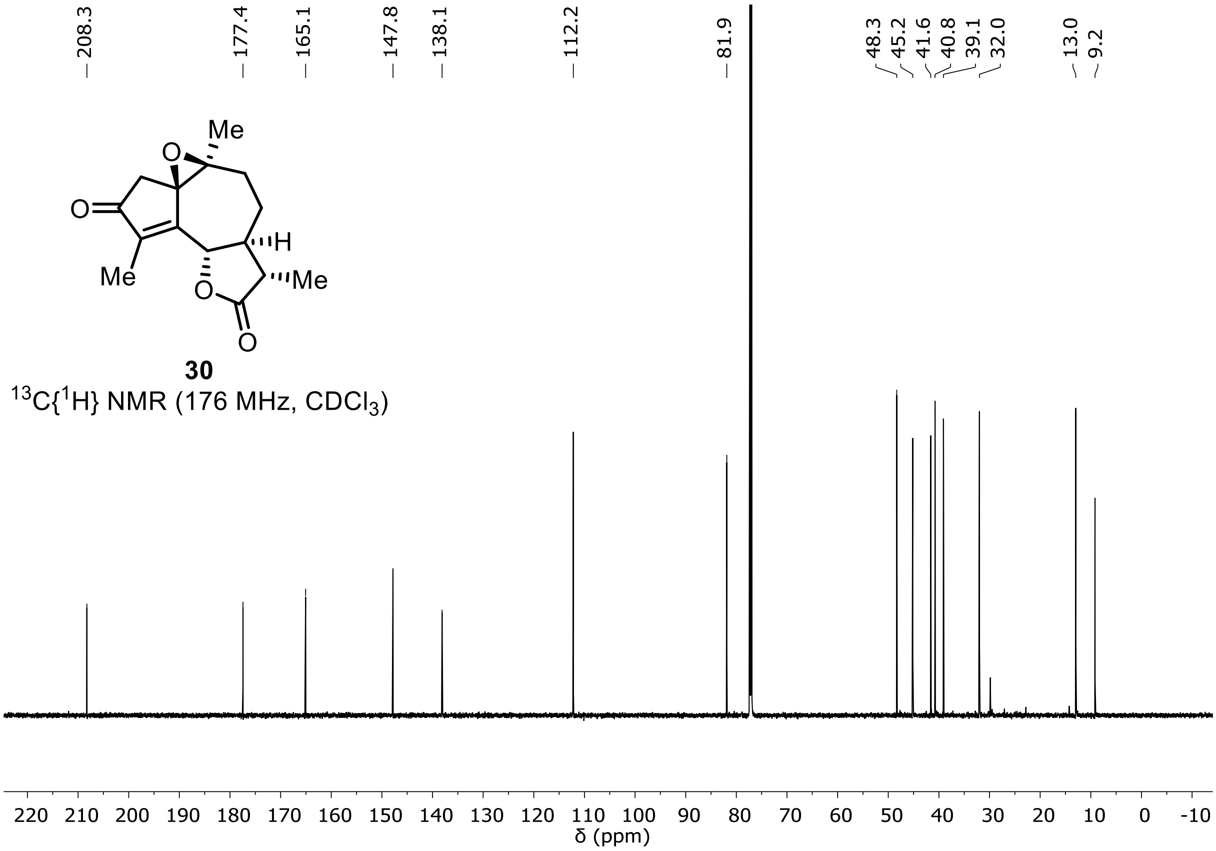

r

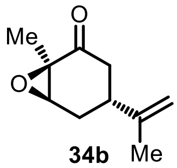

34b

^{1}H NMR (400 MHz, $CDCl_3$)

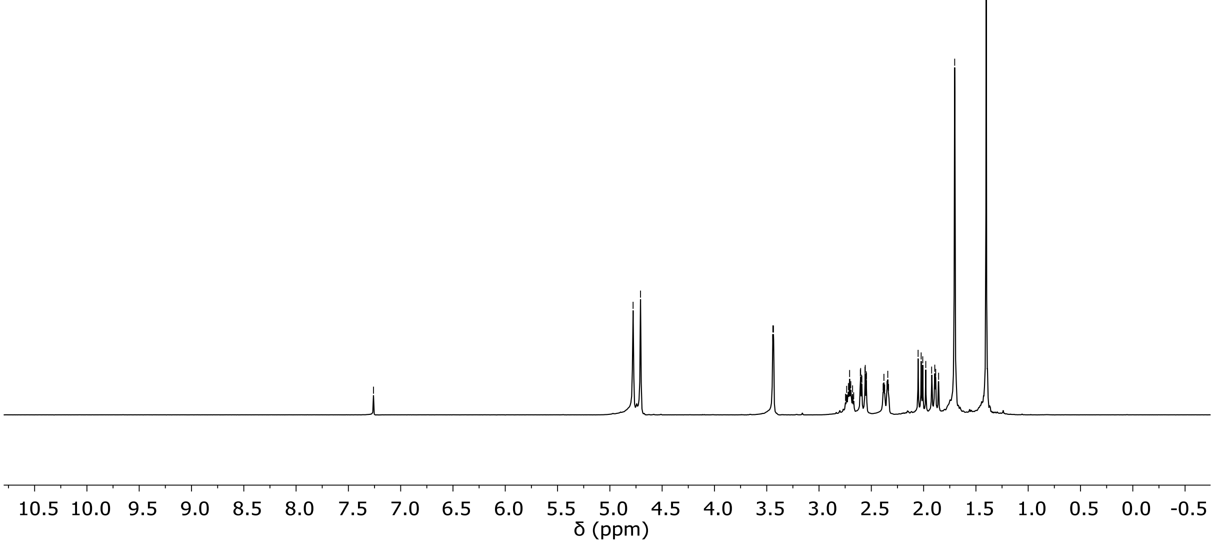

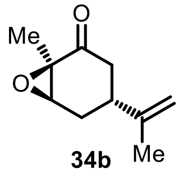

34b

^{13}C {^{1}H} NMR (151 MHz, $CDCl_3$)

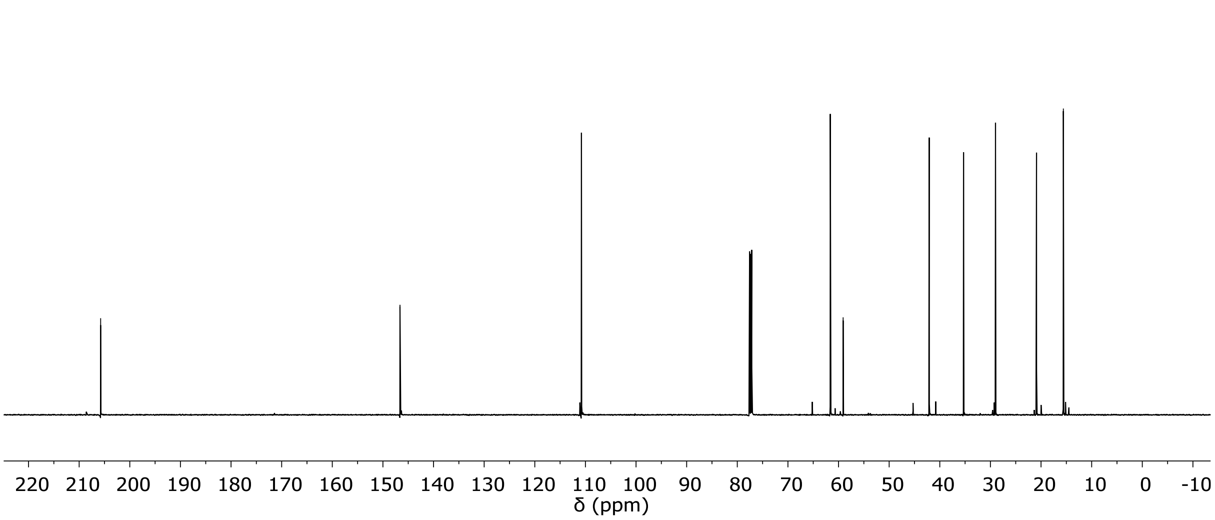

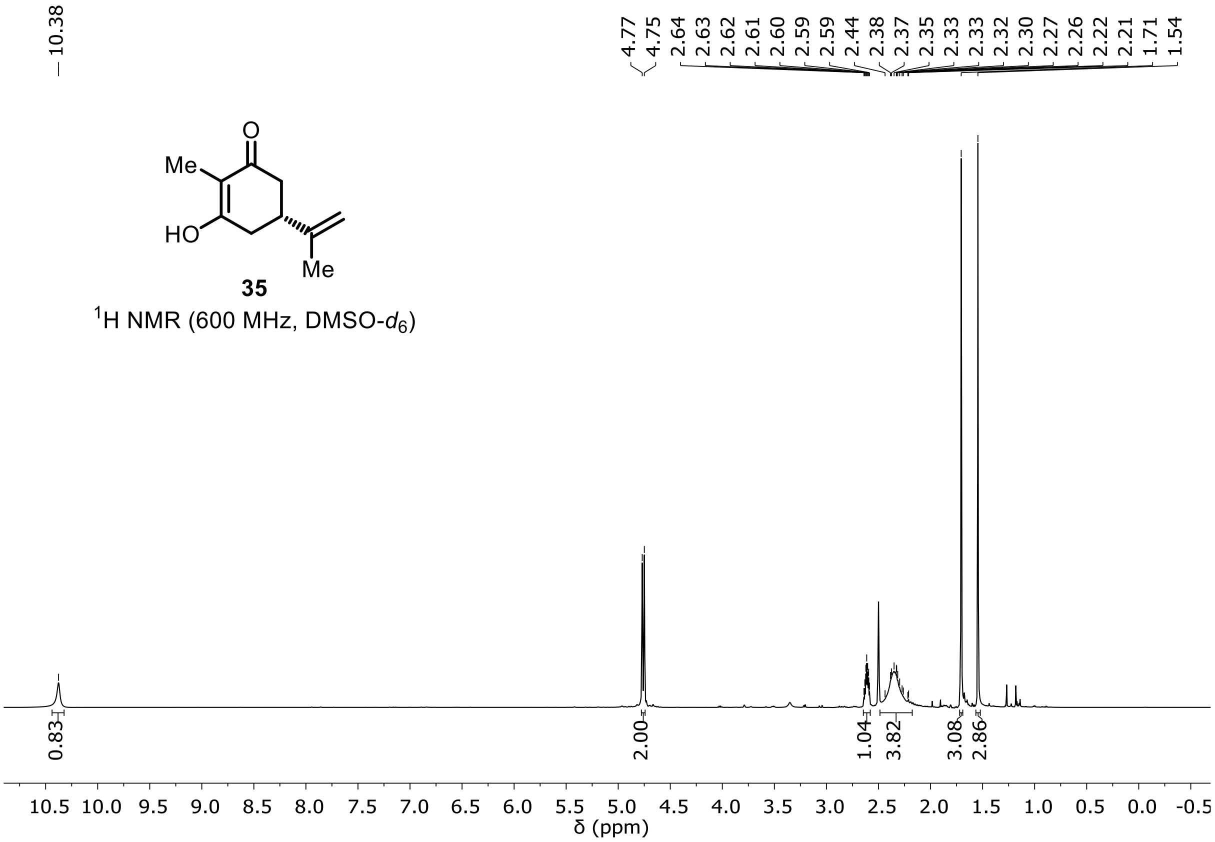
35
^{1}H NMR (600 MHz, DMSO-d_6)
10.38
4.77 4.75 2.64 2.63 2.62 2.61 2.60 2.59 2.59 2.44 2.38 2.37 2.35 2.33 2.33 2.32 2.30 2.27 2.26 2.22 2.21 1.71 1.54
0.83 2.00 1.04 3.82 3.08 2.86
10.5 10.0 9.5 9.0 8.5 8.0 7.5 7.0 6.5 6.0 5.5 5.0 4.5 4.0 3.5 3.0 2.5 2.0 1.5 1.0 0.5 0.0 -0.5
δ (ppm)

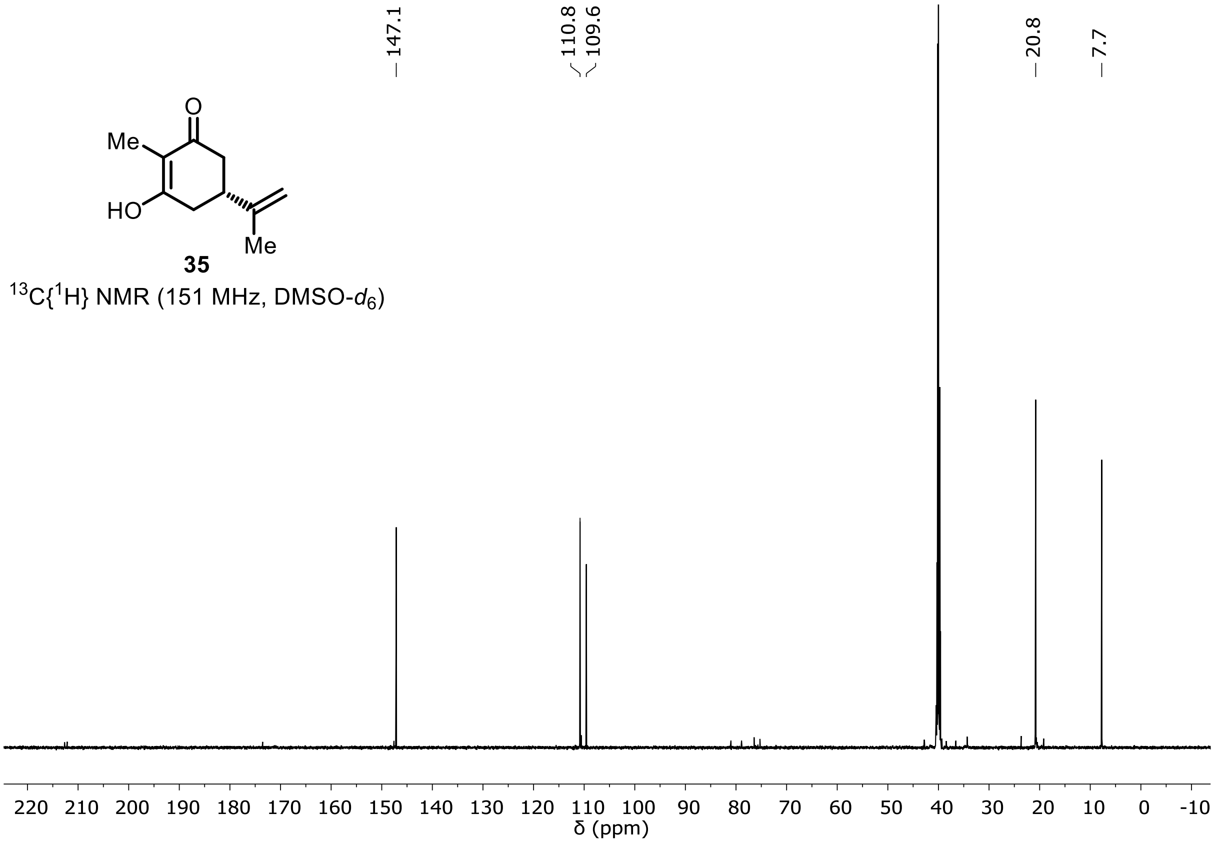
35
^{13}C{^{1}H} NMR (151 MHz, DMSO-d_6)
147.1
110.8 109.6
20.8
7.7
220 210 200 190 180 170 160 150 140 130 120 110 100 90 80 70 60 50 40 30 20 10 0 -10
δ (ppm)

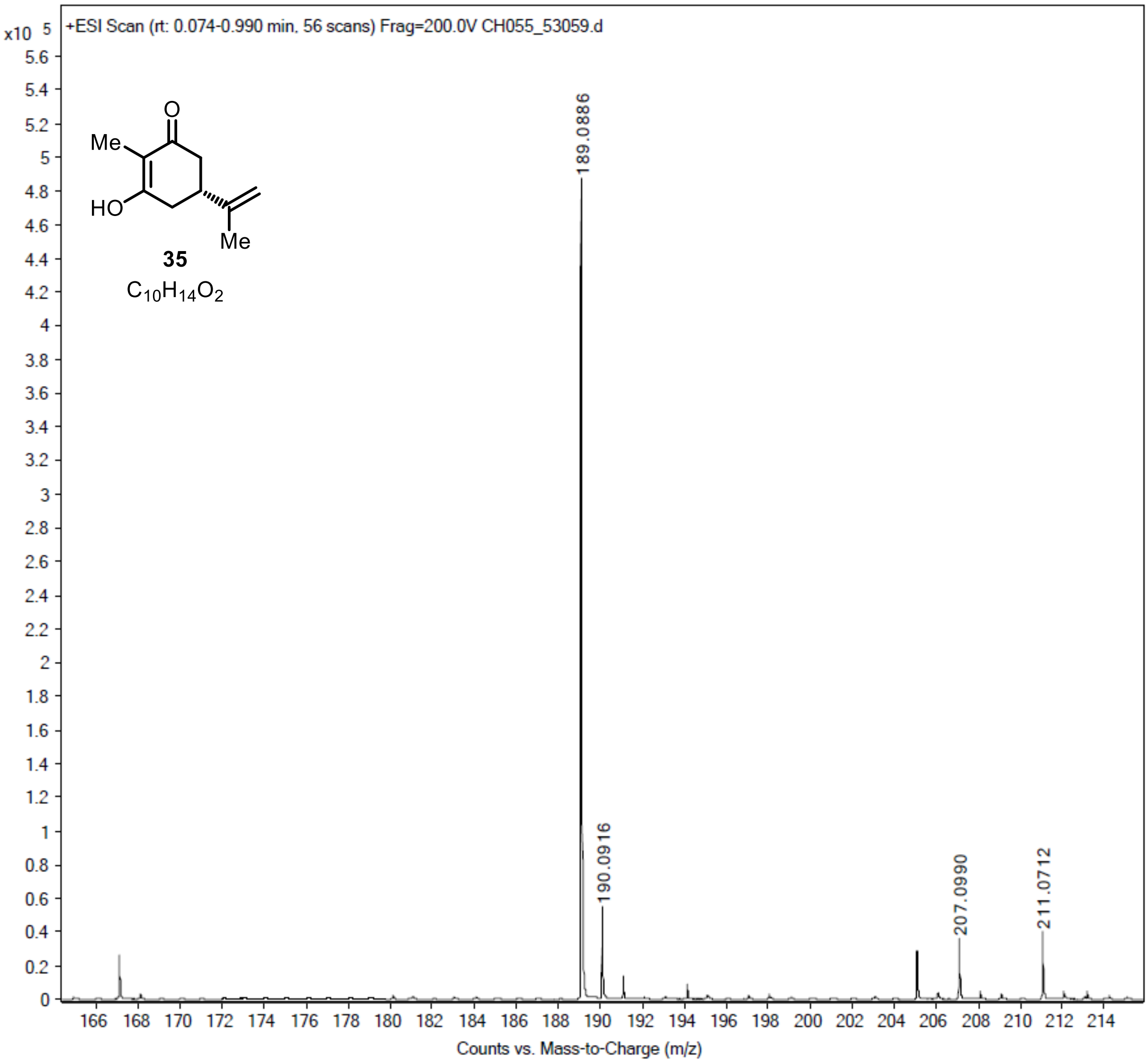

x10 5
+ESI Scan (rt: 0.074-0.990 min, 56 scans) Frag=200.0V CH055_53059.d
Me
O
HO
Me
35
$C_{10}H_{14}O_2$
189.0886
190.0916
207.0990
211.0712
Counts vs. Mass-to-Charge (m/z)

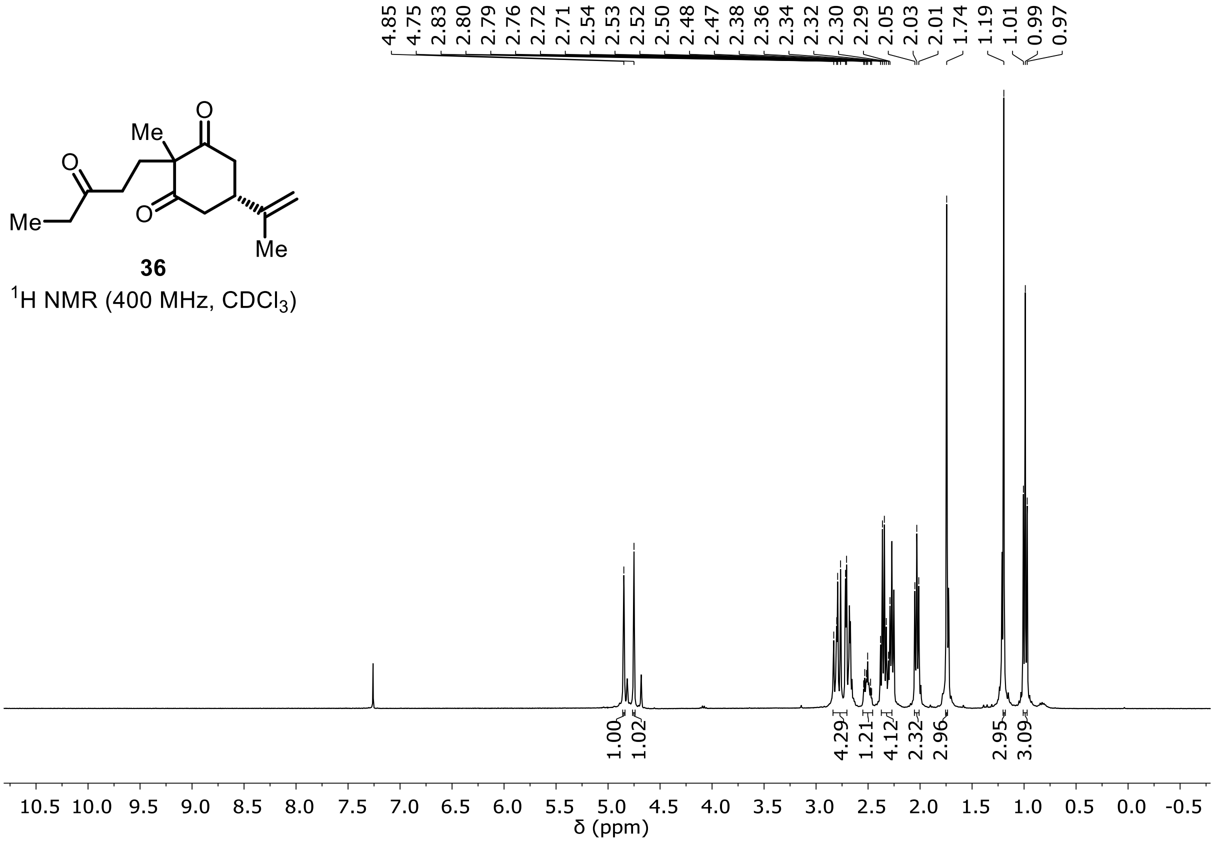
4.85 4.75 2.83 2.80 2.79 2.76 2.72 2.71 2.54 2.53 2.52 2.50 2.48 2.47 2.38 2.36 2.34 2.32 2.30 2.29 2.05 2.03 2.01 1.74 1.19 1.01 0.99 0.97
Me
O
O
O
Me
Me
36
^{1}H NMR (400 MHz, $CDCl_3$)
1.00 1.02 4.29 1.21 4.12 2.32 2.96 2.95 3.09
10.5 10.0 9.5 9.0 8.5 8.0 7.5 7.0 6.5 6.0 5.5 5.0 4.5 4.0 3.5 3.0 2.5 2.0 1.5 1.0 0.5 0.0 -0.5
δ (ppm)

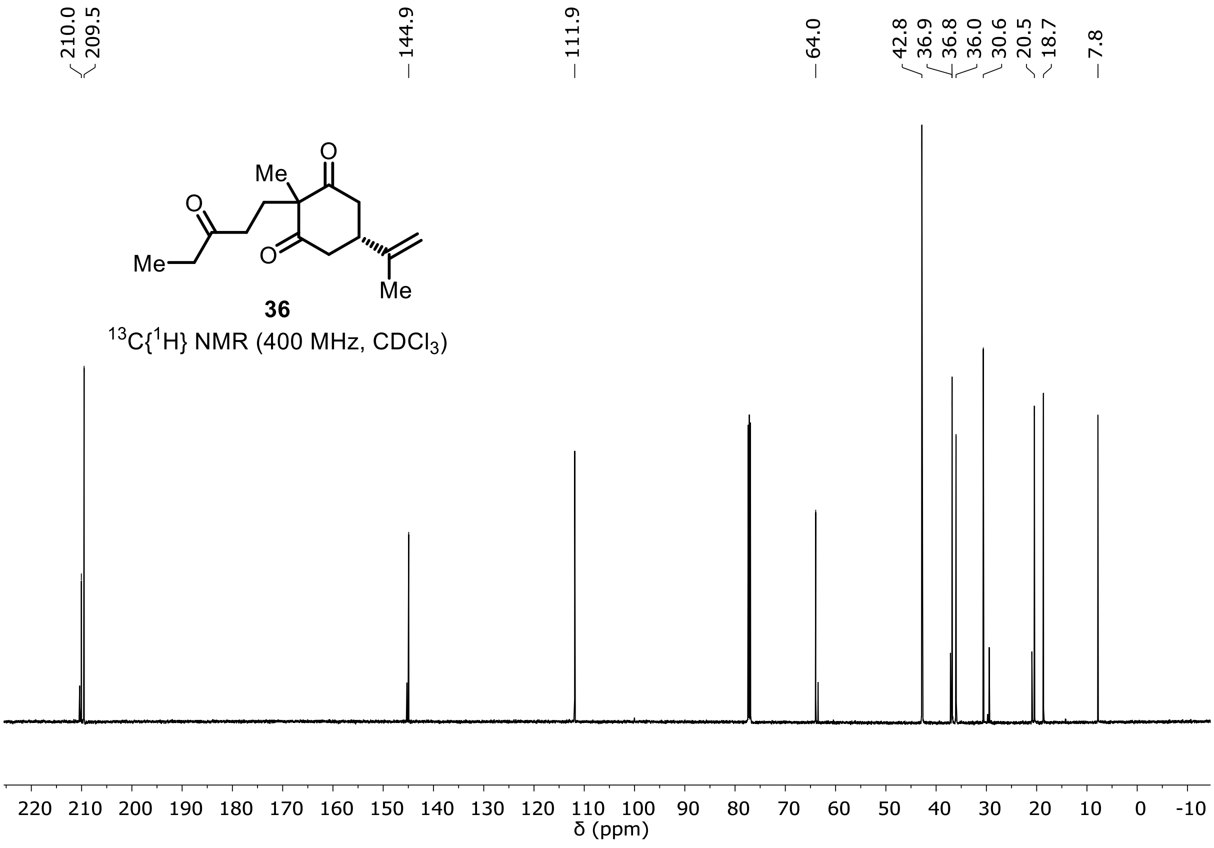
210.0 209.5 144.9 111.9 64.0 42.8 36.9 36.8 36.0 30.6 20.5 18.7 7.8
Me
O
O
O
Me
Me
36
^{13}C{^{1}H} NMR (400 MHz, $CDCl_3$)
220 210 200 190 180 170 160 150 140 130 120 110 100 90 80 70 60 50 40 30 20 10 0 -10
δ (ppm)

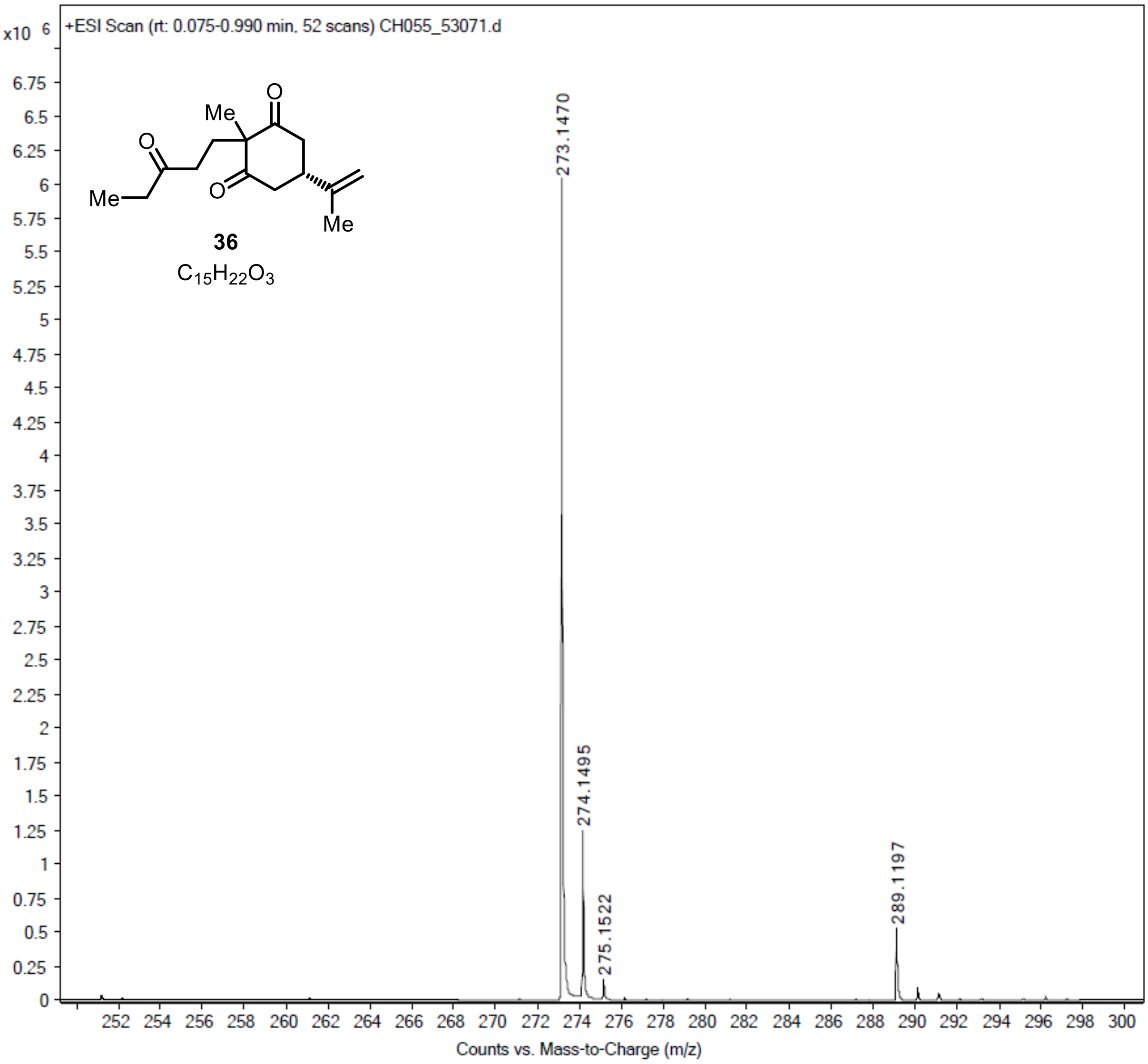
x10 6
+ESI Scan (rt: 0.075-0.990 min, 52 scans) CH055_53071.d
36
C15H22O3
273.1470
274.1495
275.1522
289.1197
Counts vs. Mass-to-Charge (m/z)

32b

^{1}H NMR (600 MHz, $CDCl_3$)

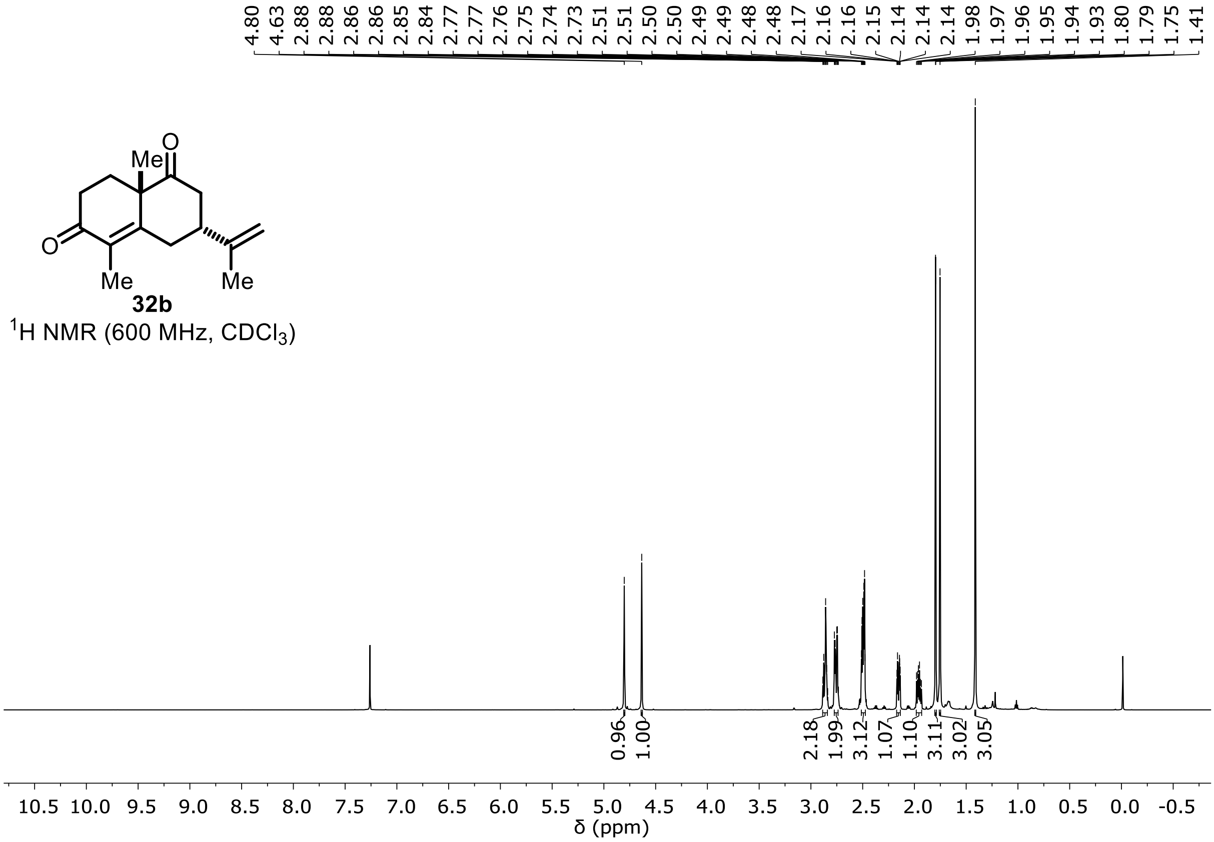

32b

$^{13}C\{^1H\}$ NMR (151 MHz, $CDCl_3$)

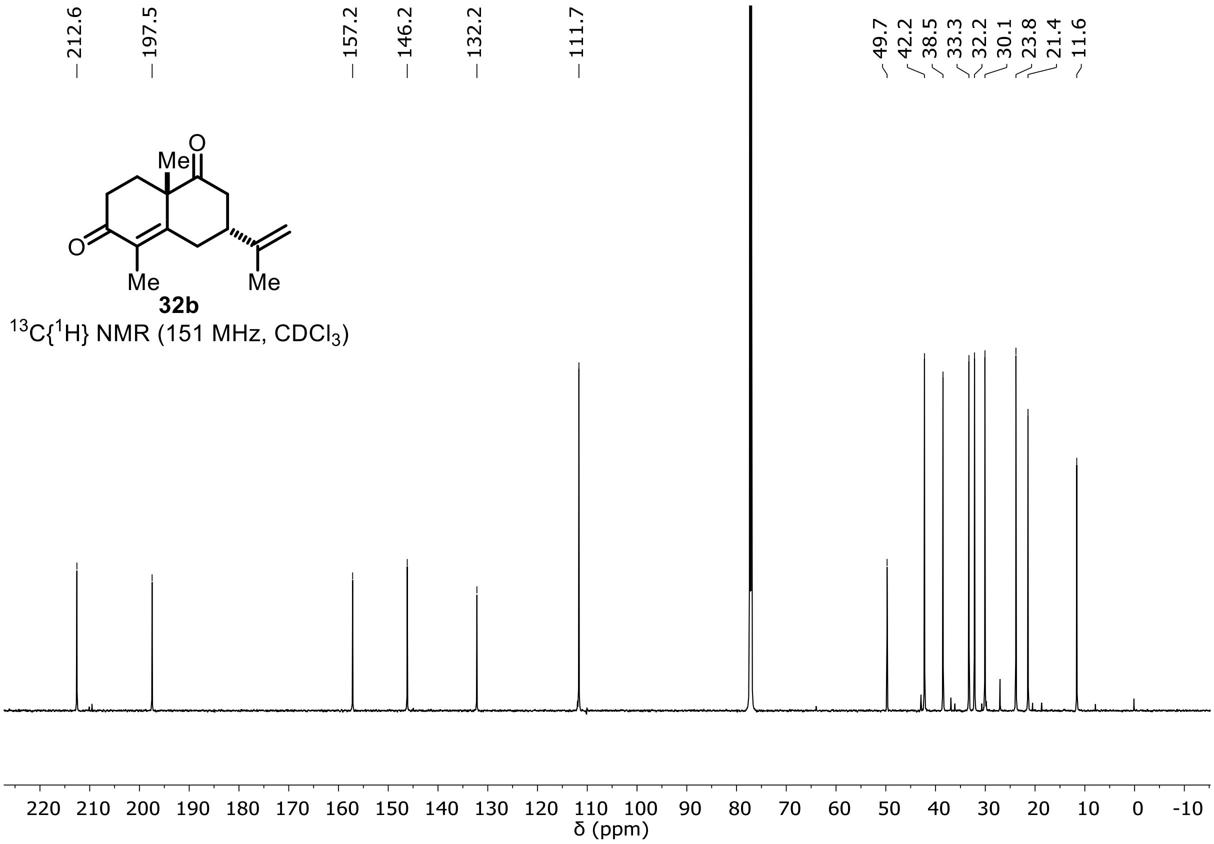

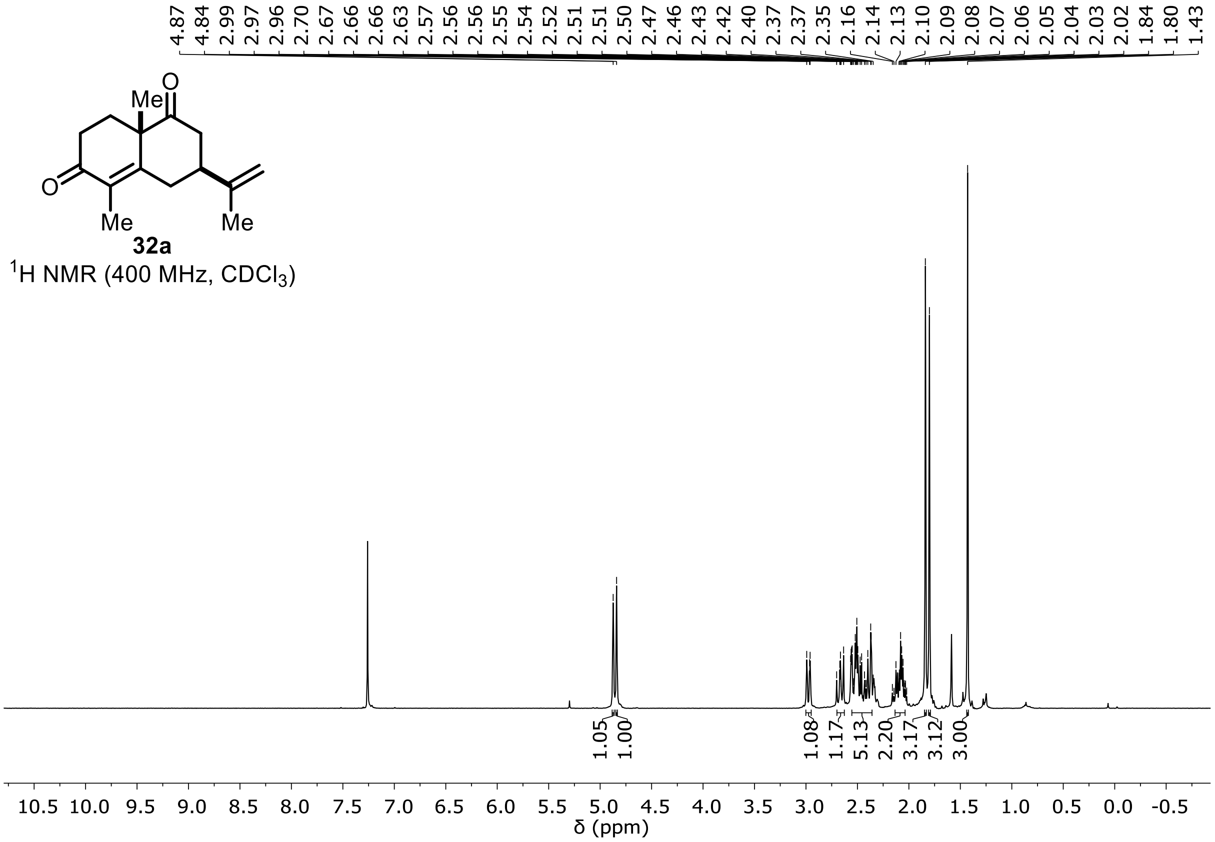

32a

^{1}H NMR (400 MHz, $CDCl_3$)

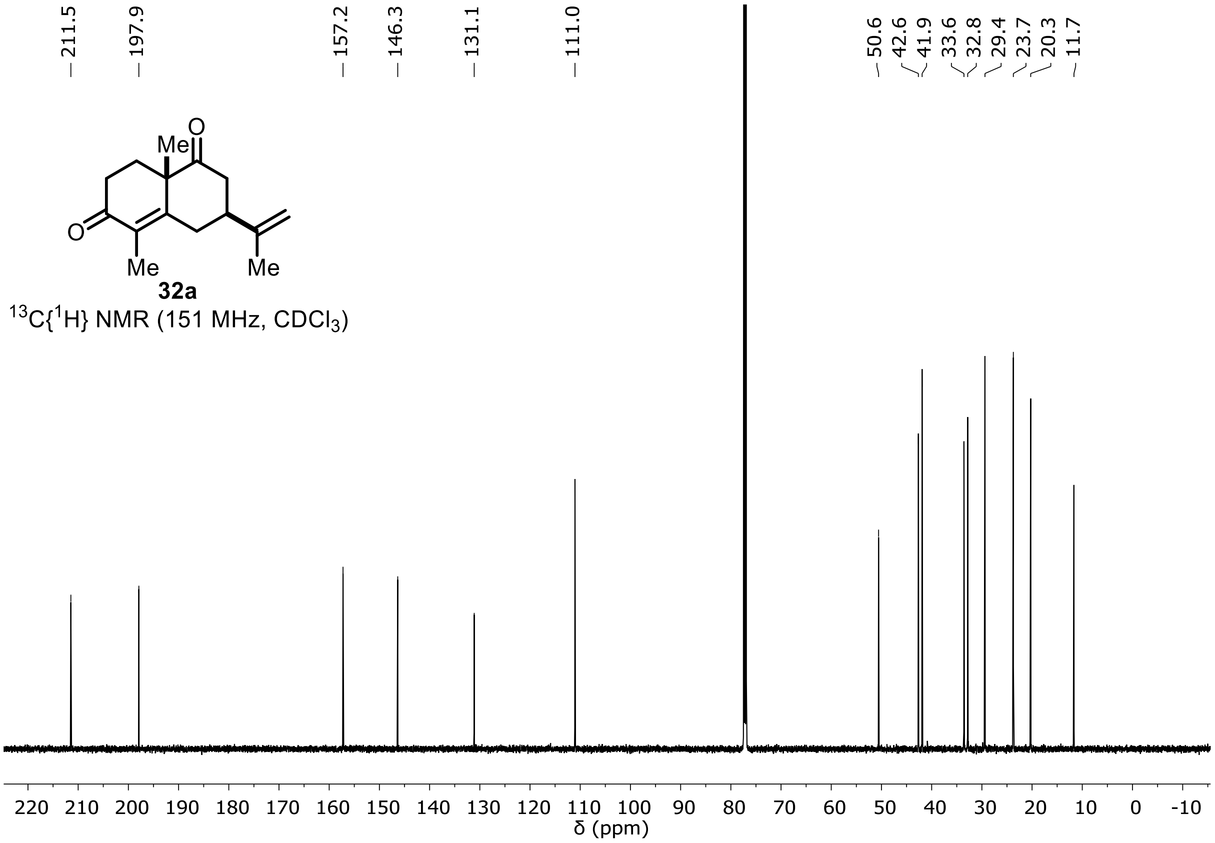

32a

$^{13}C\{^1H\}$ NMR (151 MHz, $CDCl_3$)

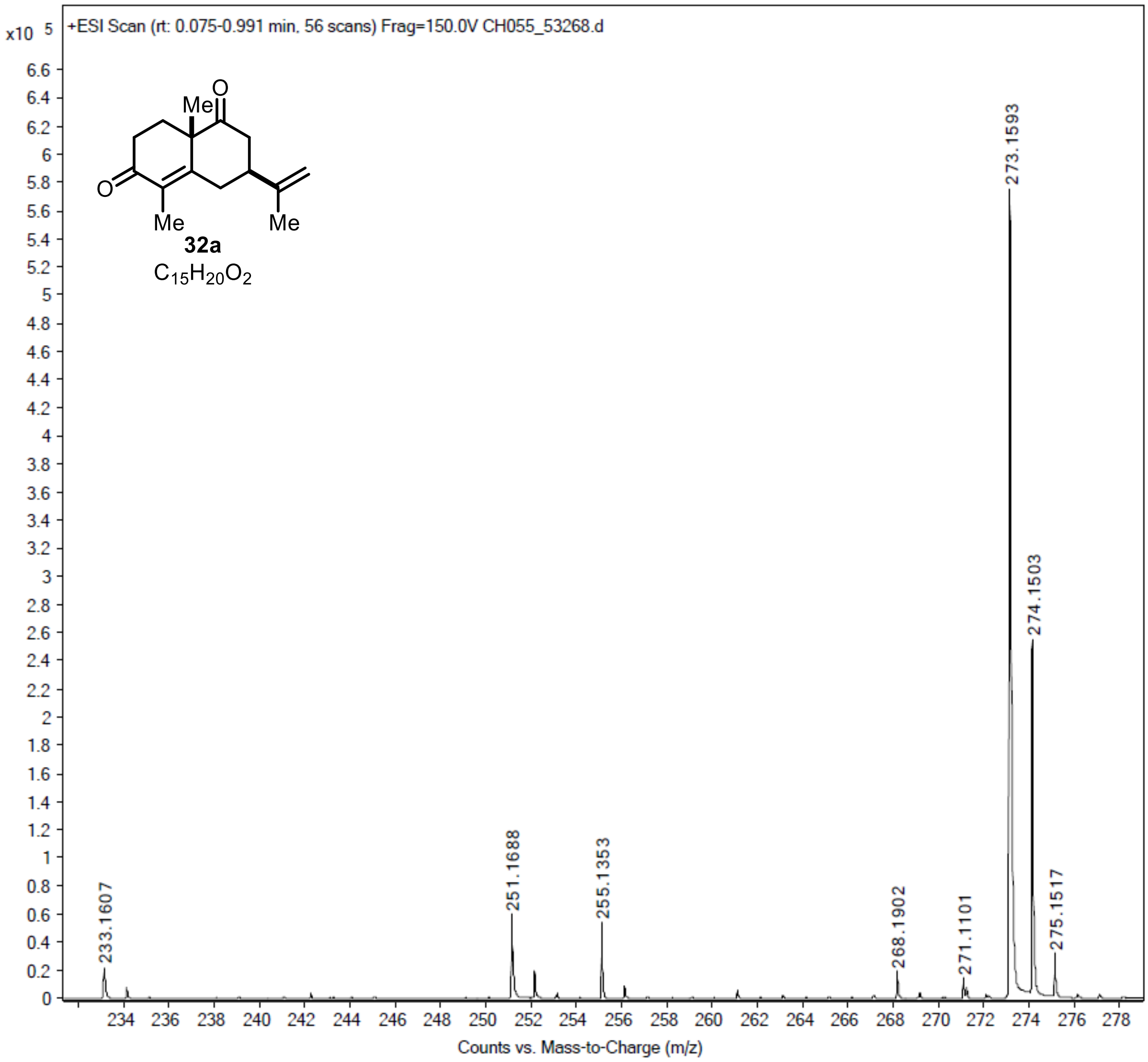
x10 5
+ESI Scan (rt: 0.075-0.991 min, 56 scans) Frag=150.0V CH055_53268.d
32a
C15H20O2
Me
O
Me
Me
233.1607
251.1688
255.1353
268.1902
271.1101
273.1593
274.1503
275.1517
Counts vs. Mass-to-Charge (m/z)

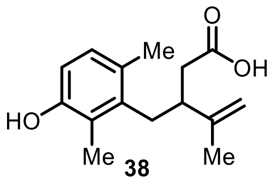

38

^{1}H NMR (600 MHz, $CDCl_3$)

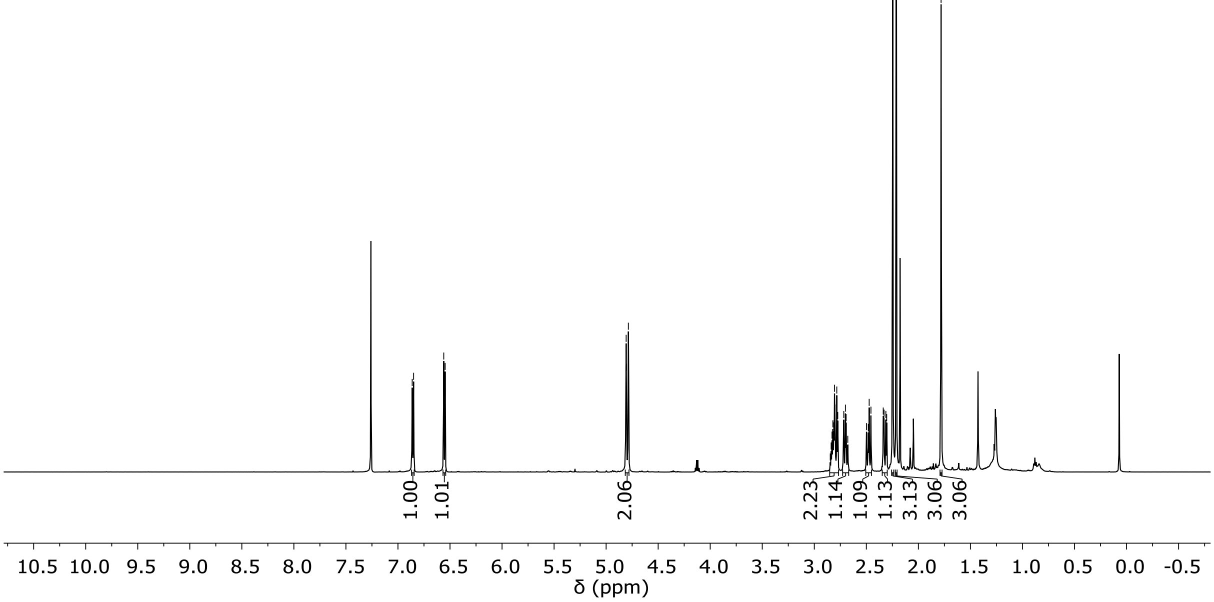

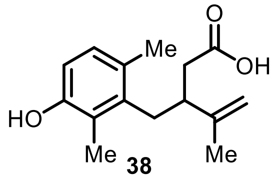

38

^{13}C{^{1}H} NMR (151 MHz, $CDCl_3$)

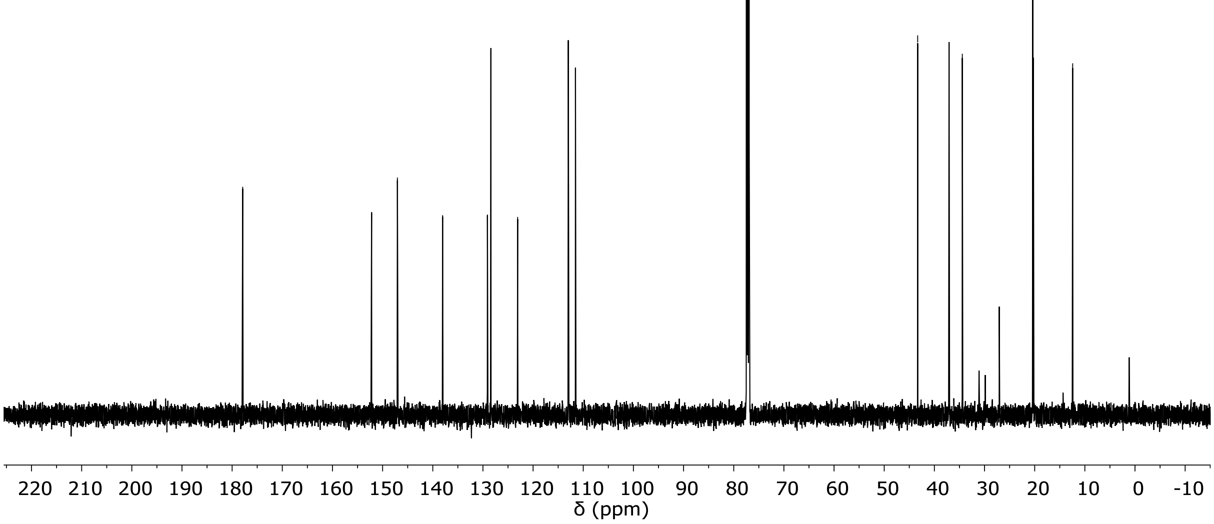

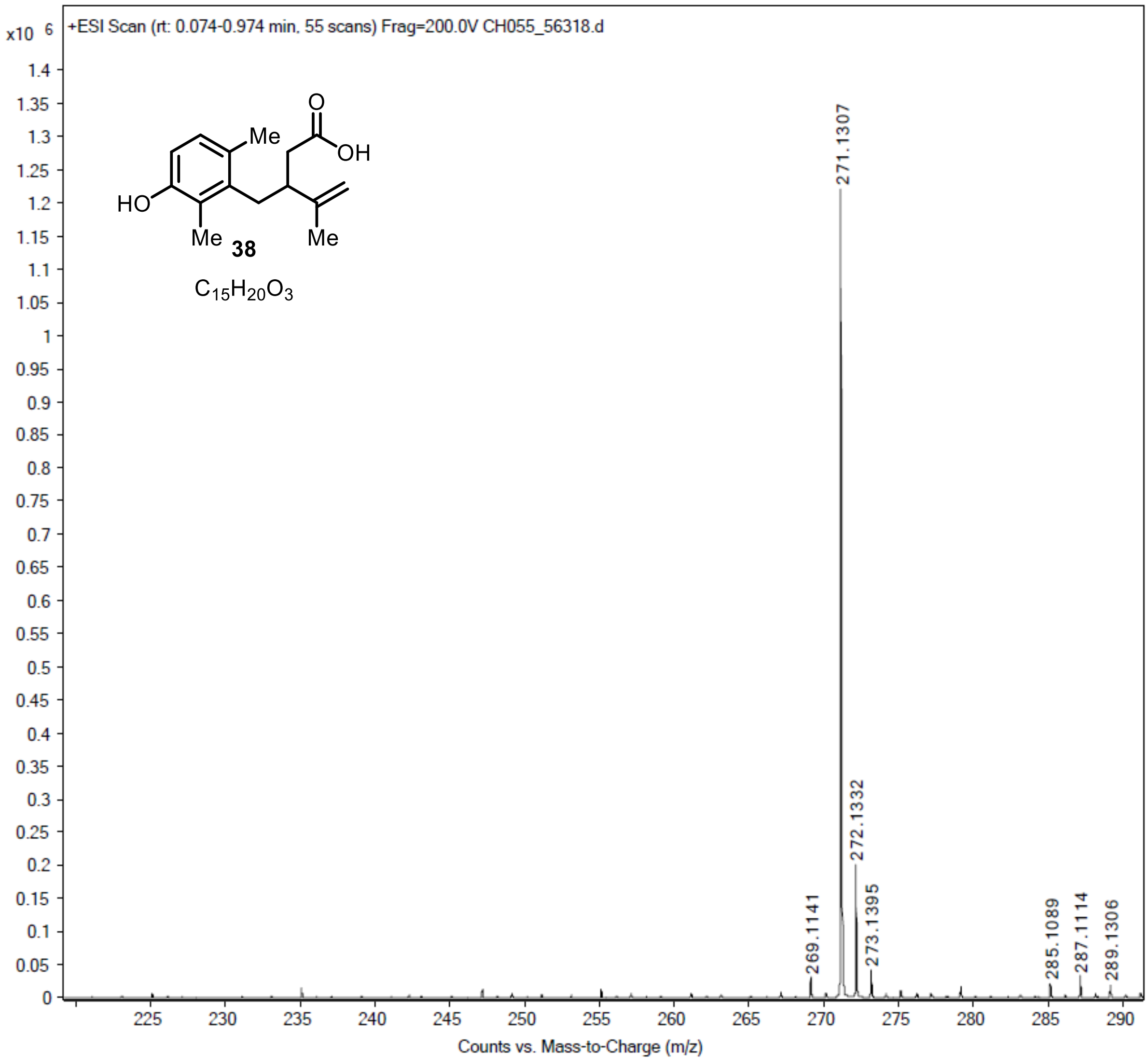

x10 6
+ESI Scan (rt: 0.074-0.974 min, 55 scans) Frag=200.0V CH055_56318.d
Me
O
OH
HO
Me
38
Me
C15H20O3
271.1307
272.1332
273.1395
269.1141
285.1089
287.1114
289.1306
Counts vs. Mass-to-Charge (m/z)

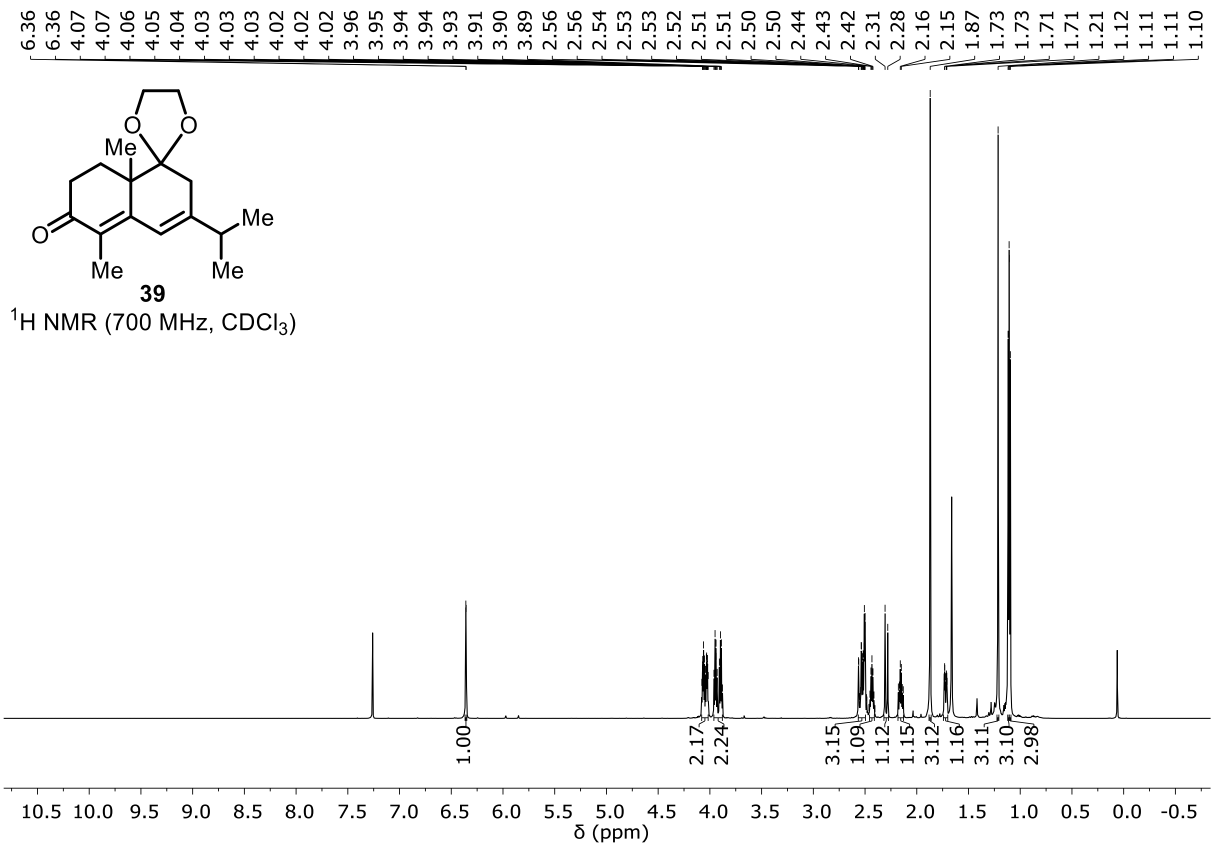
6.36 6.36 4.07 4.07 4.06 4.05 4.04 4.03 4.03 4.03 4.02 4.02 4.02 3.96 3.95 3.94 3.94 3.93 3.91 3.90 3.89 2.56 2.56 2.54 2.53 2.53 2.52 2.51 2.51 2.50 2.50 2.44 2.43 2.42 2.31 2.28 2.16 2.15 1.87 1.73 1.73 1.71 1.71 1.21 1.12 1.11 1.11 1.10
Me
O
O
O
Me
Me
Me
39
1H NMR (700 MHz, CDCl3)
1.00
2.17
2.24
3.15
1.09
1.12
1.15
3.12
1.16
3.11
3.10
2.98
10.5 10.0 9.5 9.0 8.5 8.0 7.5 7.0 6.5 6.0 5.5 5.0 4.5 4.0 3.5 3.0 2.5 2.0 1.5 1.0 0.5 0.0 -0.5
δ (ppm)

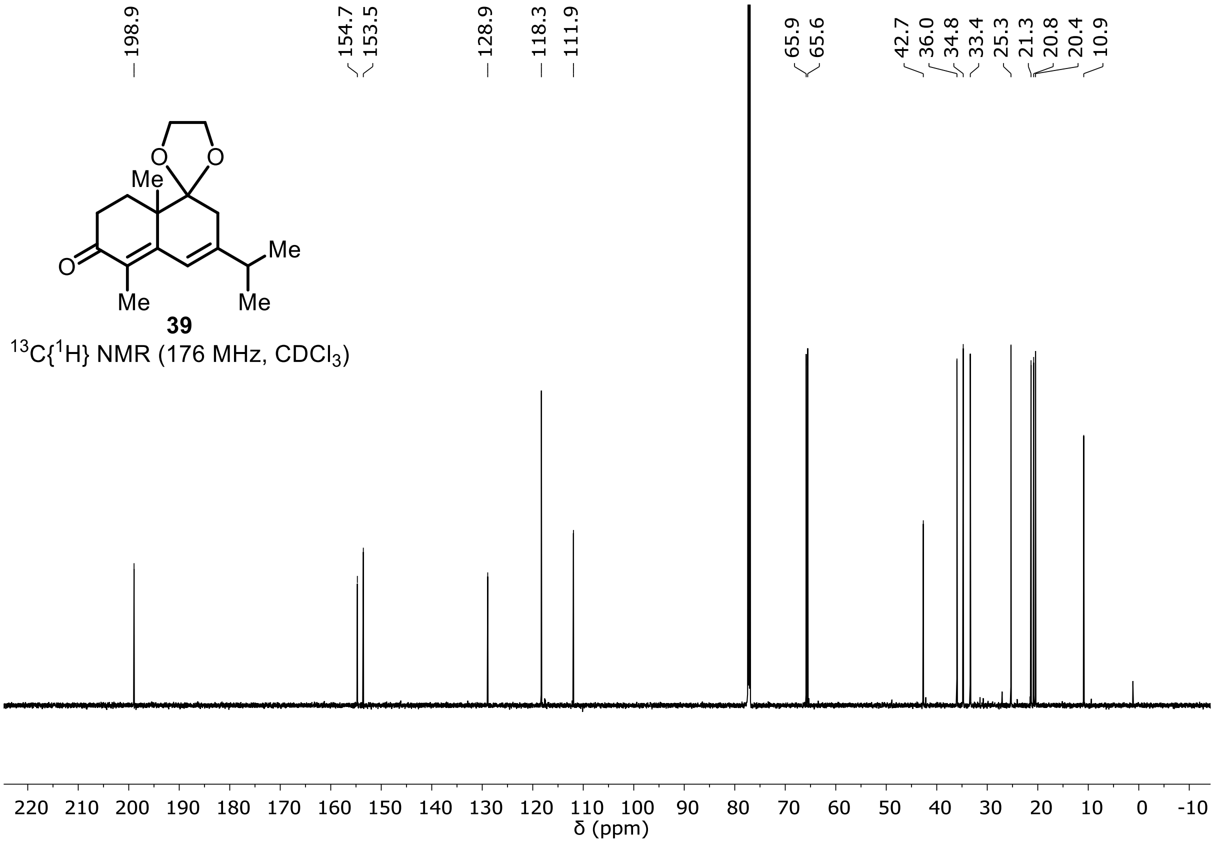
198.9
154.7 153.5
128.9
118.3
111.9
65.9 65.6
42.7 36.0 34.8 33.4 25.3 21.3 20.8 20.4 10.9
Me
O
O
O
Me
Me
Me
39
13C{1H} NMR (176 MHz, CDCl3)
220 210 200 190 180 170 160 150 140 130 120 110 100 90 80 70 60 50 40 30 20 10 0 -10
δ (ppm)

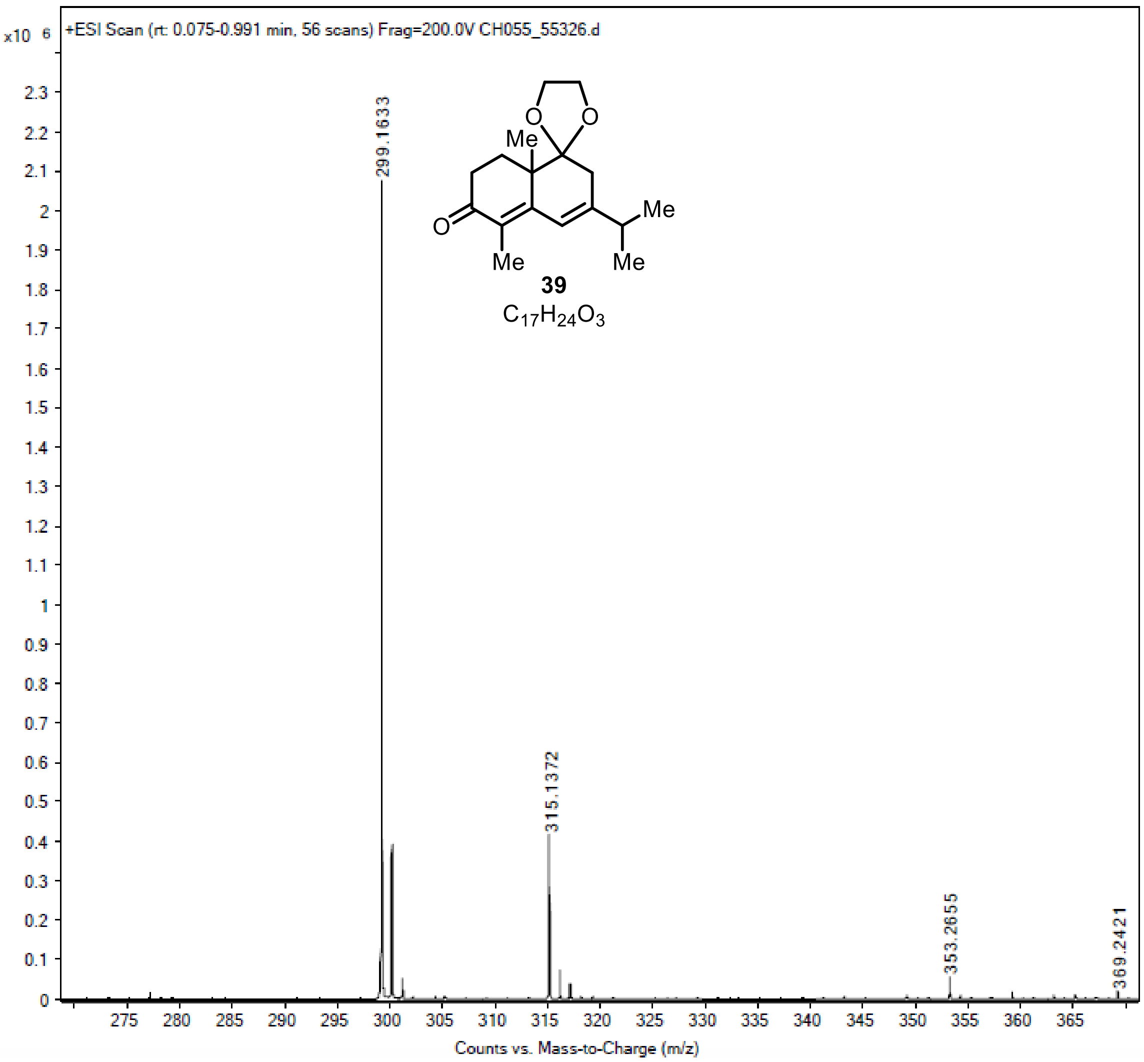

x10 6
+ESI Scan (rt: 0.075-0.991 min, 56 scans) Frag=200.0V CH055_55326.d
299.1633
315.1372
353.2655
369.2421
39
C17H24O3
Counts vs. Mass-to-Charge (m/z)

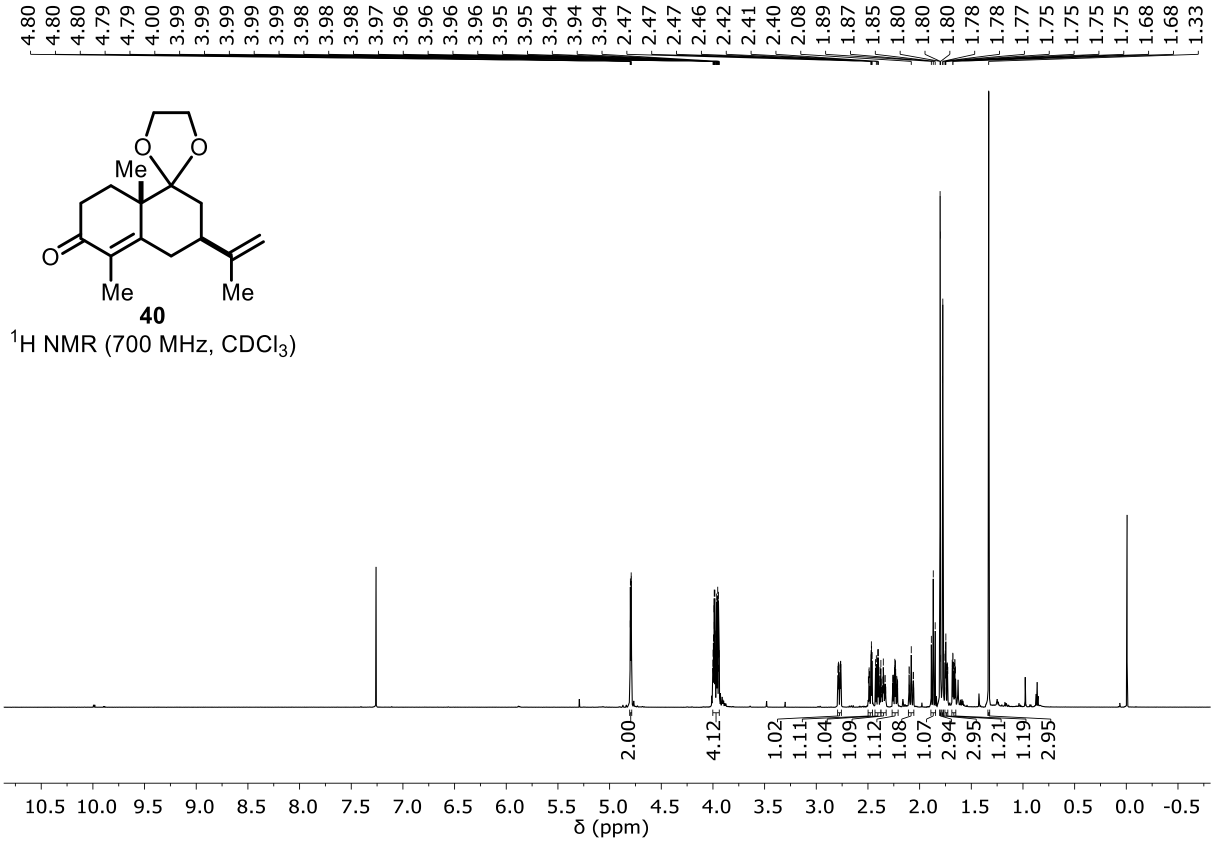
4.80 4.80 4.80 4.79 4.79 4.00 3.99 3.99 3.99 3.99 3.99 3.98 3.98 3.97 3.96 3.96 3.96 3.96 3.95 3.95 3.94 3.94 3.94 2.47 2.47 2.47 2.46 2.42 2.41 2.40 2.08 1.89 1.87 1.85 1.80 1.80 1.80 1.78 1.78 1.77 1.75 1.75 1.75 1.68 1.68 1.33
Me
O
O
O
Me
Me
40
^{1}H NMR (700 MHz, $CDCl_3$)
2.00 4.12 1.02 1.11 1.04 1.09 1.12 1.08 1.07 2.94 2.95 1.21 1.19 2.95
10.5 10.0 9.5 9.0 8.5 8.0 7.5 7.0 6.5 6.0 5.5 5.0 4.5 4.0 3.5 3.0 2.5 2.0 1.5 1.0 0.5 0.0 -0.5
δ (ppm)

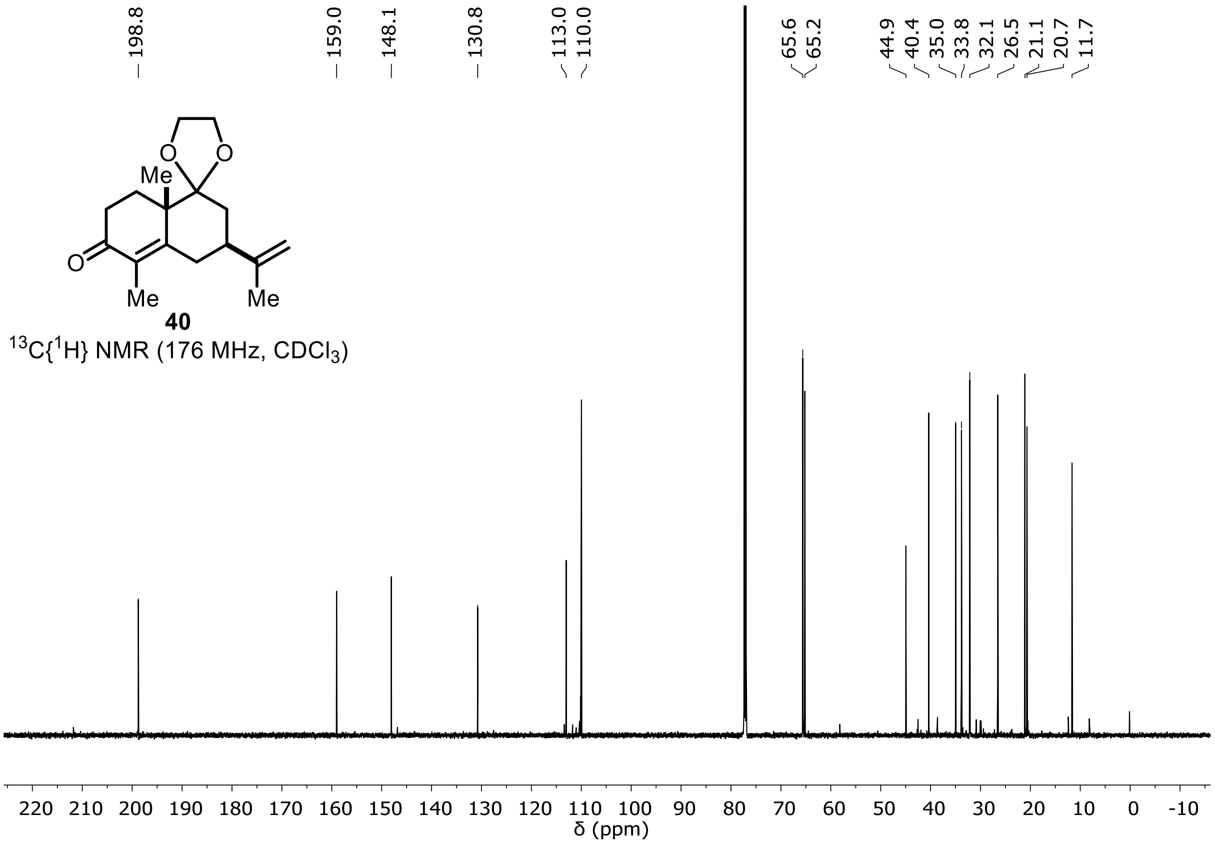
198.8 159.0 148.1 130.8 113.0 110.0 65.6 65.2 44.9 40.4 35.0 33.8 32.1 26.5 21.1 20.7 11.7
Me
O
O
O
Me
Me
40
^{13}C{^{1}H} NMR (176 MHz, $CDCl_3$)
220 210 200 190 180 170 160 150 140 130 120 110 100 90 80 70 60 50 40 30 20 10 0 -10
δ (ppm)

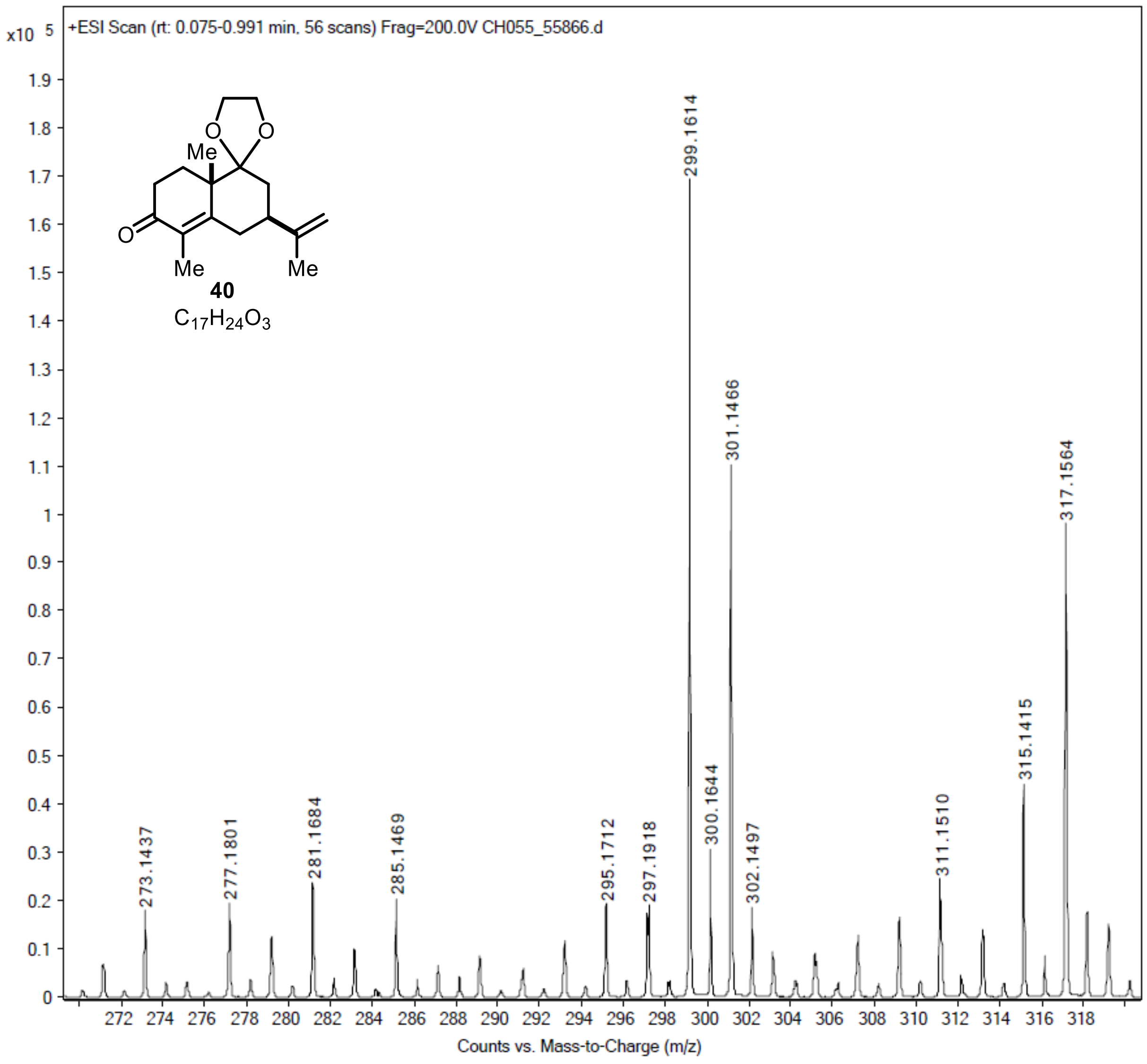

+ESI Scan (rt: 0.075-0.991 min, 56 scans) Frag=200.0V CH055_55866.d
x10 5
Me
O
O
Me
Me
40
C17H24O3
299.1614
301.1466
317.1564
315.1415
300.1644
311.1510
302.1497
297.1918
295.1712
285.1469
281.1684
277.1801
273.1437
Counts vs. Mass-to-Charge (m/z)

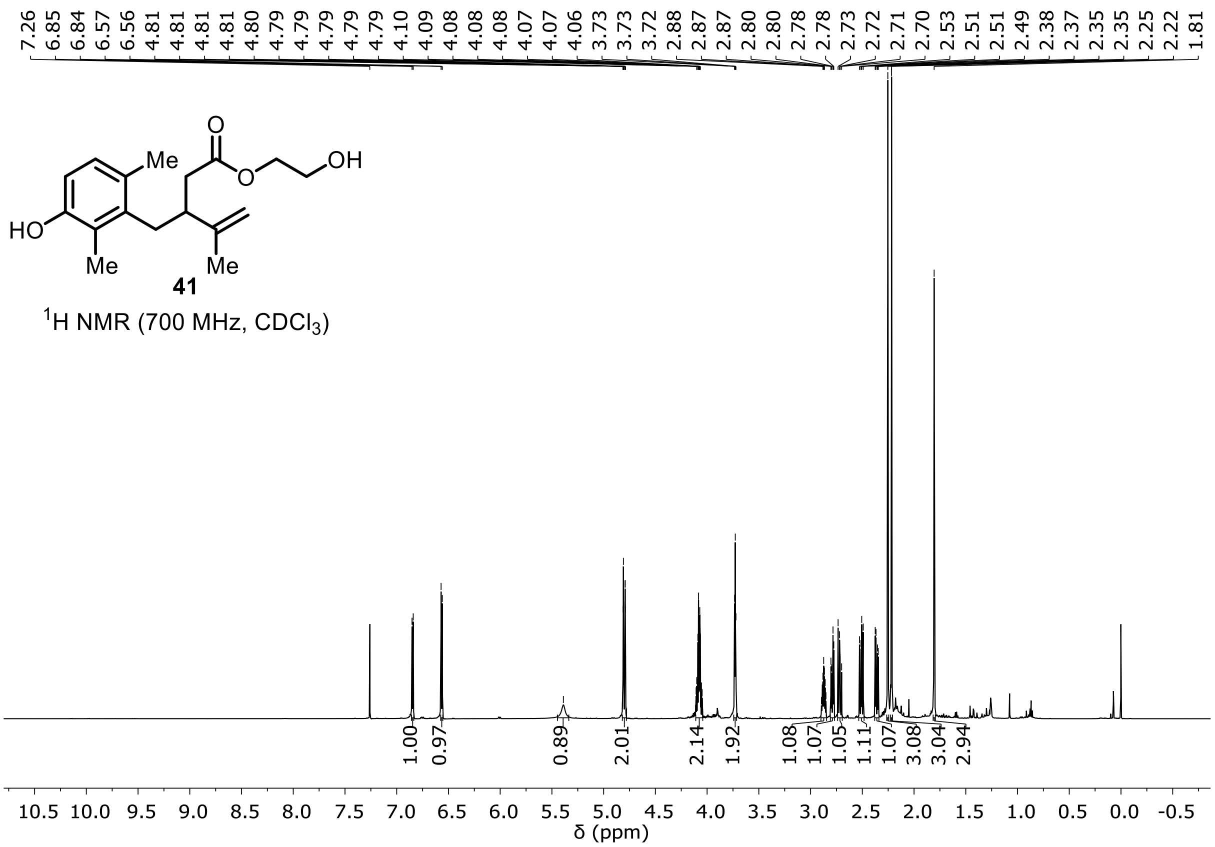
7.26 6.85 6.84 6.57 6.56 4.81 4.81 4.81 4.81 4.80 4.79 4.79 4.79 4.79 4.79 4.10 4.09 4.08 4.08 4.08 4.07 4.07 4.06 3.73 3.73 3.72 2.88 2.87 2.87 2.80 2.80 2.78 2.78 2.73 2.72 2.71 2.70 2.53 2.51 2.51 2.49 2.38 2.37 2.35 2.35 2.25 2.22 1.81
Me
O
O
OH
HO
Me
Me
41
^{1}H NMR (700 MHz, $CDCl_3$)
1.00 0.97 0.89 2.01 2.14 1.92 1.08 1.07 1.05 1.11 1.07 3.08 3.04 2.94
10.5 10.0 9.5 9.0 8.5 8.0 7.5 7.0 6.5 6.0 5.5 5.0 4.5 4.0 3.5 3.0 2.5 2.0 1.5 1.0 0.5 0.0 -0.5
δ (ppm)

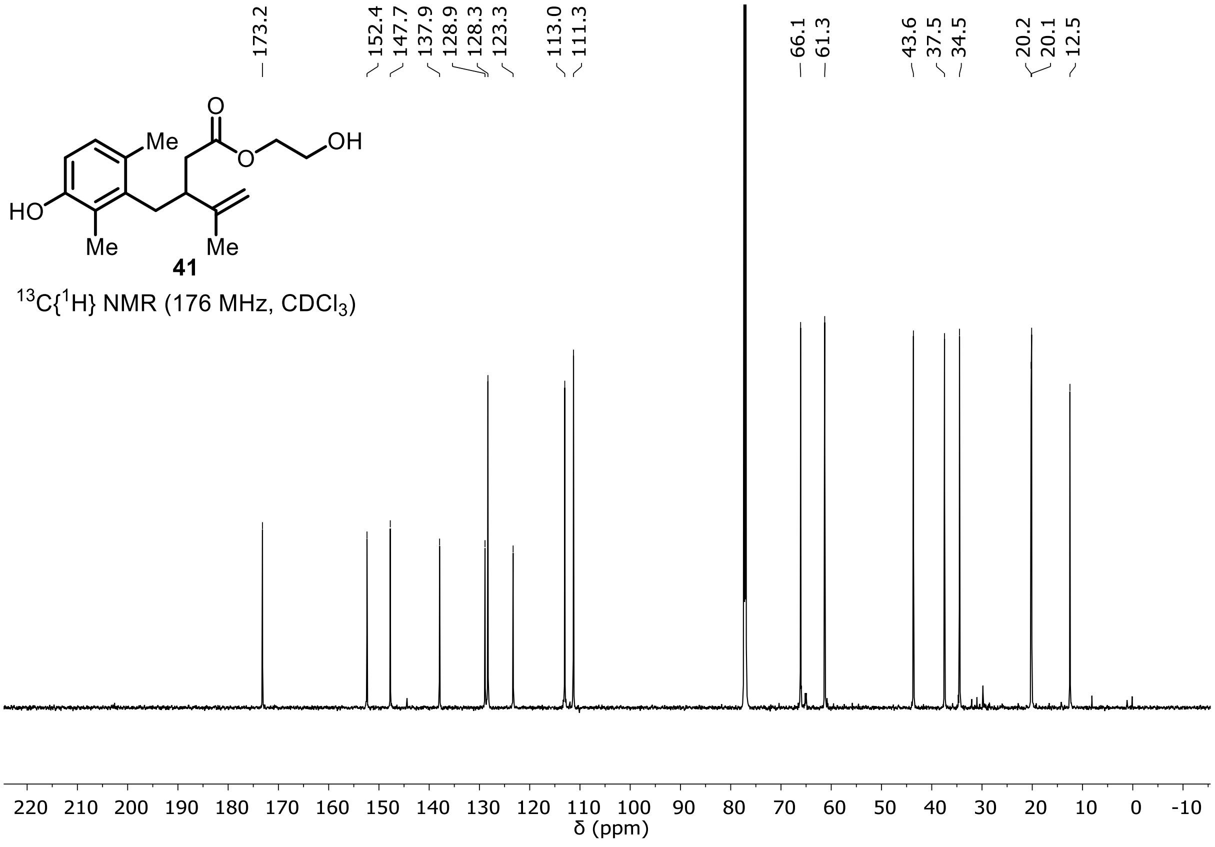
173.2 152.4 147.7 137.9 128.9 128.3 123.3 113.0 111.3 66.1 61.3 43.6 37.5 34.5 20.2 20.1 12.5
Me
O
O
OH
HO
Me
Me
41
$^{13}C\{^1H\}$ NMR (176 MHz, $CDCl_3$)
220 210 200 190 180 170 160 150 140 130 120 110 100 90 80 70 60 50 40 30 20 10 0 -10
δ (ppm)

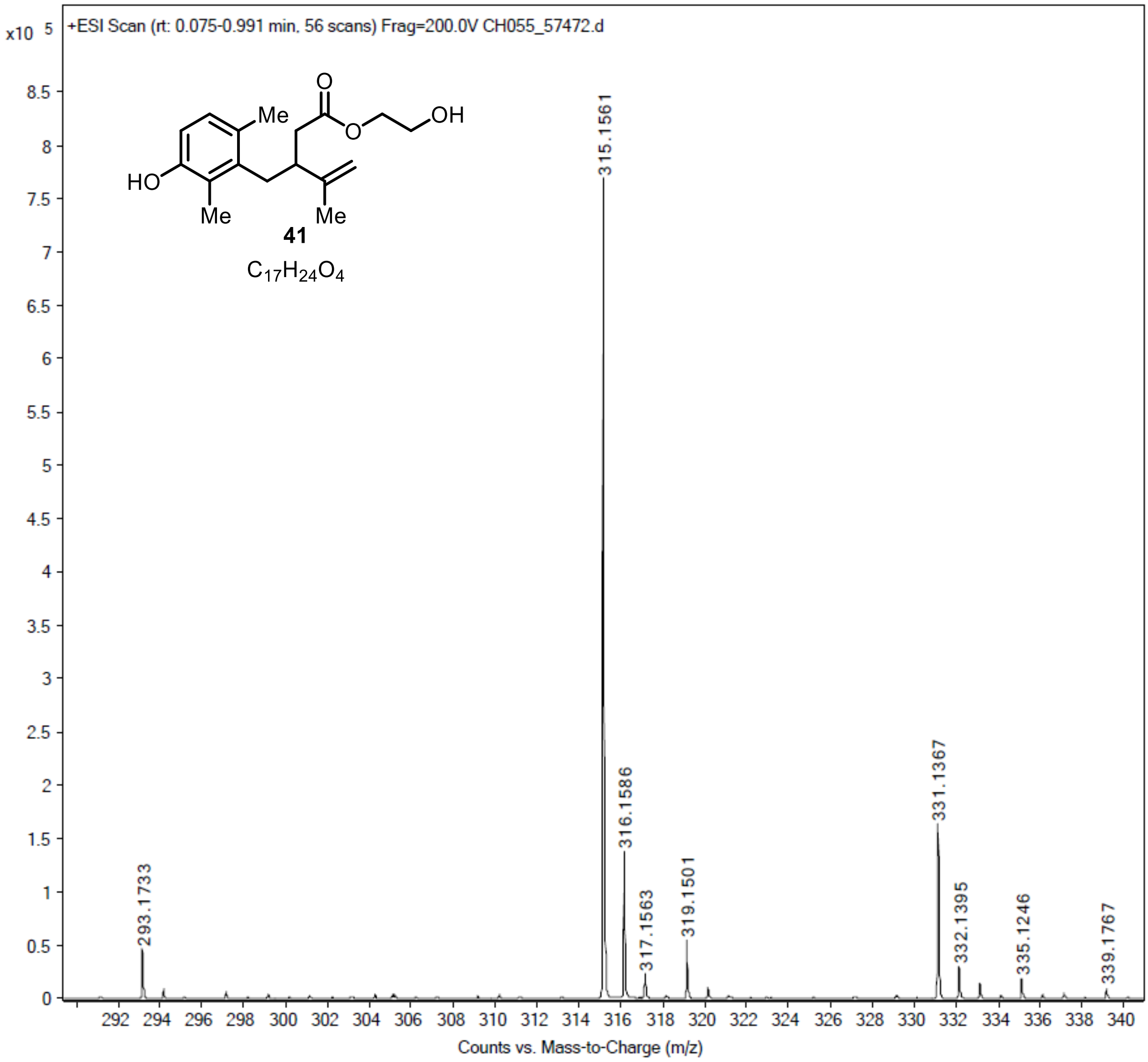

x10 5
+ESI Scan (rt: 0.075-0.991 min, 56 scans) Frag=200.0V CH055_57472.d
41
$C_{17}H_{24}O_4$
HO
Me
OH
O
315.1561
316.1586
317.1563
319.1501
331.1367
332.1395
335.1246
339.1767
293.1733
Counts vs. Mass-to-Charge (m/z)

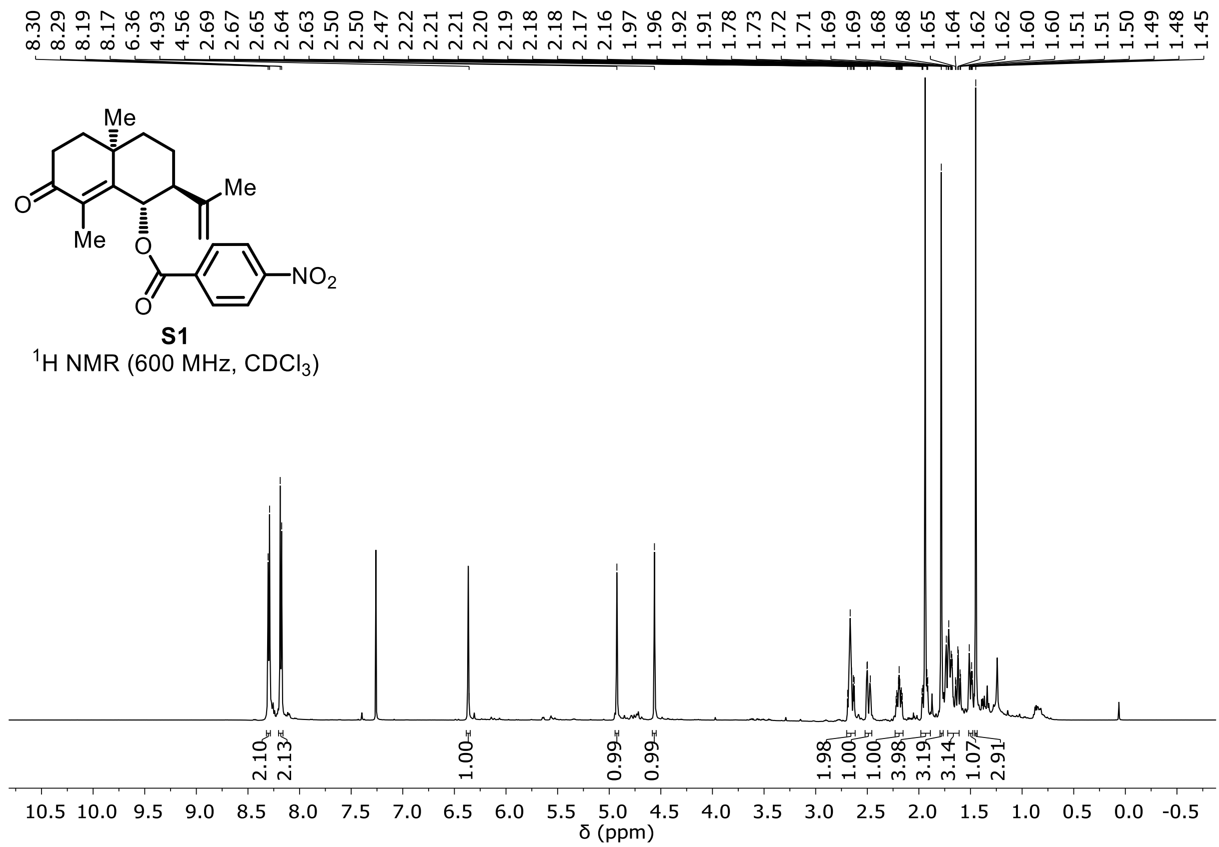
8.30 8.29 8.19 8.17 6.36 4.93 4.56 2.69 2.67 2.65 2.64 2.63 2.50 2.50 2.47 2.22 2.21 2.21 2.20 2.19 2.18 2.18 2.17 2.16 1.97 1.96 1.92 1.91 1.78 1.73 1.72 1.71 1.69 1.69 1.68 1.68 1.65 1.64 1.62 1.62 1.60 1.60 1.51 1.51 1.50 1.49 1.48 1.45
Me
Me
O
Me
O
O
NO_2
S1
^{1}H NMR (600 MHz, $CDCl_3$)
2.10
2.13
1.00
0.99
0.99
1.98
1.00
1.00
3.98
3.19
3.14
1.07
2.91
10.5 10.0 9.5 9.0 8.5 8.0 7.5 7.0 6.5 6.0 5.5 5.0 4.5 4.0 3.5 3.0 2.5 2.0 1.5 1.0 0.5 0.0 -0.5
δ (ppm)

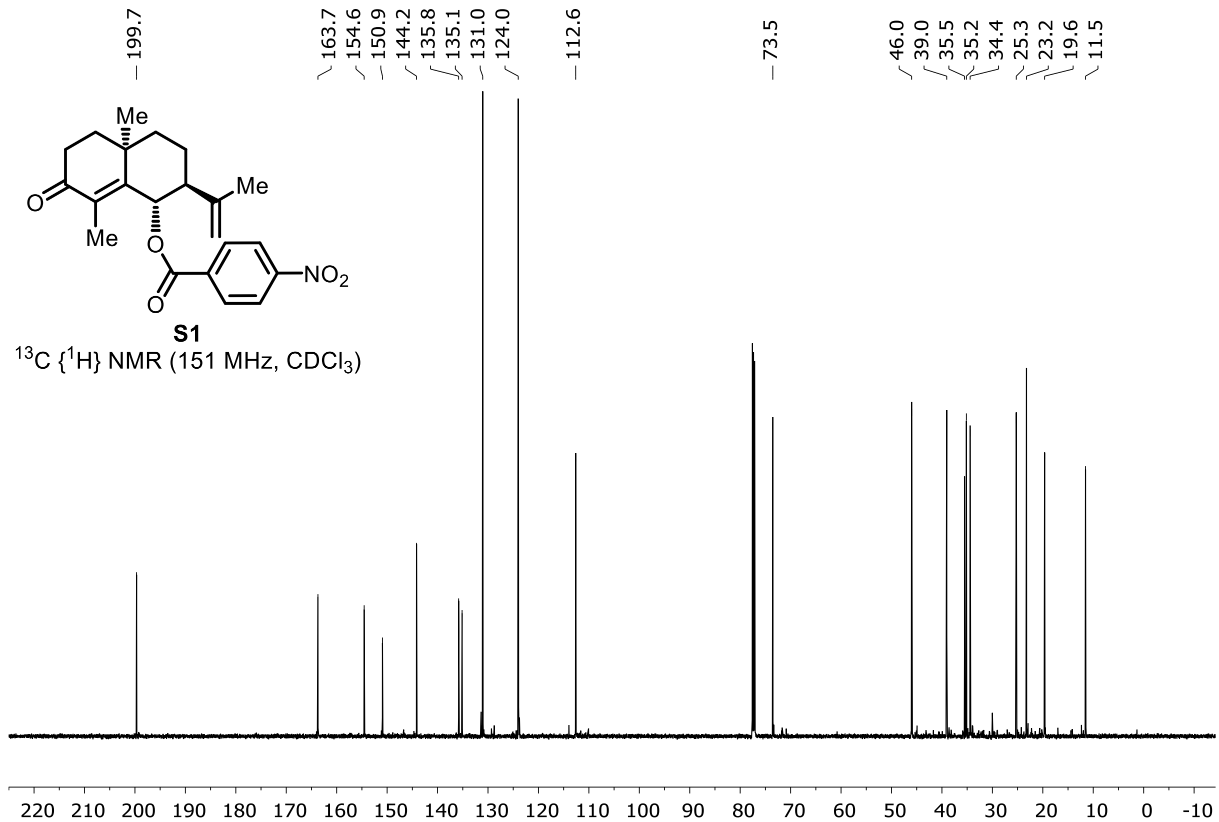
199.7
163.7 154.6 150.9 144.2 135.8 135.1 131.0 124.0
112.6
73.5
46.0 39.0 35.5 35.2 34.4 25.3 23.2 19.6 11.5
Me
Me
O
Me
O
O
NO_2
S1
^{13}C {^{1}H} NMR (151 MHz, $CDCl_3$)
220 210 200 190 180 170 160 150 140 130 120 110 100 90 80 70 60 50 40 30 20 10 0 -10
δ (ppm)

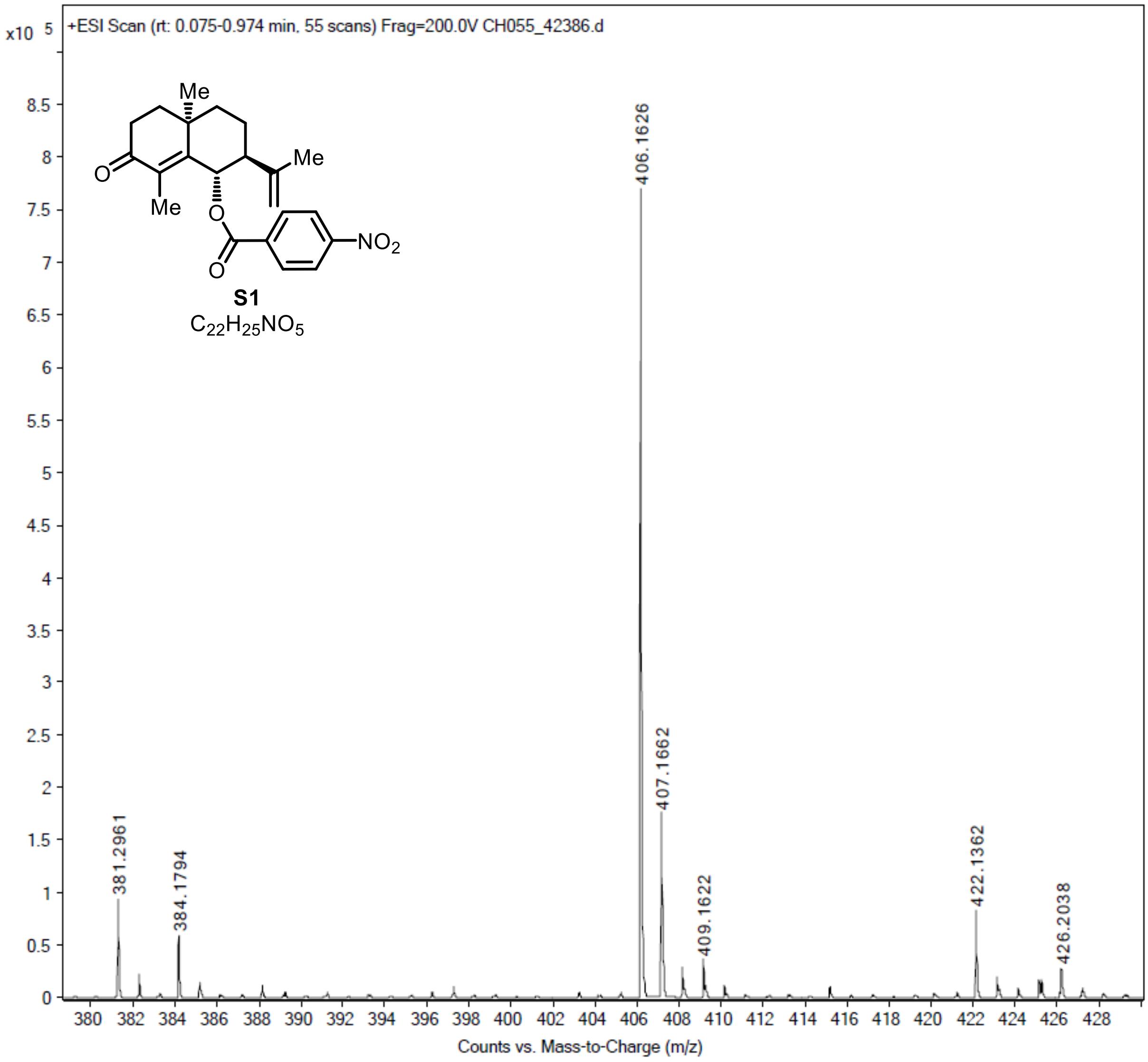

+ESI Scan (rt: 0.075-0.974 min, 55 scans) Frag=200.0V CH055_42386.d
x10 5
Me
O
Me
Me
O
O
NO2
S1
C22H25NO5
406.1626
407.1662
409.1622
381.2961
384.1794
422.1362
426.2038
Counts vs. Mass-to-Charge (m/z)

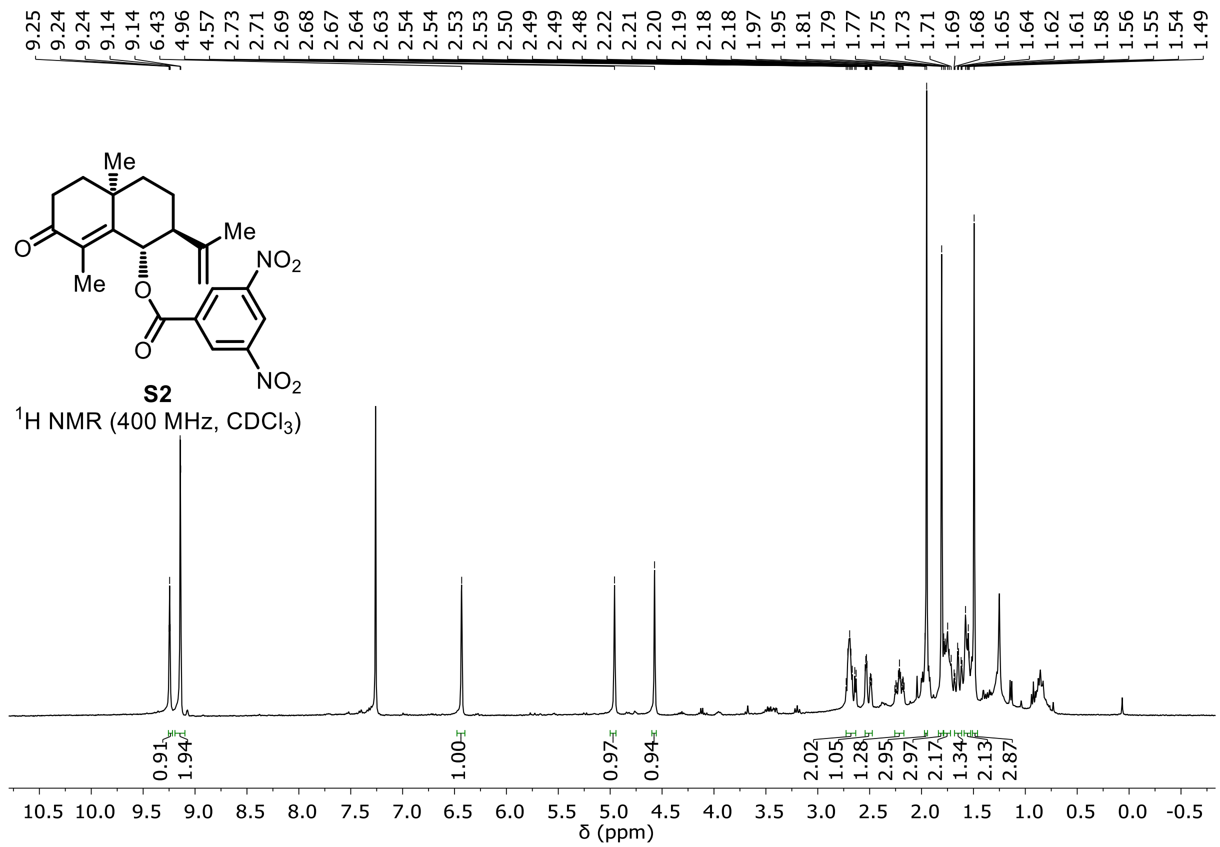
9.25 9.24 9.24 9.14 9.14 6.43 4.96 4.57 2.73 2.71 2.69 2.68 2.67 2.64 2.63 2.54 2.54 2.53 2.53 2.50 2.49 2.49 2.48 2.22 2.21 2.20 2.19 2.18 2.18 1.97 1.95 1.81 1.79 1.77 1.75 1.73 1.71 1.69 1.68 1.65 1.64 1.62 1.61 1.58 1.56 1.55 1.54 1.49
Me
O
Me
Me
NO_2
O
O
NO_2
S2
^{1}H NMR (400 MHz, $CDCl_3$)
0.91 1.94 1.00 0.97 0.94 2.02 1.05 1.28 2.95 2.97 2.17 1.34 2.13 2.87
10.5 10.0 9.5 9.0 8.5 8.0 7.5 7.0 6.5 6.0 5.5 5.0 4.5 4.0 3.5 3.0 2.5 2.0 1.5 1.0 0.5 0.0 -0.5
δ (ppm)

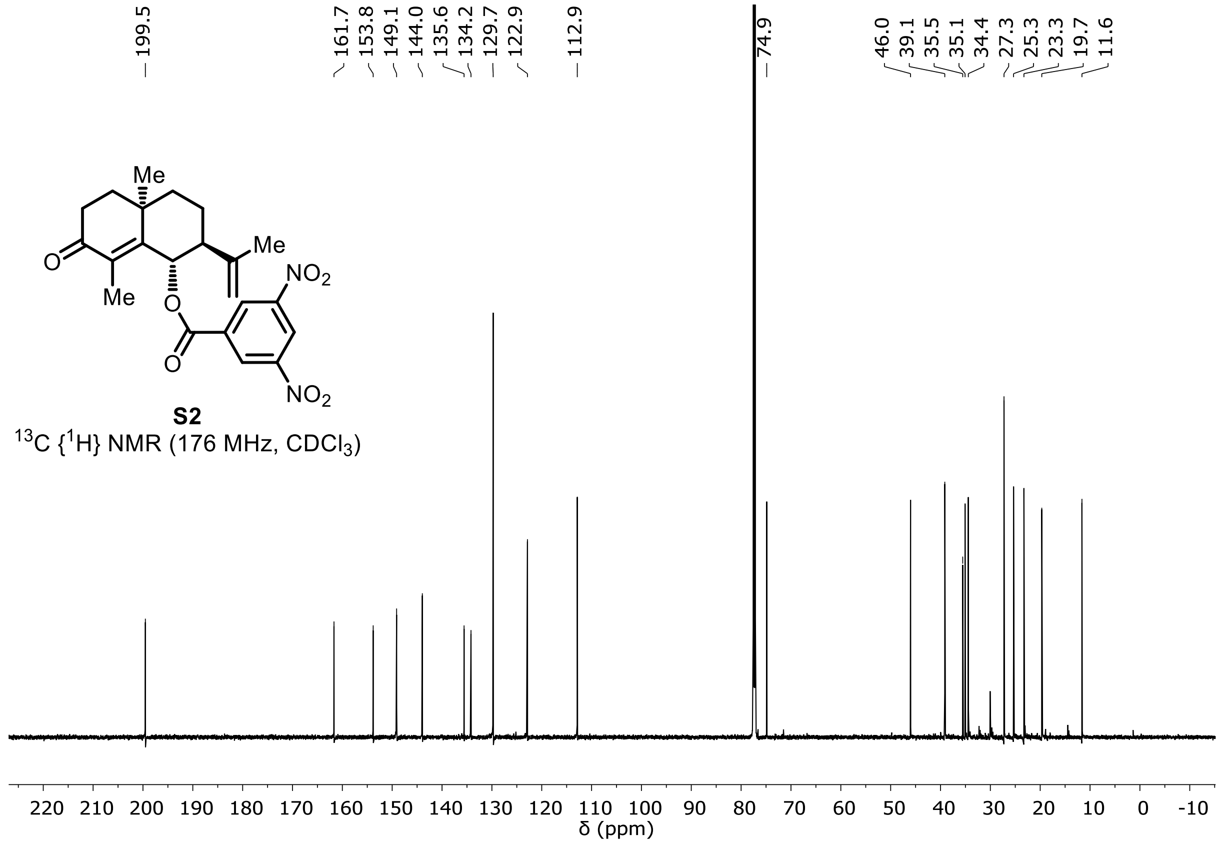
199.5
161.7 153.8 149.1 144.0 135.6 134.2 129.7 122.9
112.9
74.9
46.0 39.1 35.5 35.1 34.4 27.3 25.3 23.3 19.7 11.6
Me
O
Me
Me
NO_2
O
O
NO_2
S2
^{13}C {^{1}H} NMR (176 MHz, $CDCl_3$)
220 210 200 190 180 170 160 150 140 130 120 110 100 90 80 70 60 50 40 30 20 10 0 -10
δ (ppm)

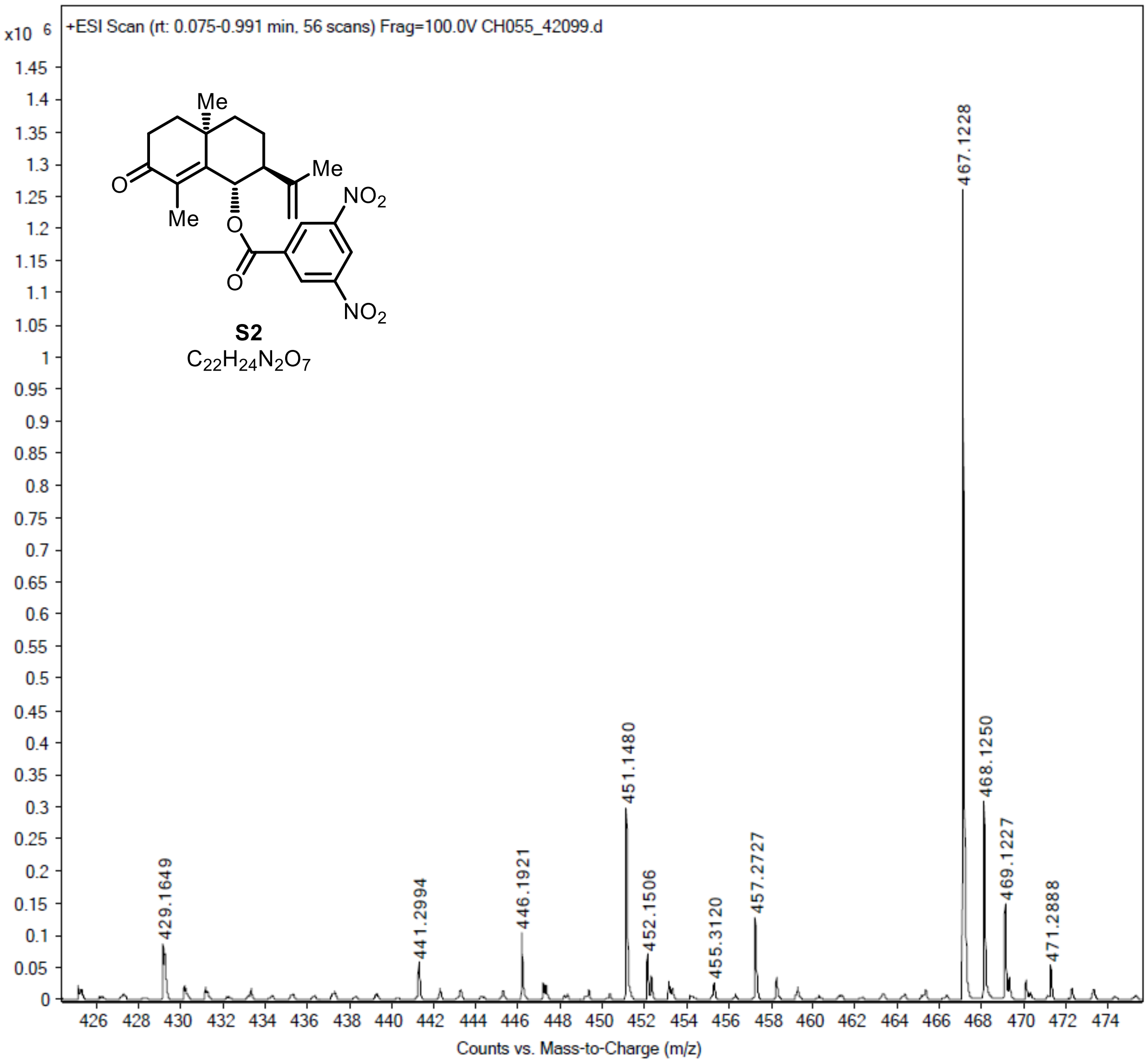
+ESI Scan (rt: 0.075-0.991 min, 56 scans) Frag=100.0V CH055_42099.d
x10 6
S2
$C_{22}H_{24}N_2O_7$
467.1228
468.1250
469.1227
471.2888
451.1480
452.1506
455.3120
457.2727
446.1921
441.2994
429.1649
Counts vs. Mass-to-Charge (m/z)

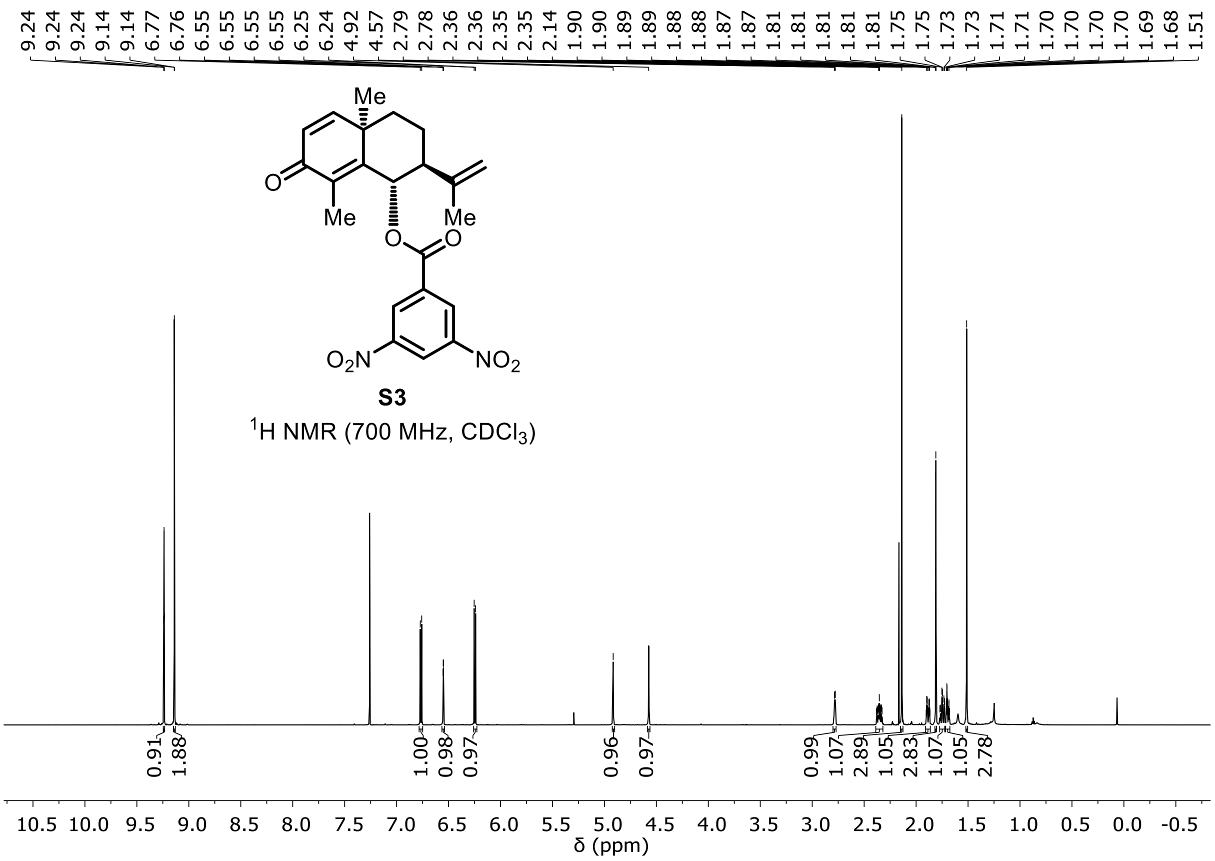
9.24 9.24 9.24 9.14 9.14 6.77 6.76 6.55 6.55 6.55 6.55 6.25 6.24 4.92 4.57 2.79 2.78 2.36 2.36 2.35 2.35 2.14 1.90 1.90 1.89 1.89 1.88 1.88 1.87 1.87 1.81 1.81 1.81 1.81 1.81 1.75 1.75 1.73 1.73 1.71 1.71 1.70 1.70 1.70 1.70 1.69 1.68 1.51
Me
O
Me
Me
O
O
O2N
NO2
S3
1H NMR (700 MHz, CDCl3)
0.91 1.88 1.00 0.98 0.97 0.96 0.97 0.99 1.07 2.89 1.05 2.83 1.07 1.05 2.78
10.5 10.0 9.5 9.0 8.5 8.0 7.5 7.0 6.5 6.0 5.5 5.0 4.5 4.0 3.5 3.0 2.5 2.0 1.5 1.0 0.5 0.0 -0.5
δ (ppm)

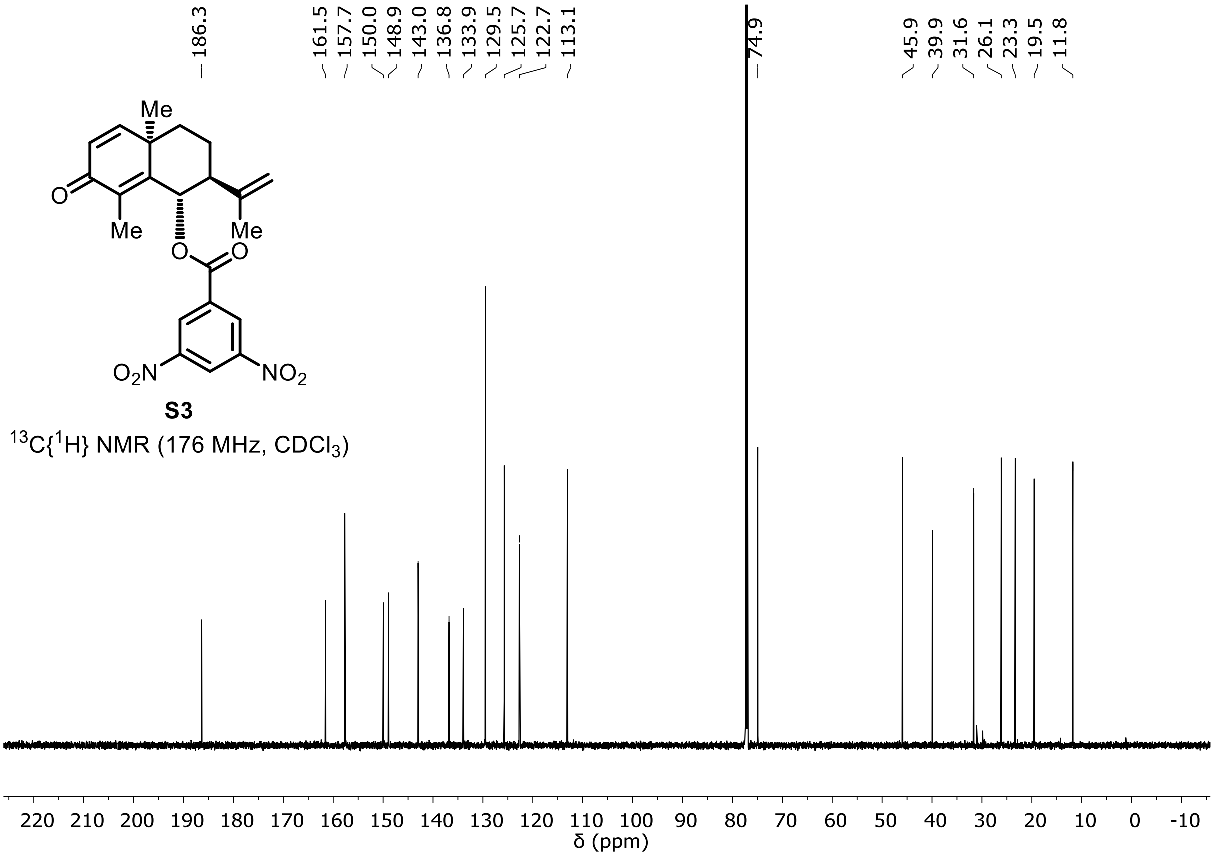
186.3 161.5 157.7 150.0 148.9 143.0 136.8 133.9 129.5 125.7 122.7 113.1 74.9 45.9 39.9 31.6 26.1 23.3 19.5 11.8
Me
O
Me
Me
O
O
O2N
NO2
S3
13C{1H} NMR (176 MHz, CDCl3)
220 210 200 190 180 170 160 150 140 130 120 110 100 90 80 70 60 50 40 30 20 10 0 -10
δ (ppm)

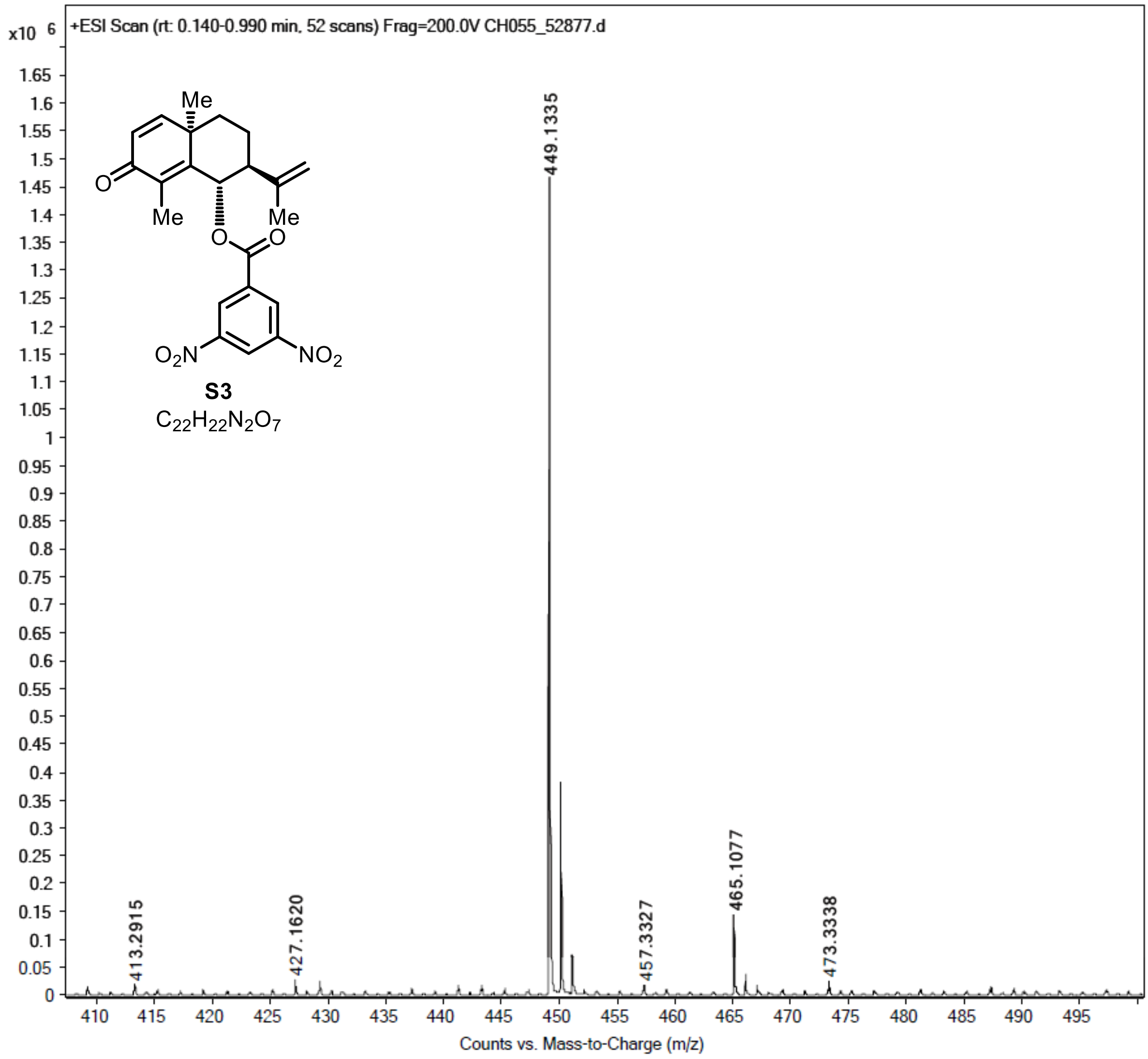
+ESI Scan (rt: 0.140-0.990 min, 52 scans) Frag=200.0V CH055_52877.d
x10 6
S3
C22H22N2O7
449.1335
465.1077
413.2915
427.1620
457.3327
473.3338
Counts vs. Mass-to-Charge (m/z)

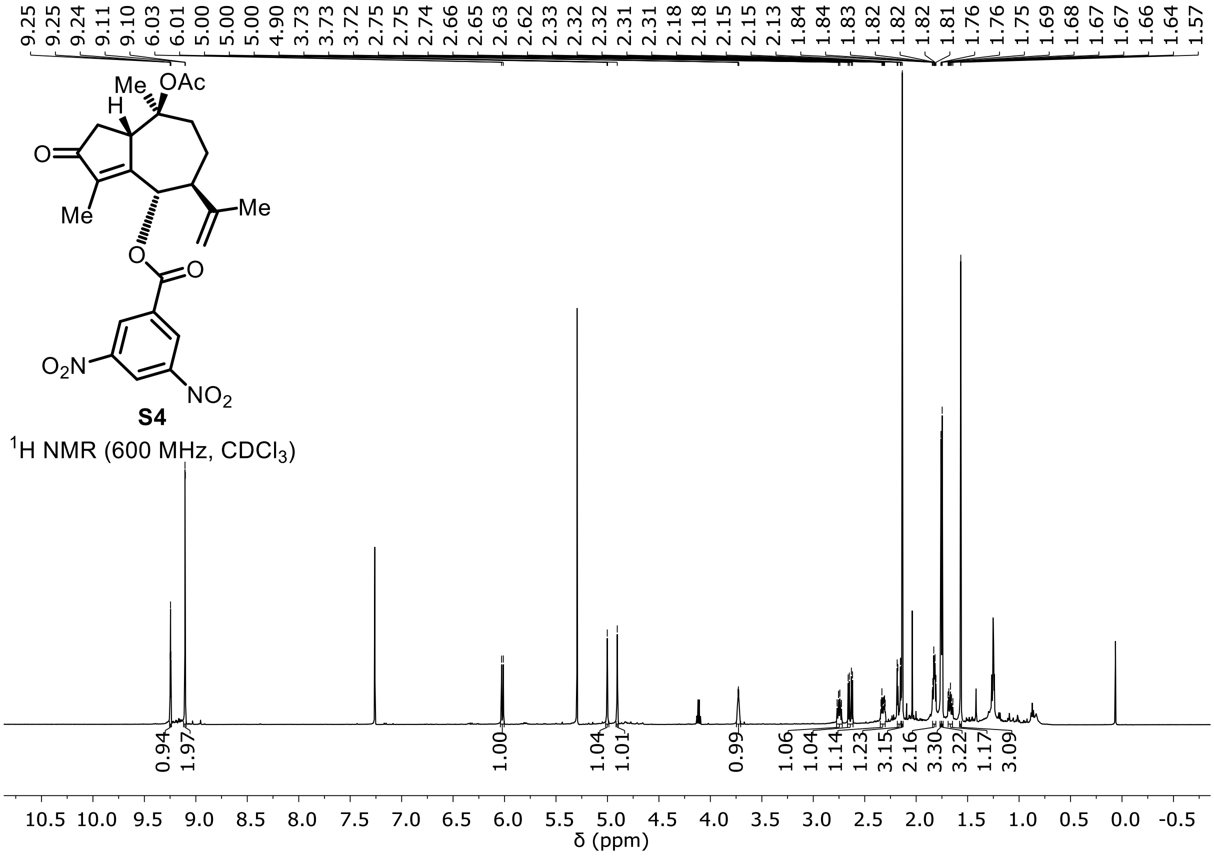
9.25 9.25 9.24 9.11 9.10 6.03 6.01 5.00 5.00 5.00 4.90 3.73 3.73 3.72 2.75 2.75 2.74 2.66 2.65 2.63 2.62 2.33 2.32 2.32 2.31 2.31 2.18 2.18 2.15 2.15 2.13 1.84 1.84 1.83 1.82 1.82 1.82 1.81 1.76 1.76 1.75 1.69 1.68 1.67 1.67 1.66 1.64 1.57
Me OAc
H
O
Me
Me
O
O
O_2N
NO_2
S4
^{1}H NMR (600 MHz, $CDCl_3$)
0.94 1.97 1.00 1.04 1.01 0.99 1.06 1.04 1.14 1.23 3.15 2.16 3.30 3.22 1.17 3.09
10.5 10.0 9.5 9.0 8.5 8.0 7.5 7.0 6.5 6.0 5.5 5.0 4.5 4.0 3.5 3.0 2.5 2.0 1.5 1.0 0.5 0.0 -0.5
δ (ppm)

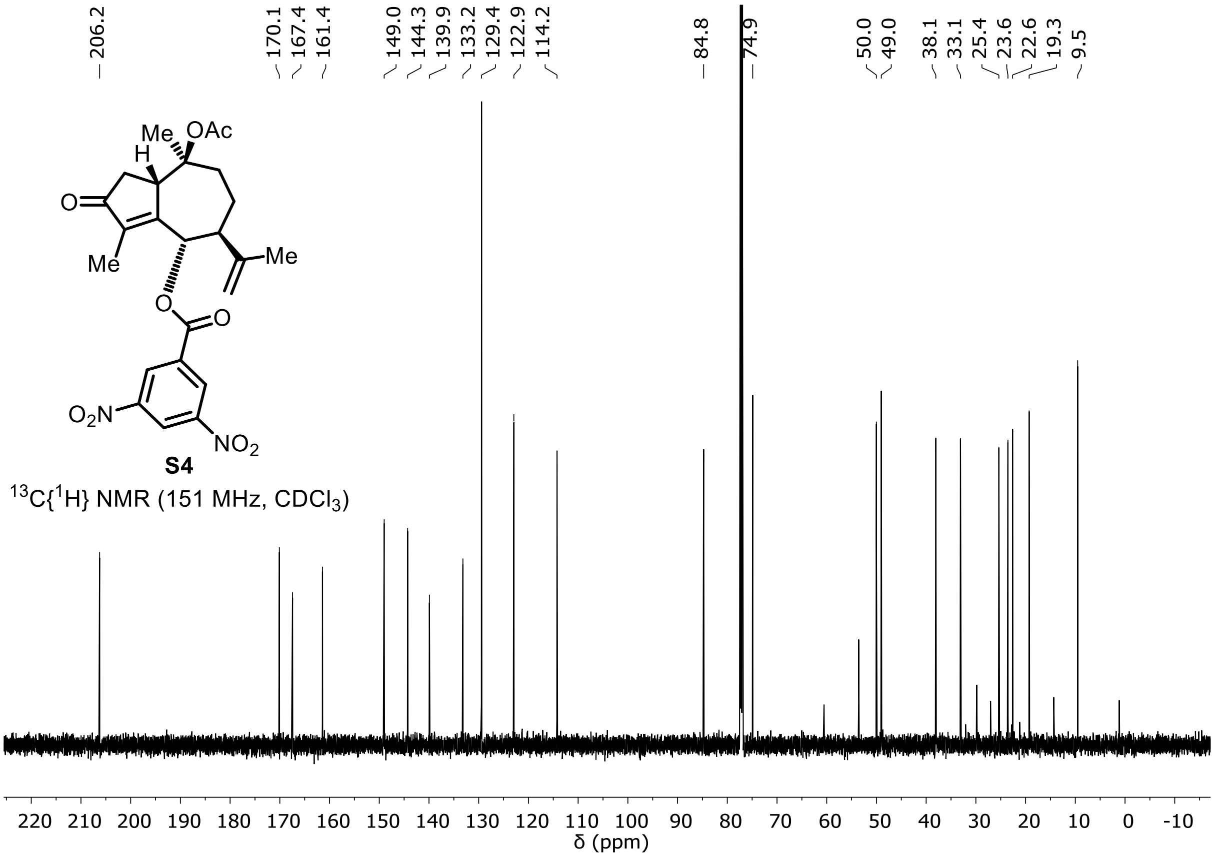
206.2 170.1 167.4 161.4 149.0 144.3 139.9 133.2 129.4 122.9 114.2 84.8 74.9 50.0 49.0 38.1 33.1 25.4 23.6 22.6 19.3 9.5
Me OAc
H
O
Me
Me
O
O
O_2N
NO_2
S4
^{13}C{^{1}H} NMR (151 MHz, $CDCl_3$)
220 210 200 190 180 170 160 150 140 130 120 110 100 90 80 70 60 50 40 30 20 10 0 -10
δ (ppm)

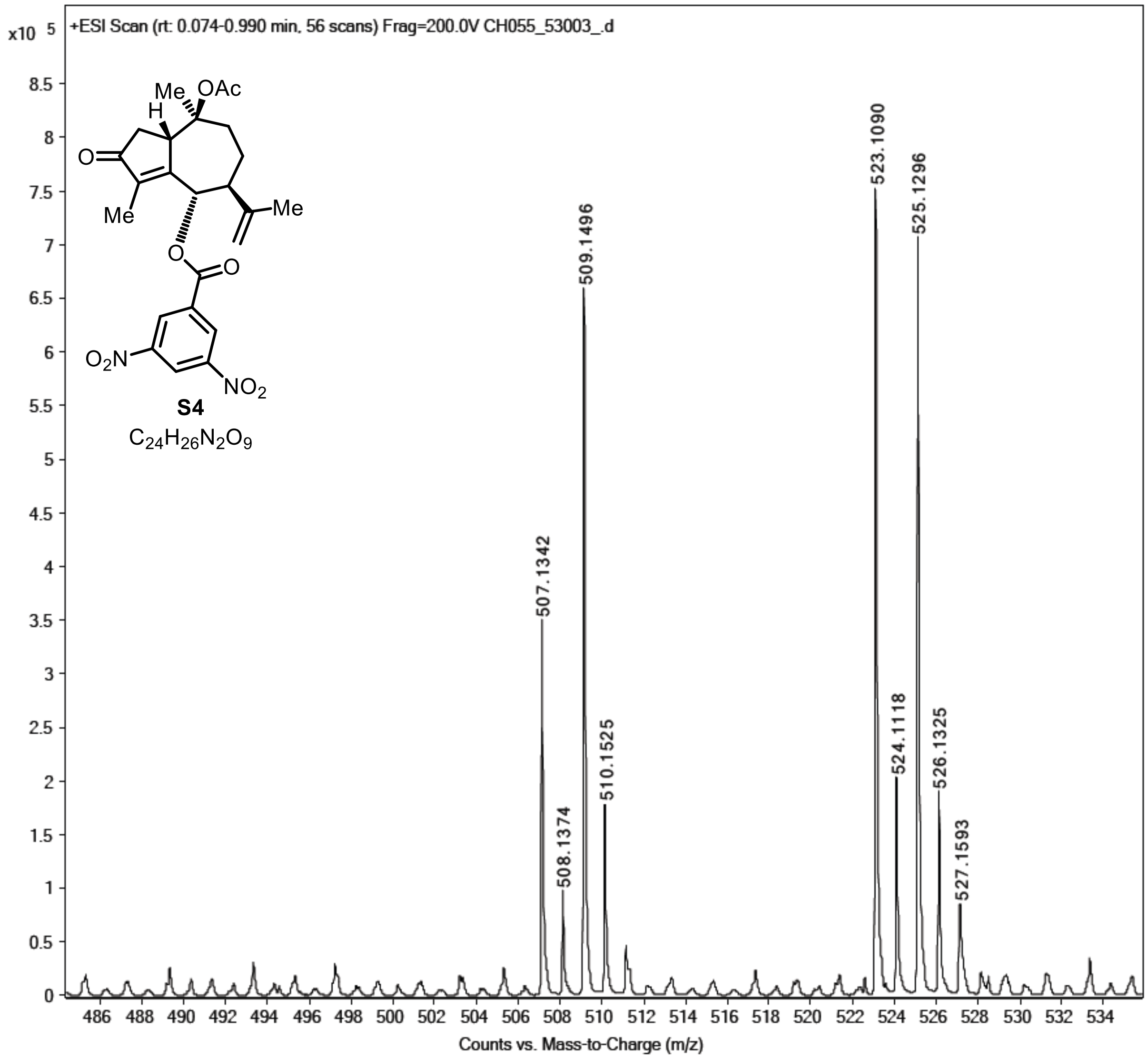

+ESI Scan (rt: 0.074-0.990 min, 56 scans) Frag=200.0V CH055_53003_.d
x10 5
S4
C24H26N2O9
507.1342
508.1374
509.1496
510.1525
523.1090
524.1118
525.1296
526.1325
527.1593
Counts vs. Mass-to-Charge (m/z)

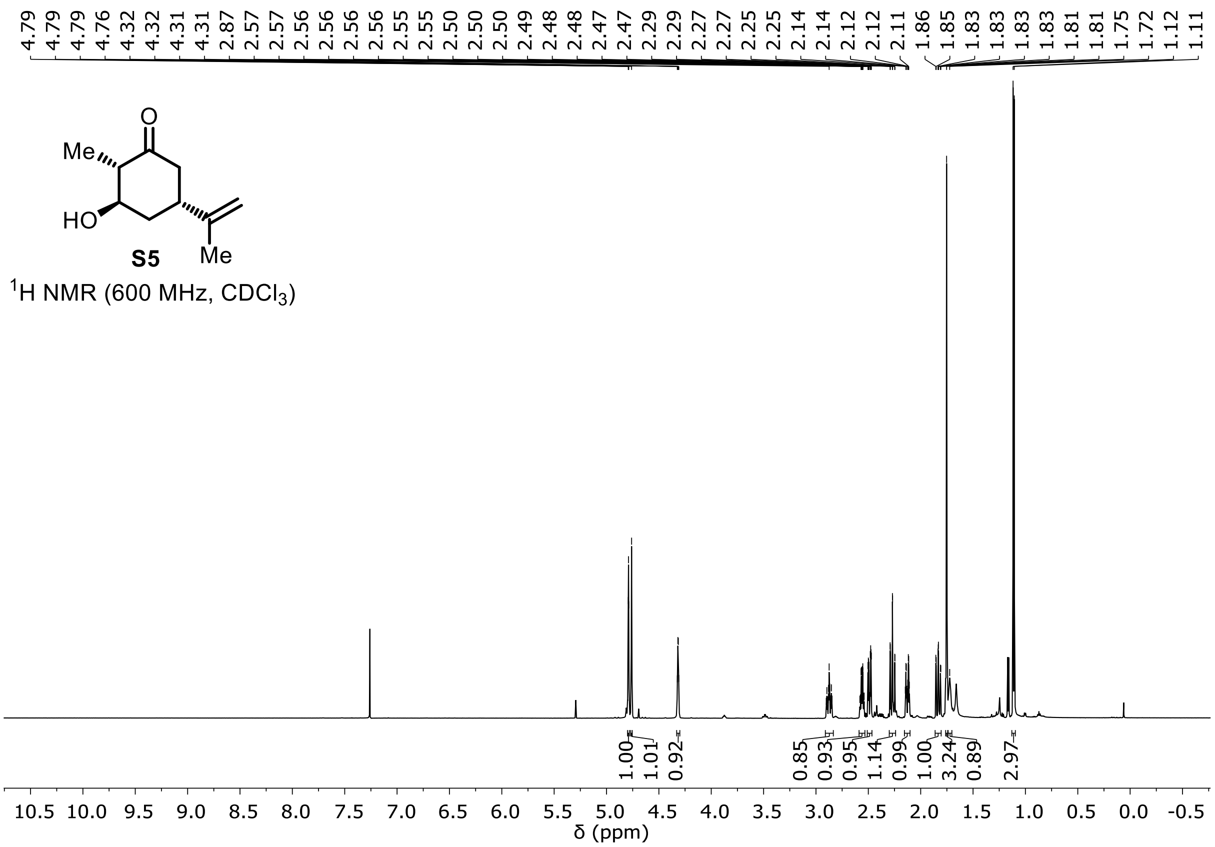
4.79 4.79 4.79 4.76 4.32 4.32 4.31 4.31 2.87 2.57 2.57 2.56 2.56 2.56 2.55 2.55 2.50 2.50 2.50 2.49 2.48 2.48 2.47 2.47 2.29 2.29 2.27 2.27 2.25 2.25 2.14 2.14 2.12 2.12 2.11 1.86 1.85 1.83 1.83 1.83 1.83 1.81 1.81 1.75 1.72 1.12 1.11
O
Me
HO
S5
Me
1H NMR (600 MHz, CDCl3)
1.00 1.01 0.92 0.85 0.93 0.95 1.14 0.99 1.00 3.24 0.89 2.97
10.5 10.0 9.5 9.0 8.5 8.0 7.5 7.0 6.5 6.0 5.5 5.0 4.5 4.0 3.5 3.0 2.5 2.0 1.5 1.0 0.5 0.0 -0.5
δ (ppm)

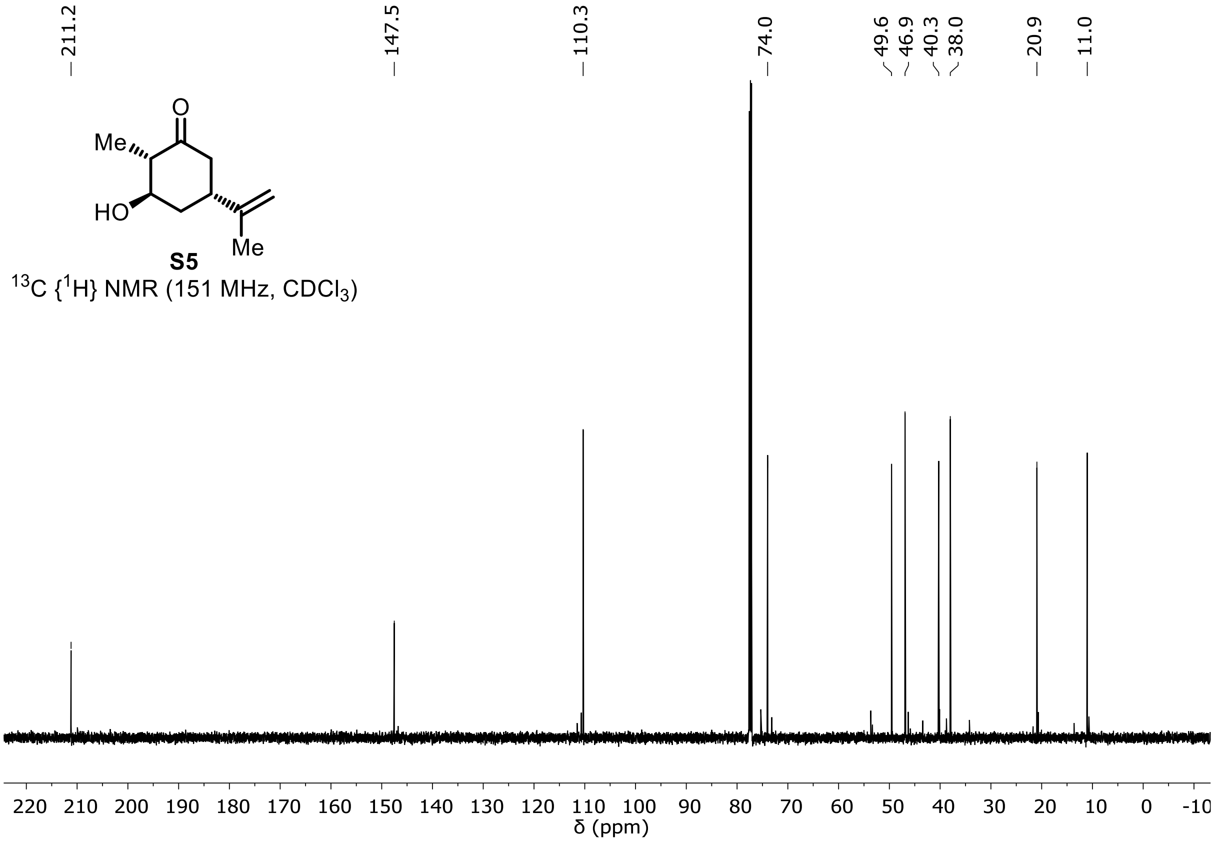
211.2
147.5
110.3
74.0
49.6 46.9 40.3 38.0
20.9
11.0
O
Me
HO
S5
Me
13C {1H} NMR (151 MHz, CDCl3)
220 210 200 190 180 170 160 150 140 130 120 110 100 90 80 70 60 50 40 30 20 10 0 -10
δ (ppm)

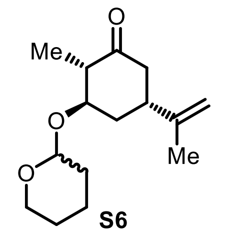

S6

^{1}H NMR (400 MHz, $CDCl_3$)

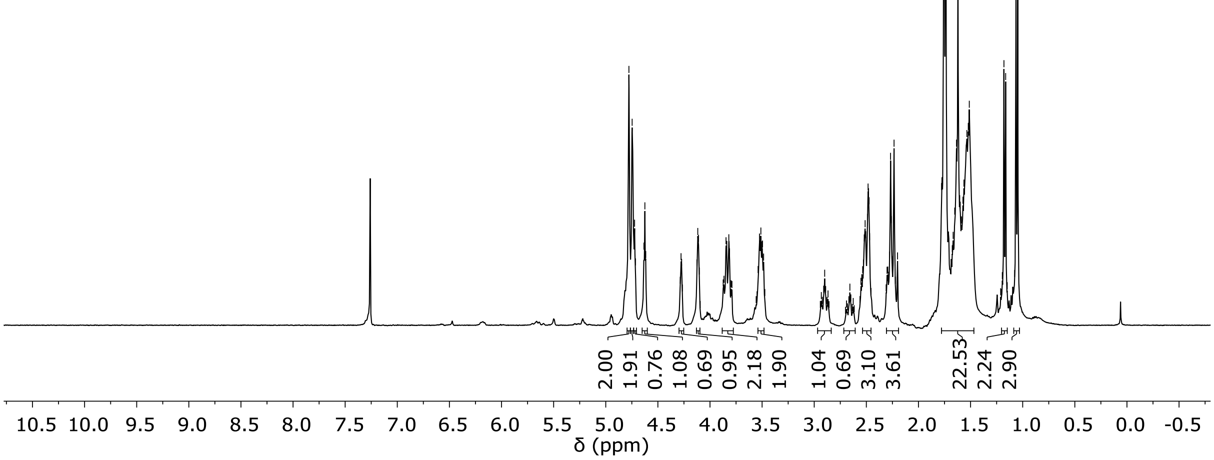

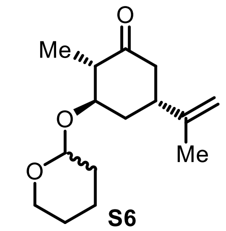

S6

^{13}C{^{1}H} NMR (151 MHz, $CDCl_3$)

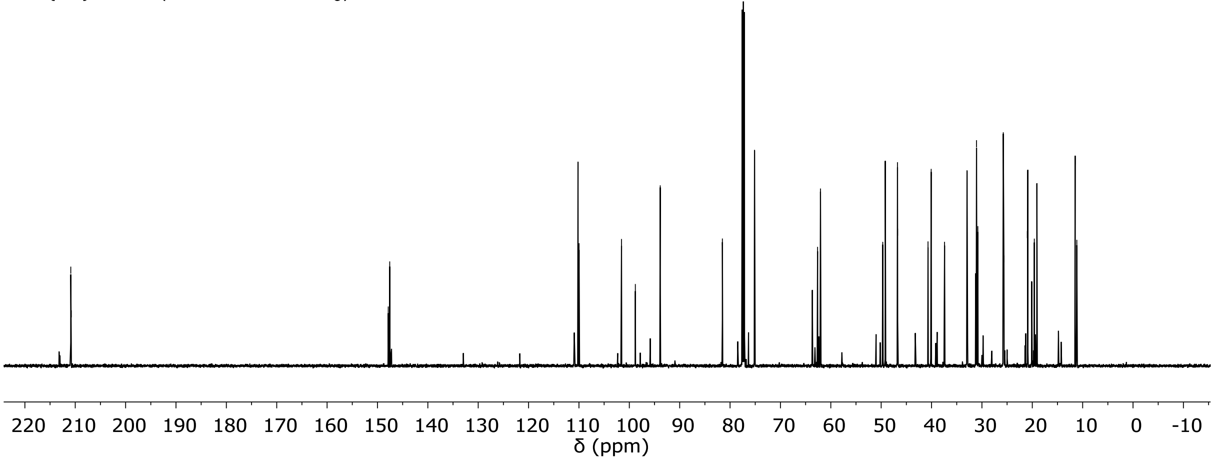

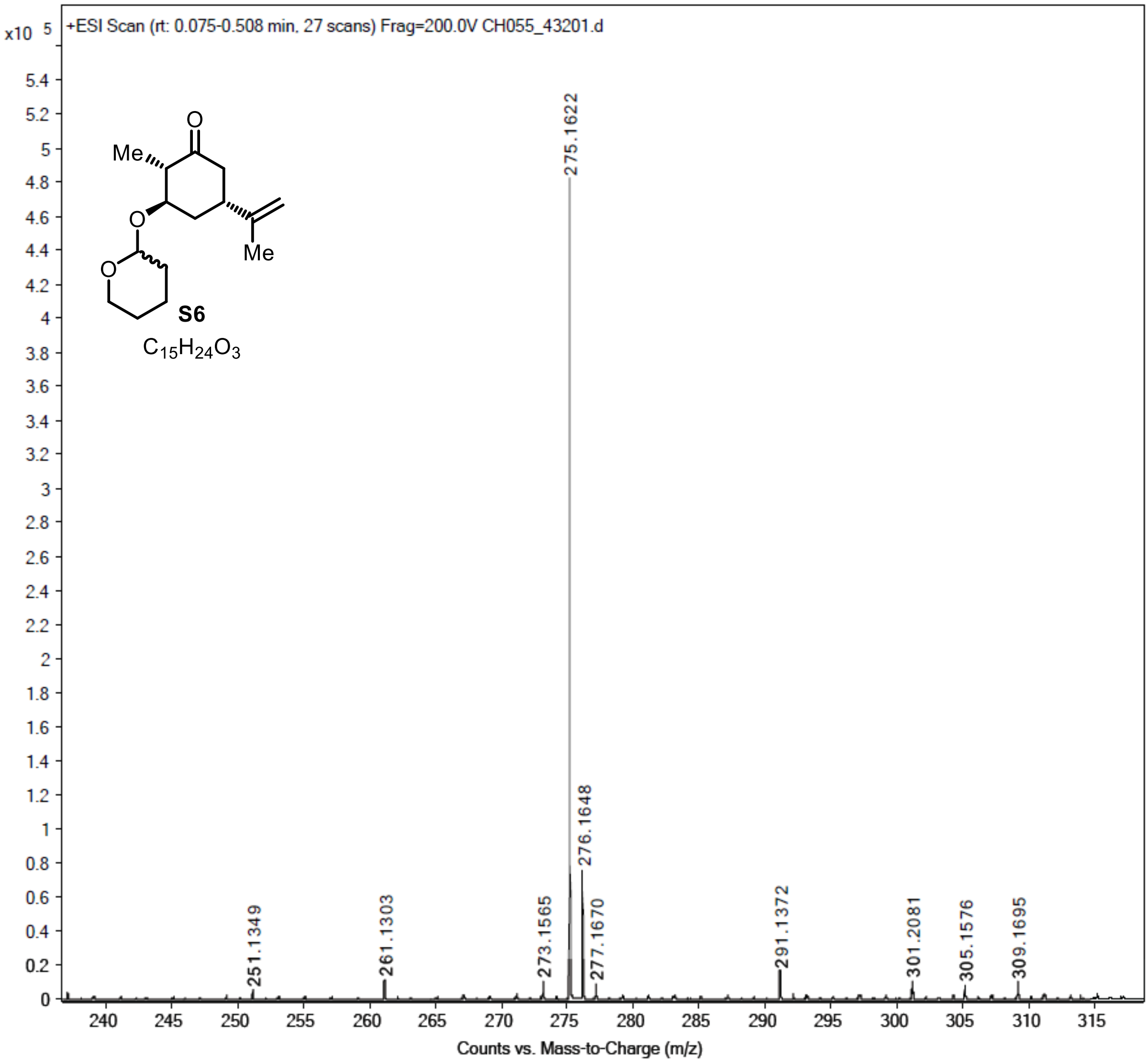
+ESI Scan (rt: 0.075-0.508 min, 27 scans) Frag=200.0V CH055_43201.d
x10 5
Me
O
Me
S6
C15H24O3
275.1622
276.1648
277.1670
273.1565
251.1349
261.1303
291.1372
301.2081
305.1576
309.1695
Counts vs. Mass-to-Charge (m/z)

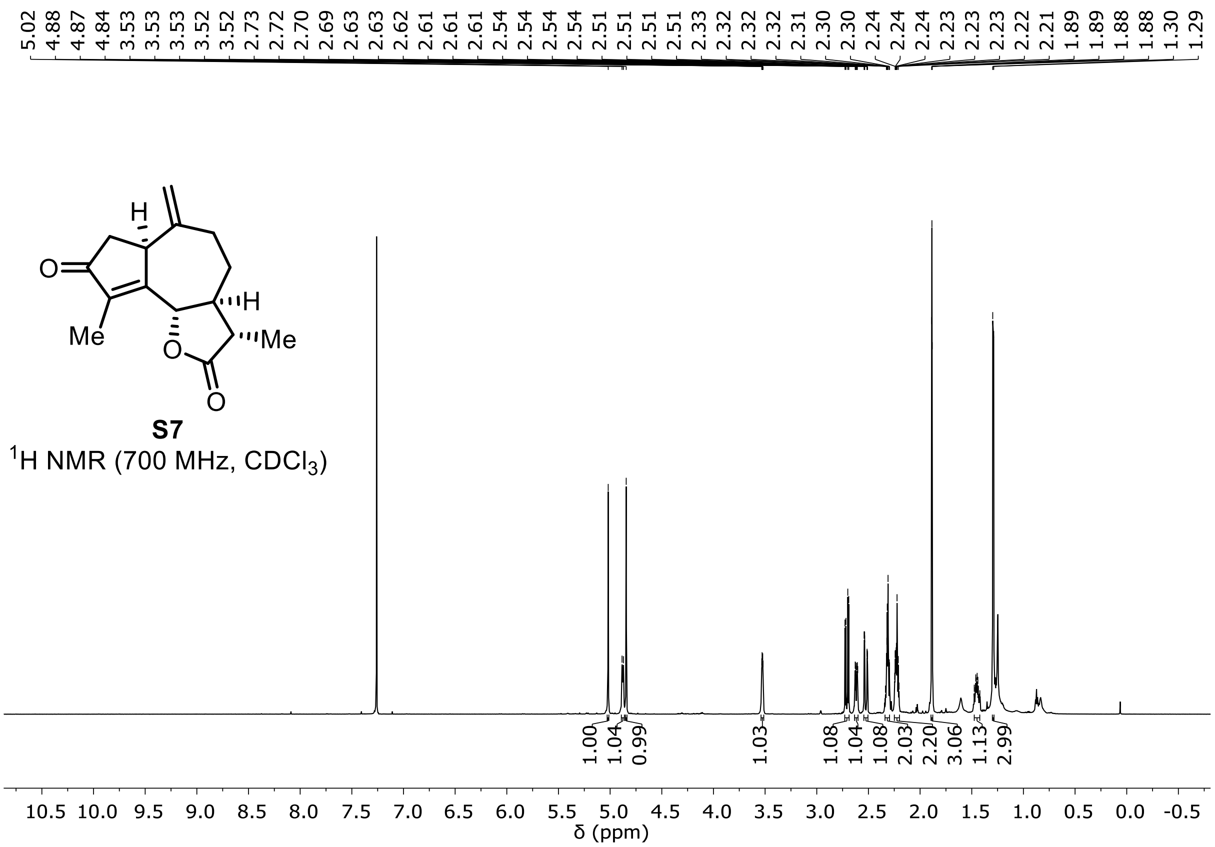
5.02 4.88 4.87 4.84 3.53 3.53 3.53 3.52 3.52 2.73 2.72 2.70 2.69 2.63 2.63 2.62 2.61 2.61 2.61 2.54 2.54 2.54 2.54 2.51 2.51 2.51 2.51 2.33 2.32 2.32 2.31 2.30 2.30 2.24 2.24 2.24 2.23 2.23 2.23 2.22 2.21 1.89 1.89 1.88 1.88 1.30 1.29
H
O
Me
H
Me
O
O
S7
^{1}H NMR (700 MHz, $CDCl_3$)
1.00 1.04 0.99 1.03 1.08 1.04 1.08 2.03 2.20 3.06 1.13 2.99
10.5 10.0 9.5 9.0 8.5 8.0 7.5 7.0 6.5 6.0 5.5 5.0 4.5 4.0 3.5 3.0 2.5 2.0 1.5 1.0 0.5 0.0 -0.5
δ (ppm)

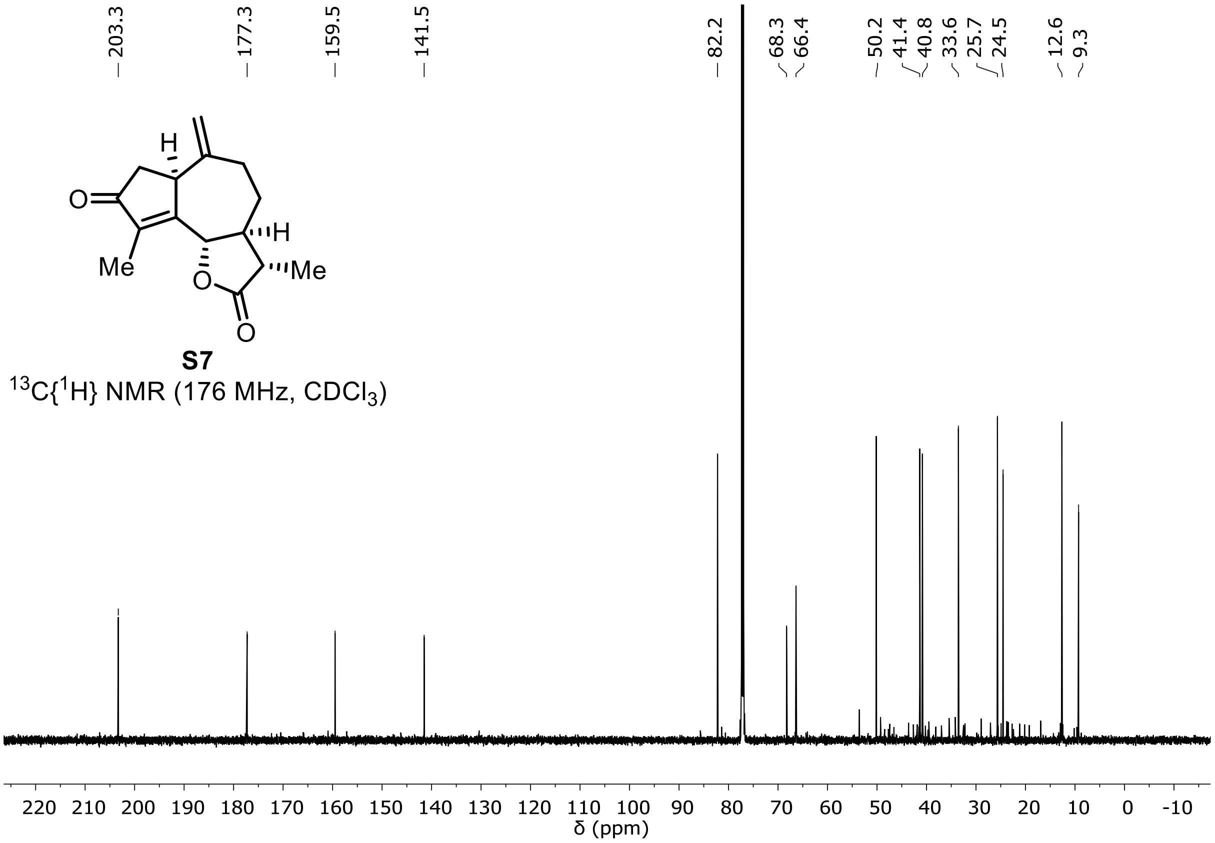
203.3 177.3 159.5 141.5 82.2 68.3 66.4 50.2 41.4 40.8 33.6 25.7 24.5 12.6 9.3
H
O
Me
H
Me
O
O
S7
^{13}C{^{1}H} NMR (176 MHz, $CDCl_3$)
220 210 200 190 180 170 160 150 140 130 120 110 100 90 80 70 60 50 40 30 20 10 0 -10
δ (ppm)

6. References

(1) Chu, H.; Smith, J. M.; Felding, J.; Baran, P. S. Scalable Synthesis of (−)-Thapsigargin. *ACS Cent. Sci.* **2017**, *3*, 47–51.

(2) Castellanos, L.; Duque, C.; Rodríguez, J.; Jiménez, C. Stereoselective Synthesis of (–)-4-Epi-axinyssamine. *Tetrahedron* **2007**, *63*, 1544–1552.

(3) Horn, E. J.; Rosen, B. R.; Chen, Y.; Tang, J.; Chen, K.; Eastgate, M. D.; Baran, P. S. Scalable and Sustainable Electrochemical Allylic C–H Oxidation. *Nature* **2016**, *533*, 77–81.

(4) Tenius, B. S. M.; Rohde, A. R.; Victor, M. M.; Viegas, C. An Efficient and Stereoselective Synthesis of (+)-α-Cyperone. *Synth. Commun.* **1996**, *26*, 197–203.

(5) Foster, S. J.; Rees, C. W. 3aH-Indenes. Part 4. Formation and Reactions of Some Dienone Intermediates. *J. Chem. Soc. Perkin 1* **1985**, 719.

(6) Piers, E.; Cheng, K. F. Conversion of (+)-α-Cyperone and (–)-7-Epi- *α* -Cyperone into Hydroazulenederivatives. *Can. J. Chem.* **1967**, *45*, 1591–1595.

(7) Dong, S.-H.; Duan, Z.-K.; Ai, Y.-F.; Zhou, X.-F.; Zhang, X.; Lian, M.-Y.; Huang, X.-X.; Bai, M.; Song, S.-J. Guaiane-Type Sesquiterpenoids with Various Ring Skeletons from Daphne Bholua Uncovered by Molecular Networking and Structural Revisions of Previously Reported Analogues. *Bioorg. Chem.* **2022**, *129*, 106208.

(8) Manzano, F. L.; Guerra, F. M.; Moreno-Dorado, F. J.; Jorge, Z. D.; Massanet, G. M. Toward the Synthesis of Thapsigargin: Enantioselective Synthesis of 7,11-Dihydroxyguaianolides. *Org. Lett.* **2006**, *8*, 2879–2882.

(9) Macías, F. A.; Santana, A.; Yamahata, A.; Varela, R. M.; Fronczek, F. R.; Molinillo, J. M. G. Facile Preparation of Bioactive *Seco* -Guaianolides and Guaianolides from *Artemisia Gorgonum* and Evaluation of Their Phytotoxicity. *J. Nat. Prod.* **2012**, *75*, 1967–1973.

(10) Zhang, W.; Luo, S.; Fang; Chen, Q.; Hu, H.; Jia, X.; Zhai, H. Total Synthesis of Absinthin. *J. Am. Chem. Soc.* **2005**, *127*, 18–19.

(11) Qi, X.-L.; Zhang, J.-T.; Feng, J.-P.; Cao, X.-P. Total Synthesis and Absolute Configuration of Malyngamide W. *Org. Biomol. Chem.* **2011**, *9*, 3817–3824.

(12) Shao, L.-D.; Chen, Y.; Wang, M.; Xiao, N.; Zhang, Z.-J.; Li, D.; Li, R.-T. Palladium-Catalyzed Direct γ-C(sp^3)–H Arylation of β-Alkoxy Cyclohexenones: Reaction Scope and Mechanistic Insights. *Org. Chem. Front.* **2022**, *9*, 2308–2315.

(13) Pinkerton, D. M.; Vanden Berg, T. J.; Bernhardt, P. V.; Williams, C. M. Gaining Synthetic Appreciation for the Gedunin ABC Ring System. *Chem. Eur. J.* **2017**, *23*, 2282–2285.

(14) Agami, C.; Meynier, F.; Puchot, C.; Guilhem, J.; Pascard, C. Stereochemistry-59: New Insights into the Mechanism of the Proline-Catalyzed Asymmetric Robinson Cyclization; Structure of Two Intermediates. Asymmetric Dehydration. *Tetrahedron* **1984**, *40*, 1031–1038.

(15) Schneider, L. M.; Schmiedel, V. M.; Pecchioli, T.; Lentz, D.; Merten, C.; Christmann, M. Asymmetric Synthesis of Carbocyclic Propellanes. *Org. Lett.* **2017**, *19*, 2310–2313.

(16) Hardouin, C.; Chevallier, F.; Rousseau, B.; Doris, E. Cp_2TiCl-Mediated Selective Reduction of α,β-Epoxy Ketones. *J. Org. Chem.* **2001**, *66*, 1046–1048.

(17) Engman, L.; Stern, D. Thiol/Diselenide Exchange for the Generation of Benzeneselenolate Ion. Catalytic Reductive Ring-Opening of .alpha.,.beta.-Epoxy Ketones. *J. Org. Chem.* **1994**, *59*, 5179–5183.

(18) Greene, A. E. Highly Stereoselective Total Syntheses of (+)-Pachydictyol A and (–)-Dictyolene, Novel Marine Diterpenes from Brown Seaweeds of the Family Dictyotaceae. *J. Am. Chem. Soc.* **1980**, *102*, 5337–5343.
